# The Plant Homeodomain Protein Clp1 Regulates Fungal Development, Virulence, and Autophagy Homeostasis in Magnaporthe oryzae

**DOI:** 10.1128/spectrum.01021-22

**Published:** 2022-08-29

**Authors:** Jing Wang, Zhicheng Huang, Pengyun Huang, Qing Wang, Yan Li, Xiao-Hong Liu, Fu-Cheng Lin, Jianping Lu

**Affiliations:** a State Key Laboratory for Managing Biotic and Chemical Threats to the Quality and Safety of Agro-products, College of Life Sciences, Zhejiang University, Hangzhou, Zhejiang Province, China; b State Key Laboratory for Managing Biotic and Chemical Threats to the Quality and Safety of Agro-products, Institute of Plant Protection and Microbiology, Zhejiang Academy of Agricultural Sciences, Hangzhou, Zhejiang Province, China; c Biotechnology Institute, Zhejiang University, Hangzhou, Zhejiang Province, China; Yeungnam University

**Keywords:** Cti6, hyperbranch, *Pyricularia oryzae*, autophagosome, pathogenicity, rice blast fungus, Atg5, PAS, appressorium, asexual development, gene transcription, plant homeodomain

## Abstract

Rice blast disease caused by Magnaporthe oryzae is a serious threat to global grain yield and food security. Cti6 is a nuclear protein containing a plant homeodomain (PHD) that is involved in transcriptional regulation in Saccharomyces cerevisiae. The biological function of its homologous protein in M. oryzae has been elusive. Here, we report Clp1 with a PHD domain in M. oryzae, a homologous protein of the yeast Cti6. Clp1 was mainly located in the nucleus and partly in the vesicles. Clp1 colocalized and interacted with the autophagy-related proteins Atg5, Atg7, Atg16, Atg24, and Atg28 at preautophagosomal structures (PAS) and autophagosomes, and the loss of Clp1 increased the fungal background autophagy level. Δ*clp1* displayed reduced hyphal growth and hyperbranching, abnormal fungal morphology (including colony, spore, and appressorium), hindered appressorial glycogen metabolism and turgor production, weakened plant infection, and decreased virulence. The PHD is indispensable for the function of Clp1. Therefore, this study revealed that Clp1 regulates development and pathogenicity by maintaining autophagy homeostasis and affecting gene transcription in M. oryzae.

**IMPORTANCE** The fungal pathogen Magnaporthe oryzae causes serious diseases of grasses such as rice and wheat. Autophagy plays an indispensable role in the pathogenic process of M. oryzae. Here, we report a Cti6-like protein, Clp1, that is involved in fungal development and infection of plants through controlling autophagy homeostasis in the cytoplasm and gene transcription in the nucleus in M. oryzae. This study will help us to understand an elaborated molecular mechanism of autophagy, gene transcription, and virulence in the rice blast fungus.

## INTRODUCTION

The rice blast pathogen Magnaporthe oryzae (synonym, Pyricularia oryzae) is a filamentous ascomycete fungus that infects plants via a specialized infection cell called the appressorium (1). After an asexual spore attaches onto the rice leaf surface, it germinates and forms an incipient appressorium from a germ tube as a response to environmental signals (2, 3). During appressorium maturation, the appressorial cell wall is melanized (4), nutrient stores, including glycogen and lipids, are transferred from conidial cells into an appressorial cell, and glycerol accumulates in the appressorium, which is required for the generation of huge turgor pressure (5). M. oryzae utilizes appressorial turgor of up to 8.0 megapascal (MPa) to rupture the rice cell cuticle and form a penetration peg. Subsequently, the penetration peg differentiates into infectious hyphae, which expand and cause disease lesions (6–8).

Autophagy is a highly conserved protective mechanism that occurs under starvation or environmental stress in eukaryotic organisms (9, 10). Upon autophagy induction, cytosolic organelles and proteins are sequestered within autophagosomes with double-layered membranes and are delivered to vacuoles or lysosomes for degradation (11, 12). As excessive or limited autophagy is adverse for cell function and survival, the regulation of autophagy homeostasis is necessary (13, 14). In M. oryzae, previous studies have shown that autophagy-related (ATG) genes play crucial roles in growth, conidiation, appressorium formation, and virulence (15–17). Autophagy is regulated by various pathways, including signaling, metabolism, and posttranslational modification. The target of rapamycin (TOR) signaling pathway is essential for cell growth and autophagy regulation (18, 19). A StAR-related lipid transfer (VASt) domain-containing protein, Vast1, negatively regulates autophagy by promoting TOR kinase activity (20). Opy2, an Mst50 interactor, is involved in the Osm1 mitogen-activated protein kinase (MAPK) and Mps1 MAPK pathways and negatively regulates autophagy (21). Autophagy is linked to CWI signaling in response to endoplasmic reticulum (ER) stress (22). Posttranslational modification and transcriptional regulation are also important ways to maintain autophagy homeostasis (23–26). In murine cells, Gadd45β-MEKK4-mediated p38 activation phosphorylates Atg5 at Thr75 to inhibit autophagosome maturation under starvation-induced autophagy (27). In human cells, the transcription factor FoxO1 shuttles between the nucleus and cytoplasm, and acetylated cytosolic FoxO1 binds to Atg7 to induce the autophagic process under stress (28). In M. oryzae, a histone acetyltransferase, MoHat1, acetylates MoAtg3 and MoAtg9 to regulate appressorium development and autophagy (29).

Cti6 (Cyc8-Tup1 interacting protein 6) is a nuclear protein containing a conserved plant homeodomain (PHD) in yeast (30, 31). In humans, the PHD of Cti6 contains a Zn finger motif of the (Cys)4-His-(Cys)2 type and is involved in transcriptional regulation (32, 33). In Saccharomyces cerevisiae, Cti6 directly interacts with Cyc8 of the Cyc8-Tup1 corepressor complex and cooperates with the SAGA (Spt-Ada-Gcn5-acetyltransferase) complex to relieve Cyc8-Tup1-mediated transcriptional inhibition (30). Furthermore, Cti6 is associated with the Rpd3-Sin3 histone deacetylase (HDAC) complex and regulates the growth of S. cerevisiae under iron-limiting conditions and the regulation of telomeric silencing (31). In the filamentous fungus Trichoderma reesei, Clp1, a Cti6-like protein, is involved in the regulation of cellulase gene expression and sporulation (34). In another filamentous fungus, Aspergillus flavus, deletion of the *CTI6* or PHD of *CTI6* resulted in alterations in mycelial morphology, slowed growth, and reduced sporulation and decreased the biosynthesis of aflatoxin B1 (AFB1) (35).

Although the functions of Cti6 have been studied in yeast and other organisms, its biological role in M. oryzae, especially in autophagy, has not been determined. In this study, we identified and characterized the roles of *CLP1* by knocking it out in M. oryzae. Clp1 is mainly localized in the nucleus and partly in preautophagosomal structures (PAS) and autophagosomes around the nucleus and participates in hyphal branching and growth, conidiation, appressorium formation, and virulence. Importantly, Clp1 affects the background autophagy level by interacting with Atg5, Atg7, Atg16, Atg24, and Atg28 in PAS and autophagosomes around the nucleus of M. oryzae.

## RESULTS

### Clp1, a Cti6-like protein in M. oryzae, has different functions than Cti6 in yeast.

In S. cerevisiae, Cti6 is a transcriptional repressor that contains a plant homeodomain (PHD) (31, 32). We identified a Cti6-like protein (MGG_02535/GenBank accession no. XP_003721271) in M. oryzae by BLASTP in NCBI (https://blast.ncbi.nlm.nih.gov/). MGG_02535 contained a PHD protein homologous to Cti6 of yeast with 47.83% sequence identity, homologous to Clp1 of Trichoderma reesei with 50.23% identity, and homologous to Cti6 of A. flavus with 43.27% identity (see Fig. S1 in the supplemental material). Here, MGG_02535 was named Cti6-like protein Clp1 in M. oryzae. To reveal the biological function of Clp1 in M. oryzae, we knocked out *CLP1* in the wild-type strain 70-15 using a previously reported DNA homologous recombination method (36, 37) (Fig. S2; Table S1) and assayed the mutant phenotype of Δ*clp1*.

**FIG 1:**
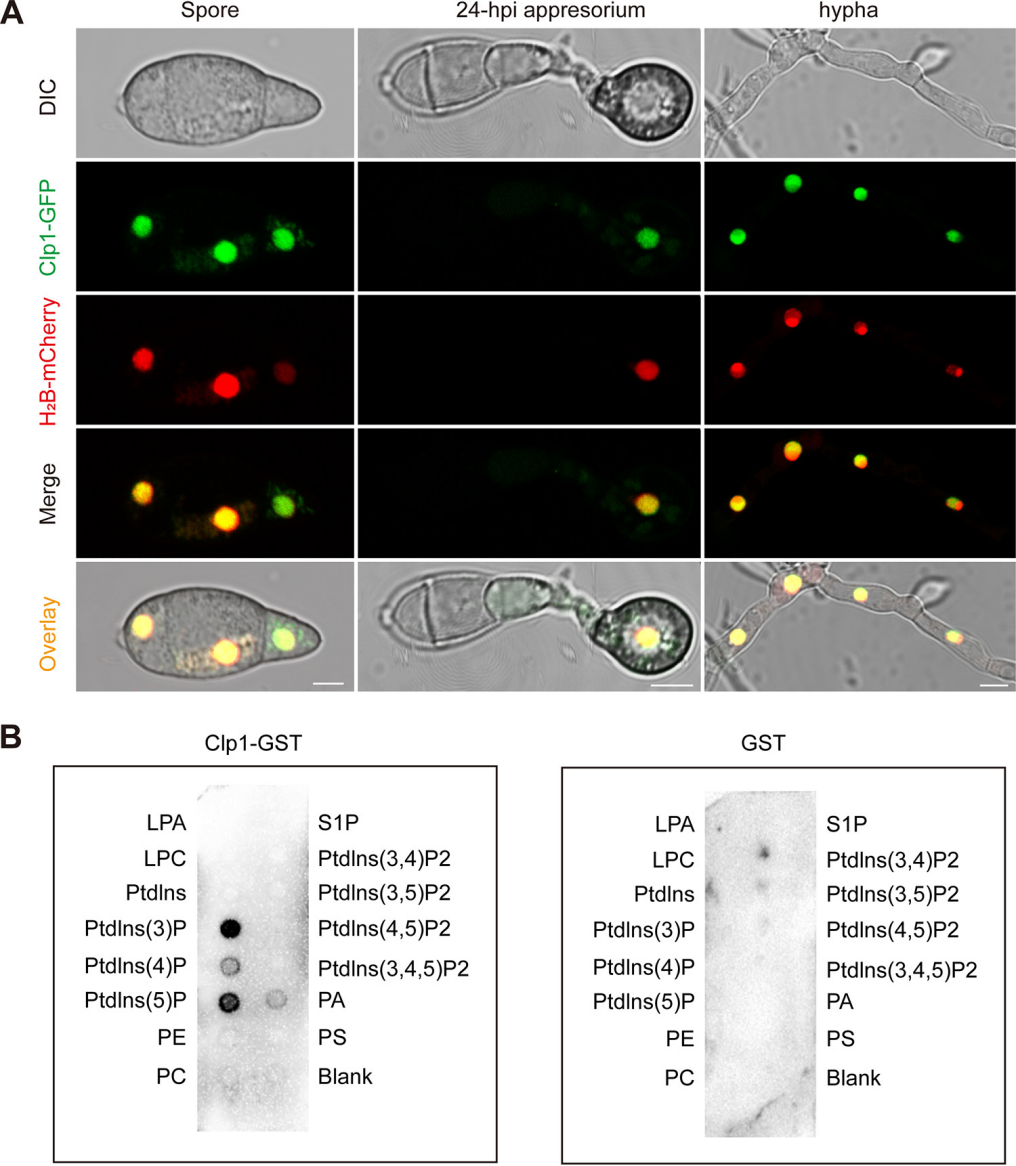
Subcellular localization of Clp1; Clp1 binds with PtdIns(3)P and PtdIns(5)P in M. oryzae. (A) Clp1-GFP and H_2_B-mCherry were coexpressed in Δ*clp1*. Appressoria were induced on hydrophobic borosilicate glass coverslips at 22°C for 24 h. Hyphae were formed by culturing spores in liquid CM at 25°C for 24 h. Bar = 5 μm. (B) Clp1 binds with PtdIns(3)P and PtdIns(5)P. For the protein lipid overlay assay, purified Cti6-GST protein and PIP Strips were coincubated overnight at 4°C. The binding proteins were detected by Western blotting with an anti-GST antibody. Purified GST was used as a negative control. LPA, lysophosphatidic acid; LPC, lysophosphocholine; PtdIns, phosphatidylinositol; PE, phosphatidylethanolamine; PC, phosphatidylcholine; S1P, sphingosine-1-phosphate; PA, phosphatidic acid; PS, phosphatidylserine.

Cti6 in yeast is a nuclear protein that interacts directly with Rpd3 and Sin3 of the histone deacetylase complex and Cyc8 of the cyc8-tup1 corepressor complex (30, 31). To characterize the cellular localization of Clp1 in M. oryzae, we constructed a Clp1-GFP fusion protein-expressing vector and an H_2_B-mCherry fusion protein-expressing vector and cotransformed them into Δ*clp1*. Under confocal fluorescence microscopy, we observed the colocalization of Clp1-GFP and H_2_B-mCherry at spores, appressoria, and hyphae. In hyphae, conidia, and appressoria, Clp1-GFP was mainly localized in the nucleus and overlapped with H_2_B-mCherry. Some Clp1-GFP signals appeared in the cytoplasm, displaying a dotted pattern around the nucleus (Fig. 1A). We then tested the interactions between Clp1 and Cyc8 and Rpd3 and Sin3 in M. oryzae using a yeast two-hybrid assay (Y2H). The Y2H results showed that Clp1 did not interact with Cyc8, Rpd3, or Sin3 in M. oryzae (Fig. S3). In yeast, Cti6 mediates the assembly of the Cti6-Cyc8-Tup1 coactivator complex by binding with endosomal phosphatidylinositol 3,5-phosphate [PI(3,5)P2] (38). In M. oryzae, the protein lipid overlay assay showed that the purified Clp1-glutathione transferase (GST) significantly did not bind with PI(3,5)P2 but bound with phosphatidylinositol-3-phosphate (PI3P) and phosphatidylinositol-5-phosphate (PI5P) *in vitro* (Fig. 1B).

### Clp1 is involved in mycelial growth, conidiation, and conidial and appressorial morphology in M. oryzae.

**FIG 2:**
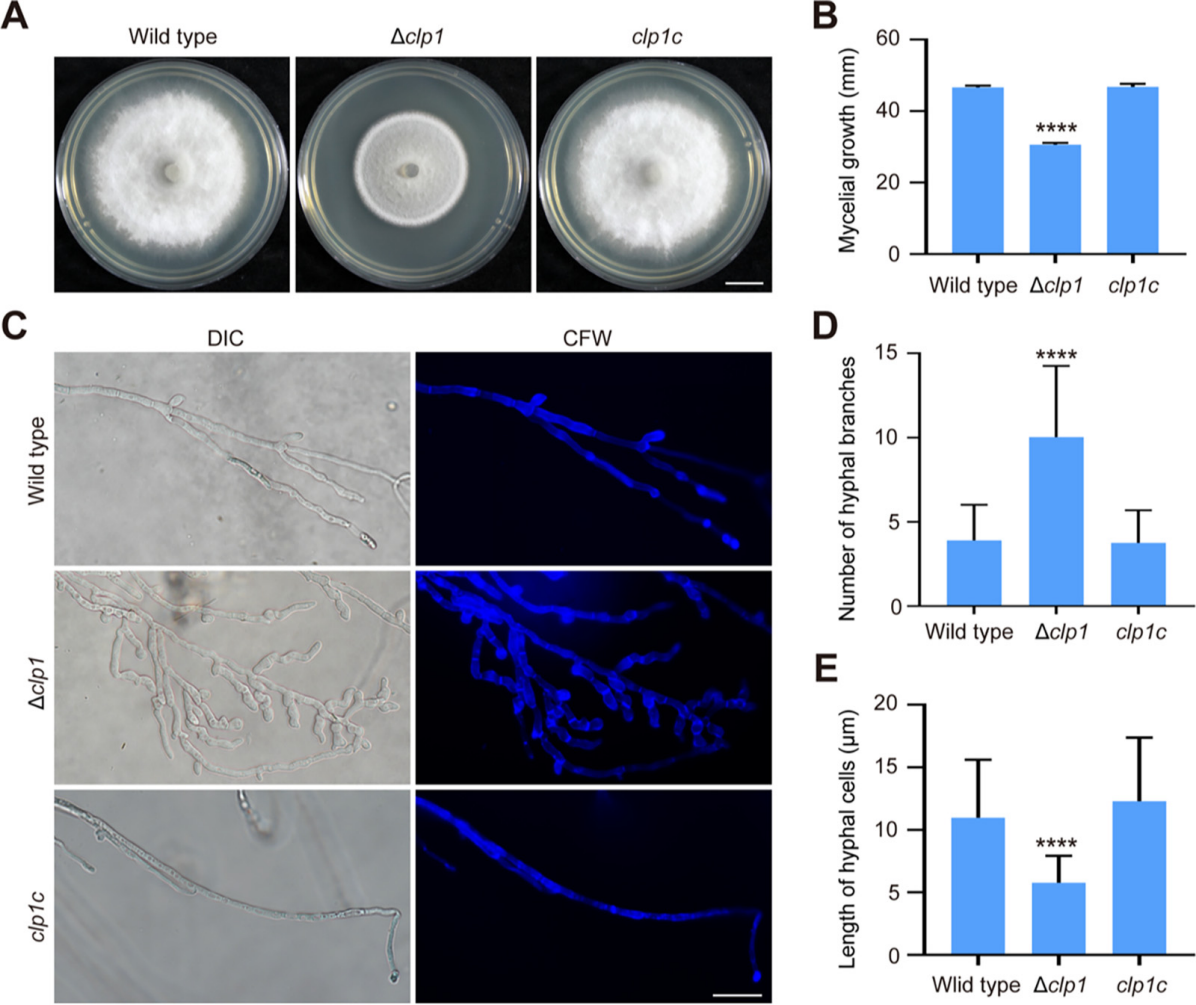
Mycelial growth and hyphal hyperbranching of Δ*clp1*. (A) The wild-type, Δ*clp1*, and complemented strain *clp1c* were cultured on CM for 7 days. Bar = 1 cm. (B) The mycelial diameters (cm) of the wild type, Δ*clp1*, and *clp1c* when cultured on CM for 7 days. (C) Hyphal branching of the wild type, Δ*clp1*, and *clp1c.* The spores were cultured in CM on a glass slide for 3 days, and the hyphal cell walls were stained with CFW. Bar = 10 μm. (D) The number of branches was counted in a section of 0.5-mm long hyphae starting from the apex. (E) The length of hyphal cells was measured with NIS-Elements D 3.2 software. Asterisks indicate statistically significant differences between Δ*clp1* and the wild type (****, *P *< 0.0001).

**FIG 3:**
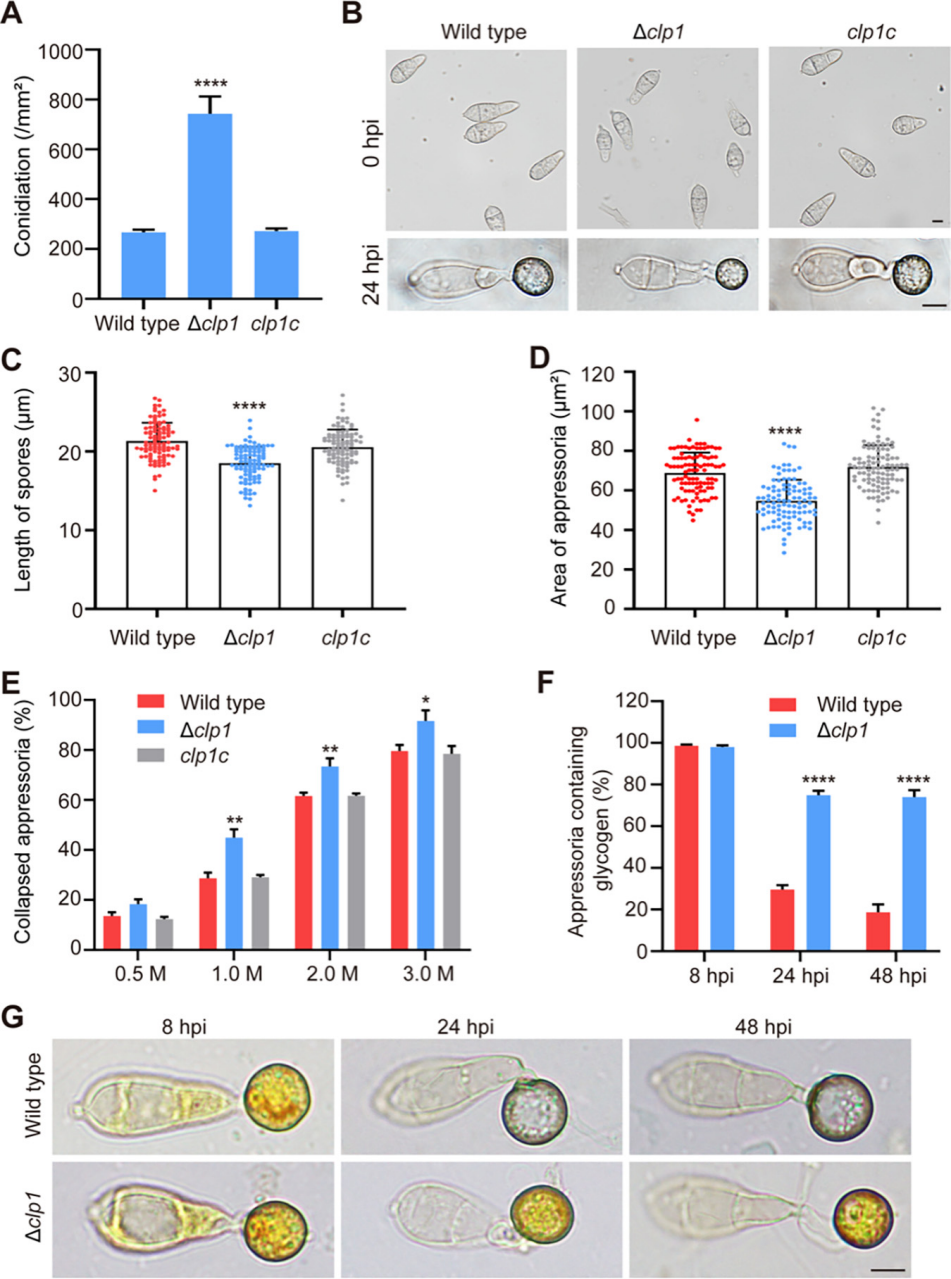
Conidiation and appressorium formation of Δ*clp1*. (A) The conidiation of the wild type, Δ*clp1*, and *clp1c*. (B) The morphology of spores (0 hpi) and appressoria (24 hpi). Bars = 5 μm. (C) The length of spores was measured by NIS-Elements D 3.2 software. (D) The area of appressoria at 24 hpi. (E) The cytorrhysis analysis of appressoria under a series of concentrations of glycerol (0.5 M to 3 M). (F and G) Observation and quantification of intracellular glycogen storage and degradation in appressoria at 8, 24, and 48 hpi. Bar = 5 μm. Asterisks indicate statistically significant differences between the wild type and Δ*clp1* (*, *P* < 0.05; **, *P* < 0.01; ****, *P* < 0.0001).

The Δ*clp1* mutant grew slower on complete medium (CM), and its colonies were blacker and had denser hyphae than the wild type (Fig. 2A and B). When we observed hyphal branching, the number of hyphal branches per unit length in Δ*clp1* was greater than that in the wild type, displaying a hyperbranching phenotype (Fig. 2C and D). We stained hyphal cell walls with calcofluor white (CFW) and measured the lengths of the hyphal cells. The length of hyphal cells in Δ*clp1* was significantly shorter than that in the wild type (Fig. 2C and E). Relative to the wild type, Δ*clp1* produced more conidia per unit colony area (Fig. 3A). The conidial germination rate and the appressorium formation rate of Δ*clp1* were comparable with those of the wild type (Fig. S4A and B). However, the conidial length was shorter and the appressorial area was smaller in Δ*clp1* than in the wild type and the complemented strain *clp1c* (Fig. 3B to D).

The rice blast fungus penetrated the plant cuticle via the mechanical force generated by appressorial turgor. We evaluated the appressorial turgor of Δ*clp1* using the incipient collapse assay (6, 39). Upon immersion in a series of concentrations of glycerol solution (0.5 M to 3.0 M), the Δ*clp1* mutant had a higher rate of collapsed appressoria (Fig. 3E), suggesting that the appressorial turgor of Δ*clp1* was reduced. As glycogen and lipids are two carbon sources that are metabolized to generate appressorial turgor (5, 40), we assayed the contents of glycogen granules and lipid droplets in appressoria by staining with iodine potassium iodide solution (I_2_/KI) for glycogen granules or BODIPY (boron pyrromethene dye) for lipid droplets. The results showed that during the appressorium development of Δ*clp1*, the translocation of glycogen granules from conidial cells to appressorial cells was normal at 8 hpi, while the degradation of glycogen in appressorial cells was hindered (Fig. 3F and G). At 24 hpi, 74.97% of appressoria contained glycogen granules in Δ*clp1*, while 29.63% of appressoria contained glycogen granules in the wild type, and at 48 hpi, 74.04% of the Δ*clp1* appressoria were still filled with glycogen granules (Fig. 3F and G). The translocation and degradation of lipid droplets during appressorium formation of Δ*clp1* were comparable with those of the wild type (Fig. S4C).

**FIG 4:**
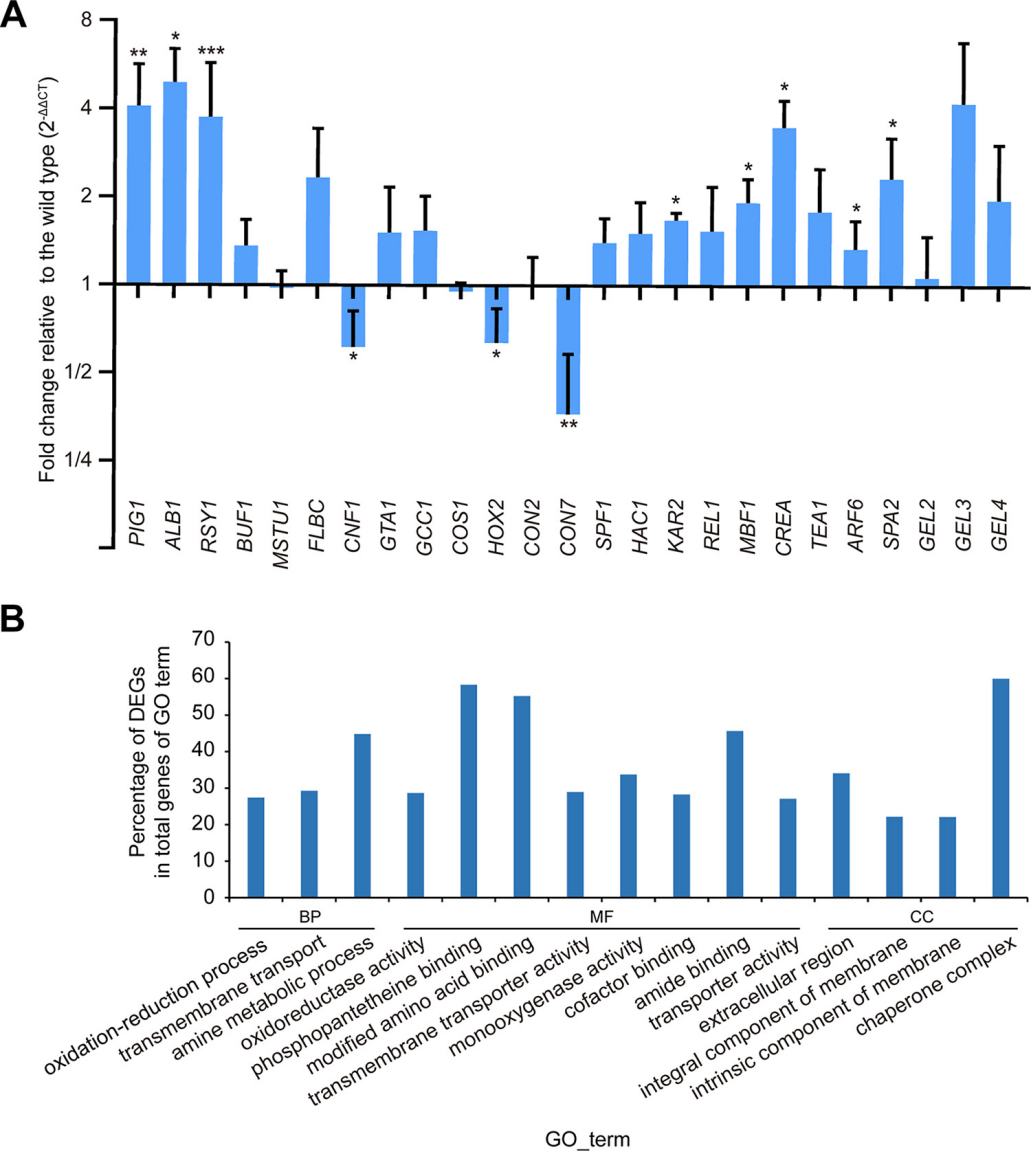
Relative expression levels of genes in Δ*clp1* compared with the wild type. (A) The expression levels of conidiation-related genes (*PIG1*, *RSY1*, *ALB1*, *BUF1*, *MSTU1*, *FLBC*, *CNF1*, *GTA1*, *GCC1*, *COS1*, *HOX2*, *CONX2*, and *CON7*), growth-related genes (*SPF1*, *HAC1*, *KAR2*, *REL1*, *MBF1*, and *CREA*), and hyperbranching phenotype-related genes (*TEA1*, *ARF6*, *SPA2*, *GEL2*, *GEL3*, and *GEL4*) were quantified using qPCR in the wild type and Δ*clp1*. *40S* and *α-ACTIN* were used as reference genes. Asterisks indicate statistically significant differences between the wild type and Δ*clp1* (*, *P* < 0.05; **, *P* < 0.01; ***, *P* < 0.001). (B) Enriched GO terms in aerial mycelial differentially expressed genes (DEGs) in Δ*clp1* by RNA-seq (*P* < 0.01).

In M. oryzae, some known genes are involved in fungal conidiation (*PIG1*, *RSY1*, *ALB1*, and *BUF1* [41, 42], *MSTU1* [43], *CNF1*, *GTA1*, *GCC1* [36], *COS1* [44], *HOX2* [45], and *FLBC*, *CONX2*, and *CON7* [46]), growth (*SPF1* [47], *HAC1* [48], *KAR2* [49], *MBF1* [50], and *REI1* and *CREA* [46]), and hyperbranching phenotype (*TEA1* [51], *ARF6* [52], *SPA2* [53], and *GEL2*, *GEL3*, and *GEL4* [54]). We quantified the expression levels of these genes in the sporulating mycelia of Δ*clp1*. Relative to the wild type, three transcription factor genes (*CNF1*, *HOX2*, and *CON7*) were significantly downregulated in Δ*clp1*, while three melanin synthesis-related genes (*PIG1*, *RSY1*, and *ALB1*), a transcription factor gene (*CREA*), and four other genes (*KAR2*, *MBF1*, *ARF6*, and *SPA2*) were significantly upregulated in Δ*clp1* (Fig. 4A).

We further measured and analyzed the gene expression profiles of the aerial mycelia in Δ*clp1* and the wild-type strain using RNA sequencing (RNA-seq). Relative to the wild type, there were 2,501 differentially expressed genes (DEGs) (|log2 fold change| > 1, *P < *0.05) in the aerial mycelia of Δ*clp1*, of which 1,323 genes were upregulated and 1,178 genes were downregulated (Table S2). The number of DEGs in Δ*clp1* accounted for 19.50% of the 12,825 total genes in the M. oryzae (70-15) genome. The Gene Ontology (GO) enrichment analysis of DEGs showed that enriched terms or GO IDs (*P < *0.01) in the aerial mycelia of Δ*clp1* were “GO:0055114/oxidation-reduction process,” “GO:0055085/transmembrane transport,” and “GO:0009308/amine metabolic process” in the biological_process (BP) category; “GO:0016491/oxidoreductase activity,” “GO:0031177/phosphopantetheine binding,” “GO:0072341/modified amino acid binding,” “GO:0022857/transmembrane transporter activity,” “GO:0004497/monooxygenase activity,” “GO:0048037/cofactor binding,” “GO:0033218/amide binding,” and “GO:0005215/transporter activity” in the molecular_function (MF) category; and “GO:0005576/extracellular region,” “GO:0016021/integral component of membrane,” “GO:0031224/intrinsic component of membrane,” and “GO:0101031/chaperone complex” in the cellular_component (CC) category (Fig. 4B). These enriched GO terms suggested that differentially expressed genes in Δ*clp1* are involved in membrane, oxidation-reduction, and amine metabolism.

### Clp1 is required for full virulence in M. oryzae.

**FIG 5:**
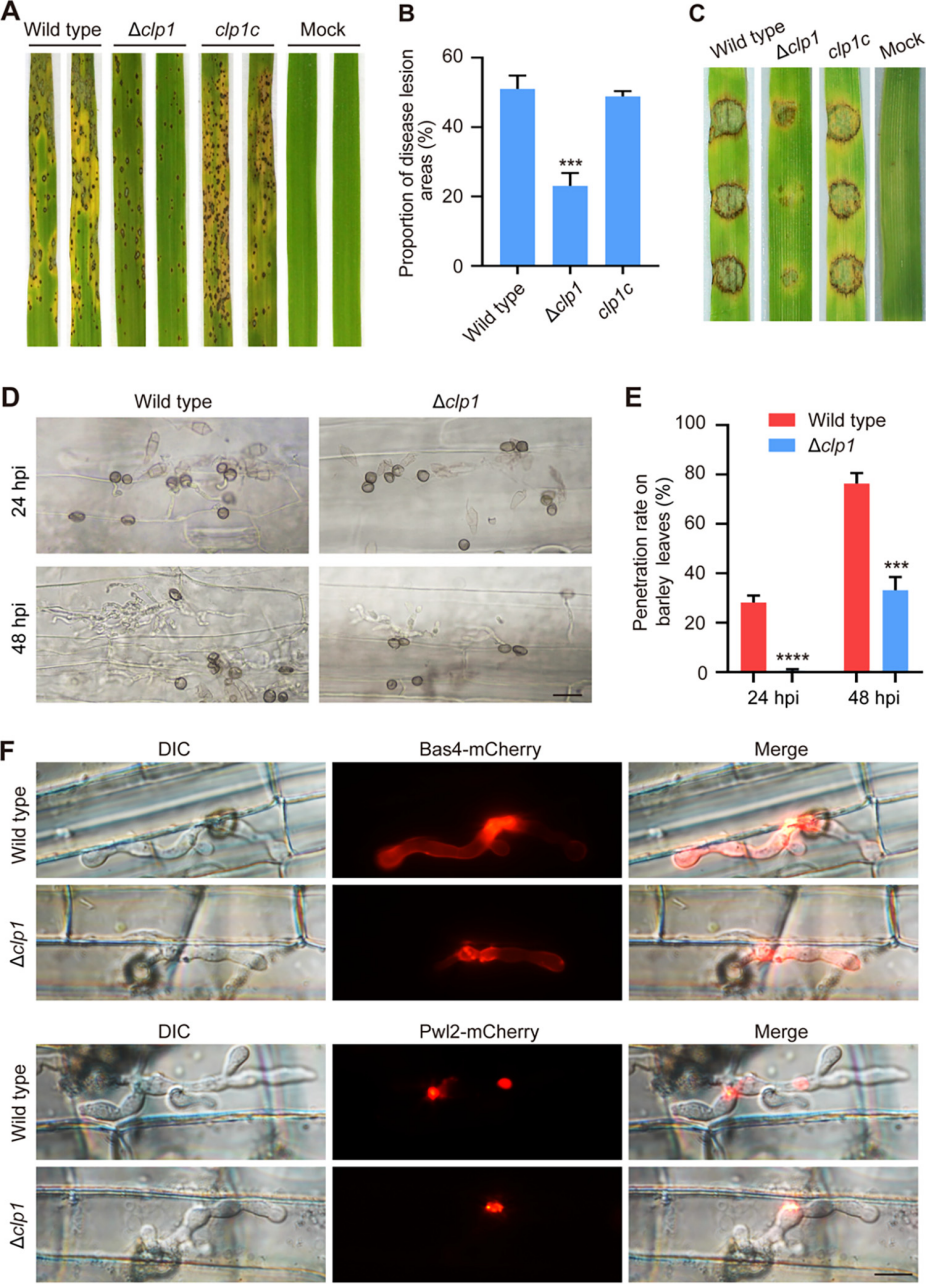
Pathogenicity and plant penetration assays of Δ*clp1*. (A) Rice seedlings (2 weeks old) were sprayed with conidial suspensions (5 × 10^4^ conidia mL^−1^) of the wild type, Δ*clp1*, and the complementation strain of Δ*clp1* (*clp1c*), and disease symptoms were observed at 5 to 7 dpi (day postinoculation). (B) The quantification of the disease severity in a 5-cm-long section of rice leaves. (C) The barley leaves *in vitro* were inoculated with mycelial blocks of the wild type, Δ*clp1*, and *clp1c* and photographed after 4 days at 25°C. (D) For infection observation, conidial suspensions (1 × 10^5^ conidia mL^−1^) of the wild type and Δ*clp1* were inoculated onto barley leaves for 24 and 48 hpi at 25°C. Bar =20 μm. (E) Penetration rates of appressoria were counted at 24 and 48 hpi. (B and E) Asterisks indicate statistically significant differences between the wild type and Δ*clp1* (***, *P* < 0.001; ****, *P* < 0.0001). (F) The conidial suspensions (1 × 10^5^ conidia mL^−1^) of the wild type and Δ*clp1* were injected into the rice leaf sheaths and incubated at 25°C, and the localization of Bas4-mCherry and Pwl2-mCherry was photographed at 32 hpi. Bar = 10 μm.

We assayed the functions of *CLP1* in the pathogenicity of rice and barley in M. oryzae. After spores (5 × 10^4^ conidia/mL^−1^) were sprayed on rice leaves, Δ*clp1* caused fewer lesions than the wild type, and its lesions expanded more slowly (Fig. 5A). The proportion of lesion areas caused by Δ*clp1* was 23.07%, while it was 51.03% in the wild type (Fig. 5B). Meanwhile, when mycelial plugs were inoculated onto leaf explants of barley, Δ*clp1* caused weaker lesions than the wild type (Fig. 5C). The appressorial penetration and invasive growth of Δ*clp1* were determined by drop-inoculating the spore suspension (1 × 10^5^ conidia/mL^−1^) onto barley leaves and culturing for 24 to 48 h in a moisture chamber. The results showed that appressorial penetration was delayed and invasive growth was slowed in Δ*clp1* (Fig. 5D and E). At 24 hpi, appressoria of Δ*clp1* formed fewer penetration pegs, and its penetration rate was 0.62%, while the wild type had formed many nascent infective hyphae, and its penetration rate was 28.20%. At 48 hpi, the penetration rate of the wild type reached 76.41%, and their invasive hyphae had expanded into 2 to 3 neighboring cells, while Δ*clp1* had a 33.17% penetration rate, and most of the invasive hyphae were limited to the first cell (Fig. 5D and E).

Effectors play important roles in M. oryzae successfully infecting plants and inhibiting the innate immunity of plants (55). To further explore whether the reduced virulence of Δ*clp1* was related to defects in effector secretion, we observed the localization of two effectors, apoplastic effector Bas4 and cytoplasmic effector Pwl2, in the wild type and Δ*clp1* during infection. The results showed that deletion of *CLP1* did not affect the secretion of either Bas4 or Pwl2 (Fig. 5F). When inoculating spores on rice sheaths for 32 h, Δ*clp1* and the wild type showed similar localization patterns of Bas4 and Pwl2. The Bas4-mCherry fusion protein was localized in the periphery of invasive hyphae named extrainvasive hyphal membrane (EIHM) structures, and the Pwl2-GFP fusion protein accumulated in biotrophic interfacial complex (BIC) structures (Fig. 5F).

### Clp1 is involved in the regulation of autophagy in M. oryzae.

**FIG 6:**
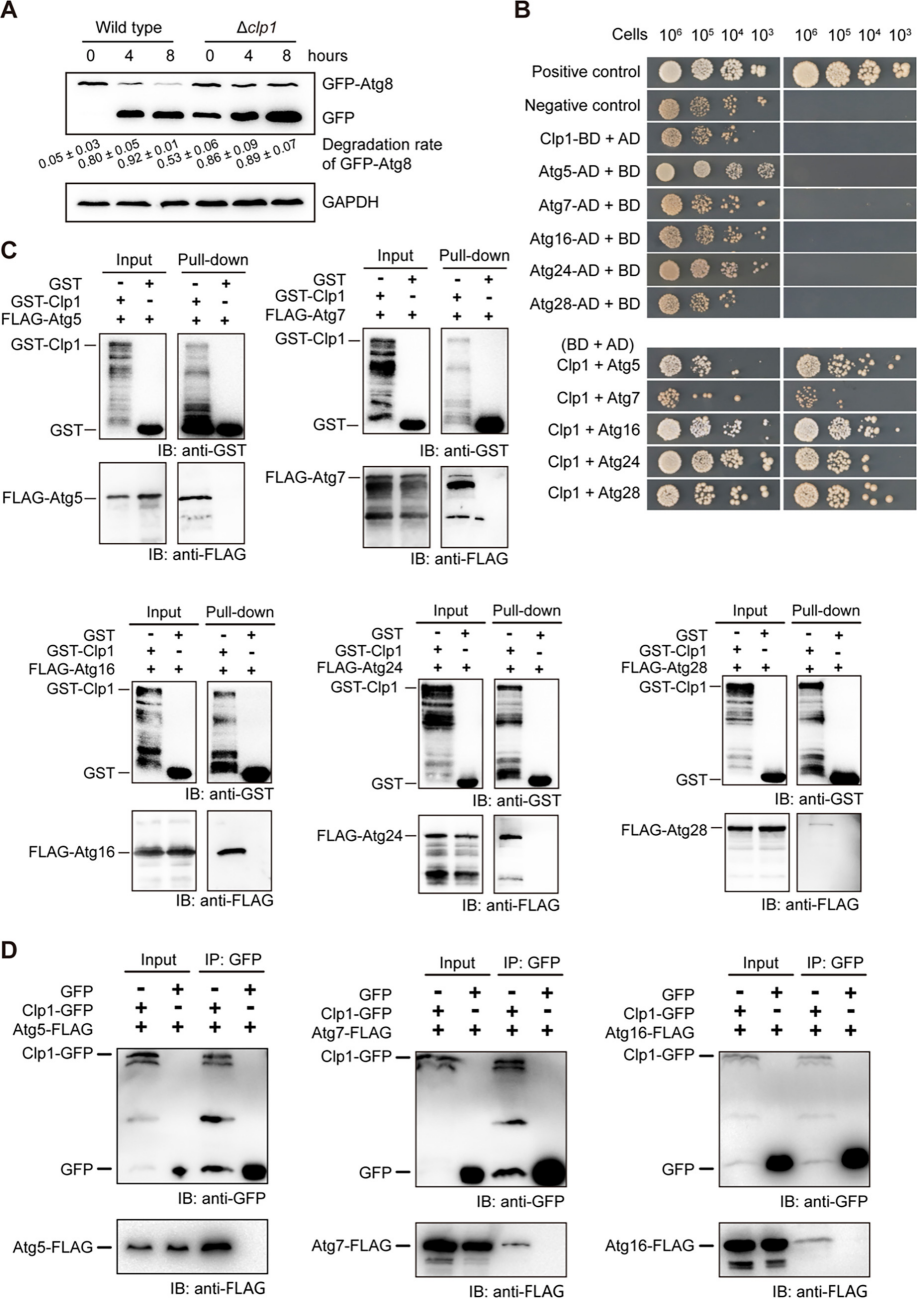
Autophagy detection and interaction assays between Clp1 and autophagy proteins. (A) Western blot analysis of GFP-Atg8 proteolysis in the wild type and Δ*clp1*. The mycelia were collected from CM and recultured in SD-N medium for 0 h (control), 4 h, and 8 h. Densitometric analysis was performed with ImageJ software. The degradation rates were calculated with the formula GFP/(GFP + GFP-Atg8). The mean ± SD was calculated from three replicates of Western blotting. (B) Yeast two-hybrid assays were used to verify the interactions between Clp1 and Atg5, Atg7, Atg16, Atg24, and Atg28. (C) The interactions between Clp1 and Atg5, Atg7, Atg16, Atg24, and Atg28 were confirmed by pulldown assays. The molecular weights of the fusion proteins were 99.26 kDa (Clp1-GST), 36.64 kDa (Atg5-3 × FLAG), 82.19 kDa (Atg7-3 × FLAG), 25.47 kDa (Atg16-3 × FLAG), 58.16 kDa (Atg24-3 × FLAG), and 73.64 kDa (Atg28-3 × FLAG). (D) The interactions between Clp1 and Atg5, Atg7, or Atg16 were confirmed by co-IP assays. The molecular weights of the fusion proteins were 99.18 kDa (Clp1-GFP), 36.64 kDa (Atg5-3 × FLAG), 82.19 kDa (Atg7-3 × FLAG), and 25.47 kDa (Atg16-3 × FLAG).

In M. oryzae, autophagy is essential for appressorium turgor, glycogen degradation, and plant infection (15, 16, 56). As Δ*clp1* displayed reduced appressorium turgor, delayed glycogen degradation, and decreased virulence (Fig. 3E and F, Fig. 5A to E), we tested whether Clp1 regulates autophagy by monitoring the degradation of GFP-Atg8, a marker protein of autophagosome formation (17), in Δ*clp1*. The mycelia of the wild type and Δ*clp1* expressing green fluorescent protein (GFP)-Atg8 were cultured in SD-N medium (nitrogen starvation) for 0 h, 4 h, and 8 h. Western blot analysis showed that Δ*clp1* had higher background autophagy activity than the wild type (Fig. 6A). When hyphae grew in liquid CM, the wild type had a very low GFP-Atg8 degradation rate (free GFP/[free GFP + GFP-Atg8] = 0.05 ± 0.03). However, Δ*clp1* had a high GFP-Atg8 degradation rate (0.53 ± 0.06), suggesting that GFP-Atg8 was quickly degraded and free GFP accumulated in Δ*clp1*. After hyphae were transferred into and cultured in SD-N liquid medium for 4 hpi and 8 hpi, GFP-Atg8 degradation greatly increased in the wild type but increased to a lesser degree in Δ*clp1* (Fig. 6A).

**FIG 7:**
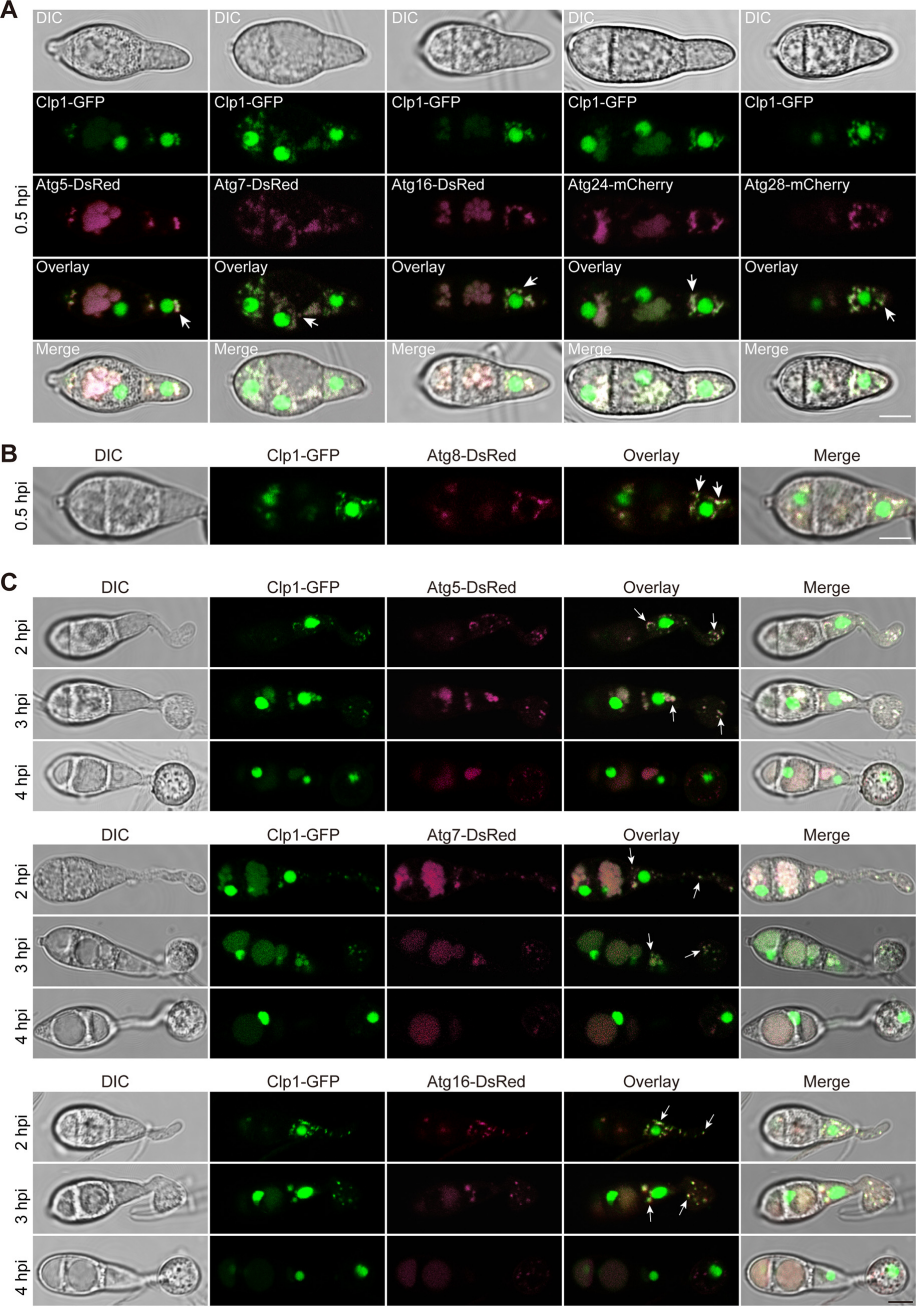
Colocalization assays between Clp1 and autophagy proteins in germinated spores and incipient appressoria. (A) The colocalization of Clp1-GFP with Atg5-DsRed, Atg7-DsRed, Atg16-DsRed, Atg24-mCherry, and Atg28-mCherry was observed after spores were incubated on hydrophobic borosilicate glass coverslips for 30 min. Bar = 5 μm. (B) The colocalization of Clp1-GFP with Atg8-DsRed at PAS or autophagosomes was observed after spores were incubated on hydrophobic borosilicate glass coverslips for 30 min. Bar = 5 μm. (C) The colocalization of Clp1-GFP with Atg5-DsRed, Atg7-DsRed, and Atg16-DsRed was observed after spores were incubated on hydrophobic borosilicate glass coverslips for 2 to 4 h. Bar = 5 μm. DsRed and mCherry were pseudocolored magenta. Arrows point to the sites in which Clp1 colocalized with Atg5, Atg7, Atg8, Atg16, Atg24, and Atg28.

To determine how Clp1 affects autophagy, we examined the interactions between Clp1 and autophagy-related proteins. Yeast two-hybrid experiments demonstrated that Atg5, Atg7, Atg16, Atg24, and Atg28 of 22 tested Atg proteins directly interacted with Clp1 (Fig. 6B). The pulldown experiments also confirmed these interactions between Clp1 and Atg5, Atg7, Atg16, Atg24, and Atg28 (Fig. 6C). We further confirmed the interaction of Clp1 with Atg5, Atg7, and Atg16 *in vivo* by coimmunoprecipitation (co-IP) experiments (Fig. 6D). Subsequently, we coexpressed Atg5-DsRed, Atg7-DsRed, Atg16-DsRed, Atg24-mCherry, or Atg28-mCherry with Clp1-GFP in Δ*clp1*. When spore suspensions were incubated on glass coverslips for 30 min, Atg5-DsRed, Atg7-DsRed, Atg16-DsRed, Atg24-mCherry, and Atg28-mCherry were colocalized with Clp1-GFP in small vesicles of the cytoplasm around the nucleus (Fig. 7A). To characterize these small vesicles, we colocalized Clp1-GFP and Atg8-DsRed in germinated spores at 0.5 hpi. The results showed that Clp1 was colocalized with Atg8 in small vesicles around the nucleus (Fig. 7B). We also stained germinated spores with CMAC (7-amino-4-chloromethylcoumarin), a fluorescent dye that monitors vacuoles, and found that CMAC-labeled large vacuoles colocalized with Atg5-DsRed, Atg7-DsRed, and Atg16-DsRed, whereas Atg5-DeRed, Atg7-DsRed, and Atg16-DsRed-labeled small vesicles were not stained by CMAC (Fig. S5), indicating that these small vesicles are not vacuoles. Atg8 is a mark of autophagosomes and their precursor structures (PAS and isolation membranes) (57). Therefore, the vesicles where Atg5, Atg7, Atg16, Atg24, and Atg28 colocalized with Clp1 are PAS and autophagosomes.

We observed changes of colocalization between Atg5, Atg7, Atg16, and Clp1 during spore germination and appressorium formation at 2 to 4 hpi. At 2 hpi, Clp1-GFP was localized in the vesicles (PAS or autophagosomes) marked by Atg5-DsRed, Atg7-DeRed, or Atg16-DsRed in spore cells, germ tubes, and expanding incipient appressoria. At 3 hpi, Clp1-GFP also colocalized with Atg5-DsRed, Atg7-dsRed, or Atg16-DsRed in the small vesicles of spore cells, germ tubes, and expanding incipient appressoria. However, at 4 hpi, Clp1-GFP was no longer clearly localized in vesicles labeled by Atg5-DsRed, Atg7-DsRed, or Atg16-DsRed (Fig. 7C). These results suggested that Clp1 was strongly involved in autophagy at the early stages of spore germination and appressorium formation.

### The PHD is vital for the function of Clp1.

**FIG 8:**
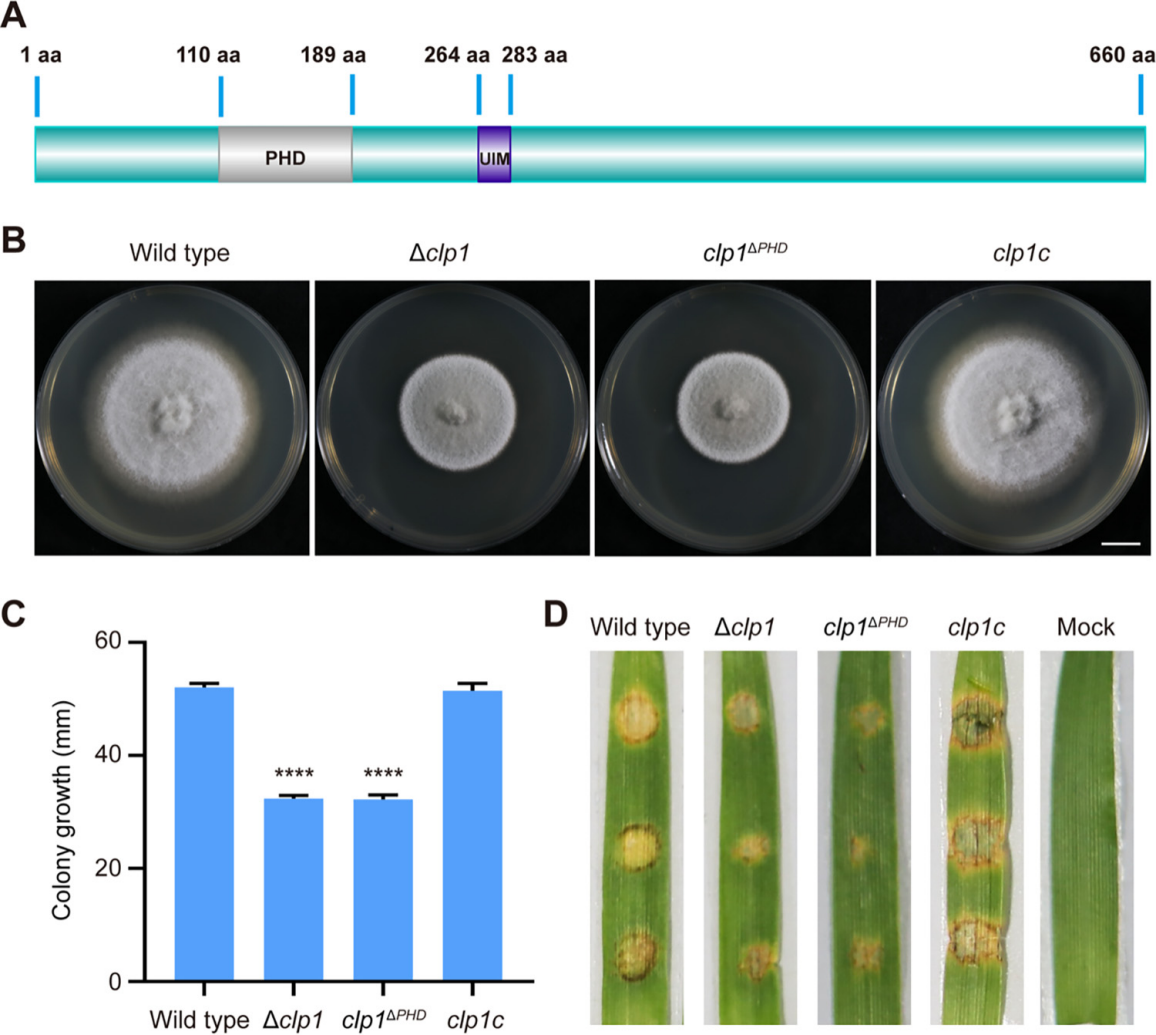
The PHD is vital for the function of Clp1. (A) A PHD and a UIM domain in Clp1. (B) Colony formation of the wild-type, Δ*clp1*, *clp1*^Δ^*^PHD^*, and complemented strain *clp1c*. Bar = 1 cm. (C) The growth of the four M. oryzae strains cultured in CM for 8 days. Asterisks indicate statistically significant differences between the wild type and the mutants (****, *P* < 0.0001). (D) Virulence assay. Mycelial blocks of the four M. oryzae strains were inoculated on barley leaves *in vitro* and cultured at 25°C for 4 days.

BLAST conserved domain (CD) search (https://www.ncbi.nlm.nih.gov/Structure/cdd/wrpsb.cgi) and InterPro search (https://www.ebi.ac.uk/interpro/) with Clp1 revealed that Clp1 had a PHD sequence between amino acids (aa) 110 and 189 and a ubiquitin-interacting motif (UIM) domain between aa 264 and 283 (Fig. 8A). We constructed a gene encoding a mutated Clp1 protein in which the PHD was deleted, transformed it into Δ*clp1*, and obtained the *clp1*^Δ^*^PHD^* mutant (Fig. S6). The colonial morphology, mycelial growth, and virulence of *clp1*^Δ^*^PHD^* were comparable with those of Δ*clp1* (Fig. 8B to D), indicating that the PHD of Clp1 plays a vital role in the biological functions of Clp1 in M. oryzae.

### Clp1 responds to oxidative and osmotic stress.

**FIG 9:**
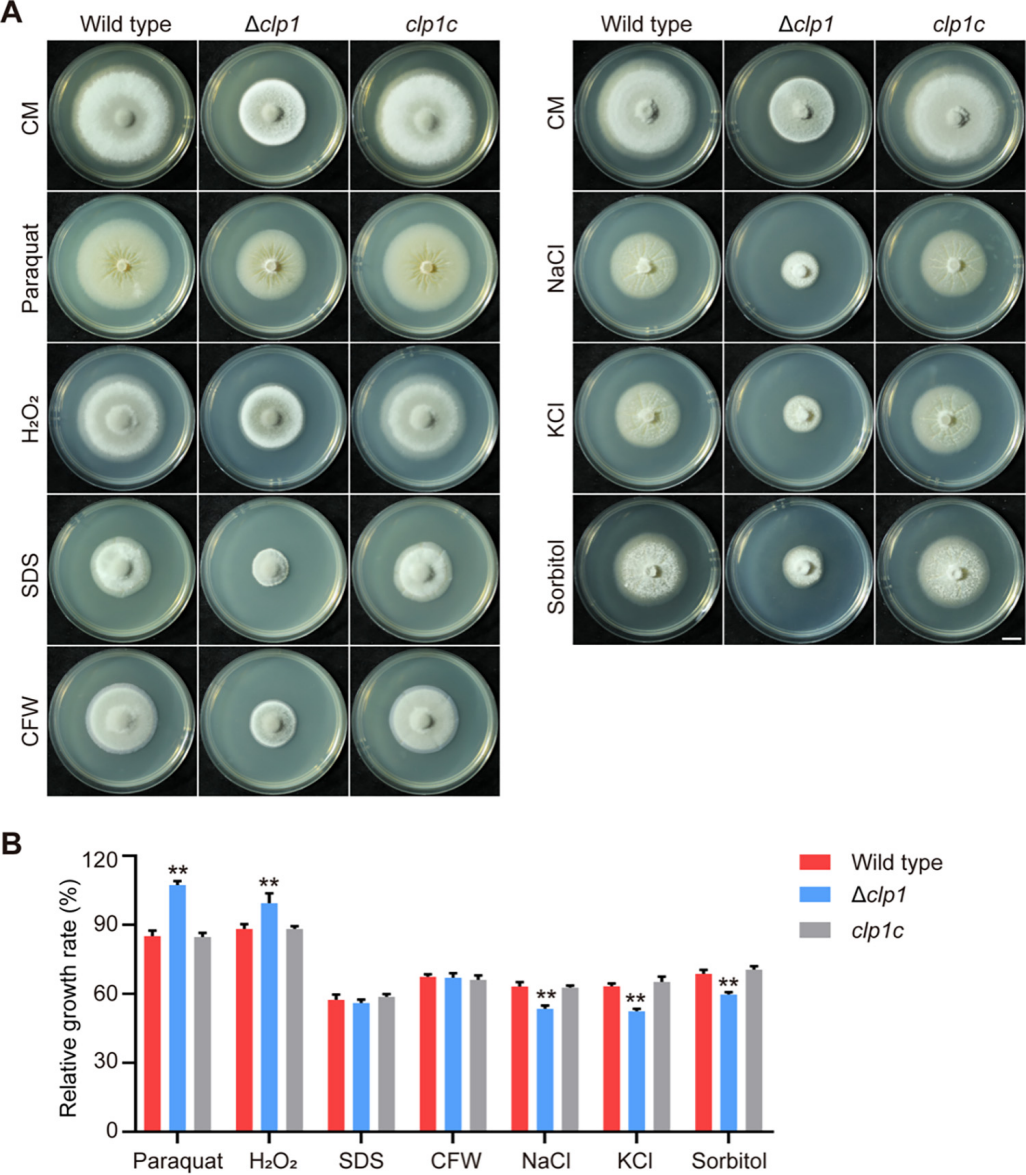
Growth of M. oryzae strains on stress media. (A) The colonies of the wild type, Δ*clp1*, and *clp1c* that were cultured on CM containing 1 mM paraquat, 8 mM hydrogen peroxide, 0.0055% SDS, or 75 μg/mL CFW under dark conditions and on CM containing 0.6 M NaCl, 0.6 M KCl, or 1 M sorbitol under light-dark (16 h/8 h) conditions at 25°C for 9 days. (B) The relative growth rate was determined by measuring colony diameters on stress media against the control medium (CM). Asterisks indicate statistically significant differences between the wild type and Δ*clp1* (**, *P* < 0.01).

We measured the growth of the wild type, Δ*clp1*, and *clp1c* on stress media containing oxidative stress factors (hydrogen peroxide or paraquat), cell wall stress factors (CFW or sodium dodecyl sulfate [SDS]), and osmotic stress factors (NaCl, KCl, or sorbitol) (Fig. 9). Δ*clp1* exhibited higher relative growth rates when exposed to hydrogen peroxide (H_2_O_2_) and paraquat than the wild type. The relative growth rates of Δ*clp1* on CFW and SDS were comparable to those of the wild type. However, the relative growth rates of Δ*clp1* on the three osmotic stress media were lower than those of the wild type (Fig. 9). These results indicated that Δ*clp1* is resistant to oxidative stresses but is sensitive to osmotic stresses.

## DISCUSSION

Magnaporthe oryzae is a filamentous fungus that infects rice through the appressorium and causes rice blast. Several PHD proteins, as epigenetic regulators, are involved in gene transcription in yeast, animals, and humans (58–60). In this study, we identified a PHD protein, Clp1, in M. oryzae and found that Clp1 regulates fungal autophagy, development, and virulence.

Clp1 is a homologous protein of Cti6, a PHD protein in yeast, with the highest sequence identity in M. oryzae. In S. cerevisiae, Cti6 plays a role in transcriptional regulation by interacting with Cyc8-Tup1 and HDAC complexes (30, 31). In M. oryzae, however, Y2H results showed that Clp1 did not directly interact with Cyc8 of the Cyc8-Tup1 corepressor complex or Rpd3 and Sin3 of the HDAC complex. In yeast, the Cti6-Cyc8-Tup1 complex is assembled in the late endosomal/vacuolar membrane after Cti6 binds to phosphatidylinositol 3,5-phosphate [PI(3,5)P2] and is then shuttled from the cytoplasm into the nucleus (38). Phosphoinositides (PtdInsPs) are lipids distributed in the cell membrane, cytoplasm, and nucleus that regulate various cell activities (61–64). In contrast to Cti6 in yeast, however, Clp1 did not bind to PI(3,5)P2 but bound to PtdIns(3)P and PtdIns(5)P. Recent studies have shown that PHD fingers may function as nuclear receptors of PtdInsPs. For example, the nuclear protein ING2, which contains a PHD, interacts with PtdIns(5)P *in vivo* to regulate the ability of ING2 to activate p53 and p53-dependent apoptotic pathways (65). In M. oryzae, the PHD was indispensable for the function of Clp1, which is consistent with previous reports in other filamentous fungi (31, 34, 35). The *clp1*^Δ^*^PHD^* strain, in which the PHD of Clp1 was deleted, had phenotypes similar to those of the Δ*clp1* mutant. We infer that Clp1 is a PtdIns(3)P and PtdIns(5)P receptor and plays a role in transcriptional regulation. Clp1 is distributed not only in the nucleus but also in vesicles around the nucleus, indicating that Clp1 also shuttles between cytoplasmic vesicles and the nucleus. Therefore, the biological function and working mechanism of Clp1 are at least partly different from those of Cti6 in yeast.

We demonstrated that Clp1 is involved in growth, hyphal branching, conidiation, spore and appressorium size, appressorium turgor pressure, plant penetration, virulence, and autophagy in M. oryzae. In S. cerevisiae, Δ*cti6* exhibits growth defects under iron-limited conditions (31). In *T. reesei*, the growth of Δ*clp1* is comparable to that of the wild type, but Δ*clp1* has a severe defect in conidiation (34). In A. flavus, deletion of *CTI6* results in attenuated growth and reduced conidiation (35). In M. oryzae, the Δ*clp1* mutant also displayed decreased growth but increased conidiation. An increase in melanin synthesis promotes conidiophore differentiation and spore production in M. oryzae strain 70-15 (41, 42). Δ*clp1* had upregulated expression levels of two melanin synthesis genes (*RSY1* and *ALB1*), higher melanin content in the mycelium, and more hyphal branching, which likely led to increased spore production per unit colony area in the mutant. In M. oryzae, Δ*clp1* showed a hyperbranching phenomenon, which also appeared in mutants with defects in polarized cell growth, such as Δ*tea1* (51), Δ*arf6* (52), Δ*spa2* (53), and Δ*gel2*Δ*gel3*Δ*gel4* (54). However, *TEA1*, *ARF6*, *SPA2*, *GEL2*, *GEL3*, and *GEL4* were not downregulated at the transcriptional level in Δ*clp1*, suggesting that a hyperbranching phenomenon is associated with defects in polarized cell growth but has diverse molecular mechanisms in M. oryzae. M. oryzae infects plants through an appressorium (66). Δ*clp1* formed a smaller appressorium that had reduced turgor and hindered glycogen metabolism. Therefore, the decreased virulence of Δ*clp1* is caused by delayed plant penetration and slowed invasive growth, and delayed appressorial penetration is due to defects in appressorial function.

In M. oryzae, the formation of functional appressorium and fungal virulence requires the sophisticated regulation of autophagy (15, 56, 67). M. oryzae degraded and reutilized conidial storage and cellular components via autophagy and other catabolic pathways during appressorium formation (17). The deletion of *CLP1* resulted in an increased background autophagy level, and Clp1 interacted with Atg5, Atg7, Atg16, Atg24, and Atg28 at PAS and autophagosomes in M. oryzae, suggesting that Clp1 regulated autophagy by directly changing the activities of autophagy proteins. In S. cerevisiae, Atg5, Atg7, and Atg16 are involved in the autophagosome elongation stage (68). Atg7 acts as an E1 enzyme in the ubiquitination reaction to activate the carboxyl terminus of Atg8 (69), and the Atg5-Atg12-Atg16 complex assists in the formation of Atg8-PE (70). Atg24 is localized to inclusion bodies and preautophagosomal structure (PAS) sites and plays a role in the nucleation stage of autophagosome formation (71, 72). Atg28 is a selective autophagy gene involved in pexophagy in Pichia pastoris (73). In M. oryzae, Atg5, Atg7, and Atg16 are required for pathogenicity, and their gene-deletion mutants lose virulence to rice, while Atg24 and Atg28 are dispensable for virulence. The regulation of transcription of autophagy-related genes or modification of autophagy-related proteins will change the autophagy level and virulence of M. oryzae. For example, acetylation of Atg7 catalyzed by Gcn5 and acetylation of Atg3 and Atg9 by Hat1 are required for autophagy and fungal virulence (29, 74). Deletion of *SNT2*, which encodes a protein with three PHDs, results in accelerated levels of background autophagy and reduced virulence. PHD1 (the first PHD of Snt2) can bind histone H3 and acts as an epigenetic regulator through histone H3 deacetylation, which determines autophagy-related growth and plant infection in M. oryzae (67). In human tumor cell lines, PHF23 (PHD finger protein 23) with a PHD-like zinc finger domain interacted with the E3 ubiquitin ligase LRSAM1 (leucine-rich repeat and sterile α motif-containing 1), which negatively regulates ubiquitin-dependent autophagy. Knockdown of *PHF23* promoted autophagosome synthesis (60). Both enhanced and attenuated autophagy resulted in impaired virulence of M. oryzae (16, 20, 75–77). Therefore, we speculate that Clp1 affects the development and pathogenicity of M. oryzae in two ways: one is to regulate autophagy homeostasis by interacting with autophagy-related proteins at PAS and autophagosomes, and the other is to regulate gene transcription in the nucleus.

Taken together, this study provided insights into the roles of a PHD protein, Clp1, in hyphal growth, conidiation, appressorium formation, appressorial glycogen metabolism and turgor production, and plant infection. Notably, we identified a connection between Clp1 and autophagy. Clp1 participates in regulating the homeostasis of autophagy by interacting with Atg5, Atg7, and Atg16 at PAS and autophagosomes during the early stages of spore germination and appressorium formation, thereby affecting autophagy-related pathogenesis.

## MATERIALS AND METHODS

### Fungal strains, culture conditions, and primers.

Magnaporthe oryzae wild-type strain 70-15 and its derivatives were cultured on complete medium (CM) at 25°C under a 16-h light/8-h dark cycle (78). For stress experiments, the fungus was inoculated on CM plates supplemented with 1 mM paraquat, 8 mM hydrogen peroxide, 0.0055% sodium dodecyl sulfate (SDS), 75 μg mL^−1^ calcofluor white (CFW) (Sigma-Aldrich), 0.6 M NaCl, 0.6 M KCl, or 1 M sorbitol and cultured at 25°C for 9 days. The experiments were repeated 3 times with 5 biological replicates each. The PCR primers used in this study are listed in Table S3.

### Generation and complementation of the null mutant.

The *CLP1* knockout cassette was built in pKO3A vectors using a method reported previously (37). The upstream and downstream fragments (approximately 1,300 bp) of *CLP1* amplified from the genomic DNA of the wild-type strain 70-15 and a hygromycin-resistance gene (*HPH*) were fused into the HindIII-SalI site of the pKO3A vector using a fusion enzyme (Vazyme Biotech, China). The knockout cassette was transformed into the wild-type strain 70-15 through Agrobacterium tumefaciens-mediated transformation (ATMT) (37). The transformants were screened on selective medium containing 0.5 μM 5-fluoro-2′-deoxyuridine (F2dU) and 200 μg mL^−1^ hygromycin B. The null mutants were identified by confirmation of the deleted gene using double PCR, confirmation of recombination of *HPH* in the targeted locus using PCR, and quantification of the copy number of *HPH* in the genome using quantitative real-time PCR (qPCR), as previously reported (36, 46).

The Δ*clp1* mutant was complemented with the native copy of *CLP1* cloned from the wild type. *CLP1* was cloned into the site of EcoRI-Xbal of the pKD3 vector, which contained an herbicide bialaphos-resistance gene (*BAR*) (41), and was then transformed into Δ*clp1* via ATMT. The complementation strain (*clp1c*) was screened on selection plates supplemented with 600 μg/mL glufosinate ammonium and confirmed at the mRNA level by reverse transcriptase PCR (RT-PCR). To obtain the *clp1*^Δ^*^PHD^* strain, the Δ*clp1* mutant was complemented with a mutated *CLP1* gene in which a coding sequence for the PHD was deleted.

### Quantitative real-time PCR and RNA-seq.

A total of 200 μL of spore suspension (5 × 10^4^ conidia mL^−1^) was spread evenly onto cellophane films that covered CM plates and was cultured at 25°C for 3 days. Total RNA of the aerial mycelia was isolated with TRIzol following the manufacturer’s instructions (TaKaRa, Japan). mRNA was reverse transcribed into cDNA using a PrimeScript RT reagent kit with genomic DNA (gDNA) Eraser (TaKaRa, Japan). Quantitative real-time PCR (qPCR) was used to quantify gene expression levels on a real-time PCR detection system Mastercycler (Eppendorf, Germany) with a SYBR premix *Ex Taq* (Tli RNaseH Plus) kit (TaKaRa, Japan). qPCR was performed with four biological replicates, using *40S* and *α-ACTIN* as reference genes.

RNA sequencing (RNA-seq) was performed according to previous reports (36, 79). Total RNA of the aerial mycelia was isolated using an RNeasy Plus minikit (Qiagen, Germany). The experiments were repeated with biological triplicates. The cDNA libraries were sequenced using a next-generation sequencing (NGS) technology with a 2 × 150-nucleotide (nt) method based on the Illumina HiSeq platform (NovaSeq 6000) at Personalbio Technology Co. (Shanghai, China). The amount of data obtained by sequencing was 6 billion bases (G)/sample. Clean reads were mapped to the M. oryzae (70-15) genome database (MG8) using Tophat2 software (HISAT2). Data were analyzed using HTSeq software and then normalized to fragments per kilobase/million (FPKM). Significant differences in FPKM between different samples were analyzed using DESeq software.

### Phenotypic analysis.

The strains were cultured on CM for 8 days, and then the colony diameter and spore production were measured. For the conidial germination rate at 4 h postinoculation (hpi) and the appressorium formation rate at 24 hpi, 25 μL of spore suspension (1 × 10^5^ conidia mL^−1^) was dropped onto hydrophobic coverslips and incubated at 22°C in the dark for 4 h and 24 h (46). The spore diameters and areas of mature appressoria were measured using NIS-Elements D 3.2 software. Appressorium turgor was evaluated using incipient cytorrhysis (cell collapse) assays as described previously (15). The 24-hpi appressoria were treated with a series of glycerol solutions with different concentrations (0.5 M to 3 M) for 5 min, and the rate of collapsed appressoria was determined. The experiments were performed three times with three replicates each time.

### Cell wall, vacuole, glycogen, and lipid droplet staining.

A total of 10 μL of spore suspension (5 × 10^4^ conidia mL^−1^) was inoculated onto a thin layer of CM overlying the glass slide in a petri dish and was then covered with a coverslip and cultured at 25°C for 3 days. The hyphal cell wall was stained with 10 μg/mL CFW for 5 min. The number of branches in a 0.5-mm length of hyphae was counted, and the length of hyphal cells was measured via NIS-Elements D 3.2 software (52). To label vacuoles, germinated spores were stained with 10 μm CMAC (7-amino-4-chloromethylcoumarin) and incubated for 30 min (52).

For glycogen observation, spores and appressoria were stained with KI/I_2_ solution (60 mg mL^−1^ KI and 10 mg mL^−1^ I_2_) for 2 min (5). For lipid droplet staining, appressoria were induced on hydrophobic borosilicate glass coverslips (Thermo Scientific, USA) with spore suspensions supplemented with 8 μg mL^−1^ tricyclazole, an inhibitor of melanin synthesis. The lipid droplets were stained with a dye, BODIPY (boron pyrromethene; Thermo Fisher, USA), for 3 min and then observed under a fluorescence microscope (80).

### Plant infections.

For the virulence test on rice, 2.5 mL of spore suspension (5 × 10^4^ spores mL^−1^) in 0.2% gelatin (Sango Biotech) was evenly sprayed onto 12- to 14-day-old rice seedlings (Oryza sativa cv. CO39). The rice seedlings were first cultured in the dark at 22°C for 2 day and were then recultured at 25°C (16 h light/8 h dark) for 3 to 4 d. When typical pyriform-like lesions appeared on rice leaves, the disease severity in 5-cm-long infected leaves was calculated (46, 81). For the virulence test on barley, 5-cm-diameter mycelial blocks were inoculated on isolated barley leaves and cultured at 25°C for 4 days.

To examine appressorial penetration on leaves, 20 μL of spore suspension (1 × 10^5^ spores mL^−1^) was dropped onto barley leaves and cultured at 25°C. Barley leaves were collected at 24 hpi, 48 hpi, and 72 hpi, immersed in methanol for decolorization, and fixed in an alcoholic lactophenol solution. The leaves were observed under a microscope, and the proportion of appressorium penetration was calculated (39).

### Observation of fluorescent fusion proteins.

The coding sequence (CDS) fragment of *CLP1* was fused with the N terminus of GFP at the BamHI/SmaI site of pKD3-GFP, which contained an herbicide bialaphos-resistance gene (*BAR*) (82), and the fusion gene was transformed into Δ*clp1* via ATMT. The coding sequence of *H_2_B* was fused into the N terminus of mCherry in pKD8-mCherry, the CDS fragments of *ATG5*, *ATG7*, and *ATG16* were inserted into the BamHI/SmaI site of the pKD8-DsRed vector (83), and the CDS fragments of *ATG24* and *ATG28* were fused into the BamHI/SmaI site of pKD8-mCherry. The ATG8-DsRed fusion expression vector was previously constructed (84). These fusion genes were transformed into Δ*clp1* expressing Clp1-GFP. pKD8-mCherry is a binary vector that is built by replacing GFP with mCherry in pKD8-DsRed. The appressoria were induced on hydrophobic borosilicate glass coverslips (Thermo Scientific, USA). The colocalization of Clp1 and H_2_B, Atg5, Atg7, Atg8, Atg16, Atg24, or Atg28 in hyphae, spores, and appressoria was observed under a laser scanning confocal microscope (FV3000, Olympus).

### Autophagy detection.

The GFP-Atg8 vector (75) was transformed into wild-type 70-15 and Δ*clp1* strains via ATMT. To induce autophagy, the strains expressing GFP-Atg8 were cultured in liquid CM medium at 25°C for 36 h, transferred to a nitrogen starvation medium, SD-N (synthetic defined medium without amino acids and ammonium sulfate), and cultured for 4 h and 8 h. Total proteins were extracted from the mycelium, and GFP and GFP-Atg8 were detected using Western blotting. The primary antibody to detect GFP-Atg8 and GFP was an anti-GFP antibody (ab32146, Abcam), and the amounts of GFP-Atg8 and free GFP were measured using densitometric analysis in ImageJ software.

### Yeast two-hybrid assay.

The CDS fragments in cDNAs of *CYC8*, *RPD3*, *SIN3*, *ATG5*, *ATG7*, *ATG16*, *ATG24*, and *ATG28* were cloned into the prey vector pGADT7, and the CDS fragment of *CLP1* was cloned into the bait vector pGBKT7. A pair of vectors (prey and bait vectors) were cotransformed into the yeast strain Y_2_H Gold according to the instructions of the BD Matchmaker library construction and screening kits (Clontech). The transformed yeast cells were cultured on a synthetic defined double-deficient medium (SD-Leu-Trp) and four-deficient medium (SD-Leu-Trp-His-Ade) at 30°C for 4 days. The yeast cells were washed from the SD-Leu-Trp medium with double-distilled water (ddH_2_O) and diluted to a series of concentrations, spotted onto SD-Leu-Trp and SD-Leu-Trp-His-Ade plates, and cultured for 3 to 5 days. A pair of vectors, pGADT7-T and pGBKT7-53, were used as positive controls, and pGADT7-T and pGBKT7-Lam were used as negative controls.

### Pulldown assays.

The amplified *CLP1* cDNA fragment was fused into the EcoRI site of a GST-tagged pGEX4T vector, and the cDNA fragments of *ATG5*, *ATG7*, *ATG16*, *ATG24*, and *ATG28* were fused into the SalI-HindIII site of a 3× FLAG-tagged pET21a vector. The plasmids were transferred into Escherichia coli BL21. The proteins were expressed in bacteria by inducing with 2 μL of 1 M IPTG (isopropyl-β-d-thiogalactopyranoside; final concentration at 4 mM) and culturing at 18°C for 16 h. The GST-Clp1 or GST protein lysis supernatants were incubated with GST beads (BBI, China) at 4°C for 2 h. After washing, 3× FLAG-Atg5, Atg7, Atg16, Atg24, and Atg28 protein lysis supernatants were coincubated with beads at 4°C for an additional 2 h. Finally, the proteins were eluted from beads and detected via Western blotting. The primary antibodies were an anti-GST antibody EM80701 (HUABIO, China) and an anti-FLAG antibody M1403-2 (HUABIO, China).

### Coimmunoprecipitation (co-IP) assays.

The CDS fragments of *ATG5*, *ATG7*, and *ATG16* were inserted into the BamHI-SmaI site of pKD7 vector with 3× FLAG-tag (51). These fusion genes were transformed into Δ*clp1* expressing Clp1-GFP. Total protein was extracted and incubated with anti-GFP affinity beads 4FF SA070005 (Smart-Lifesciences, China) for 4 h at 4°C. After the beads were washed with low- and high-salt buffers, proteins were eluted with 0.2 M glycerol-HCl buffer (pH 3.3). Eluted and total proteins were detected by Western blotting with anti-GFP antibody and anti-FLAG antibody (HUABIO, China).

### Protein lipid overlay assay.

The GST-Clp1 and GST proteins were purified with GST beads and were diluted to a final concentration of 1 μg mL^−1^ with Tris-buffered saline with Tween 20 (TBST) buffer containing 5% skim milk powder. PIP Strips (P-6001, Echelon Biosciences) were sealed in 5% skim milk powder solution at room temperature for 1 h, placed in diluted GST-Clp1 or GST protein solution, and incubated overnight at 4°C. PIP Strips were washed three times with TBST buffer, and GST-Clp1 and GST proteins were detected using an anti-GST antibody, EM80701 (HUABIO, China) following a previous report (20).

### Statistical analysis.

All values are shown as the mean ± the standard deviation (SD). The *P* value was calculated using unpaired two-tailed Student’s *t* test and GraphPad Prism 8. *P* values of <0.05 were considered significant, while *P* values of >0.05 were considered nonsignificant.

### Data availability.

All data supporting the findings of the current study are available within the figures and supporting information. All strains generated during this study are available from the corresponding author upon reasonable request. The RNA-seq data were deposited in OMIX, China National Center for Bioinformation/Beijing Institute of Genomics, Chinese Academy of Sciences (https://ngdc.cncb.ac.cn/omix; accession no. CRA007268).
