# Supplementary material for: The Plant Homeodomain Protein Clp1 Regulates Fungal Development, Virulence, and Autophagy Homeostasis in Magnaporthe oryzae
Source: Microbiol Spectr. 2022 Aug 29;10(5):e01021-22. doi: 10.1128/spectrum.01021-22 (PMC9602895; doi:10.1128/spectrum.01021-22)
Supplement: Supplemental file 1 — Fig. S1-S6; Tables S1-S3. Download spectrum.01021-22-s0001.pdf, PDF file, 3.0 MB [file spectrum.01021-22-s0001.pdf]

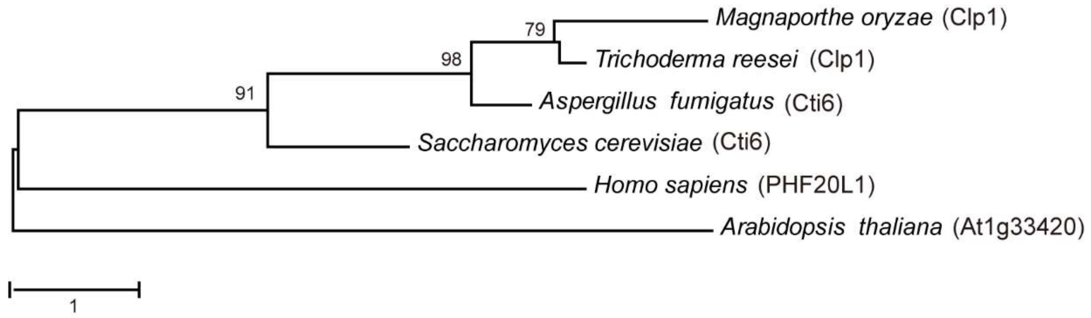

Figure S1. Alignment tree of Clp1 in *M. oryzae* with its homologous proteins in other fungal species was constructed using the neighbor-joining method in MEGA 5.0. The scale bar indicated that the genetic distance is 1.

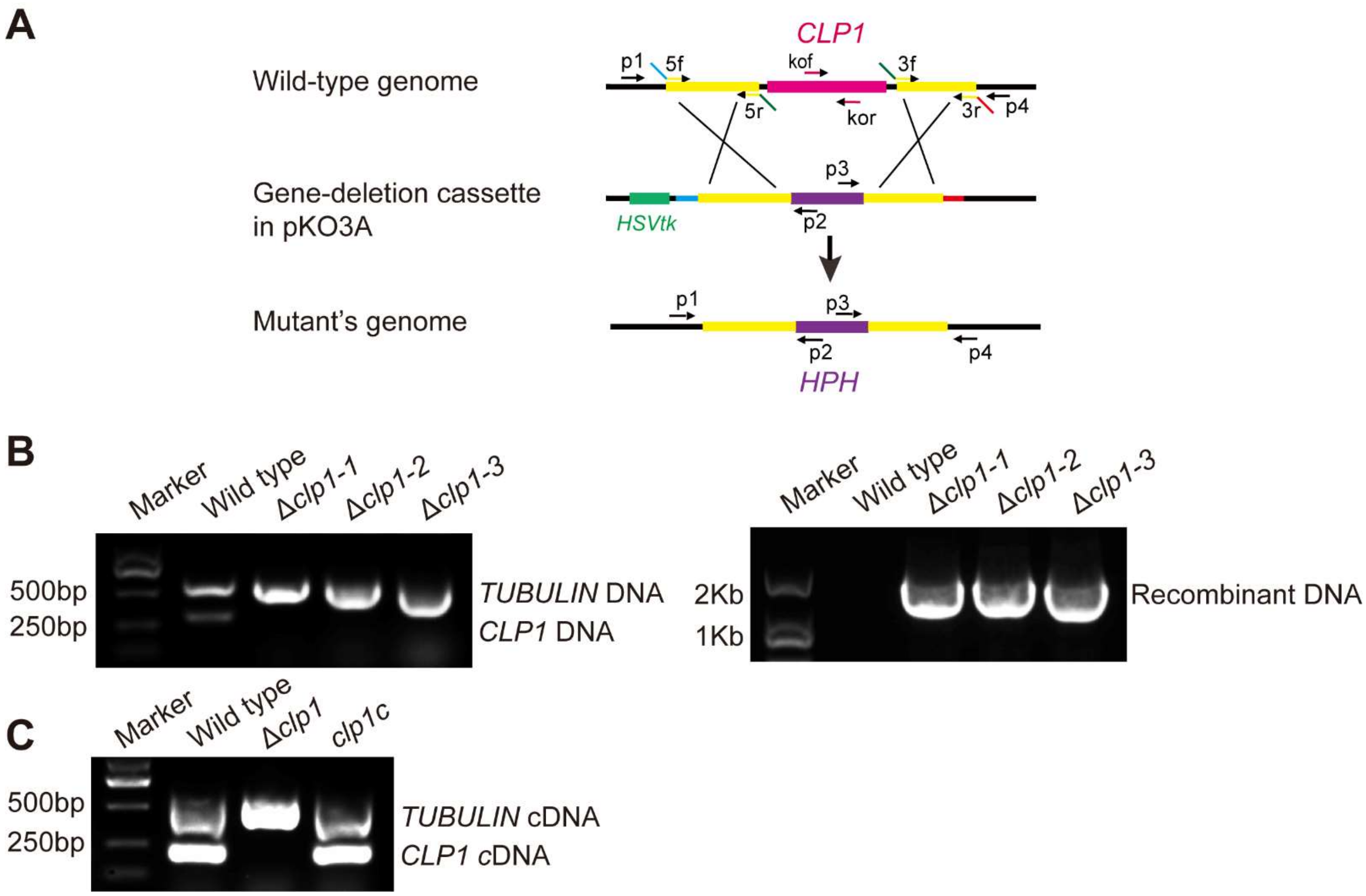

Figure S2. Knockout and complementation of *CLP1* in *M. oryzae*. A. Targeted gene deletion strategy. Two pairs of primer sets (5f/5r and 3f/3r) were used to clone the up- and downstream flanking fragments of the targeted gene. The primer set kof/kor was used to clone a fragment of the *CLP1* gene in transformants. The primer sets p1/p2 and p3/p4 were used to clone the recombinational DNA fragments in mutants. pKO3A is a vector containing HSVtk (a suicide gene). **B**. *CLP1* deletion events in three mutants were confirmed at the DNA level by PCR for both *CLP1* (amplified by primers kof/kor) and the positive control of *β-TUBULIN* DNA (left panel) and by PCR for the recombinational DNA event using primer sets p1/p2 or p3/p4 (right panel). C. Complementation of Δ*clp1* by a native *CLP1* (*clp1c*). The complement of *CLP1* in Δ*clp1* was confirmed at the RNA level by RT–PCR using *β-TUBULIN* as a control.

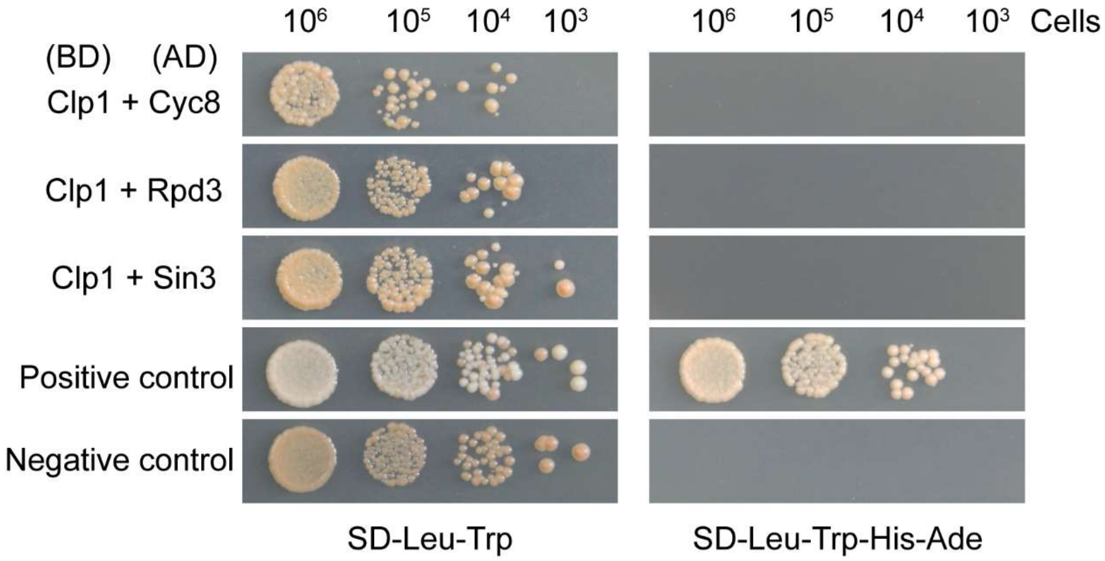

Figure S3. A yeast two-hybrid assay showed that Clp1 did not interact directly with Cyc8, Rpd3 or Sin3 in *M. oryzae*.

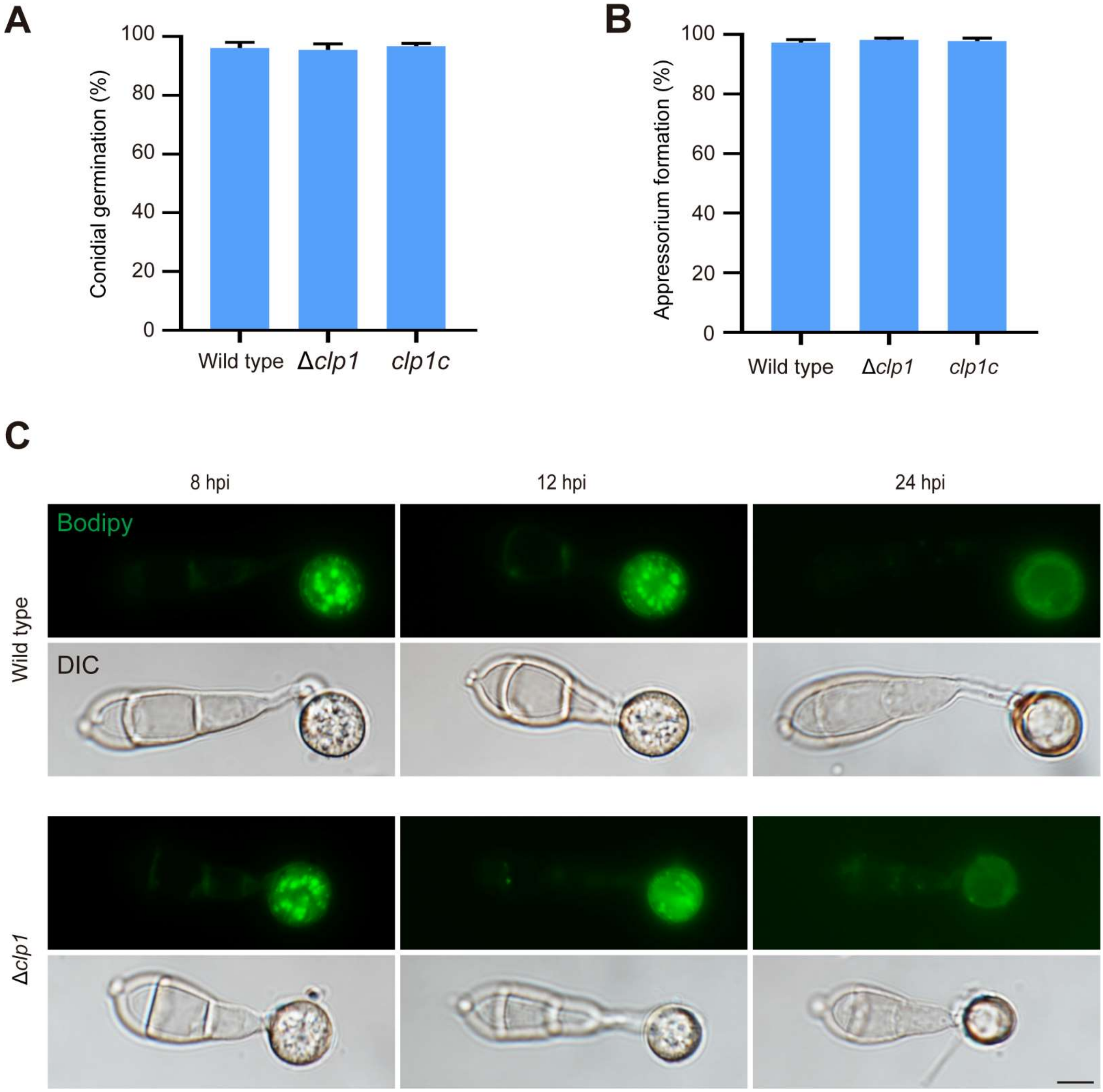

Figure S4. Conidial germination and appressorium formation of Δ*clp1*. A. Conidial germination rates of the wild-type, Δ*clp1* and *clp1c* strains on plastic coverslips at 4 hpi. **B**. Appressorium formation rates of the three *M*. *oryzae* strains on plastic coverslips at 24 hpi. **C**. Observation of lipid droplets in conidial and appressorial cells at 8 hpi, 12 hpi, and 24 hpi. Lipid droplets were stained with the dye BODIPY (Boron pyrromethene). Bar, 5 μm.

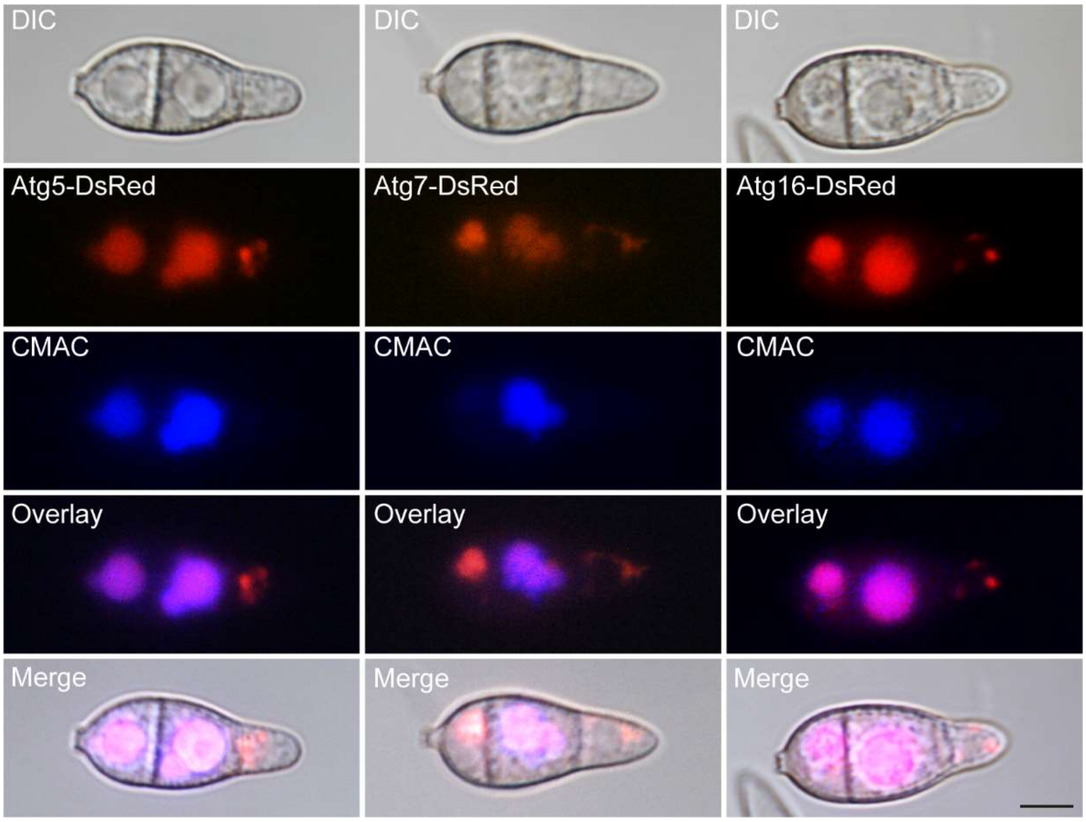

Figure S5. Colocalization assays between CMAC and three autophagy proteins. CMAC-labeled vacuoles were observed in spores expressing Atg5-DsRed, Atg7-DsRed, or Atg16-DsRed. Bar, 5 μm.

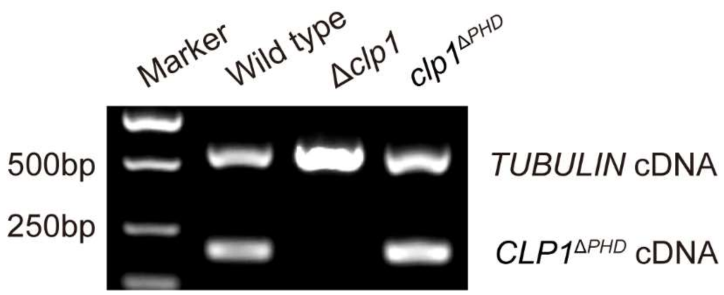

Figure S6. **The *clp1*$^{\Delta PHD}$ mutant, in which a mutated Clp1 protein without a PHD domain was expressed, was confirmed by PCR**.

Table S1. Copy identification of *HPH* inserted in the mutant genome by qPCR.

Mutant No	Copy numbers of *HPH*[a]	Insertion event
Δ*clp1-1*	1.13	Single[b]
Δ*clp1-2*	1.04	Single
Δ*clp1-3*	0.93	Single

[a] Copy number of a resistant gene *HPH* in the mutant genome was quantified by qPCR after normalization with the *β-TUBULIN* gene.

[b] "Single" represents the targeted gene deletion event without ectopic insertion.

Table S2. Differentially expressed genes in Δ*clp1* when compared with the wild-type strain (*P* < 0.05).

Gene ID	FPKM_WT	FPKM_Δ*clp1*	FoldChange(Δ*clp1* /WT)	log2(FoldChange)	pval	padj	Description
MGG_00015	6.920316046	322.262615	46.56761524	5.541255098	1.63652E-07	7.309E-06	catechol O-methyltransferase [Pyricularia oryzae 70-15]
MGG_00016	414.9347634	2584.359273	6.228350817	2.638850208	5.2054E-10	4.27265E-08	2-epi-5-epi-valiolone synthase [Pyricularia oryzae 70-15]
MGG_00017	358.8259772	893.9480117	2.491313529	1.316906595	0.001267296	0.01424155	L-aminoadipate-semialdehyde dehydrogenase
MGG_00019	2202.089198	65741.65829	29.85422132	4.899863035	0.001290437	0.014434832	uncharacterized protein MGG_00019 [Pyricularia oryzae 70-15]
MGG_00020	336.3361457	7627.340105	22.67772942	4.503204294	7.64328E-23	4.88687E-20	uncharacterized protein MGG_00020 [Pyricularia oryzae 70-15]
MGG_00021	11.04556841	61.02914736	5.525215643	2.466030772	1.87543E-05	0.000461189	uncharacterized protein MGG_00021 [Pyricularia oryzae 70-15]
MGG_00022	2.31722272	21.68175967	9.356787106	3.226013228	0.000272197	0.004266637	bassianolide synthetase [Pyricularia oryzae 70-15]
MGG_00041	2590.844991	1146.227394	0.442414501	-1.176529422	0.003176916	0.028314878	O-methyltransferase [Pyricularia oryzae 70-15]
MGG_00042	83.70251077	20.83153691	0.248875891	-2.006501617	0.000115817	0.002128503	uncharacterized protein MGG_00042 [Pyricularia oryzae 70-15]
MGG_00059	22.64642581	7.015848072	0.309799353	-1.690593962	0.020242078	0.105085797	isotrichodermin C-15 hydroxylase [Pyricularia oryzae 70-15]
MGG_00082	2190.079552	341.4041904	0.155886662	-2.681430604	3.2645E-10	2.81257E-08	uncharacterized protein MGG_00082 [Pyricularia oryzae 70-15]
MGG_00083	29.40104096	2.364886614	0.080435472	-3.636024316	5.18854E-06	0.000155793	uncharacterized protein MGG_00083 [Pyricularia oryzae 70-15]
MGG_00085	3272.50198	1121.617347	0.342740006	-1.544813497	0.004000563	0.033585188	lactose permease [Pyricularia oryzae 70-15]
MGG_00088	29.51130456	10.81240442	0.366381784	-1.448580317	0.026184448	0.125744471	glycosyl hydrolase [Pyricularia oryzae 70-15]
MGG_00097	186.3971254	750.5244706	4.026480929	2.0095195	0.004737997	0.038345895	uncharacterized protein MGG_00097 [Pyricularia oryzae 70-15]
MGG_00099	199.614343	3756.452566	18.81855036	4.234083593	1.6353E-20	8.6372E-18	uncharacterized protein MGG_00099 [Pyricularia oryzae 70-15]
MGG_00101	910.1292207	2131.044629	2.341474793	1.227417507	0.002511329	0.02388884	ATPase NPA3 [Pyricularia oryzae 70-15]
MGG_00104	1271.740913	2915.675456	2.292664666	1.197025356	0.003088905	0.027878168	uncharacterized protein MGG_00104 [Pyricularia oryzae 70-15]
MGG_00111	2116.840479	910.4828532	0.43011406	-1.217208802	0.00311912	0.028025938	phospholipid-transporting ATPase 1 [Pyricularia oryzae 70-15]
MGG_00135	2377.995059	6466.087969	2.719134316	1.443147417	0.000393023	0.005623614	T-complex protein 1 [Pyricularia oryzae 70-15]
MGG_00136	982.7051765	1973.10754	2.007832651	1.005639028	0.013328223	0.079290528	uncharacterized protein MGG_00136 [Pyricularia oryzae 70-15]
MGG_00152	1145.887492	560.3897517	0.489044305	-1.031962924	0.013105338	0.078596933	uncharacterized protein MGG_00152 [Pyricularia oryzae 70-15]
MGG_00156	112.8697442	44.71307665	0.396147586	-1.335890085	0.00426501	0.035159899	uncharacterized protein MGG_00156 [Pyricularia oryzae 70-15]
MGG_00176	1277.252192	341.2334685	0.267162171	-1.904212352	4.27571E-06	0.000131497	uncharacterized protein MGG_00176 [Pyricularia oryzae 70-15]
MGG_00180	386.7445166	823.1020596	2.128283723	1.08969049	0.036854361	0.157754328	ubiquitin-conjugating enzyme [Pyricularia oryzae 70-15]
MGG_00190	102.9982469	14.680804	0.142534504	-2.810616897	1.15645E-07	5.38259E-06	uncharacterized protein MGG_00190 [Pyricularia oryzae 70-15]
MGG_00194	612.9819733	1439.942428	2.349077934	1.232094578	0.002750603	0.025663841	uncharacterized protein MGG_00194 [Pyricularia oryzae 70-15]
MGG_00198	1561.598886	3515.444796	2.251182955	1.170683311	0.003732932	0.031867644	flavohemoglobin [Pyricularia oryzae 70-15]
MGG_00203	2462.363519	489.5534528	0.198814452	-2.330505466	0.046526916	0.18424459	uncharacterized protein MGG_00203 [Pyricularia oryzae 70-15]
MGG_00212	2878.395859	6439.036402	2.237022535	1.16157979	0.003240521	0.028588124	superoxide dismutase [Pyricularia oryzae 70-15]
MGG_00220	2894.060332	7326.422807	2.53153769	1.340013963	0.003644338	0.031221031	NADP-dependent alcohol dehydrogenase 6
MGG_00223	7283.567399	15667.78914	2.151114733	1.105084475	0.005704423	0.043976248	fructose-bisphosphate aldolase 1 [Pyricularia oryzae 70-15]
MGG_00225	17521.80173	7918.56	0.451926127	-1.145841128	0.022023606	0.111522622	uncharacterized protein MGG_00225 [Pyricularia oryzae 70-15]
MGG_00226	4122.173943	1346.279701	0.326594588	-1.61442721	6.7362E-05	0.001337113	uncharacterized protein MGG_00226 [Pyricularia oryzae 70-15]
MGG_00233	261.6867905	10.95112673	0.041848221	-4.578689884	3.33846E-18	1.26736E-15	fatty acid synthase S-acetyltransferase [Pyricularia oryzae 70-15]
MGG_00244	4697.436897	576.854117	0.122801887	-3.02559536	3.94585E-10	3.32876E-08	15-hydroxyprostaglandin dehydrogenase [Pyricularia oryzae 70-15]
MGG_00245	36.75191207	122.4987678	3.333126386	1.736876023	0.000340412	0.005049232	uncharacterized protein MGG_00245 [Pyricularia oryzae 70-15]
MGG_00259	65.5762643	25.08523268	0.38253525	-1.386335398	0.007629529	0.054074397	uncharacterized protein MGG_00259 [Pyricularia oryzae 70-15]
MGG_00266	0	13.70505105	Inf	Inf	2.24282E-05	0.000533186	uncharacterized protein MGG_00266 [Pyricularia oryzae 70-15]
MGG_00269	8.897985557	74.32751891	8.35329732	3.062345789	5.7405E-07	2.27152E-05	uncharacterized protein MGG_00269 [Pyricularia oryzae 70-15]
MGG_00270	204.7929859	952.9106068	4.653043181	2.218174576	1.39623E-07	6.32887E-06	zinc-containing alcohol dehydrogenase [Pyricularia oryzae 70-15]
MGG_00272	522.0727965	1742.259065	3.337195648	1.738636269	0.000112031	0.002084149	uncharacterized protein MGG_00272 [Pyricularia oryzae 70-15]
MGG_00282	833.1530075	384.1394444	0.461067104	-1.116951358	0.0059364	0.045384131	subtilisin [Pyricularia oryzae 70-15]
MGG_00287	911.5611205	162.6799591	0.178463029	-2.486302859	6.69628E-09	4.28139E-07	uncharacterized protein MGG_00287 [Pyricularia oryzae 70-15]
MGG_00290	237.7943944	71.42408861	0.300360691	-1.735232079	0.000167926	0.002853097	uncharacterized protein MGG_00290 [Pyricularia oryzae 70-15]
MGG_00294	958.741384	415.5911027	0.433475711	-1.205976939	0.003448839	0.02990471	uncharacterized protein MGG_00294 [Pyricularia oryzae 70-15]

MGG_00295	320.3089747	117.1217913	0.365652543	-1.451454697	0.00073492	0.009203925	uncharacterized protein MGG_00295 [Pyricularia oryzae 70-15]
MGG_00299	837.6216764	257.7815068	0.307754102	-1.700150009	4.07633E-05	0.000885855	uncharacterized protein MGG_00299 [Pyricularia oryzae 70-15]
MGG_00304	14.83490103	84.40512898	5.689632093	2.508335367	4.04465E-06	0.000125663	peptide transporter MTD1 [Pyricularia oryzae 70-15]
MGG_00314	1669.79211	633.5926507	0.379444032	-1.398040992	0.00292999	0.026842776	lipase 4 [Pyricularia oryzae 70-15]
MGG_00321	5.795605258	153.1700632	26.42865695	4.724031207	1.90081E-15	4.44059E-13	uncharacterized protein MGG_00321 [Pyricularia oryzae 70-15]
MGG_00325	45.98337389	7.335046971	0.159515198	-2.648234214	7.99819E-05	0.001548551	uncharacterized protein MGG_00325 [Pyricularia oryzae 70-15]
MGG_00336	1790.048882	3912.013135	2.185422517	1.127912229	0.004960745	0.039520536	COP9 signalosome complex subunit 4 [Pyricularia oryzae 70-15]
MGG_00338	778.8796574	307.3186489	0.394564996	-1.341665123	0.000990436	0.011670046	uncharacterized protein MGG_00338 [Pyricularia oryzae 70-15]
MGG_00343	935.706197	3227.580903	3.44935292	1.786325745	1.34688E-05	0.000348869	uncharacterized protein MGG_00343 [Pyricularia oryzae 70-15]
MGG_00346	32.69684081	92.921667	2.841915754	1.506863788	0.002179172	0.021504936	pi-transporter A-1 [Pyricularia oryzae 70-15]
MGG_00350	306.916117	748.4021939	2.438458434	1.28596938	0.001927662	0.019744717	uncharacterized protein MGG_00350 [Pyricularia oryzae 70-15]
MGG_00363	492.7295413	170.0589322	0.345136465	-1.534761186	0.000260923	0.004121835	uncharacterized protein MGG_00363 [Pyricularia oryzae 70-15]
MGG_00383	3626.496821	7873.065681	2.170983753	1.11834893	0.005385214	0.042052933	S-adenosylmethionine synthase [Pyricularia oryzae 70-15]
MGG_00387	244.9263288	83.35549159	0.340328833	-1.554998713	0.000424611	0.005956541	uncharacterized protein MGG_00387 [Pyricularia oryzae 70-15]
MGG_00394	2154.702293	455.7756613	0.211526048	-2.24109276	5.89991E-08	2.97395E-06	uncharacterized protein MGG_00394 [Pyricularia oryzae 70-15]
MGG_00395	9848.703978	1458.788201	0.148119814	-2.755163452	1.46253E-09	1.07029E-07	uncharacterized protein MGG_00395 [Pyricularia oryzae 70-15]
MGG_00409	1357.331301	3623.258178	2.669398529	1.41651471	0.000512208	0.006921365	uncharacterized protein MGG_00409 [Pyricularia oryzae 70-15]
MGG_00416	6.553044186	0.34480135	0.052616973	-4.248327932	0.014809233	0.085020113	MFS transporter [Pyricularia oryzae 70-15]
MGG_00418	18.65708124	2.859597973	0.153271454	-2.705839066	0.007612845	0.054019894	uncharacterized protein MGG_00418 [Pyricularia oryzae 70-15]
MGG_00419	65.41062693	17.28111361	0.264194282	-1.920328853	0.010727978	0.06838875	major facilitator superfamily transporter [Pyricularia oryzae 70-15]
MGG_00422	602.8227455	256.8284657	0.426043091	-1.23092874	0.002753236	0.025668692	retinol dehydrogenase 12 [Pyricularia oryzae 70-15]
MGG_00423	3687.813804	1244.967467	0.337589567	-1.566657774	0.000122423	0.002209807	laccase TilA [Pyricularia oryzae 70-15]
MGG_00426	3.217161839	20.78900083	6.461907069	2.691960003	0.001236349	0.013958332	short-chain dehydrogenase/reductase SDR
MGG_00429	800.1234799	279.3242243	0.349101397	-1.518281966	0.00025862	0.004096105	uncharacterized protein MGG_00429 [Pyricularia oryzae 70-15]
MGG_00435	370.9061959	970.9999321	2.617912407	1.388416827	0.000784981	0.009681165	uncharacterized protein MGG_00435 [Pyricularia oryzae 70-15]
MGG_00447	1952.853788	947.9380987	0.485411711	-1.04271918	0.010049546	0.06563542	brefeldin A resistance protein [Pyricularia oryzae 70-15]
MGG_00458	699.7979174	1599.686587	2.285926475	1.192779001	0.042519473	0.173873902	caltractin [Pyricularia oryzae 70-15]
MGG_00464	6.116781201	21.99769471	3.596286019	1.846507766	0.014187332	0.082621146	MFS phospholipid transporter Git1 [Pyricularia oryzae 70-15]
MGG_00485	374.1912926	128.0965897	0.342329157	-1.54654392	0.003689678	0.031564933	DNA polymerase IV [Pyricularia oryzae 70-15]
MGG_00488	1458.501562	4285.848586	2.938528622	1.555093951	0.023399333	0.11616473	uncharacterized protein MGG_00488 [Pyricularia oryzae 70-15]
MGG_00491	683.9116682	1463.684794	2.14016643	1.097722992	0.00653897	0.048584348	superoxide dismutase [Pyricularia oryzae 70-15]
MGG_00497	1550.047052	749.62762	0.483616042	-1.048065994	0.009361843	0.062214261	uncharacterized protein MGG_00497 [Pyricularia oryzae 70-15]
MGG_00499	96.66542442	34.16574331	0.353443266	-1.50044944	0.00237016	0.02290589	acetylcholinesterase [Pyricularia oryzae 70-15]
MGG_00501	18663.97912	4921.406509	0.263684741	-1.923114005	2.82573E-06	9.17832E-05	CGBP1 [Pyricularia oryzae 70-15]
MGG_00505	6014.065174	12556.79558	2.087904806	1.062055936	0.008030112	0.05574274	septation protein SUN4 [Pyricularia oryzae 70-15]
MGG_00511	4.569972256	53.05818465	11.61017654	3.537318004	1.48394E-07	6.70143E-06	uncharacterized protein MGG_00511 [Pyricularia oryzae 70-15]
MGG_00513	65.85082888	24.01159322	0.364636158	-1.455470468	0.036253875	0.156174495	uncharacterized protein MGG_00513 [Pyricularia oryzae 70-15]
MGG_00518	1748.408196	5235.772995	2.994594173	1.582360503	0.012966542	0.077978985	proliferating cell nuclear antigen [Pyricularia oryzae 70-15]
MGG_00531	414.49551	171.1847319	0.412995383	-1.275802442	0.002342559	0.022711415	uncharacterized protein MGG_00531 [Pyricularia oryzae 70-15]
MGG_00537	577.3381064	1987.998627	3.443387168	1.783828404	1.23781E-05	0.000325474	ammonium transporter MEP1 [Pyricularia oryzae 70-15]
MGG_00539	3524.24662	10757.72524	3.052489339	1.609986257	7.21419E-05	0.001413515	uncharacterized protein MGG_00539 [Pyricularia oryzae 70-15]
MGG_00544	244.2687497	501.775956	2.054196276	1.038574036	0.013515476	0.080072179	uncharacterized protein MGG_00544 [Pyricularia oryzae 70-15]
MGG_00552	185.6700585	511.1679451	2.753098422	1.461056186	0.004172007	0.034737176	acid phosphatase [Pyricularia oryzae 70-15]
MGG_00562	692.6751012	1391.972498	2.009560464	1.006879986	0.010610708	0.067877241	NADPH-dependent 1-acyldihydroxyacetone phosphate reductase
MGG_00563	124.9872715	32.16921927	0.257379963	-1.958028353	5.14613E-05	0.001072303	uncharacterized protein MGG_00563 [Pyricularia oryzae 70-15]
MGG_00564	566.9730885	266.2076106	0.469524244	-1.090728442	0.008660477	0.058873793	uncharacterized protein MGG_00564 [Pyricularia oryzae 70-15]
MGG_00567	19048.6548	8881.654585	0.466261512	-1.100788749	0.005389893	0.042052933	uncharacterized protein MGG_00567 [Pyricularia oryzae 70-15]
MGG_00572	821.9814075	2381.931622	2.897792578	1.534954331	0.000168487	0.002858624	cytochrome P450 [Pyricularia oryzae 70-15]

MGG_00591	972.2311421	335.0422468	0.344611721	-1.536956324	0.000225006	0.003634799	uncharacterized protein MGG_00591 [Pyricularia oryzae 70-15]
MGG_00602	80261.82219	25227.84534	0.314319369	-1.669696916	5.88858E-05	0.001208352	cross-pathway control protein 1 [Pyricularia oryzae 70-15]
MGG_00606	1486.357506	246.5879315	0.165900822	-2.591607063	1.37362E-09	1.01748E-07	uncharacterized protein MGG_00606 [Pyricularia oryzae 70-15]
MGG_00611	1632.750204	600.0050553	0.367481231	-1.444257527	0.000359968	0.005274902	2OG-Fe(II) oxygenase [Pyricularia oryzae 70-15]
MGG_00613	484.8515324	1028.834688	2.121958206	1.085396241	0.00800067	0.055724809	uncharacterized protein MGG_00613 [Pyricularia oryzae 70-15]
MGG_00614	16.5468319	85.34392434	5.15771991	2.36673343	0.002513198	0.02388884	uncharacterized protein MGG_00614 [Pyricularia oryzae 70-15]
MGG_00619	57.00366713	22.94706343	0.402554162	-1.312745192	0.014636011	0.084463938	uncharacterized protein MGG_00619 [Pyricularia oryzae 70-15]
MGG_00623	1747.949686	857.7866199	0.490738736	-1.026972941	0.01136062	0.07110191	hexokinase-1 [Pyricularia oryzae 70-15]
MGG_00634	260.2811454	3194.916902	12.27486877	3.617635695	4.82944E-09	3.17124E-07	nitrite reductase [Pyricularia oryzae 70-15]
MGG_00635	2898.700399	775.3638606	0.26748672	-1.90246083	0.007752904	0.054534963	uncharacterized protein MGG_00635 [Pyricularia oryzae 70-15]
MGG_00636	1390.228822	512.209939	0.368435707	-1.440515208	0.000484536	0.006636007	GPI mannosyltransferase 2 [Pyricularia oryzae 70-15]
MGG_00637	3.776663273	0	0	-Inf	0.046205841	0.183494133	uncharacterized protein MGG_00637 [Pyricularia oryzae 70-15]
MGG_00649	5611.216637	546.6311091	0.097417573	-3.359674141	1.41629E-08	8.51736E-07	uncharacterized protein MGG_00649 [Pyricularia oryzae 70-15]
MGG_00651	163.8869129	1093.816998	6.674218085	2.738598828	1.40843E-10	1.31612E-08	isotrichodermin C-15 hydroxylase [Pyricularia oryzae 70-15]
MGG_00659	1537.516124	506.4287073	0.329381071	-1.602170448	0.010206331	0.066232107	glucan 1,3-beta-glucosidase [Pyricularia oryzae 70-15]
MGG_00660	4139.678784	1831.764409	0.442489503	-1.176284863	0.019584025	0.102903445	uncharacterized protein MGG_00660 [Pyricularia oryzae 70-15]
MGG_00677	661.3251411	224.404796	0.339325972	-1.559256239	0.001784475	0.01855976	endoglucanase-1 [Pyricularia oryzae 70-15]
MGG_00684	1297.471801	212.3143568	0.163636972	-2.611429348	0.015444055	0.087019659	uncharacterized protein MGG_00684 [Pyricularia oryzae 70-15]
MGG_00688	546.0957616	1177.061215	2.15541174	1.107963488	0.006533123	0.04857061	uncharacterized protein MGG_00688 [Pyricularia oryzae 70-15]
MGG_00702	999.3556858	482.4917855	0.482802862	-1.050493866	0.011084416	0.069913293	uncharacterized protein MGG_00702 [Pyricularia oryzae 70-15]
MGG_00703	142.3672214	1229.536971	8.636376822	3.110426192	1.59569E-12	2.20277E-10	MAS3 protein [Pyricularia oryzae 70-15]
MGG_00715	31935.17932	84087.19469	2.633058479	1.396739563	0.007012656	0.051040078	glucose-repressible protein [Pyricularia oryzae 70-15]
MGG_00722	628.4414915	274.1418507	0.436224938	-1.196855847	0.003281309	0.02884323	uncharacterized protein MGG_00722 [Pyricularia oryzae 70-15]
MGG_00737	8630.62897	18715.86693	2.168540322	1.116724269	0.005228069	0.041213875	uncharacterized protein MGG_00737 [Pyricularia oryzae 70-15]
MGG_00741	1336.803719	318.8355617	0.238505891	-2.067903196	2.6979E-05	0.000629062	uncharacterized protein MGG_00741 [Pyricularia oryzae 70-15]
MGG_00755	6173.782935	584.6910535	0.094705476	-3.400408338	5.72311E-15	1.1987E-12	indoleamine 2,3-dioxygenase [Pyricularia oryzae 70-15]
MGG_00759	1970.355259	935.5587105	0.474817273	-1.074555677	0.011857067	0.073190876	peroxin 24 [Pyricularia oryzae 70-15]
MGG_00779	217.3111288	95.62961787	0.440058539	-1.184232643	0.006027469	0.045849523	choline dehydrogenase [Pyricularia oryzae 70-15]
MGG_00798	113.4734135	25.86857635	0.227970373	-2.133081753	1.99964E-05	0.000484863	uncharacterized protein MGG_00798 [Pyricularia oryzae 70-15]
MGG_00805	1602.147773	406.0440695	0.25343734	-1.980298997	1.47046E-06	5.25386E-05	kynurenine 3-monooxygenase [Pyricularia oryzae 70-15]
MGG_00810	1223.937609	554.8620824	0.453341803	-1.141328896	0.005540119	0.042982981	palmitoyltransferase SWF1 [Pyricularia oryzae 70-15]
MGG_00814	2076.055773	6338.402833	3.053098532	1.61027415	7.87391E-05	0.001527991	hsp90-like protein [Pyricularia oryzae 70-15]
MGG_00815	863.9146735	243.5348953	0.281896931	-1.826760322	0.014866426	0.085227627	uncharacterized protein MGG_00815 [Pyricularia oryzae 70-15]
MGG_00817	1119.562181	544.7775204	0.486598717	-1.03919558	0.010308708	0.066682742	uncharacterized protein MGG_00817 [Pyricularia oryzae 70-15]
MGG_00822	337.3862403	4.294972406	0.012730135	-6.295608464	3.7469E-27	4.55173E-24	uncharacterized protein MGG_00822 [Pyricularia oryzae 70-15]
MGG_00834	4392.05109	615.3813983	0.140112532	-2.835342097	0.033288693	0.148238418	uncharacterized protein MGG_00834 [Pyricularia oryzae 70-15]
MGG_00835	46370.06048	2369.552539	0.051100915	-4.290507054	0.009934668	0.06520062	flotillin domain-containing protein [Pyricularia oryzae 70-15]
MGG_00836	232.0866221	49.53329019	0.213425874	-2.228193007	9.20743E-07	3.51567E-05	uncharacterized protein MGG_00836 [Pyricularia oryzae 70-15]
MGG_00848	1388.414855	2790.512259	2.009854799	1.007091278	0.023312911	0.115959318	uncharacterized protein MGG_00848 [Pyricularia oryzae 70-15]
MGG_00850	2017.315918	813.3224451	0.403170588	-1.310537699	0.001296184	0.014467249	uncharacterized protein MGG_00850 [Pyricularia oryzae 70-15]
MGG_00856	2643.394168	1256.80916	0.4754528	-1.072625967	0.014720627	0.084711593	uncharacterized protein MGG_00856 [Pyricularia oryzae 70-15]
MGG_00863	118.9834074	56.42272639	0.474206678	-1.076412114	0.021627746	0.110207155	uncharacterized protein MGG_00863 [Pyricularia oryzae 70-15]
MGG_00868	19.5533869	3.163122686	0.161768532	-2.627997104	0.002054244	0.020623932	global transactivator [Pyricularia oryzae 70-15]
MGG_00870	1403.925533	346.4881622	0.24679953	-2.018588446	1.18516E-06	4.3761E-05	uncharacterized protein MGG_00870 [Pyricularia oryzae 70-15]
MGG_00878	2865.349165	5939.375352	2.072827781	1.051600257	0.009407278	0.062447874	uncharacterized protein MGG_00878 [Pyricularia oryzae 70-15]
MGG_00887	792.4006405	2065.288103	2.606368543	1.382041097	0.013291334	0.079226262	uncharacterized protein MGG_00887 [Pyricularia oryzae 70-15]
MGG_00889	581.8130035	197.2091009	0.338956159	-1.56082941	0.000189863	0.0031509	modification methylase DdeI [Pyricularia oryzae 70-15]
MGG_00892	1961.84483	5421.858175	2.76365291	1.466576438	0.000294627	0.004530539	ATP synthase subunit 9 [Pyricularia oryzae 70-15]

MGG_00904	996.4515677	2538.102234	2.54714059	1.348878591	0.001092717	0.01266634	prefoldin subunit 3 [Pyricularia oryzae 70-15]
MGG_00906	712.3626579	1584.12514	2.223762184	1.15300251	0.004194274	0.034818332	uncharacterized protein MGG_00906 [Pyricularia oryzae 70-15]
MGG_00917	803.0021824	2056.531186	2.561053046	1.356737135	0.000922968	0.011035641	carrier protein YMC1 [Pyricularia oryzae 70-15]
MGG_00920	300.068731	674.5264875	2.247906622	1.168582107	0.004786613	0.038559532	YagE family protein [Pyricularia oryzae 70-15]
MGG_00930	384.1442216	112.1928708	0.292059244	-1.775667049	3.11837E-05	0.000712067	copper-transporting ATPase 1 [Pyricularia oryzae 70-15]
MGG_00936	1.743861961	27.07771107	15.52743948	3.95674804	0.001204172	0.013656001	uncharacterized protein MGG_00936 [Pyricularia oryzae 70-15]
MGG_00937	2251.87837	486.2323297	0.215922998	-2.211411179	0.001283821	0.014387326	ATP-binding cassette sub-family A member 8-A
MGG_00947	6591.842202	3139.710604	0.476302452	-1.070050119	0.006647147	0.04910664	uncharacterized protein MGG_00947 [Pyricularia oryzae 70-15]
MGG_00953	3284.302487	1065.829557	0.324522349	-1.62361026	6.9447E-05	0.001369549	uncharacterized protein MGG_00953 [Pyricularia oryzae 70-15]
MGG_00954	10.62347025	0.34480135	0.032456565	-4.94534588	0.000771007	0.009557335	uncharacterized protein MGG_00954 [Pyricularia oryzae 70-15]
MGG_00966	4103.151384	9336.290544	2.275395097	1.186117075	0.003252603	0.028673895	uncharacterized protein MGG_00966 [Pyricularia oryzae 70-15]
MGG_00967	608.8370711	1412.545497	2.320071435	1.214169227	0.005903098	0.045214904	DnaJ domain-containing protein [Pyricularia oryzae 70-15]
MGG_00989	14.72552191	3.456719135	0.234743404	-2.090843476	0.032628042	0.146272383	uncharacterized protein MGG_00989 [Pyricularia oryzae 70-15]
MGG_00992	1319.726182	3888.318623	2.946307103	1.558907815	0.000151213	0.002616713	uncharacterized protein MGG_00992 [Pyricularia oryzae 70-15]
MGG_00993	3490.887939	492.423384	0.141059637	-2.825622867	2.82338E-11	3.00864E-09	vacuolar iron transporter Ccc1 [Pyricularia oryzae 70-15]
MGG_00994	681.8939919	2910.209308	4.267832454	2.09350354	4.50275E-07	1.81124E-05	mannosyl-oligosaccharide 1,2-alpha-mannosidase IC
MGG_01001	1871.015267	802.2911324	0.428799886	-1.221623574	0.003103734	0.027948863	endo-1,3(4)-beta-glucanase 1 [Pyricularia oryzae 70-15]
MGG_01006	1323.001981	2686.532743	2.030633953	1.0219302	0.011729322	0.072623756	vacuolar transporter chaperone 1 [Pyricularia oryzae 70-15]
MGG_01008	4588.413308	1759.705737	0.38351073	-1.382661152	0.012228286	0.074622274	uncharacterized protein MGG_01008 [Pyricularia oryzae 70-15]
MGG_01017	2476.175527	645.249553	0.260583123	-1.940184444	2.87426E-06	9.26166E-05	uncharacterized protein MGG_01017 [Pyricularia oryzae 70-15]
MGG_01018	424.1179405	132.3863937	0.312145234	-1.679710658	7.73046E-05	0.001504961	uncharacterized protein MGG_01018 [Pyricularia oryzae 70-15]
MGG_01033	106.5971189	41.34217464	0.38783576	-1.366482263	0.004269991	0.035167357	uncharacterized protein MGG_01033 [Pyricularia oryzae 70-15]
MGG_01035	741.6632041	1954.236853	2.634938396	1.397769232	0.000622207	0.008058175	uncharacterized protein MGG_01035 [Pyricularia oryzae 70-15]
MGG_01036	1764.731554	453.599906	0.257036208	-1.959956495	2.16398E-06	7.35149E-05	uncharacterized protein MGG_01036 [Pyricularia oryzae 70-15]
MGG_01061	1391.246163	4458.896943	3.20496621	1.680309146	0.008111853	0.05611776	coproporphyrinogen III oxidase [Pyricularia oryzae 70-15]
MGG_01093	1479.640052	3319.230379	2.24326881	1.165602508	0.004157094	0.034645977	uncharacterized protein MGG_01093 [Pyricularia oryzae 70-15]
MGG_01096	2566.343055	1136.462545	0.442833449	-1.175163898	0.003303923	0.028979106	glucoamylase [Pyricularia oryzae 70-15]
MGG_01099	570.1949273	1151.032937	2.018665692	1.013402008	0.012731763	0.076947988	peptidyl-tRNA hydrolase 2 [Pyricularia oryzae 70-15]
MGG_01104	840.6257045	1775.718993	2.112377701	1.078867817	0.022477075	0.113261817	acetolactate synthase small subunit [Pyricularia oryzae 70-15]
MGG_01111	1369.978873	3375.811817	2.464134217	1.301080839	0.001843441	0.019026446	cytochrome c oxidase subunit 6B [Pyricularia oryzae 70-15]
MGG_01114	1308.483897	6946.596299	5.308889405	2.408410087	0.000198741	0.003275614	NADP-dependent mannitol dehydrogenase
MGG_01119	5.864596384	31.77899575	5.418786507	2.437969808	0.018536173	0.099066182	transmembrane and coiled-coil domain-containing protein 4
MGG_01125	3119.744112	1453.258666	0.465826239	-1.102136189	0.006531392	0.04857061	amine oxidase [Pyricularia oryzae 70-15]
MGG_01138	69.45748326	13.89746032	0.200085861	-2.321308874	2.394E-05	0.00056252	cytochrome P450 2C31 [Pyricularia oryzae 70-15]
MGG_01145	32.97436432	144.1281	4.370913677	2.127934886	1.23624E-05	0.000325474	uncharacterized protein MGG_01145 [Pyricularia oryzae 70-15]
MGG_01147	7074.832036	3397.535427	0.480228422	-1.058207303	0.033545958	0.149038243	alpha-N-arabinofuranosidase A [Pyricularia oryzae 70-15]
MGG_01160	9478.579222	21522.49874	2.270646079	1.183102853	0.016248703	0.090379689	histone H4 [Pyricularia oryzae 70-15]
MGG_01169	3701.516332	1416.240335	0.382610857	-1.386050284	0.001151915	0.013151747	uncharacterized protein MGG_01169 [Pyricularia oryzae 70-15]
MGG_01175	478.7983399	974.3756134	2.035043842	1.025059876	0.011963216	0.073584379	kinesin-II 85 kDa subunit [Pyricularia oryzae 70-15]
MGG_01188	2.014424045	15.34106274	7.615607439	2.928959114	0.003377083	0.029471327	uncharacterized protein MGG_01188 [Pyricularia oryzae 70-15]
MGG_01189	3456.813465	1332.51333	0.385474468	-1.37529279	0.001233086	0.013952525	uncharacterized protein MGG_01189 [Pyricularia oryzae 70-15]
MGG_01199	82.3357775	33.84324375	0.411039341	-1.282651611	0.009486474	0.062870521	uncharacterized protein MGG_01199 [Pyricularia oryzae 70-15]
MGG_01205	638.3946239	276.3326946	0.43285561	-1.208042236	0.003856605	0.032739371	tRNA-dihydrouridine synthase 3 [Pyricularia oryzae 70-15]
MGG_01214	53.9383789	15.83625175	0.293598956	-1.768081255	0.011802117	0.072962907	uncharacterized protein MGG_01214 [Pyricularia oryzae 70-15]
MGG_01221	212.0585631	105.4708663	0.497366693	-1.007618197	0.024939157	0.121329947	uncharacterized protein MGG_01221 [Pyricularia oryzae 70-15]
MGG_01230	1185.264147	3894.032705	3.285371209	1.716056388	1.67064E-05	0.000418453	succinate-semialdehyde dehydrogenase [Pyricularia oryzae 70-15]
MGG_01231	2207.74224	1047.986135	0.474686816	-1.074952114	0.028353001	0.132678067	sorbitol dehydrogenase [Pyricularia oryzae 70-15]
MGG_01272	837.1781901	1743.017468	2.082014903	1.057980395	0.009497202	0.062873031	CORD and CS domain-containing protein

MGG_01273	103.3115449	44.51073911	0.430839933	-1.21477612	0.011637946	0.072205194	uncharacterized protein MGG_01273 [Pyricularia oryzae 70-15]
MGG_01276	2.180124947	66.12857941	30.33247223	4.922791184	4.50497E-12	5.47264E-10	uncharacterized protein MGG_01276 [Pyricularia oryzae 70-15]
MGG_01287	2554.1796	5132.415981	2.009418594	1.006778132	0.012100251	0.074161392	farnesyltransferase subunit beta [Pyricularia oryzae 70-15]
MGG_01290	2197.535377	1076.524463	0.489878103	-1.029505288	0.010106643	0.065878096	nicotinamide mononucleotide adenylyl transferase
MGG_01299	1734.888059	698.079944	0.402377514	-1.31337841	0.001333579	0.014754387	uncharacterized protein MGG_01299 [Pyricularia oryzae 70-15]
MGG_01300	506.3848224	1841.884905	3.637322494	1.862876844	7.92578E-06	0.000226547	L-galactonate dehydratase [Pyricularia oryzae 70-15]
MGG_01303	543.6889049	1205.497621	2.217256246	1.148775511	0.005037643	0.039972105	3-keto-steroid reductase [Pyricularia oryzae 70-15]
MGG_01304	540.7201806	128.3592418	0.237385706	-2.074695028	1.0947E-06	4.07927E-05	uncharacterized protein MGG_01304 [Pyricularia oryzae 70-15]
MGG_01308	838.7079399	1713.893544	2.04349269	1.031037082	0.011269548	0.070750629	GTPase binding protein Rid1 [Pyricularia oryzae 70-15]
MGG_01324	1733.017471	691.9361445	0.399266687	-1.324575389	0.000979684	0.01157705	urease [Pyricularia oryzae 70-15]
MGG_01328	100.778103	31.77321302	0.315278935	-1.665299312	0.000632504	0.00817411	endoglucanase [Pyricularia oryzae 70-15]
MGG_01336	1446.213116	646.3322923	0.446913588	-1.161932187	0.004115855	0.034340252	bacteriodes thetaiotaomicron symbiotic chitinase
MGG_01337	602.0225193	217.9128298	0.361967905	-1.466066315	0.000402602	0.005733663	uncharacterized protein MGG_01337 [Pyricularia oryzae 70-15]
MGG_01342	1210.247604	235.1795654	0.194323512	-2.363467622	2.85094E-08	1.54886E-06	sensory/regulatory protein rpfC [Pyricularia oryzae 70-15]
MGG_01343	2390.927511	946.9689496	0.396067612	-1.336181364	0.003562651	0.030564323	uncharacterized protein MGG_01343 [Pyricularia oryzae 70-15]
MGG_01349	2624.680082	673.2522598	0.256508313	-1.962922513	1.90301E-06	6.64303E-05	uncharacterized protein MGG_01349 [Pyricularia oryzae 70-15]
MGG_01356	54.55780354	118.3710063	2.169643912	1.117458283	0.018227372	0.097927425	uncharacterized protein MGG_01356 [Pyricularia oryzae 70-15]
MGG_01359	2016.256113	4158.211426	2.062342873	1.044284207	0.009523861	0.062980872	saccharopine dehydrogenase [Pyricularia oryzae 70-15]
MGG_01364	6355.030331	2376.331369	0.373929194	-1.419162983	0.000450904	0.006260088	uncharacterized protein MGG_01364 [Pyricularia oryzae 70-15]
MGG_01368	69.30200822	336.7919191	4.859771424	2.280888459	6.49036E-05	0.001298927	uncharacterized protein MGG_01368 [Pyricularia oryzae 70-15]
MGG_01382	4230.678843	1452.341133	0.343287966	-1.542508812	0.000134622	0.002377019	short chain dehydrogenase [Pyricularia oryzae 70-15]
MGG_01385	2192.8356	788.0857291	0.35939116	-1.476373174	0.000320163	0.004813541	choline transporter [Pyricularia oryzae 70-15]
MGG_01387	830.2913327	414.0121154	0.498634755	-1.003944652	0.012873338	0.077533618	endonuclease/exonuclease/phosphatase
MGG_01391	565.4993119	229.3149723	0.405508844	-1.302194715	0.040298608	0.167710687	ent-kaurene oxidase [Pyricularia oryzae 70-15]
MGG_01402	153.1054732	59.84235123	0.390857035	-1.355287092	0.002736459	0.025583964	BNR/Asp-box repeat domain-containing protein
MGG_01405	6.093885993	0.34480135	0.056581523	-4.143525182	0.019647916	0.103107564	uncharacterized protein MGG_01405 [Pyricularia oryzae 70-15]
MGG_01413	167.9361618	66.1899799	0.394137744	-1.343228182	0.002433371	0.023386548	uncharacterized protein MGG_01413 [Pyricularia oryzae 70-15]
MGG_01417	396.8544985	180.7390114	0.455428909	-1.134702222	0.006645106	0.04910664	uncharacterized protein MGG_01417 [Pyricularia oryzae 70-15]
MGG_01419	1205.573038	515.7570722	0.427810722	-1.224955453	0.002429851	0.023371204	small nuclear ribonucleoprotein U1a [Pyricularia oryzae 70-15]
MGG_01420	834.2269518	98.93571883	0.118595687	-3.075876546	0.001564292	0.016698616	uncharacterized protein MGG_01420 [Pyricularia oryzae 70-15]
MGG_01427	313.760175	655.826515	2.090215927	1.063651986	0.009118009	0.061230276	SET1 complex component ash2 [Pyricularia oryzae 70-15]
MGG_01430	466.3421439	208.580477	0.447269199	-1.160784685	0.00496772	0.039520536	uncharacterized protein MGG_01430 [Pyricularia oryzae 70-15]
MGG_01435	91.24316797	30.11934922	0.330099775	-1.59902594	0.001250048	0.014099896	uncharacterized protein MGG_01435 [Pyricularia oryzae 70-15]
MGG_01437	184.1197243	370.6090828	2.012870072	1.009254051	0.026105444	0.125545895	cyclin domain-containing protein [Pyricularia oryzae 70-15]
MGG_01444	6071.28301	12442.11958	2.049339416	1.035158946	0.010293215	0.066617998	uncharacterized protein MGG_01444 [Pyricularia oryzae 70-15]
MGG_01484	3067.252821	6686.985029	2.180121894	1.124408801	0.005004341	0.039759797	uncharacterized protein MGG_01484 [Pyricularia oryzae 70-15]
MGG_01487	68.06190488	18.02688534	0.264860135	-1.916697378	0.000397785	0.0056717	uncharacterized protein MGG_01487 [Pyricularia oryzae 70-15]
MGG_01507	1595.335894	771.1799632	0.48339661	-1.048720739	0.00921231	0.061523444	acetylglutamate synthase [Pyricularia oryzae 70-15]
MGG_01518	2151.133634	723.4876455	0.336328545	-1.572056865	0.000124854	0.002240985	nitrogen assimilation transcription factor nit-4
MGG_01519	1316.693193	2683.562066	2.038107343	1.027230038	0.011609343	0.072101377	uncharacterized protein MGG_01519 [Pyricularia oryzae 70-15]
MGG_01535	719.0663801	4732.524511	6.581484884	2.718413115	0.004819592	0.038748116	pyridoxamine 5'-phosphate oxidase [Pyricularia oryzae 70-15]
MGG_01539	6802.934694	2724.569874	0.400499196	-1.320128749	0.001036964	0.012114572	uncharacterized protein MGG_01539 [Pyricularia oryzae 70-15]
MGG_01540	477.9454447	989.7411838	2.070824599	1.050205361	0.010635559	0.068000407	uncharacterized protein MGG_01540 [Pyricularia oryzae 70-15]
MGG_01548	1050.543519	219.9747061	0.209391331	-2.255726377	1.05291E-07	4.99639E-06	uncharacterized protein MGG_01548 [Pyricularia oryzae 70-15]
MGG_01552	406.4705144	183.3033127	0.450963369	-1.148917844	0.005705183	0.043976248	uncharacterized protein MGG_01552 [Pyricularia oryzae 70-15]
MGG_01556	1370.77411	3092.017359	2.255672423	1.173557569	0.009441037	0.062603561	V-type proton ATPase subunit F [Pyricularia oryzae 70-15]
MGG_01566	635.2462879	1318.546239	2.075645721	1.05356022	0.010059557	0.065665504	homoisocitrate dehydrogenase [Pyricularia oryzae 70-15]
MGG_01569	9545.592839	25430.51743	2.664110848	1.413654111	0.030147652	0.138724877	minor allergen Alt a 7 [Pyricularia oryzae 70-15]

MGG_01571	3.180407306	363.6152368	114.3297703	6.837057305	2.02467E-29	3.55381E-26	tyrosinase [Pyricularia oryzae 70-15]
MGG_01589	3992.366041	8394.616939	2.102667153	1.072220493	0.006701234	0.049277597	uncharacterized protein MGG_01589 [Pyricularia oryzae 70-15]
MGG_01612	66.48740932	28.74258907	0.432301234	-1.209891141	0.015869697	0.088914242	uncharacterized protein MGG_01612 [Pyricularia oryzae 70-15]
MGG_01655	5760.017762	1833.649222	0.318340897	-1.651355583	4.78409E-05	0.001008979	copper radical oxidase [Pyricularia oryzae 70-15]
MGG_01657	2211.071601	830.4231505	0.37557497	-1.412827176	0.000517801	0.006981411	uncharacterized protein MGG_01657 [Pyricularia oryzae 70-15]
MGG_01659	1349.305751	643.5777072	0.476969513	-1.06803104	0.007301679	0.052489992	uncharacterized protein MGG_01659 [Pyricularia oryzae 70-15]
MGG_01668	924.2284639	410.7239438	0.444396553	-1.170080468	0.02091654	0.107849798	uncharacterized protein MGG_01668 [Pyricularia oryzae 70-15]
MGG_01675	560.7274565	1192.939511	2.127485461	1.089149273	0.010531617	0.067656313	uncharacterized protein MGG_01675 [Pyricularia oryzae 70-15]
MGG_01689	5574.917411	11869.98471	2.129176782	1.090295739	0.006980465	0.050838542	rho GDP dissociation inhibitor [Pyricularia oryzae 70-15]
MGG_01692	253.2917426	1436.92381	5.672999028	2.504111617	4.1279E-09	2.75526E-07	uncharacterized protein MGG_01692 [Pyricularia oryzae 70-15]
MGG_01709	321.2167409	976.8715525	3.041160152	1.604621793	0.000703451	0.008883079	uncharacterized protein MGG_01709 [Pyricularia oryzae 70-15]
MGG_01719	2360.896025	6097.442767	2.582681618	1.368869805	0.000572508	0.007494427	electron transfer flavoprotein subunit alpha
MGG_01723	305.5803474	991.1902587	3.243632214	1.697610246	4.80536E-05	0.001011707	cytochrome b2 [Pyricularia oryzae 70-15]
MGG_01731	635.4821252	291.9670023	0.459441723	-1.122046219	0.006306645	0.047467857	uncharacterized protein MGG_01731 [Pyricularia oryzae 70-15]
MGG_01732	1699.297376	562.7802625	0.331184094	-1.594294708	8.73749E-05	0.00167418	3-carboxymuconate cyclase [Pyricularia oryzae 70-15]
MGG_01750	1298.422874	506.021806	0.389720342	-1.35948886	0.000799346	0.00983835	uncharacterized protein MGG_01750 [Pyricularia oryzae 70-15]
MGG_01758	794.5442343	283.2297031	0.356468137	-1.488154967	0.016023767	0.089536193	leucine carboxyl methyltransferase 1 [Pyricularia oryzae 70-15]
MGG_01764	133.5256721	64.82522606	0.485488858	-1.042489908	0.025782769	0.124536411	uncharacterized protein MGG_01764 [Pyricularia oryzae 70-15]
MGG_01777	1310.344571	541.6370389	0.413354663	-1.274547932	0.001695108	0.017797896	C6 zinc finger domain-containing protein [Pyricularia oryzae 70-15]
MGG_01806	662.667031	175.605408	0.264997955	-1.915946869	6.56326E-05	0.001311357	uncharacterized protein MGG_01806 [Pyricularia oryzae 70-15]
MGG_01811	925.5561394	342.7225037	0.370288186	-1.433279575	0.000613818	0.007975035	uncharacterized protein MGG_01811 [Pyricularia oryzae 70-15]
MGG_01827	29.59208055	4.419279965	0.149339955	-2.743327898	0.001632366	0.017258467	uncharacterized protein MGG_01827 [Pyricularia oryzae 70-15]
MGG_01853	6.497912386	128.6389949	19.79697283	4.307207938	1.17993E-05	0.000314497	uncharacterized protein MGG_01853 [Pyricularia oryzae 70-15]
MGG_01864	78.74417243	306.5017745	3.892374064	1.960650362	0.000208345	0.003415628	uncharacterized protein MGG_01864 [Pyricularia oryzae 70-15]
MGG_01865	617.6323714	242.8178225	0.393142966	-1.346874052	0.001150595	0.013149034	uncharacterized protein MGG_01865 [Pyricularia oryzae 70-15]
MGG_01867	475.5050853	167.883191	0.353062872	-1.50200298	0.000417181	0.005895846	uncharacterized protein MGG_01867 [Pyricularia oryzae 70-15]
MGG_01876	119.6438945	355.9419223	2.975011167	1.572895084	0.000246073	0.00392812	chitinase 3 [Pyricularia oryzae 70-15]
MGG_01877	385.1286143	1519.424449	3.945239053	1.98011272	2.86026E-06	9.26166E-05	MFS transporter [Pyricularia oryzae 70-15]
MGG_01890	322.6745142	146.0672894	0.452676871	-1.1434465	0.034133278	0.150296961	uncharacterized protein MGG_01890 [Pyricularia oryzae 70-15]
MGG_01906	21.920339	75.74174057	3.455317938	1.788818465	0.000729992	0.009151638	nicotianamine synthase 3 [Pyricularia oryzae 70-15]
MGG_01917	666.8885482	1976.70581	2.964072206	1.567580593	0.000147826	0.002565413	DNA replication complex GINS protein PSF2
MGG_01920	517.4732858	152.7931887	0.295267781	-1.759904155	0.000184689	0.003086104	C2H2 type zinc finger domain-containing protein
MGG_01924	33.32502787	130.6403893	3.920188449	1.970923008	3.79027E-05	0.000834134	benzoate 4-monooxygenase cytochrome P450
MGG_01931	953.2159777	3188.922366	3.345435285	1.742193937	2.28775E-05	0.000542805	siderophore iron transporter mirB [Pyricularia oryzae 70-15]
MGG_01947	0	9.753620553	Inf	Inf	0.000440257	0.006154485	cytochrome P450 3A19 [Pyricularia oryzae 70-15]
MGG_01950	0	37.18810247	Inf	Inf	2.29531E-11	2.48959E-09	uncharacterized protein MGG_01950 [Pyricularia oryzae 70-15]
MGG_01951	21.41566386	85.28384029	3.982311304	1.993606003	0.00014273	0.002494806	4-coumarate-CoA ligase 2 [Pyricularia oryzae 70-15]
MGG_01956	44.99457089	105.6500834	2.348062918	1.231471067	0.008922468	0.060224412	uncharacterized protein MGG_01956 [Pyricularia oryzae 70-15]
MGG_01961	29.88220988	132.898306	4.447405548	2.152963966	1.06229E-05	0.000289342	UDP-glucuronosyl/UDP-glucosyltransferase
MGG_01963	3.790522598	67.90749575	17.91507477	4.163102159	2.76261E-10	2.50449E-08	uncharacterized protein MGG_01963 [Pyricularia oryzae 70-15]
MGG_01979	1976.862773	4254.963969	2.152382061	1.105934188	0.006437669	0.048215045	T-complex protein 1 subunit beta [Pyricularia oryzae 70-15]
MGG_01984	19.74561647	72.81074021	3.687438187	1.882618866	0.004626626	0.037645181	uncharacterized protein MGG_01984 [Pyricularia oryzae 70-15]
MGG_01985	1901.317036	4006.107394	2.107017041	1.075201982	0.007754261	0.054534963	Delta(12) fatty acid desaturase [Pyricularia oryzae 70-15]
MGG_01990	4201.223066	2024.073475	0.481781958	-1.053547727	0.009246535	0.061650334	IDI4 [Pyricularia oryzae 70-15]
MGG_01992	0	10.00471774	Inf	Inf	0.000310436	0.004708091	uncharacterized protein MGG_01992 [Pyricularia oryzae 70-15]
MGG_02000	373.4808914	875.0915515	2.343069141	1.228399527	0.003095398	0.027916034	uncharacterized protein MGG_02000 [Pyricularia oryzae 70-15]
MGG_02001	657.3691453	2223.396399	3.382264614	1.757989534	2.07773E-05	0.000500975	uncharacterized protein MGG_02001 [Pyricularia oryzae 70-15]
MGG_02004	5027.358839	16862.20158	3.354087528	1.745920338	2.20628E-05	0.000527596	uncharacterized protein MGG_02004 [Pyricularia oryzae 70-15]

MGG_02041	499.4773415	2091.093101	4.186562488	2.065766159	0.018165179	0.097782382	uncharacterized protein MGG_02041 [Pyricularia oryzae 70-15]
MGG_02043	484.0265023	161.5953993	0.333856511	-1.582699918	0.000164236	0.002798233	uncharacterized protein MGG_02043 [Pyricularia oryzae 70-15]
MGG_02058	86.31557074	196.5636346	2.277267392	1.187303699	0.007302279	0.052489992	mitochondrial chaperone BCS1 [Pyricularia oryzae 70-15]
MGG_02067	65.90107371	24.63428002	0.37380696	-1.419634664	0.005711022	0.043993342	uncharacterized protein MGG_02067, partial
MGG_02069	3359.90185	503.9118904	0.149978158	-2.737175686	1.10844E-10	1.07723E-08	glyoxalase/bleomycin resistance protein/dioxygenase
MGG_02074	43644.8033	17642.64037	0.404232326	-1.306743397	0.001330279	0.014744737	potassium/sodium efflux P-type ATPase [Pyricularia oryzae 70-15]
MGG_02082	79.35086421	28.28140401	0.356409527	-1.488392192	0.005946271	0.045431007	uncharacterized protein MGG_02082 [Pyricularia oryzae 70-15]
MGG_02083	104.689567	32.229093	0.307853915	-1.699682182	0.024132517	0.118640961	uncharacterized protein MGG_02083 [Pyricularia oryzae 70-15]
MGG_02084	1050.146511	3520.839155	3.352712328	1.745328701	0.000540567	0.007216273	uncharacterized protein MGG_02084 [Pyricularia oryzae 70-15]
MGG_02086	2998.024928	6108.136316	2.037386767	1.02671988	0.010343917	0.066768283	proteasome component PRE3 [Pyricularia oryzae 70-15]
MGG_02101	2903.653105	656.3438419	0.226040721	-2.145345397	2.03735E-07	8.83919E-06	uncharacterized protein MGG_02101 [Pyricularia oryzae 70-15]
MGG_02104	247.2967044	106.9711154	0.432561831	-1.209021725	0.005349964	0.041875878	uncharacterized protein MGG_02104 [Pyricularia oryzae 70-15]
MGG_02106	1004.743861	259.4472936	0.258222323	-1.953314371	1.49552E-05	0.000380074	uncharacterized protein MGG_02106 [Pyricularia oryzae 70-15]
MGG_02115	28.50110184	65.16428149	2.286377624	1.193063702	0.022519063	0.113322941	oxidoreductase [Pyricularia oryzae 70-15]
MGG_02119	4.721813832	43.49615478	9.211747079	3.203474801	0.000954272	0.011361075	high-affinity nicotinic acid transporter [Pyricularia oryzae 70-15]
MGG_02120	16.35460233	154.4217234	9.442095892	3.239107135	0.00106963	0.012422435	sterigmatocystin 8-O-methyltransferase [Pyricularia oryzae 70-15]
MGG_02125	201.0918553	521.8260967	2.594963858	1.375714445	0.001109737	0.012839132	branched-chain-amino-acid aminotransferase 1
MGG_02127	3224.2237	1498.638063	0.464805858	-1.105299844	0.006178111	0.04661596	alcohol oxidase [Pyricularia oryzae 70-15]
MGG_02128	260.4807878	46.8624336	0.179907447	-2.474673186	6.49073E-08	3.21834E-06	uncharacterized protein MGG_02128 [Pyricularia oryzae 70-15]
MGG_02143	1.422686019	22.78600252	16.01618503	4.001458641	1.70213E-05	0.000424589	uncharacterized protein MGG_02143 [Pyricularia oryzae 70-15]
MGG_02154	0	13.45273123	Inf	Inf	2.75164E-05	0.000636704	uncharacterized protein MGG_02154 [Pyricularia oryzae 70-15]
MGG_02155	0	19.37504651	Inf	Inf	0.001614779	0.017117226	uncharacterized protein MGG_02155 [Pyricularia oryzae 70-15]
MGG_02157	1703.880781	558.3572673	0.32769738	-1.609563957	8.03209E-05	0.001551253	uncharacterized protein MGG_02157 [Pyricularia oryzae 70-15]
MGG_02158	952.0249424	345.4710169	0.362880216	-1.46243469	0.002968488	0.027093312	plasma membrane iron permease [Pyricularia oryzae 70-15]
MGG_02160	2.689012521	15.11111833	5.619579015	2.490462056	0.007915634	0.05532746	uncharacterized protein MGG_02160 [Pyricularia oryzae 70-15]
MGG_02191	16.41003963	43.4032324	2.644919414	1.403223767	0.01533632	0.086734458	uncharacterized protein MGG_02191 [Pyricularia oryzae 70-15]
MGG_02203	22.53585671	77.26459559	3.428518231	1.777585194	0.000740387	0.009249522	uncharacterized protein MGG_02203 [Pyricularia oryzae 70-15]
MGG_02204	10.68311999	29.79192233	2.788691164	1.479588171	0.021200349	0.108705905	vitamin H transporter [Pyricularia oryzae 70-15]
MGG_02206	95.65244068	858.6550365	8.976823073	3.166204961	1.90172E-12	2.5111E-10	uncharacterized protein MGG_02206 [Pyricularia oryzae 70-15]
MGG_02208	834.734363	1745.28579	2.090827774	1.064074229	0.009092292	0.061091354	uncharacterized protein MGG_02208 [Pyricularia oryzae 70-15]
MGG_02225	10.4999263	51.30775686	4.886487332	2.288797751	0.010280553	0.066571515	2,3-dihydro-2,3-dihydroxybenzoate dehydrogenase
MGG_02234	1320.417442	322.2813164	0.244075325	-2.034601642	1.25205E-06	4.56755E-05	uncharacterized protein MGG_02234 [Pyricularia oryzae 70-15]
MGG_02236	2.941197338	65.99797466	22.43915218	4.487946262	3.96112E-06	0.000123701	uncharacterized protein MGG_02236 [Pyricularia oryzae 70-15]
MGG_02239	142.1449568	308.4608002	2.170043927	1.117724246	0.018598503	0.099145935	uncharacterized protein MGG_02239 [Pyricularia oryzae 70-15]
MGG_02245	1809.158041	222.884764	0.123198062	-3.020948536	3.99507E-12	4.95226E-10	endoglucanase type F [Pyricularia oryzae 70-15]
MGG_02246	286.4611999	6876.53397	24.00511473	4.585269926	6.44003E-05	0.001295257	uncharacterized protein MGG_02246 [Pyricularia oryzae 70-15]
MGG_02254	3265.496523	6536.934714	2.00181953	1.001311917	0.013310908	0.079244576	uncharacterized protein MGG_02254 [Pyricularia oryzae 70-15]
MGG_02255	0.660729151	40.89091784	61.88756435	5.951577639	2.94755E-10	2.61364E-08	uncharacterized protein MGG_02255 [Pyricularia oryzae 70-15]
MGG_02267	2489.627132	5100.127373	2.048550687	1.03460359	0.013150511	0.078734552	uncharacterized protein MGG_02267 [Pyricularia oryzae 70-15]
MGG_02272	205.2359048	74.679252	0.363870308	-1.458503762	0.001165817	0.013297973	ent-kaurene oxidase [Pyricularia oryzae 70-15]
MGG_02273	685.2081762	120.6315891	0.176051007	-2.505934619	6.62906E-08	3.24455E-06	uncharacterized protein MGG_02273 [Pyricularia oryzae 70-15]
MGG_02274	74.75738574	1.529115409	0.020454372	-5.611446917	8.78622E-11	8.74877E-09	uncharacterized protein MGG_02274 [Pyricularia oryzae 70-15]
MGG_02275	48.4982286	891.427363	18.38061696	4.200113287	3.46041E-15	7.50661E-13	endoprotease [Pyricularia oryzae 70-15]
MGG_02283	0	17.87323383	Inf	Inf	1.48812E-06	5.30136E-05	uncharacterized protein MGG_02283 [Pyricularia oryzae 70-15]
MGG_02287	0.679106417	12.024803	17.70680219	4.146231783	0.001128051	0.012977531	O-methyltransferase [Pyricularia oryzae 70-15]
MGG_02288	0.660729151	8.547445547	12.93638329	3.693362425	0.010233952	0.06637099	uncharacterized protein MGG_02288 [Pyricularia oryzae 70-15]
MGG_02290	26.35498244	82.25325315	3.120975449	1.641997008	0.001196729	0.013599497	uncharacterized protein MGG_02290 [Pyricularia oryzae 70-15]
MGG_02296	0	4.176888417	Inf	Inf	0.042855365	0.174641723	uncharacterized protein MGG_02296 [Pyricularia oryzae 70-15]

MGG_02303	365.313835	1349.682372	3.694583241	1.885411634	0.000614659	0.007977436	phosphoribulokinase/uridine kinase [Pyricularia oryzae 70-15]
MGG_02304	44.64479182	210.3125811	4.710797665	2.235971368	1.33058E-06	4.81068E-05	leucoanthocyanidin reductase [Pyricularia oryzae 70-15]
MGG_02305	14.68907287	147.4487524	10.03798903	3.32739837	5.84016E-10	4.72975E-08	uncharacterized protein MGG_02305 [Pyricularia oryzae 70-15]
MGG_02306	45.37325859	318.595645	7.021661103	2.811812367	6.9091E-10	5.52182E-08	abhydrolase [Pyricularia oryzae 70-15]
MGG_02309	1446.384683	4845.9026	3.350355308	1.744314103	2.29987E-05	0.000543558	carboxypeptidase S1 [Pyricularia oryzae 70-15]
MGG_02313	0.642351884	154.6952636	240.826356	7.911849479	8.2595E-24	7.71819E-21	uncharacterized protein MGG_02313 [Pyricularia oryzae 70-15]
MGG_02318	3.45096939	24.07024432	6.97492258	2.802177203	0.000441783	0.006161626	alpha-ketoglutarate-dependent taurine dioxygenase
MGG_02319	0.743579602	7.113293742	9.566284127	3.257958641	0.026650417	0.127510542	pantothenate transporter liz1 [Pyricularia oryzae 70-15]
MGG_02320	14.99156605	86.34932538	5.759860251	2.526033809	2.87305E-06	9.26166E-05	thiol-specific monooxygenase [Pyricularia oryzae 70-15]
MGG_02327	2.698048403	11.22160279	4.159155478	2.056290617	0.046416305	0.184089216	uncharacterized protein MGG_02327 [Pyricularia oryzae 70-15]
MGG_02328	101.8690817	269.5971404	2.646506044	1.404088949	0.001487146	0.016015823	methionine permease [Pyricularia oryzae 70-15]
MGG_02329	1.858949005	44.02547836	23.68299413	4.565779581	4.12468E-09	2.75526E-07	isotrichodermin C-15 hydroxylase [Pyricularia oryzae 70-15]
MGG_02331	2885.035176	7703.116728	2.670025237	1.416853378	0.020202446	0.105014684	uncharacterized protein MGG_02331 [Pyricularia oryzae 70-15]
MGG_02334	5.285833207	43.36147811	8.203338323	3.03621113	8.75644E-06	0.000246806	uncharacterized protein MGG_02334 [Pyricularia oryzae 70-15]
MGG_02335	12.68399021	30.39897176	2.39664106	1.261013856	0.044218119	0.178577698	uncharacterized protein MGG_02335 [Pyricularia oryzae 70-15]
MGG_02336	0.371789801	5.523045168	14.85528961	3.892904826	0.037017934	0.158120204	isoflavone reductase [Pyricularia oryzae 70-15]
MGG_02337	50.96796742	326.7684878	6.411252093	2.680606137	5.29191E-09	3.45624E-07	retinol dehydrogenase 8 [Pyricularia oryzae 70-15]
MGG_02338	1.642634244	9.3064094	5.665539626	2.502213375	0.037837932	0.160887366	uncharacterized protein MGG_02338 [Pyricularia oryzae 70-15]
MGG_02339	19.25266268	60.47622574	3.141187624	1.651310118	0.002558215	0.024203425	uncharacterized protein MGG_02339 [Pyricularia oryzae 70-15]
MGG_02342	60.64027369	151.4819544	2.498042062	1.320797769	0.004068093	0.034058711	uncharacterized protein MGG_02342 [Pyricularia oryzae 70-15]
MGG_02345	3.506101189	105.5186601	30.09572581	4.911486705	1.09835E-12	1.56973E-10	uncharacterized protein MGG_02345 [Pyricularia oryzae 70-15]
MGG_02346	797.1428046	2440.371834	3.061398559	1.61419088	6.00542E-05	0.001224058	uncharacterized protein MGG_02346 [Pyricularia oryzae 70-15]
MGG_02351	38.66510839	111.4059959	2.881305666	1.526722718	0.001671073	0.017591158	tyrocidine synthetase 1 [Pyricularia oryzae 70-15]
MGG_02354	11.50955005	125.8192835	10.93172913	3.450449713	1.3307E-10	1.26291E-08	uncharacterized protein MGG_02354 [Pyricularia oryzae 70-15]
MGG_02355	97.21314098	242.0829343	2.4902285	1.316278128	0.002335995	0.022683991	uncharacterized protein MGG_02355 [Pyricularia oryzae 70-15]
MGG_02372	28.04252262	79.09526811	2.820547537	1.495975252	0.003238673	0.028588124	uncharacterized protein MGG_02372 [Pyricularia oryzae 70-15]
MGG_02379	570.0718921	230.2366035	0.403872927	-1.308026656	0.028078024	0.131709675	uncharacterized protein MGG_02379 [Pyricularia oryzae 70-15]
MGG_02382	6.277964162	154.4627898	24.60396169	4.62081873	1.88071E-12	2.51065E-10	uncharacterized protein MGG_02382 [Pyricularia oryzae 70-15]
MGG_02383	69.26681269	265.2528001	3.829435624	1.937131786	1.69459E-05	0.000423579	uncharacterized protein MGG_02383 [Pyricularia oryzae 70-15]
MGG_02387	2065.328657	754.7618035	0.365443922	-1.452278058	0.000394109	0.005625899	uncharacterized protein MGG_02387 [Pyricularia oryzae 70-15]
MGG_02403	10013.32218	2024.056008	0.202136311	-2.306599588	3.11071E-08	1.67951E-06	hypothetical protein, variant [Pyricularia oryzae 70-15]
MGG_02404	177.9217981	58.53247018	0.328978634	-1.603934204	0.000337727	0.005027825	uncharacterized protein MGG_02404 [Pyricularia oryzae 70-15]
MGG_02407	1650.303992	3989.898752	2.41767503	1.273620339	0.046688762	0.184507184	uncharacterized protein MGG_02407 [Pyricularia oryzae 70-15]
MGG_02408	1215.415079	523.6570042	0.43084623	-1.214755036	0.002545479	0.0241205	uncharacterized protein MGG_02408 [Pyricularia oryzae 70-15]
MGG_02409	9067.214787	2450.243703	0.270231131	-1.887734213	4.22598E-06	0.000130629	nonspecific lipid-transfer protein [Pyricularia oryzae 70-15]
MGG_02410	67248.15911	15651.11989	0.232736778	-2.103228884	0.004897259	0.039165179	erythrocyte band 7 integral membrane protein
MGG_02420	653.5694084	103.4020582	0.158211288	-2.660075555	1.55463E-05	0.000392634	uncharacterized protein MGG_02420 [Pyricularia oryzae 70-15]
MGG_02421	148.8233139	63.50793562	0.426733782	-1.228591769	0.030931741	0.140891933	uncharacterized protein MGG_02421 [Pyricularia oryzae 70-15]
MGG_02434	912.6519334	1945.741402	2.131964368	1.092183326	0.007650602	0.054160558	AP-3 complex subunit sigma [Pyricularia oryzae 70-15]
MGG_02449	2479.964186	5773.468416	2.328045078	1.219118993	0.002609699	0.024594743	tyrosyl-tRNA synthetase [Pyricularia oryzae 70-15]
MGG_02451	1197.760826	3638.628735	3.037859193	1.603055001	0.000100536	0.001893499	cyanate hydratase [Pyricularia oryzae 70-15]
MGG_02502	975.8736805	331.9660009	0.340173126	-1.555658922	0.037900606	0.161041119	uncharacterized protein MGG_02502 [Pyricularia oryzae 70-15]
MGG_02507	1214.597163	417.0864289	0.343394865	-1.542059629	0.009715527	0.063831382	inulinase [Pyricularia oryzae 70-15]
MGG_02509	175.6434679	87.49470817	0.498138127	-1.005382257	0.021134204	0.108603347	juvenile hormone epoxide hydrolase 2 [Pyricularia oryzae 70-15]
MGG_02514	720.2462668	333.684225	0.463291849	-1.110006793	0.006489153	0.048421146	ankyrin repeat protein nuc-2 [Pyricularia oryzae 70-15]
MGG_02519	677.4311972	1412.797132	2.085521213	1.060407987	0.009044988	0.060840815	uncharacterized protein MGG_02519 [Pyricularia oryzae 70-15]
MGG_02521	6068.712533	2572.75936	0.423938248	-1.238073961	0.001860538	0.019176815	carboxylesterase 3 [Pyricularia oryzae 70-15]
MGG_02529	288.9923066	1437.703245	4.974884147	2.314662929	5.2849E-08	2.7089E-06	uncharacterized protein MGG_02529 [Pyricularia oryzae 70-15]

MGG_02532	48.5950019	101.843221	2.095755058	1.067470112	0.026461771	0.12665784	endoglucanase 1 [Pyricularia oryzae 70-15]
MGG_02535	1422.062502	585.266098	0.411561445	-1.280820258	0.014956413	0.085538429	uncharacterized protein MGG_02535 [Pyricularia oryzae 70-15]
MGG_02545	105.6721466	44.35011904	0.419695449	-1.252585275	0.009137197	0.061287119	uncharacterized protein MGG_02545 [Pyricularia oryzae 70-15]
MGG_02548	63.75828227	20.83401897	0.326765688	-1.613671593	0.002441346	0.023444638	uncharacterized protein MGG_02548 [Pyricularia oryzae 70-15]
MGG_02562	1297.731455	3330.740875	2.566587149	1.359851248	0.000816162	0.010004781	initiation-specific alpha-1,6-mannosyltransferase
MGG_02564	1.66101151	12.4789827	7.512881533	2.909366353	0.0150947	0.085847571	uncharacterized protein MGG_02564 [Pyricularia oryzae 70-15]
MGG_02566	1613.06626	3856.105284	2.390543637	1.257338741	0.038493741	0.162481572	trans-2-enoyl-CoA reductase [Pyricularia oryzae 70-15]
MGG_02570	3061.70529	10746.68713	3.510033172	1.811484665	0.000954861	0.011361075	dienelactone hydrolase [Pyricularia oryzae 70-15]
MGG_02582	1137.600684	542.6116278	0.476978992	-1.068002369	0.009563546	0.06308753	uncharacterized protein MGG_02582 [Pyricularia oryzae 70-15]
MGG_02583	116.3914101	278.8740121	2.396001662	1.260628909	0.002988681	0.027195876	mitochondrial ATP-binding cassette sub-family B member 6
MGG_02590	0.321175942	5.136967181	15.99424648	3.999481121	0.048041873	0.187756029	uncharacterized protein MGG_02590 [Pyricularia oryzae 70-15]
MGG_02591	10.60026954	367.2625869	34.64653285	5.114639081	2.44972E-09	1.68131E-07	uncharacterized protein MGG_02591 [Pyricularia oryzae 70-15]
MGG_02592	144.5859843	867.3776641	5.99904388	2.584732584	1.44594E-09	1.06456E-07	uncharacterized protein MGG_02592 [Pyricularia oryzae 70-15]
MGG_02609	24.28454212	9.431939587	0.388392729	-1.364411902	0.042661502	0.174143792	uncharacterized protein MGG_02609 [Pyricularia oryzae 70-15]
MGG_02610	10398.50371	5070.480246	0.487616333	-1.036181645	0.009571149	0.06308753	uncharacterized protein MGG_02610 [Pyricularia oryzae 70-15]
MGG_02615	193.9518645	458.9439427	2.366277549	1.242619303	0.003204915	0.028460024	uncharacterized protein MGG_02615 [Pyricularia oryzae 70-15]
MGG_02616	2547.417031	5399.519005	2.119605443	1.083795738	0.007172596	0.051864697	isocitrate lyase [Pyricularia oryzae 70-15]
MGG_02619	2395.324191	10069.52511	4.203825583	2.071702813	0.00228892	0.022383658	hydroxyquinol 1,2-dioxygenase [Pyricularia oryzae 70-15]
MGG_02625	13794.90395	31953.21419	2.316305667	1.211825649	0.04935545	0.191617132	superoxide dismutase [Pyricularia oryzae 70-15]
MGG_02631	166.492992	49.47919919	0.297184876	-1.750567397	0.000133322	0.002360919	uncharacterized protein MGG_02631 [Pyricularia oryzae 70-15]
MGG_02637	7994.580335	18385.88222	2.299793291	1.201504195	0.008639348	0.058763046	uncharacterized protein MGG_02637 [Pyricularia oryzae 70-15]
MGG_02638	416.7004261	1590.622239	3.817184094	1.932508766	0.003189184	0.028361792	uncharacterized protein MGG_02638 [Pyricularia oryzae 70-15]
MGG_02648	3871.721904	10960.17441	2.830826874	1.50122352	0.000968148	0.011485408	interferon-induced GTP-binding protein Mx
MGG_02656	632.547565	299.691853	0.473785482	-1.077694103	0.009171523	0.061419876	protein kinase rad3 [Pyricularia oryzae 70-15]
MGG_02662	387.3008172	98.99570299	0.255604168	-1.968016735	7.23494E-06	0.000209761	uncharacterized protein MGG_02662 [Pyricularia oryzae 70-15]
MGG_02671	959.5933565	2622.100865	2.732512525	1.450228109	0.003835143	0.032602745	uncharacterized protein MGG_02671 [Pyricularia oryzae 70-15]
MGG_02692	108.6447596	39.99027197	0.368082843	-1.441897591	0.00232287	0.022606544	uncharacterized protein MGG_02692 [Pyricularia oryzae 70-15]
MGG_02695	1892.870463	3805.446937	2.010410649	1.007490218	0.013767352	0.080919812	cysteine proteinase 1 [Pyricularia oryzae 70-15]
MGG_02698	1388.425826	150.5870544	0.10845884	-3.20478045	1.46298E-08	8.71192E-07	uncharacterized protein MGG_02698 [Pyricularia oryzae 70-15]
MGG_02739	312.877264	1237.290411	3.954555199	1.983515432	0.000825616	0.010110469	uncharacterized protein MGG_02739 [Pyricularia oryzae 70-15]
MGG_02744	1386.222257	651.2721482	0.469817985	-1.089826152	0.006667341	0.049147367	uncharacterized protein MGG_02744 [Pyricularia oryzae 70-15]
MGG_02754	158.4556332	728.0176053	4.594457077	2.19989439	4.21145E-07	1.71106E-05	uncharacterized protein MGG_02754 [Pyricularia oryzae 70-15]
MGG_02758	289.2199416	788.5122097	2.726341086	1.446966066	0.00049303	0.006714488	uncharacterized protein MGG_02758 [Pyricularia oryzae 70-15]
MGG_02761	450.3958633	150.3379616	0.333790725	-1.582984229	0.000188057	0.003133772	pre-rRNA-processing protein IPI1 [Pyricularia oryzae 70-15]
MGG_02791	1.454922611	68.96124187	47.39856356	5.566771433	1.27173E-09	9.47787E-08	uncharacterized protein MGG_02791 [Pyricularia oryzae 70-15]
MGG_02792	646.408725	150.5712435	0.232935042	-2.102000404	5.61026E-06	0.000167865	cytochrome P450 52E1 [Pyricularia oryzae 70-15]
MGG_02793	1887.467946	615.1527106	0.325914256	-1.617435638	6.81813E-05	0.001348969	glycosyl hydrolase family 76 protein [Pyricularia oryzae 70-15]
MGG_02795	79.81402489	39.32008496	0.492646311	-1.02137584	0.038016774	0.161389625	RIP defective [Pyricularia oryzae 70-15]
MGG_02805	13446.81501	32371.39619	2.407365325	1.267455092	0.036757857	0.15756381	uncharacterized protein MGG_02805 [Pyricularia oryzae 70-15]
MGG_02810	409.4702676	203.1957501	0.496240548	-1.010888471	0.014023124	0.081900437	CAMK/CAMKL/GIN4 protein kinase [Pyricularia oryzae 70-15]
MGG_02812	945.8207802	2049.133296	2.166513296	1.115375091	0.035902958	0.155600833	1-aminocyclopropane-1-carboxylate deaminase
MGG_02814	2102.850696	4319.592095	2.054160147	1.038548662	0.042356067	0.17353845	uncharacterized protein MGG_02814 [Pyricularia oryzae 70-15]
MGG_02822	587.6472284	1305.701839	2.2219144	1.151803237	0.021483428	0.109840354	uncharacterized protein MGG_02822 [Pyricularia oryzae 70-15]
MGG_02839	170.0596595	64.77232088	0.380879987	-1.39259161	0.001798388	0.018656548	IBR domain-containing protein [Pyricularia oryzae 70-15]
MGG_02840	159.0696428	49.11454131	0.308761247	-1.695436404	0.000294389	0.004530539	uncharacterized protein MGG_02840 [Pyricularia oryzae 70-15]
MGG_02866	2388.531747	646.5165088	0.270675284	-1.885364938	5.06905E-06	0.000153181	uncharacterized protein MGG_02866 [Pyricularia oryzae 70-15]
MGG_02879	328.6973152	857.0224132	2.607330129	1.382573262	0.000881774	0.010637332	uncharacterized protein MGG_02879 [Pyricularia oryzae 70-15]
MGG_02880	2624.618588	1223.958396	0.466337624	-1.100553265	0.006608877	0.048983917	transcriptional activator xlnR [Pyricularia oryzae 70-15]

MGG_02881	541.4097416	196.8016028	0.363498452	-1.459978876	0.000492955	0.006714488	deoxyribose-phosphate aldolase 2 [Pyricularia oryzae 70-15]
MGG_02884	1642.945435	62.71640076	0.038173149	-4.71129799	1.95635E-23	1.58438E-20	beta-Ig-H3/Fasciclin [Pyricularia oryzae 70-15]
MGG_02889	1522.751952	691.8396864	0.454335117	-1.138171275	0.005214598	0.041134376	uncharacterized protein MGG_02889 [Pyricularia oryzae 70-15]
MGG_02899	498.8696056	1414.603798	2.83561833	1.503663361	0.000219994	0.003572846	proline-specific permease [Pyricularia oryzae 70-15]
MGG_02903	392.2566358	171.3172306	0.43674782	-1.195127592	0.005238503	0.041269346	carboxylesterase [Pyricularia oryzae 70-15]
MGG_02907	533.1007645	155.5484662	0.29178061	-1.777044081	4.64542E-05	0.000984862	uncharacterized protein MGG_02907 [Pyricularia oryzae 70-15]
MGG_02911	9852.461875	3860.634425	0.391844645	-1.351646314	0.003783285	0.032225832	xylosidase/arabinosidase [Pyricularia oryzae 70-15]
MGG_02916	2580.104875	1154.281618	0.44737779	-1.160434458	0.004764952	0.038479953	uncharacterized protein MGG_02916 [Pyricularia oryzae 70-15]
MGG_02919	168.6893563	345.1317791	2.045960615	1.032778373	0.016014009	0.089536193	uncharacterized protein MGG_02919 [Pyricularia oryzae 70-15]
MGG_02922	163.0253188	10291.91566	63.13078079	5.980271689	3.10471E-20	1.51672E-17	uncharacterized protein MGG_02922 [Pyricularia oryzae 70-15]
MGG_02929	715.0802994	158.3904285	0.221500199	-2.174620101	3.4276E-07	1.42598E-05	phosphotransferase enzyme family protein
MGG_02935	55.27967792	25.96479939	0.469698818	-1.090192131	0.044513609	0.179353674	uncharacterized protein MGG_02935 [Pyricularia oryzae 70-15]
MGG_02936	8.544573023	0.985681214	0.115357574	-3.115815366	0.016719752	0.092197706	uncharacterized protein MGG_02936 [Pyricularia oryzae 70-15]
MGG_02938	1291.606274	3325.438426	2.574653355	1.364378204	0.00098543	0.011633623	uncharacterized protein MGG_02938 [Pyricularia oryzae 70-15]
MGG_02966	909.5766222	198.0770391	0.217768393	-2.199133521	2.53529E-07	1.0694E-05	uncharacterized protein MGG_02966 [Pyricularia oryzae 70-15]
MGG_02970	1216.690473	2465.478213	2.026380799	1.018905312	0.012050867	0.074011088	ubiquitin carboxyl-terminal hydrolase 6 [Pyricularia oryzae 70-15]
MGG_02979	242.783455	622.8346474	2.565391647	1.359179093	0.001143104	0.013100398	uncharacterized protein MGG_02979 [Pyricularia oryzae 70-15]
MGG_02986	282.6689719	107.9439454	0.381874051	-1.388831205	0.001412931	0.015445603	DNA polymerase zeta catalytic subunit [Pyricularia oryzae 70-15]
MGG_02988	50.87519661	6.513690514	0.128032734	-2.965415385	6.44264E-06	0.000189504	uncharacterized protein MGG_02988 [Pyricularia oryzae 70-15]
MGG_02989	18.20332547	4.496087317	0.246992635	-2.017460074	0.012099403	0.074161392	uncharacterized protein MGG_02989 [Pyricularia oryzae 70-15]
MGG_03006	487.5442928	1020.234425	2.092598437	1.06529549	0.008864371	0.059924529	uncharacterized protein MGG_03006 [Pyricularia oryzae 70-15]
MGG_03009	84.92570029	40.98670005	0.482618335	-1.05104537	0.032488616	0.145869337	uncharacterized protein MGG_03009 [Pyricularia oryzae 70-15]
MGG_03012	209.9835855	95.92076907	0.456801273	-1.130361425	0.009862085	0.064759248	uncharacterized protein MGG_03012 [Pyricularia oryzae 70-15]
MGG_03016	83.41683585	39.33619998	0.471561881	-1.084480994	0.025846863	0.124739389	3-oxoacyl-[acyl-carrier-protein] reductase
MGG_03020	6.740755818	46.36323576	6.878047063	2.781998988	0.000463478	0.006411947	uncharacterized protein MGG_03020 [Pyricularia oryzae 70-15]
MGG_03037	419.1656538	71.89727998	0.17152474	-2.543511411	3.91895E-05	0.000857791	uncharacterized protein MGG_03037 [Pyricularia oryzae 70-15]
MGG_03045	3619.798625	10118.29212	2.795263815	1.48298445	0.027847968	0.131072108	ankyrin repeat protein [Pyricularia oryzae 70-15]
MGG_03054	522.6404173	253.1159939	0.484302372	-1.046020026	0.010389144	0.066953487	uncharacterized protein MGG_03054 [Pyricularia oryzae 70-15]
MGG_03055	168.4820139	47.04896025	0.279252124	-1.84035984	6.65094E-05	0.001323908	uncharacterized protein MGG_03055 [Pyricularia oryzae 70-15]
MGG_03059	5153.243178	2324.481458	0.451071564	-1.148571756	0.004409493	0.036218069	uncharacterized protein MGG_03059 [Pyricularia oryzae 70-15]
MGG_03070	1912.385702	807.2014671	0.422091352	-1.244372823	0.001901242	0.019507004	epoxide hydrolase domain-containing protein
MGG_03073	1622.699361	3517.062431	2.167414689	1.115975209	0.020549097	0.106319632	uncharacterized protein MGG_03073 [Pyricularia oryzae 70-15]
MGG_03078	75.34232142	23.9546424	0.317944045	-1.653155209	0.001350777	0.01490394	uncharacterized protein MGG_03078 [Pyricularia oryzae 70-15]
MGG_03085	104.2703449	219.6040613	2.106102762	1.074575831	0.014788402	0.084979766	uncharacterized protein MGG_03085 [Pyricularia oryzae 70-15]
MGG_03088	927.3895457	421.7430319	0.454763625	-1.136811231	0.004830244	0.038782422	uncharacterized protein MGG_03088 [Pyricularia oryzae 70-15]
MGG_03098	4937.975008	1359.285609	0.275271869	-1.861070912	0.004885779	0.039099109	thiazole biosynthetic enzyme [Pyricularia oryzae 70-15]
MGG_03107	2810.385643	1276.574629	0.454234682	-1.13849023	0.022750046	0.113901884	solute carrier family 35 member F1 [Pyricularia oryzae 70-15]
MGG_03115	105.2284067	294.8970008	2.802446697	1.486686933	0.000556966	0.007370399	uncharacterized protein MGG_03115 [Pyricularia oryzae 70-15]
MGG_03126	532.1563011	195.8209795	0.367976437	-1.442314708	0.000510857	0.006910797	2,4-dichlorophenol 6-monooxygenase [Pyricularia oryzae 70-15]
MGG_03127	601.0831977	181.380426	0.301755941	-1.72854592	0.000676828	0.008582579	uncharacterized protein MGG_03127 [Pyricularia oryzae 70-15]
MGG_03138	2067.097182	986.2812449	0.477133467	-1.067535212	0.011028447	0.069777901	uncharacterized protein MGG_03138 [Pyricularia oryzae 70-15]
MGG_03153	1222.011731	391.6060672	0.320460154	-1.641783113	6.48516E-05	0.001298927	uncharacterized protein MGG_03153 [Pyricularia oryzae 70-15]
MGG_03159	746.6610842	319.4605604	0.427852164	-1.224815709	0.003485205	0.030148975	YdiU domain-containing protein [Pyricularia oryzae 70-15]
MGG_03161	427.6412483	148.9272712	0.348252821	-1.521793055	0.000340847	0.005049529	uncharacterized protein MGG_03161 [Pyricularia oryzae 70-15]
MGG_03163	1096.50674	3480.549735	3.174216453	1.66640051	0.025012599	0.121541222	uncharacterized protein MGG_03163 [Pyricularia oryzae 70-15]
MGG_03168	125.2375283	40.87232075	0.326358411	-1.615470873	0.000996754	0.011733107	uncharacterized protein MGG_03168 [Pyricularia oryzae 70-15]
MGG_03176	82.43938518	14.29472601	0.173396805	-2.527850778	6.38861E-05	0.001289182	uncharacterized protein MGG_03176 [Pyricularia oryzae 70-15]
MGG_03177	5.282199743	0.34480135	0.065276091	-3.93730153	0.039687324	0.165620617	uncharacterized protein MGG_03177 [Pyricularia oryzae 70-15]

MGG_03198	4322.910173	1735.605976	0.401490178	-1.3165634	0.001120501	0.012926725	uncharacterized protein MGG_03198 [Pyricularia oryzae 70-15]
MGG_03208	438.8751776	1494.193323	3.404597478	1.76748424	1.13477E-05	0.000305658	glycolipid-anchored surface protein 5 [Pyricularia oryzae 70-15]
MGG_03213	51.17789973	3.559128937	0.069544256	-3.845924839	2.57167E-08	1.42651E-06	uncharacterized protein MGG_03213 [Pyricularia oryzae 70-15]
MGG_03231	46.81017297	12.90433291	0.275673686	-1.858966533	0.001298099	0.014467249	pentachlorophenol 4-monooxygenase [Pyricularia oryzae 70-15]
MGG_03238	1997.616785	4281.178142	2.143142856	1.099728019	0.0060336	0.045867442	zinc finger protein ZPR1 [Pyricularia oryzae 70-15]
MGG_03239	1668.79961	500.4000776	0.299856301	-1.737656807	2.21119E-05	0.000527732	peptidyl-prolyl cis-trans isomerase-like 3 [Pyricularia oryzae 70-15]
MGG_03259	141.4251442	51.51669572	0.364268292	-1.456926675	0.012279776	0.074736835	uncharacterized protein MGG_03259 [Pyricularia oryzae 70-15]
MGG_03272	3112.861503	19374.69975	6.224080233	2.637860658	3.17192E-10	2.77212E-08	uncharacterized protein MGG_03272 [Pyricularia oryzae 70-15]
MGG_03279	2280.187285	300.5961097	0.13182957	-2.923254083	1.35989E-11	1.54391E-09	uncharacterized protein MGG_03279 [Pyricularia oryzae 70-15]
MGG_03280	5142.311926	2414.196397	0.469476848	-1.09087408	0.005959202	0.045441286	nitrilase 2 [Pyricularia oryzae 70-15]
MGG_03295	1469.510287	539.4699988	0.367108692	-1.445720822	0.000654322	0.008384711	uncharacterized protein MGG_03295 [Pyricularia oryzae 70-15]
MGG_03298	17538.59667	2997.172001	0.170890069	-2.548859533	8.21961E-10	6.50396E-08	uncharacterized protein MGG_03298 [Pyricularia oryzae 70-15]
MGG_03308	316.242265	98.12266398	0.310276882	-1.688371885	0.000674203	0.008567172	uncharacterized protein MGG_03308 [Pyricularia oryzae 70-15]
MGG_03310	1824.26942	3830.835464	2.099928564	1.07034025	0.008139508	0.056213046	T-complex protein 1 subunit eta [Pyricularia oryzae 70-15]
MGG_03312	53.77835388	12.73878552	0.236875706	-2.077797854	0.00041783	0.005895846	uncharacterized protein MGG_03312 [Pyricularia oryzae 70-15]
MGG_03319	4359.928511	2062.527676	0.473064563	-1.079891001	0.007693987	0.054341017	uncharacterized protein MGG_03319 [Pyricularia oryzae 70-15]
MGG_03327	51831.91817	10380.09903	0.200264613	-2.320020577	0.000215713	0.003517425	uncharacterized protein MGG_03327 [Pyricularia oryzae 70-15]
MGG_03332	2593.449974	1079.373767	0.416192245	-1.264678012	0.001539661	0.016508207	copper amine oxidase 1 [Pyricularia oryzae 70-15]
MGG_03335	1299.220855	4036.609459	3.106946324	1.635497318	0.000572504	0.007494427	methylglutaconyl-CoA hydratase [Pyricularia oryzae 70-15]
MGG_03339	11.58669258	300.992683	25.9774462	4.699187704	2.40537E-06	7.98373E-05	uncharacterized protein MGG_03339 [Pyricularia oryzae 70-15]
MGG_03348	137.6796231	54.77348577	0.397832915	-1.329765452	0.004074258	0.034086836	phosphate-repressible phosphate permease
MGG_03352	291.2900323	115.0919145	0.395111064	-1.339669851	0.014918092	0.085362685	3-hydroxyphenylacetate 6 hydroxylase [Pyricularia oryzae 70-15]
MGG_03353	338.1332246	997.2762658	2.94935899	1.560401435	0.005665933	0.043756998	uncharacterized protein MGG_03353 [Pyricularia oryzae 70-15]
MGG_03356	1367.175086	550.3019188	0.402510201	-1.312902748	0.001056879	0.012308432	ricin B lectin:Parallel beta-helix [Pyricularia oryzae 70-15]
MGG_03360	1319.519767	4381.383552	3.320437981	1.731373552	2.71457E-05	0.000630527	carboxylic acid transporter [Pyricularia oryzae 70-15]
MGG_03364	283.8809046	73.60601657	0.259284846	-1.947390206	0.00010263	0.00192399	uncharacterized protein MGG_03364 [Pyricularia oryzae 70-15]
MGG_03365	139.3009847	29.18905525	0.209539475	-2.254706034	3.18275E-06	0.000100688	uncharacterized protein MGG_03365 [Pyricularia oryzae 70-15]
MGG_03367	121.5525414	605.0749391	4.977888015	2.315533775	7.59877E-08	3.67769E-06	uncharacterized protein MGG_03367 [Pyricularia oryzae 70-15]
MGG_03379	274.3098449	110.6547456	0.403393271	-1.309741077	0.002298725	0.022429644	uncharacterized protein MGG_03379 [Pyricularia oryzae 70-15]
MGG_03383	147.937444	553.2279874	3.739607583	1.902886888	9.16405E-06	0.000257697	uncharacterized protein MGG_03383 [Pyricularia oryzae 70-15]
MGG_03384	761.7670366	332.445195	0.436413206	-1.196233335	0.003147861	0.028200753	uncharacterized protein MGG_03384 [Pyricularia oryzae 70-15]
MGG_03395	150.1824941	17.33105907	0.115399995	-3.115284933	1.12096E-09	8.40581E-08	uncharacterized protein MGG_03395 [Pyricularia oryzae 70-15]
MGG_03403	11.33481326	91.40662539	8.064237432	3.011538116	0.000231904	0.003736297	uncharacterized protein MGG_03403 [Pyricularia oryzae 70-15]
MGG_03407	1037.502967	425.3756045	0.40999941	-1.28630626	0.046618919	0.184403559	uncharacterized protein MGG_03407 [Pyricularia oryzae 70-15]
MGG_03414	1629.881732	536.5040514	0.329167473	-1.603106312	8.6899E-05	0.001667693	short chain dehydrogenase/reductase family protein
MGG_03415	552.8369949	223.1346851	0.403617499	-1.308939372	0.034239142	0.150374947	uncharacterized protein MGG_03415 [Pyricularia oryzae 70-15]
MGG_03425	5.240927269	27.87298743	5.31833128	2.410973646	0.000871979	0.0105401	uncharacterized protein MGG_03425 [Pyricularia oryzae 70-15]
MGG_03433	25.22486923	5.060159829	0.200602024	-2.317591931	0.001882555	0.019347951	averantin oxidoreductase [Pyricularia oryzae 70-15]
MGG_03442	700.9208902	190.9024815	0.272359526	-1.876415767	1.04767E-05	0.000286001	uncharacterized protein MGG_03442 [Pyricularia oryzae 70-15]
MGG_03443	5.82332391	0	0	-Inf	0.007826021	0.054890588	uncharacterized protein MGG_03443 [Pyricularia oryzae 70-15]
MGG_03454	75.22054642	31.58206319	0.419859529	-1.252021364	0.014795209	0.084979766	uncharacterized protein MGG_03454 [Pyricularia oryzae 70-15]
MGG_03462	4282.606495	1579.964121	0.368925822	-1.438597326	0.000346669	0.005110847	uncharacterized protein MGG_03462 [Pyricularia oryzae 70-15]
MGG_03463	2548.388481	1038.6307	0.407563724	-1.294902447	0.001204026	0.013656001	uncharacterized protein MGG_03463 [Pyricularia oryzae 70-15]
MGG_03464	1074.18053	2538.614993	2.363303859	1.240805134	0.009308132	0.061959006	uncharacterized protein MGG_03464 [Pyricularia oryzae 70-15]
MGG_03465	617.672403	113.6973758	0.184073912	-2.441642917	1.61326E-08	9.46759E-07	uncharacterized protein MGG_03465 [Pyricularia oryzae 70-15]
MGG_03468	61.26476376	10.63689196	0.173621692	-2.525980885	1.45822E-05	0.000373723	intracellular hyphae protein 1 [Pyricularia oryzae 70-15]
MGG_03474	385.4868508	139.1047303	0.36085467	-1.47051017	0.01195014	0.073584379	uncharacterized protein MGG_03474 [Pyricularia oryzae 70-15]
MGG_03488	209.6285504	462.1906738	2.204807851	1.140652931	0.006557668	0.048663747	CDC7 protein kinase [Pyricularia oryzae 70-15]

MGG_03495	48.14668057	113.1597874	2.350313378	1.232853131	0.009424475	0.062527867	uncharacterized protein MGG_03495 [Pyricularia oryzae 70-15]
MGG_03496	31.28621313	74.9827399	2.396670367	1.261031497	0.014069491	0.082131755	uncharacterized protein MGG_03496 [Pyricularia oryzae 70-15]
MGG_03497	203.4818805	96.18601367	0.472700633	-1.081001299	0.014791704	0.084979766	uncharacterized protein MGG_03497 [Pyricularia oryzae 70-15]
MGG_03498	412.2229197	175.4185343	0.425542894	-1.232623534	0.003055482	0.027638124	uncharacterized protein MGG_03498 [Pyricularia oryzae 70-15]
MGG_03505	477.5939156	174.2747519	0.364901533	-1.454420883	0.000529576	0.007105508	uncharacterized protein MGG_03505 [Pyricularia oryzae 70-15]
MGG_03519	214.9445143	29.67380153	0.138053309	-2.856702622	1.87594E-09	1.33254E-07	uncharacterized protein MGG_03519 [Pyricularia oryzae 70-15]
MGG_03525	1993.962329	4010.792564	2.011468575	1.008249199	0.012294147	0.074786832	uncharacterized protein MGG_03525 [Pyricularia oryzae 70-15]
MGG_03526	1246.117725	24.9253944	0.02000244	-5.643680227	2.0478E-29	3.55381E-26	uncharacterized protein MGG_03526 [Pyricularia oryzae 70-15]
MGG_03529	2658.938551	754.50263	0.283760838	-1.817252601	0.032429849	0.14585628	uncharacterized protein MGG_03529 [Pyricularia oryzae 70-15]
MGG_03557	438.2779607	128.7498799	0.293763071	-1.767275047	0.007334088	0.052594153	uncharacterized protein MGG_03557 [Pyricularia oryzae 70-15]
MGG_03567	95.35932092	220.2791125	2.309990365	1.207886834	0.005294757	0.041503884	uncharacterized protein MGG_03567 [Pyricularia oryzae 70-15]
MGG_03568	2620.611329	1130.854499	0.431523167	-1.212490082	0.002868869	0.02643158	vacuole morphology and inheritance protein 14
MGG_03575	146.154015	63.32759572	0.433293575	-1.20658325	0.008129486	0.056175765	uncharacterized protein MGG_03575 [Pyricularia oryzae 70-15]
MGG_03593	7236.593365	25188.42043	3.480701368	1.799378041	1.15729E-05	0.000311033	uncharacterized protein MGG_03593 [Pyricularia oryzae 70-15]
MGG_03594	245.219869	613.9469351	2.503659013	1.324038087	0.00114961	0.013149034	uncharacterized protein MGG_03594 [Pyricularia oryzae 70-15]
MGG_03604	983.5197902	2069.291232	2.103965017	1.073110717	0.049137979	0.190955908	nuclear movement protein nudC [Pyricularia oryzae 70-15]
MGG_03620	6620.837348	2669.207414	0.403152543	-1.310602272	0.015262501	0.08655876	glucose transporter rco-3 [Pyricularia oryzae 70-15]
MGG_03627	1437.956819	602.9725962	0.419325941	-1.253856012	0.025294291	0.122561495	uncharacterized protein MGG_03627 [Pyricularia oryzae 70-15]
MGG_03629	835.960919	343.9973317	0.411499298	-1.281038124	0.001861168	0.019176815	uncharacterized protein MGG_03629 [Pyricularia oryzae 70-15]
MGG_03635	374.5786472	934.1696714	2.493921313	1.318415946	0.011065713	0.069905385	uncharacterized protein MGG_03635 [Pyricularia oryzae 70-15]
MGG_03662	1326.729011	4885.09883	3.682062268	1.880514024	5.16872E-06	0.000155793	phosphoadenosine phosphosulfate reductase
MGG_03669	2518.427334	1235.47872	0.490575489	-1.027452942	0.01023866	0.06637099	uncharacterized protein MGG_03669 [Pyricularia oryzae 70-15]
MGG_03671	151.8475716	346.3450961	2.280873461	1.18958641	0.012753969	0.077043864	uncharacterized protein MGG_03671 [Pyricularia oryzae 70-15]
MGG_03682	836.9973575	417.6293179	0.498961334	-1.003000074	0.012353157	0.075108185	uncharacterized protein MGG_03682 [Pyricularia oryzae 70-15]
MGG_03685	315.9737647	40.35993079	0.127731905	-2.968809171	0.036207766	0.156031194	uncharacterized protein MGG_03685 [Pyricularia oryzae 70-15]
MGG_03692	393.0649559	186.3702386	0.474146158	-1.07659625	0.00854568	0.058354648	glutaminyl-peptide cyclotransferase [Pyricularia oryzae 70-15]
MGG_03701	4293.265464	1157.272661	0.269555347	-1.891346568	9.8853E-05	0.001867599	uncharacterized protein MGG_03701 [Pyricularia oryzae 70-15]
MGG_03709	168.2207932	72.9110089	0.433424475	-1.206147473	0.006964036	0.050804353	uncharacterized protein MGG_03709 [Pyricularia oryzae 70-15]
MGG_03711	183.0604221	87.92216269	0.480290396	-1.058021133	0.017030752	0.093530548	uncharacterized protein MGG_03711 [Pyricularia oryzae 70-15]
MGG_03715	1159.310828	283.4400583	0.244490133	-2.032151853	0.009339169	0.062109985	uncharacterized protein MGG_03715 [Pyricularia oryzae 70-15]
MGG_03722	43.92804639	14.75016515	0.33578013	-1.574411232	0.005994191	0.045653566	uncharacterized protein MGG_03722 [Pyricularia oryzae 70-15]
MGG_03725	2246.447382	5482.816874	2.44066116	1.287272018	0.005077339	0.040182097	COP9 signalosome complex subunit 7a [Pyricularia oryzae 70-15]
MGG_03728	682.5714763	1506.29274	2.206791219	1.141950145	0.004878227	0.039064401	ubiquitin-conjugating enzyme E2-20 kDa [Pyricularia oryzae 70-15]
MGG_03736	5882.168898	2293.503305	0.389907761	-1.358795223	0.000666001	0.008489597	multidrug resistance-associated protein 1
MGG_03743	944.6966933	387.0670871	0.409726307	-1.28726757	0.008007756	0.055724809	IQ calmodulin-binding domain-containing protein
MGG_03754	960.5389463	476.0774872	0.495635798	-1.012647703	0.013178108	0.078805168	uncharacterized protein MGG_03754 [Pyricularia oryzae 70-15]
MGG_03757	585.0720615	1429.862802	2.443908872	1.289190491	0.001724682	0.018046026	uncharacterized protein MGG_03757 [Pyricularia oryzae 70-15]
MGG_03758	487.8944415	1164.221353	2.386215653	1.254724432	0.002137859	0.02120058	uncharacterized protein MGG_03758 [Pyricularia oryzae 70-15]
MGG_03760	1.808335146	14.07919635	7.785722897	2.960830998	0.003113672	0.027997692	uncharacterized protein MGG_03760 [Pyricularia oryzae 70-15]
MGG_03764	4104.483284	546.5193907	0.133151813	-2.908856022	1.58484E-11	1.78266E-09	salicylate hydroxylase [Pyricularia oryzae 70-15]
MGG_03772	13.20191367	31.28176552	2.369487205	1.24457487	0.049928794	0.193249742	cholinesterase [Pyricularia oryzae 70-15]
MGG_03791	0	6.972089369	Inf	Inf	0.045329441	0.181497051	uncharacterized protein MGG_03791 [Pyricularia oryzae 70-15]
MGG_03793	699.3906072	119.5712154	0.170964857	-2.548228292	5.63762E-09	3.66234E-07	2,3-dihydroxybenzoic acid decarboxylase
MGG_03800	384.8591848	917.0376687	2.382787536	1.252650318	0.001980153	0.020095993	uncharacterized protein MGG_03800 [Pyricularia oryzae 70-15]
MGG_03810	10.78346323	65.61483011	6.084764115	2.605201336	0.000235094	0.003782675	polyketide synthase [Pyricularia oryzae 70-15]
MGG_03811	10.49000594	59.25219862	5.648442808	2.497853193	2.31593E-05	0.00054629	uncharacterized protein MGG_03811 [Pyricularia oryzae 70-15]
MGG_03813	0.339553209	24.22634109	71.34770182	6.156795055	2.09113E-07	8.95872E-06	uncharacterized protein MGG_03813 [Pyricularia oryzae 70-15]
MGG_03825	0.642351884	16.46720335	25.63579831	4.680087919	7.5788E-05	0.001480126	isotrichodermin C-15 hydroxylase [Pyricularia oryzae 70-15]

MGG_03829	1.624256977	11.62505524	7.157152721	2.839385765	0.012069361	0.074049795	uncharacterized protein MGG_03829 [Pyricularia oryzae 70-15]
MGG_03831	41.07391195	114.1740308	2.779721371	1.47494028	0.00220555	0.021721662	uncharacterized protein MGG_03831 [Pyricularia oryzae 70-15]
MGG_03851	322.4998536	670.0686249	2.077733114	1.055010351	0.016187132	0.090209383	uncharacterized protein MGG_03851 [Pyricularia oryzae 70-15]
MGG_03857	1980.914643	5219.506969	2.634897464	1.397746821	0.001425982	0.015550117	T-complex protein 1 subunit zeta [Pyricularia oryzae 70-15]
MGG_03865	2516.383117	9345.616244	3.713908339	1.892938209	0.001007604	0.01181503	uncharacterized protein MGG_03865 [Pyricularia oryzae 70-15]
MGG_03870	1639.410768	642.4150911	0.391857308	-1.35159969	0.002293457	0.022396233	minor extracellular protease vpr [Pyricularia oryzae 70-15]
MGG_03881	399.0424058	133.3928807	0.334282469	-1.580860398	0.000203226	0.003340724	tRNA (uracil-5-)-methyltransferase [Pyricularia oryzae 70-15]
MGG_03883	4178.939379	8622.378563	2.063293525	1.044949074	0.010563522	0.067682314	alpha-1,2-mannosyltransferase alg-11 [Pyricularia oryzae 70-15]
MGG_03888	389.4197969	168.6266279	0.433020173	-1.207493857	0.004510572	0.036873774	uncharacterized protein MGG_03888 [Pyricularia oryzae 70-15]
MGG_03904	27.13412659	7.163276015	0.263995083	-1.921417033	0.006807443	0.049884092	aquaporin [Pyricularia oryzae 70-15]
MGG_03912	1245.782887	555.2907236	0.445736355	-1.16573746	0.004460388	0.03661135	uncharacterized protein MGG_03912 [Pyricularia oryzae 70-15]
MGG_03915	1465.051432	705.5237003	0.481569237	-1.05418486	0.009211934	0.061523444	pyridoxamine phosphate oxidase [Pyricularia oryzae 70-15]
MGG_03920	1420.019348	2994.792824	2.108980296	1.076545616	0.007513059	0.053561407	inositolphosphorylceramide-B C-26 hydroxylase
MGG_03933	225.6563009	89.71676375	0.39758147	-1.330677577	0.002659798	0.024989351	phosphatidylinositol N-acetylglucosaminyltransferase GPI3 subunit
MGG_03939	491.2157385	182.4727099	0.371471628	-1.428676071	0.000647456	0.008305486	pathway-specific nitrogen regulator [Pyricularia oryzae 70-15]
MGG_03963	62.05905278	11.37273543	0.183256671	-2.448062376	1.51289E-05	0.000383687	uncharacterized protein MGG_03963 [Pyricularia oryzae 70-15]
MGG_03966	193.7090846	74.52219993	0.384711951	-1.378149446	0.001613519	0.017117226	uncharacterized protein MGG_03966 [Pyricularia oryzae 70-15]
MGG_03967	384.1718253	156.5251114	0.407435166	-1.295357589	0.026020491	0.1251869	uncharacterized protein MGG_03967 [Pyricularia oryzae 70-15]
MGG_03988	89.1275162	14.67505807	0.164652385	-2.602504689	1.08056E-06	4.03899E-05	uncharacterized protein MGG_03988 [Pyricularia oryzae 70-15]
MGG_04003	383.3298052	184.4753163	0.481244385	-1.055158387	0.013668173	0.080602413	uncharacterized protein MGG_04003 [Pyricularia oryzae 70-15]
MGG_04014	2317.052432	5137.122362	2.217093706	1.148669747	0.004707421	0.03814927	dihydroxyacetone kinase [Pyricularia oryzae 70-15]
MGG_04017	1830.225483	259.6598162	0.141873129	-2.817326728	3.85685E-11	4.07417E-09	L-asparaginase [Pyricularia oryzae 70-15]
MGG_04018	77.80225428	30.30927642	0.389568101	-1.360052546	0.007586693	0.053959689	mitochondrial chaperone BCS1 [Pyricularia oryzae 70-15]
MGG_04019	539.5709515	235.9115675	0.43722066	-1.193566521	0.003904807	0.032987205	transcription factor tau subunit sfc1 [Pyricularia oryzae 70-15]
MGG_04024	1105.226648	478.9180946	0.433321161	-1.206491402	0.00298049	0.027182429	uncharacterized protein MGG_04024 [Pyricularia oryzae 70-15]
MGG_04026	282.4187471	604.2395921	2.139516581	1.097284859	0.009207549	0.061523444	uncharacterized protein MGG_04026 [Pyricularia oryzae 70-15]
MGG_04030	5194.269844	2090.135811	0.402392612	-1.313324277	0.00105164	0.012260381	uncharacterized protein MGG_04030 [Pyricularia oryzae 70-15]
MGG_04034	16445.94665	6192.309689	0.376524977	-1.409182523	0.000372991	0.005387752	formate dehydrogenase [Pyricularia oryzae 70-15]
MGG_04036	283.1242043	105.1170551	0.37127541	-1.429438326	0.012652266	0.076611634	methyltransferase domain-containing protein
MGG_04039	49.99479271	22.12074284	0.442460937	-1.176378003	0.028995314	0.134594983	oxidoreductase [Pyricularia oryzae 70-15]
MGG_04040	316.6557918	89.43023575	0.28242097	-1.824080883	3.5714E-05	0.000796062	DEAH box polypeptide 37 [Pyricularia oryzae 70-15]
MGG_04070	272.5580227	119.7105354	0.439211197	-1.18701326	0.004856294	0.038914416	isoform CRA_b [Pyricularia oryzae 70-15]
MGG_04074	297.1116634	134.2576181	0.45187596	-1.146001289	0.00641319	0.048120712	uncharacterized protein MGG_04074 [Pyricularia oryzae 70-15]
MGG_04094	5.735955518	18.65287281	3.251920757	1.701292102	0.031034902	0.141202992	uncharacterized protein MGG_04094 [Pyricularia oryzae 70-15]
MGG_04108	3724.341772	1620.488388	0.435107326	-1.200556788	0.002983523	0.027189671	transcriptional activator protein acu-15 [Pyricularia oryzae 70-15]
MGG_04109	1379.085513	332.9838649	0.24145266	-2.05018774	0.039554159	0.165235185	uncharacterized protein MGG_04109 [Pyricularia oryzae 70-15]
MGG_04110	939.761065	2080.579115	2.213944791	1.146619246	0.004716327	0.03819596	queuine tRNA-ribosyltransferase [Pyricularia oryzae 70-15]
MGG_04120	671.7414779	293.5563429	0.437007915	-1.194268684	0.003429074	0.029786208	uncharacterized protein MGG_04120 [Pyricularia oryzae 70-15]
MGG_04121	85.3432805	31.18180097	0.36536914	-1.452573311	0.002966313	0.027093312	uncharacterized protein MGG_04121 [Pyricularia oryzae 70-15]
MGG_04141	590.6196508	186.0301648	0.31497456	-1.666692785	7.16575E-05	0.001406657	uncharacterized protein MGG_04141 [Pyricularia oryzae 70-15]
MGG_04145	422.5085228	873.58988	2.067626646	1.0479757	0.011049927	0.069877412	chitin synthase 2 [Pyricularia oryzae 70-15]
MGG_04147	1894.302352	3908.837469	2.063470737	1.045072979	0.038307338	0.162032571	uncharacterized protein MGG_04147 [Pyricularia oryzae 70-15]
MGG_04159	3.707672147	31.41922839	8.474111825	3.083062167	0.000976174	0.011558056	calcium-proton exchanger [Pyricularia oryzae 70-15]
MGG_04161	12.90845638	39.16565166	3.034108069	1.601272472	0.0088929	0.060083952	cation efflux system protein czcD [Pyricularia oryzae 70-15]
MGG_04172	1453.584305	403.1480284	0.277347538	-1.850233176	7.8317E-06	0.000224386	glycosyl hydrolase [Pyricularia oryzae 70-15]
MGG_04182	858.3948541	414.8595151	0.483296834	-1.049018551	0.009079224	0.06103731	multidrug and toxin extrusion protein 1 [Pyricularia oryzae 70-15]
MGG_04202	35.61334195	6.712323359	0.188477772	-2.407533702	0.000285196	0.004419086	MAS3 protein [Pyricularia oryzae 70-15]
MGG_04203	5443.169264	1794.723968	0.329720404	-1.600684928	7.1676E-05	0.001406657	4-coumarate-CoA ligase [Pyricularia oryzae 70-15]

MGG_04211	394.8248278	111.1335154	0.281475499	-1.828918747	0.031746957	0.143582293	uncharacterized protein MGG_04211 [Pyricularia oryzae 70-15]
MGG_04212	4967.38663	2478.79882	0.499014674	-1.002845855	0.012251326	0.074622274	L-ornithine 5-monooxygenase (L-ornithine N(5)-oxygenase)
MGG_04225	20401.01806	7397.406417	0.362599866	-1.463549703	0.008933519	0.060224412	MFS quinate transporter [Pyricularia oryzae 70-15]
MGG_04234	41117.19139	9219.081006	0.224214755	-2.157046872	1.58206E-07	7.09185E-06	hexose transporter [Pyricularia oryzae 70-15]
MGG_04235	3497.100036	1181.93692	0.337976297	-1.565006025	0.000119062	0.002163874	uncharacterized protein MGG_04235 [Pyricularia oryzae 70-15]
MGG_04241	3138.510303	1288.516017	0.410550195	-1.284369474	0.001441045	0.015602334	uncharacterized protein MGG_04241 [Pyricularia oryzae 70-15]
MGG_04243	673.8976834	2287.149239	3.39391171	1.762949035	0.000138521	0.00243524	peptide methionine sulfoxide [Pyricularia oryzae 70-15]
MGG_04244	2342.939925	714.574662	0.304990604	-1.713163299	0.028488394	0.132915008	uncharacterized protein MGG_04244 [Pyricularia oryzae 70-15]
MGG_04246	349.2319968	1009.684323	2.891156401	1.531646656	0.023688142	0.117086588	uncharacterized protein MGG_04246 [Pyricularia oryzae 70-15]
MGG_04247	1574.08609	6013.287458	3.820176988	1.93363948	1.91754E-06	6.67457E-05	uncharacterized protein MGG_04247 [Pyricularia oryzae 70-15]
MGG_04248	1.776098554	17.30545662	9.743522726	3.284443466	0.00063862	0.008226884	uncharacterized protein MGG_04248 [Pyricularia oryzae 70-15]
MGG_04257	106.2208747	250.9410773	2.36244597	1.240281335	0.004566129	0.037202776	uncharacterized protein MGG_04257 [Pyricularia oryzae 70-15]
MGG_04263	31.96562505	100.8343826	3.154463036	1.657394445	0.000884601	0.010660851	uncharacterized protein MGG_04263 [Pyricularia oryzae 70-15]
MGG_04271	11.29235081	36.39483053	3.22296315	1.688387693	0.008161008	0.056329505	uncharacterized protein MGG_04271 [Pyricularia oryzae 70-15]
MGG_04272	279.5213933	695.9359216	2.489741173	1.315995771	0.028383546	0.132718752	uncharacterized protein MGG_04272 [Pyricularia oryzae 70-15]
MGG_04296	705.4431311	1594.178995	2.259826377	1.176211934	0.004965611	0.039520536	uncharacterized protein MGG_04296 [Pyricularia oryzae 70-15]
MGG_04305	22.59913992	6.885353752	0.304673265	-1.714665184	0.027843005	0.131072108	cell wall glycosyl hydrolase YteR [Pyricularia oryzae 70-15]
MGG_04318	139.6477413	45.00793253	0.322296173	-1.633541039	0.000479904	0.006602445	uncharacterized protein MGG_04318 [Pyricularia oryzae 70-15]
MGG_04319	169.2733119	56.9653788	0.336529002	-1.571197252	0.00049196	0.006714488	uncharacterized protein MGG_04319 [Pyricularia oryzae 70-15]
MGG_04339	148.5619341	972.0442417	6.543023606	2.709957476	3.99907E-10	3.35039E-08	uncharacterized protein MGG_04339 [Pyricularia oryzae 70-15]
MGG_04340	800.7788061	4516.903599	5.640638294	2.495858427	3.93266E-08	2.0823E-06	uncharacterized protein MGG_04340 [Pyricularia oryzae 70-15]
MGG_04341	48.91403986	204.1818248	4.174298941	2.061533922	7.76621E-06	0.000223035	uncharacterized protein MGG_04341 [Pyricularia oryzae 70-15]
MGG_04345	1809.727707	28962.39584	16.00373124	4.0003364	4.36209E-16	1.17757E-13	cytochrome P450 17A1 [Pyricularia oryzae 70-15]
MGG_04348	34.85562956	119.0443002	3.415353611	1.772034956	0.000288183	0.004459677	pectate lyase [Pyricularia oryzae 70-15]
MGG_04360	844.8549142	192.1443179	0.227428775	-2.136513297	2.77552E-06	9.05735E-05	uncharacterized protein MGG_04360 [Pyricularia oryzae 70-15]
MGG_04361	628.0925963	227.9066902	0.362855241	-1.462533988	0.00052993	0.007105508	uncharacterized protein MGG_04361 [Pyricularia oryzae 70-15]
MGG_04362	5372.021822	1852.53164	0.344848123	-1.535966982	0.045403158	0.18167245	uncharacterized protein MGG_04362 [Pyricularia oryzae 70-15]
MGG_04368	2018.227814	853.8633633	0.423075808	-1.241011901	0.002019205	0.020407077	stage V sporulation protein K [Pyricularia oryzae 70-15]
MGG_04369	1020.515029	410.4042576	0.402154055	-1.314179826	0.001292775	0.014447685	spastin [Pyricularia oryzae 70-15]
MGG_04370	1249.047933	342.0635201	0.273859402	-1.868492684	7.20875E-06	0.000209502	uncharacterized protein MGG_04370 [Pyricularia oryzae 70-15]
MGG_04379	963.7233314	385.151742	0.399649702	-1.323192082	0.038137835	0.161596936	uncharacterized protein MGG_04379 [Pyricularia oryzae 70-15]
MGG_04393	2169.874375	13388.50355	6.170174506	2.625311293	5.29686E-10	4.31854E-08	uncharacterized protein MGG_04393 [Pyricularia oryzae 70-15]
MGG_04396	4545.788455	9652.198057	2.123327593	1.086326971	0.007050536	0.051180845	valyl-tRNA synthetase [Pyricularia oryzae 70-15]
MGG_04406	1023.188859	326.3452262	0.318949159	-1.648601619	5.86331E-05	0.001207245	uncharacterized protein MGG_04406 [Pyricularia oryzae 70-15]
MGG_04421	772.8783705	256.600567	0.332006402	-1.590717034	0.000134921	0.002378834	uncharacterized protein MGG_04421 [Pyricularia oryzae 70-15]
MGG_04425	2441.23902	5810.629381	2.380196832	1.251080883	0.00202839	0.020460307	phenylalanyl-tRNA synthetase subunit alpha
MGG_04428	16648.29287	7027.95952	0.422142953	-1.244196463	0.001954733	0.019921218	zinc finger transcription factor ace1 [Pyricularia oryzae 70-15]
MGG_04433	1960.51701	914.4898813	0.466453429	-1.100195047	0.00681662	0.049884517	sulfate transporter [Pyricularia oryzae 70-15]
MGG_04447	1071.797943	230.2404756	0.214817053	-2.218819569	1.80845E-07	7.95979E-06	uncharacterized protein MGG_04447 [Pyricularia oryzae 70-15]
MGG_04458	536.2348974	1502.125589	2.801245492	1.486068422	0.000278849	0.004342892	thymidylate synthase [Pyricularia oryzae 70-15]
MGG_04461	36.81037183	12.8168155	0.348184896	-1.522074472	0.023341127	0.115963817	uncharacterized protein MGG_04461 [Pyricularia oryzae 70-15]
MGG_04464	5071.007907	15039.86141	2.965852487	1.568446844	0.000117223	0.002140282	alpha-mannosidase [Pyricularia oryzae 70-15]
MGG_04465	1207.491198	5867.526653	4.859270744	2.280739817	0.008522712	0.058263311	uncharacterized protein MGG_04465 [Pyricularia oryzae 70-15]
MGG_04469	3364.650703	1166.034713	0.346554462	-1.528846002	0.006643805	0.04910664	cytochrome P450 97B3 [Pyricularia oryzae 70-15]
MGG_04475	87.08744797	24.46999207	0.280981848	-1.831451162	0.000370269	0.005376242	AGC/NDR protein kinase [Pyricularia oryzae 70-15]
MGG_04490	241.5989989	65.17947803	0.269783726	-1.890124773	2.08695E-05	0.000502024	uncharacterized protein MGG_04490 [Pyricularia oryzae 70-15]
MGG_04495	4744.615509	11851.19064	2.497818974	1.320668924	0.00103215	0.012079742	phosphoglucomutase [Pyricularia oryzae 70-15]
MGG_04504	1391.904608	3817.761035	2.742832385	1.455666461	0.000370424	0.005376242	translation machinery-associated protein 22

MGG_04509	1.66101151	30.45469995	18.33503245	4.196530915	1.02378E-06	3.85929E-05	uncharacterized protein MGG_04509 [Pyricularia oryzae 70-15]
MGG_04510	1.064755544	167.7542033	157.5518477	7.299682863	5.97091E-24	6.04455E-21	uncharacterized protein MGG_04510 [Pyricularia oryzae 70-15]
MGG_04512	2561.661262	1231.396358	0.48070226	-1.056784509	0.009655721	0.063507145	uncharacterized protein MGG_04512 [Pyricularia oryzae 70-15]
MGG_04516	762.9056633	323.7892993	0.424415907	-1.236449365	0.002472438	0.02366838	glycosyl transferase family 17 protein [Pyricularia oryzae 70-15]
MGG_04522	2786.067814	480.9502447	0.172626898	-2.534270817	1.66472E-09	1.21096E-07	uncharacterized protein MGG_04522 [Pyricularia oryzae 70-15]
MGG_04525	669.8213516	252.361633	0.376759613	-1.408283773	0.003018089	0.027381434	uncharacterized protein MGG_04525 [Pyricularia oryzae 70-15]
MGG_04526	1437.922955	671.5069785	0.466997885	-1.098512079	0.006660118	0.049123929	uncharacterized protein MGG_04526 [Pyricularia oryzae 70-15]
MGG_04544	290.7762069	617.3305051	2.123043394	1.08613386	0.007423184	0.053138972	F1F0 ATP synthase assembly protein Atp11
MGG_04554	153.0716776	30.28663369	0.197859161	-2.337454231	1.38823E-06	5.00424E-05	uncharacterized protein MGG_04554 [Pyricularia oryzae 70-15]
MGG_04557	135.0536838	58.39500731	0.432383669	-1.209616061	0.007889649	0.055209364	uncharacterized protein MGG_04557 [Pyricularia oryzae 70-15]
MGG_04566	863.7953097	2202.5775	2.549883607	1.350431394	0.022753649	0.113901884	RWD domain-containing protein [Pyricularia oryzae 70-15]
MGG_04568	1987.22662	929.1475685	0.467559945	-1.096776754	0.006063842	0.045924909	uncharacterized protein MGG_04568 [Pyricularia oryzae 70-15]
MGG_04574	522.2773264	1250.038952	2.393439059	1.259085073	0.002049799	0.02059633	3-beta-hydroxysteroid-Delta(8),Delta(7)-isomerase
MGG_04580	3.927620372	0	0	-Inf	0.047106033	0.185672967	uncharacterized protein MGG_04580 [Pyricularia oryzae 70-15]
MGG_04593	1149.321731	352.5669055	0.306760845	-1.704813746	3.88534E-05	0.000851969	serine/threonine protein kinase [Pyricularia oryzae 70-15]
MGG_04598	6.475901656	19.40731337	2.996851157	1.583447432	0.033226401	0.148157889	uncharacterized protein MGG_04598 [Pyricularia oryzae 70-15]
MGG_04603	366.7917483	779.1784249	2.124307399	1.086992547	0.007479361	0.053383827	uncharacterized protein MGG_04603 [Pyricularia oryzae 70-15]
MGG_04606	239.2760683	106.4869774	0.445038144	-1.1679991	0.007139953	0.051721016	uncharacterized protein MGG_04606 [Pyricularia oryzae 70-15]
MGG_04631	1092.041846	276.2716981	0.252986366	-1.982868458	0.029700423	0.13713445	terpene synthase metal binding domain-containing protein
MGG_04632	2300.416439	514.8619417	0.223812495	-2.159637516	0.044458758	0.179258724	uncharacterized protein MGG_04632 [Pyricularia oryzae 70-15]
MGG_04635	11150.23735	1935.760904	0.173607148	-2.526101747	0.008361304	0.057385941	uncharacterized protein MGG_04635 [Pyricularia oryzae 70-15]
MGG_04644	295.3213045	119.6298665	0.405083767	-1.303707822	0.002762511	0.025735419	uncharacterized protein MGG_04644 [Pyricularia oryzae 70-15]
MGG_04646	1100.050671	464.7259353	0.422458663	-1.243117914	0.002599702	0.024538602	2-succinylbenzoate-CoA ligase [Pyricularia oryzae 70-15]
MGG_04654	1943.010276	836.9092988	0.43072819	-1.215150349	0.002516019	0.02388884	uncharacterized protein MGG_04654 [Pyricularia oryzae 70-15]
MGG_04657	7915.298904	2383.260537	0.301095457	-1.731707156	0.001433423	0.015597758	general alpha-glucoside permease [Pyricularia oryzae 70-15]
MGG_04668	127.0074035	275.976638	2.172917723	1.119633548	0.00885428	0.05988964	uncharacterized protein MGG_04668 [Pyricularia oryzae 70-15]
MGG_04698	780.5208726	379.020282	0.485599162	-1.042162162	0.012473921	0.0758045	PQ-loop repeat-containing protein 2 [Pyricularia oryzae 70-15]
MGG_04723	81.29999411	35.70723224	0.43920338	-1.187038936	0.014534993	0.084081462	uncharacterized protein MGG_04723 [Pyricularia oryzae 70-15]
MGG_04728	164.0272751	66.9427938	0.408119892	-1.292935065	0.028983028	0.134593099	uncharacterized protein MGG_04728 [Pyricularia oryzae 70-15]
MGG_04733	17.64864748	44.57635876	2.525766284	1.336721149	0.020956008	0.10800746	alkaline protease [Pyricularia oryzae 70-15]
MGG_04736	409.1245608	1002.523944	2.450412514	1.29302464	0.026395302	0.126539119	uncharacterized protein MGG_04736 [Pyricularia oryzae 70-15]
MGG_04749	2.367836579	11.27777183	4.762901261	2.251840641	0.032761288	0.146749311	uncharacterized protein MGG_04749 [Pyricularia oryzae 70-15]
MGG_04751	243.1738635	49.5072469	0.203587862	-2.296276547	3.98547E-07	1.63566E-05	uncharacterized protein MGG_04751 [Pyricularia oryzae 70-15]
MGG_04762	422.4550009	187.5479619	0.443947785	-1.171538092	0.004984053	0.039624529	uncharacterized protein MGG_04762 [Pyricularia oryzae 70-15]
MGG_04770	13.02806136	50.64702046	3.887533152	1.958854978	0.000781019	0.009651905	uncharacterized protein MGG_04770 [Pyricularia oryzae 70-15]
MGG_04775	2052.044191	965.3482701	0.470432496	-1.087940375	0.007288145	0.05248645	fatty acid synthase S-acetyltransferase [Pyricularia oryzae 70-15]
MGG_04778	367.4396297	779.6562702	2.121862225	1.085330983	0.008088555	0.056020392	uncharacterized protein MGG_04778 [Pyricularia oryzae 70-15]
MGG_04787	20.17647704	3.308068563	0.163956699	-2.60861325	0.002101839	0.02098067	tetracenomycin polyketide synthesis O-methyltransferase tcmP
MGG_04815	68.96431952	265.9767889	3.856730419	1.947378307	0.021031034	0.108164692	uncharacterized protein MGG_04815 [Pyricularia oryzae 70-15]
MGG_04819	864.2289581	267.7115583	0.309769252	-1.690734145	4.69705E-05	0.000994072	uncharacterized protein MGG_04819 [Pyricularia oryzae 70-15]
MGG_04824	161.621284	542.3391691	3.355617253	1.746578169	0.000198018	0.003269907	acylphosphatase [Pyricularia oryzae 70-15]
MGG_04828	10469.85979	3650.81318	0.348697428	-1.519952371	0.000155766	0.002676437	uncharacterized protein MGG_04828 [Pyricularia oryzae 70-15]
MGG_04835	295.8971408	135.7111856	0.458643112	-1.124556121	0.007810117	0.054810689	uncharacterized protein MGG_04835 [Pyricularia oryzae 70-15]
MGG_04836	719.7416111	259.9010955	0.361103334	-1.469516353	0.0004207	0.005921979	uncharacterized protein MGG_04836 [Pyricularia oryzae 70-15]
MGG_04839	2123.459968	4708.196229	2.217228626	1.148757539	0.00452894	0.036966923	peroxisomal dehydratase [Pyricularia oryzae 70-15]
MGG_04847	728.3257832	1861.250649	2.555519373	1.353616528	0.000907367	0.010881236	peptidase M14 [Pyricularia oryzae 70-15]
MGG_04888	1328.59459	559.1011495	0.420821486	-1.248719729	0.02982006	0.137634533	MFS hexose transporter [Pyricularia oryzae 70-15]
MGG_04890	905.1804652	401.826603	0.443918775	-1.171632369	0.004522074	0.036942943	plasma membrane calcium-transporting ATPase 4

MGG_04894	1309.222704	3180.08562	2.428987529	1.280355083	0.001526971	0.016386613	histone H1-binding protein [Pyricularia oryzae 70-15]
MGG_04897	621.3989934	233.0959648	0.375114809	-1.414595875	0.021376728	0.109386898	uncharacterized protein MGG_04897 [Pyricularia oryzae 70-15]
MGG_04901	1698.929735	3485.64264	2.051669689	1.036798481	0.010334116	0.066740481	methyltransferase [Pyricularia oryzae 70-15]
MGG_04903	908.2439524	313.5265798	0.345200845	-1.534492099	0.000215517	0.003517425	uncharacterized protein MGG_04903 [Pyricularia oryzae 70-15]
MGG_04909	303.067249	622.1916527	2.052982151	1.037721084	0.010113817	0.065878096	mitochondrial import protein mmp37 [Pyricularia oryzae 70-15]
MGG_04910	119.0370757	18.31674029	0.153874246	-2.700176306	1.325E-07	6.05118E-06	uncharacterized protein MGG_04910 [Pyricularia oryzae 70-15]
MGG_04911	797.3432618	174.9590233	0.219427481	-2.188183877	3.15494E-07	1.32159E-05	cytochrome P450 3A5 [Pyricularia oryzae 70-15]
MGG_04917	1580.310144	526.7361038	0.333311854	-1.585055469	0.00012438	0.002240985	uncharacterized protein MGG_04917 [Pyricularia oryzae 70-15]
MGG_04923	1192.476291	464.4547616	0.389487628	-1.360350595	0.000854937	0.010417026	uncharacterized protein MGG_04923 [Pyricularia oryzae 70-15]
MGG_04927	803.0056512	2240.719288	2.790415341	1.480479877	0.000331735	0.004950759	high-affinity nicotinic acid transporter [Pyricularia oryzae 70-15]
MGG_04939	653.6957463	234.9789807	0.359462307	-1.476087598	0.005663599	0.043756998	uncharacterized protein MGG_04939 [Pyricularia oryzae 70-15]
MGG_04956	5507.478275	806.1678448	0.146376945	-2.772239754	6.10405E-05	0.001242078	uncharacterized protein MGG_04956 [Pyricularia oryzae 70-15]
MGG_04962	1899.294276	862.9540449	0.454355102	-1.138107815	0.004310635	0.035478045	uncharacterized protein MGG_04962 [Pyricularia oryzae 70-15]
MGG_04974	9685.710915	2994.867796	0.309204747	-1.693365626	0.022721431	0.113901884	uncharacterized protein MGG_04974 [Pyricularia oryzae 70-15]
MGG_04980	238.9855699	661.7709067	2.769083116	1.469408358	0.002936057	0.026878084	uncharacterized protein MGG_04980 [Pyricularia oryzae 70-15]
MGG_04984	3898.197437	1852.333988	0.475177057	-1.073462913	0.007313166	0.052517239	uncharacterized protein MGG_04984 [Pyricularia oryzae 70-15]
MGG_04990	1108.305306	2557.189762	2.30729723	1.206203866	0.003214534	0.028481764	uncharacterized protein MGG_04990 [Pyricularia oryzae 70-15]
MGG_05009	1005.18202	483.5683952	0.481075453	-1.055664909	0.034364328	0.150870203	canalicular multispecific organic anion transporter 2
MGG_05010	141.8595553	56.4107569	0.397652148	-1.330421131	0.008775722	0.059424452	uncharacterized protein MGG_05010 [Pyricularia oryzae 70-15]
MGG_05011	7583.435263	1441.707505	0.19011272	-2.395073032	0.007247053	0.052278624	initiation-specific alpha-1,6-mannosyltransferase
MGG_05012	566.3175066	130.2535129	0.230000859	-2.120288846	3.01651E-06	9.6433E-05	uncharacterized protein MGG_05012 [Pyricularia oryzae 70-15]
MGG_05030	3668.317173	1647.655122	0.449158305	-1.154704087	0.047078066	0.185622961	arrestin [Pyricularia oryzae 70-15]
MGG_05046	516.9152352	173.3531741	0.33536093	-1.576213471	0.00021443	0.003505915	adenine phosphoribosyltransferase [Pyricularia oryzae 70-15]
MGG_05051	25.93621224	3.970809374	0.153099047	-2.707462793	0.000336589	0.005017034	uncharacterized protein MGG_05051 [Pyricularia oryzae 70-15]
MGG_05058	1053.210648	5895.303546	5.597459119	2.484772086	5.5592E-08	2.82566E-06	uncharacterized protein MGG_05058 [Pyricularia oryzae 70-15]
MGG_05059	4052.146166	10082.07485	2.488082719	1.31503445	0.033779595	0.149738831	scytalone dehydratase [Pyricularia oryzae 70-15]
MGG_05063	9303.519783	24683.19794	2.653103182	1.407680785	0.000480115	0.006602445	phosphoglycerate kinase [Pyricularia oryzae 70-15]
MGG_05065	2253.384083	1045.385676	0.463918106	-1.108057941	0.005820966	0.044733922	mitochondrial outer membrane protein IML2
MGG_05112	1655.078506	823.3256502	0.497454137	-1.007364573	0.012067979	0.074049795	ATP-dependent RNA helicase MRH4 [Pyricularia oryzae 70-15]
MGG_05116	4569.236968	1236.056241	0.270516992	-1.886208877	3.81435E-06	0.000119425	malic acid transporter [Pyricularia oryzae 70-15]
MGG_05118	1274.291757	630.1722456	0.494527444	-1.015877509	0.028052691	0.131678551	uncharacterized protein MGG_05118 [Pyricularia oryzae 70-15]
MGG_05121	732.2713229	244.7979843	0.334299564	-1.580786619	0.000160577	0.002747454	uncharacterized protein MGG_05121 [Pyricularia oryzae 70-15]
MGG_05122	997.3698972	217.6813683	0.218255402	-2.19591073	0.0465273	0.18424459	uncharacterized protein MGG_05122 [Pyricularia oryzae 70-15]
MGG_05123	6635.678347	1341.065399	0.202099217	-2.306864362	0.015048053	0.085783082	uncharacterized protein MGG_05123 [Pyricularia oryzae 70-15]
MGG_05124	1554.592168	265.6441569	0.170877071	-2.548969269	2.31767E-09	1.61811E-07	uncharacterized protein MGG_05124 [Pyricularia oryzae 70-15]
MGG_05125	2985.099951	879.5522213	0.294647494	-1.762938097	0.019784062	0.103593441	uncharacterized protein MGG_05125 [Pyricularia oryzae 70-15]
MGG_05128	296.6587348	710.1835227	2.393941049	1.259387626	0.001989799	0.020160196	dicarboxylic amino acid permease [Pyricularia oryzae 70-15]
MGG_05131	240.1467297	95.56722527	0.397953474	-1.329328326	0.002042902	0.020544019	uncharacterized protein MGG_05131 [Pyricularia oryzae 70-15]
MGG_05132	43.13806537	154.497402	3.581463393	1.840549196	0.000114547	0.002114772	uncharacterized protein MGG_05132 [Pyricularia oryzae 70-15]
MGG_05163	31.27955721	7.679848321	0.245522923	-2.026070367	0.002807297	0.02607266	uncharacterized protein MGG_05163 [Pyricularia oryzae 70-15]
MGG_05164	6774.107217	15136.70405	2.234494312	1.159948372	0.032492791	0.145869337	uncharacterized protein MGG_05164 [Pyricularia oryzae 70-15]
MGG_05167	554.4146343	1250.428892	2.255403834	1.173385774	0.003988151	0.033528069	uncharacterized protein MGG_05167 [Pyricularia oryzae 70-15]
MGG_05172	26.20314086	2.123754504	0.081049616	-3.62505084	2.40009E-05	0.000562863	uncharacterized protein MGG_05172 [Pyricularia oryzae 70-15]
MGG_05181	266.5718297	99.04713841	0.37155891	-1.428337131	0.001138937	0.013077327	uncharacterized protein MGG_05181 [Pyricularia oryzae 70-15]
MGG_05188	3323.242479	7140.657424	2.148701899	1.103465344	0.006383494	0.047986812	uncharacterized protein MGG_05188 [Pyricularia oryzae 70-15]
MGG_05192	8.943775973	32.54458721	3.638797227	1.863461659	0.004339789	0.035693806	uncharacterized protein MGG_05192 [Pyricularia oryzae 70-15]
MGG_05214	43.54328174	122.5494013	2.814427309	1.492841387	0.030545528	0.13991971	uncharacterized protein MGG_05214 [Pyricularia oryzae 70-15]
MGG_05216	1791.045264	737.473625	0.411755995	-1.280138439	0.00166411	0.017533054	uncharacterized protein MGG_05216 [Pyricularia oryzae 70-15]

MGG_05230	1081.466997	419.6078899	0.387998793	-1.365875929	0.03327795	0.148238418	uncharacterized protein MGG_05230 [Pyricularia oryzae 70-15]
MGG_05232	1016.660169	272.1586247	0.267698719	-1.901317861	0.004192769	0.034818332	uncharacterized protein MGG_05232 [Pyricularia oryzae 70-15]
MGG_05235	1102.081092	279.7193444	0.253810129	-1.978178449	5.13016E-05	0.001070811	uncharacterized protein MGG_05235 [Pyricularia oryzae 70-15]
MGG_05240	217.2109447	74.16298383	0.341432997	-1.550325604	0.000861675	0.010446732	MYB DNA-binding domain-containing protein
MGG_05247	38320.37933	12696.79939	0.331332821	-1.593646975	0.000103697	0.001938012	NAD-specific glutamate dehydrogenase [Pyricularia oryzae 70-15]
MGG_05274	1369.78707	3150.657582	2.300107551	1.201701322	0.003269744	0.028762379	COP9 signalosome complex subunit 5 [Pyricularia oryzae 70-15]
MGG_05287	3224.267741	1245.691901	0.386348778	-1.372024261	0.000722351	0.00908397	uncharacterized protein MGG_05287 [Pyricularia oryzae 70-15]
MGG_05288	215.152792	83.35675102	0.387430487	-1.36799061	0.003126762	0.028073836	uncharacterized protein MGG_05288 [Pyricularia oryzae 70-15]
MGG_05300	189.8776271	91.95200092	0.484269802	-1.046117052	0.015651871	0.087905189	uncharacterized protein MGG_05300 [Pyricularia oryzae 70-15]
MGG_05302	758.1526701	207.8656864	0.274173916	-1.866836771	9.73582E-06	0.000270024	uncharacterized protein MGG_05302 [Pyricularia oryzae 70-15]
MGG_05306	6965.757192	2187.788416	0.314077617	-1.670806965	3.28678E-05	0.000744921	uncharacterized protein MGG_05306 [Pyricularia oryzae 70-15]
MGG_05308	46.41518245	21.71354888	0.467811344	-1.096001249	0.040466294	0.168120569	uncharacterized protein MGG_05308 [Pyricularia oryzae 70-15]
MGG_05324	672.3112119	332.903153	0.495162281	-1.014026674	0.014223618	0.082757225	mitochondrial nuclease [Pyricularia oryzae 70-15]
MGG_05327	64.54374535	31.0005794	0.480303385	-1.057982117	0.039227997	0.164347945	uncharacterized protein MGG_05327 [Pyricularia oryzae 70-15]
MGG_05332	3223.20027	1533.706177	0.475833349	-1.071471709	0.008992716	0.060556271	1-phosphatidylinositol-4,5-bisphosphate phosphodiesterase delta 1
MGG_05337	1554.91428	519.606291	0.334170377	-1.581344246	9.43374E-05	0.001796256	glucooligosaccharide oxidase [Pyricularia oryzae 70-15]
MGG_05339	517.3260262	1643.856306	3.177602174	1.667938515	6.27991E-05	0.001273595	uncharacterized protein MGG_05339 [Pyricularia oryzae 70-15]
MGG_05354	11.92172784	31.71952606	2.660648395	1.411777871	0.025630344	0.123948016	uncharacterized protein MGG_05354 [Pyricularia oryzae 70-15]
MGG_05358	634.5110511	1305.04542	2.05677335	1.040382822	0.01942724	0.102431471	LYR family protein [Pyricularia oryzae 70-15]
MGG_05363	121.6022388	44.54126888	0.366286586	-1.448955227	0.001956629	0.019922315	sugar transporter [Pyricularia oryzae 70-15]
MGG_05364	8599.194912	28982.64323	3.370390312	1.752915674	1.47497E-05	0.000375637	endoglucanase-4 [Pyricularia oryzae 70-15]
MGG_05366	377.1671385	177.8036184	0.471418637	-1.084919299	0.045875697	0.182720645	feruloyl esterase B [Pyricularia oryzae 70-15]
MGG_05383	505.2692627	3713.348256	7.349246293	2.877596301	0.000198996	0.003275614	HHE domain-containing protein [Pyricularia oryzae 70-15]
MGG_05384	1141.329864	477.8890481	0.418712471	-1.255968205	0.002119349	0.021098803	ankyrin repeat domain-containing protein 29
MGG_05386	0	6.265112206	Inf	Inf	0.00605133	0.045898524	uncharacterized protein MGG_05386 [Pyricularia oryzae 70-15]
MGG_05396	7.613281789	33.50140212	4.400389089	2.137631095	0.024178192	0.118817426	uncharacterized protein MGG_05396 [Pyricularia oryzae 70-15]
MGG_05397	2146.3033	4343.443634	2.023685857	1.016985354	0.011635081	0.072205194	CMGC protein kinase [Pyricularia oryzae 70-15]
MGG_05398	2674.635073	7571.814658	2.830971124	1.501297034	0.001693037	0.017791538	uncharacterized protein MGG_05398 [Pyricularia oryzae 70-15]
MGG_05401	2342.466465	514.5770019	0.219673156	-2.186569509	1.57484E-07	7.0856E-06	bifunctional P-450:NADPH-P450 reductase
MGG_05404	1.353694894	9.752397924	7.204280645	2.848854383	0.017324088	0.094458269	uncharacterized protein MGG_05404 [Pyricularia oryzae 70-15]
MGG_05411	898.3048193	338.3555469	0.37666006	-1.408665034	0.000725018	0.009108088	uncharacterized protein MGG_05411 [Pyricularia oryzae 70-15]
MGG_05419	1134.985161	2451.249554	2.159719474	1.110843933	0.00665193	0.04910664	uncharacterized protein MGG_05419 [Pyricularia oryzae 70-15]
MGG_05424	17.19940965	126.8568778	7.375653028	2.882770789	2.58497E-08	1.42737E-06	uncharacterized protein MGG_05424 [Pyricularia oryzae 70-15]
MGG_05427	3518.078992	8478.781302	2.410059956	1.269069038	0.020089422	0.104629142	microtubule-associated protein RP/EB family member 1
MGG_05433	90791.26047	34617.11824	0.381282494	-1.391067801	0.000528473	0.007101648	solute carrier family 6 protein [Pyricularia oryzae 70-15]
MGG_05435	361.1710771	159.9288578	0.442806382	-1.175252081	0.007988371	0.055675695	membrane transporter [Pyricularia oryzae 70-15]
MGG_05436	1215.867957	5055.895081	4.158259993	2.055979965	0.003181616	0.028335977	translation machinery-associated protein 20
MGG_05437	2011.505365	4570.833248	2.272344547	1.184181602	0.004262927	0.035159899	uncharacterized protein MGG_05437 [Pyricularia oryzae 70-15]
MGG_05442	701.6017723	209.8096988	0.299043855	-1.741571022	0.008772911	0.059424452	endo alpha-1,4 polygalactosaminidase precusor
MGG_05457	1055.077131	5057.115323	4.793123814	2.260966207	5.84461E-08	2.95835E-06	uncharacterized protein MGG_05457 [Pyricularia oryzae 70-15]
MGG_05459	1823.571988	4755.059238	2.607552249	1.382696161	0.000608879	0.007919333	uncharacterized protein MGG_05459 [Pyricularia oryzae 70-15]
MGG_05473	0.371789801	6.31505767	16.98555919	4.08623681	0.022486556	0.113261817	uncharacterized protein MGG_05473 [Pyricularia oryzae 70-15]
MGG_05479	7479.753755	3175.809489	0.424587439	-1.235866405	0.008192864	0.056517271	xylosidase/arabinosidase [Pyricularia oryzae 70-15]
MGG_05491	1.303081035	9.056571654	6.950121604	2.79703822	0.028002472	0.131598953	L-aminoadipate-semialdehyde dehydrogenase
MGG_05504	11.88133985	75.20445632	6.329627575	2.662120616	3.80906E-05	0.000836754	uncharacterized protein MGG_05504 [Pyricularia oryzae 70-15]
MGG_05505	2077.270976	9560.752466	4.602554302	2.202434743	1.19334E-07	5.5331E-06	autophagy protein [Pyricularia oryzae 70-15]
MGG_05506	365.4083238	1964.691918	5.37670269	2.426721699	9.46038E-09	5.83374E-07	glyoxylate reductase [Pyricularia oryzae 70-15]
MGG_05514	397.701851	1517.338231	3.815265701	1.931783532	2.98801E-06	9.60273E-05	uncharacterized protein MGG_05514 [Pyricularia oryzae 70-15]

MGG_05515	230.8415998	907.1046829	3.929554655	1.974365818	2.65918E-06	8.76204E-05	uncharacterized protein MGG_05515 [Pyricularia oryzae 70-15]
MGG_05518	8.104676574	34.58405135	4.267172297	2.093280364	0.001323456	0.014695927	uncharacterized protein MGG_05518 [Pyricularia oryzae 70-15]
MGG_05520	1202.814629	121.7069026	0.101185087	-3.304931422	0.011155814	0.070218043	exoglucanase 2 [Pyricularia oryzae 70-15]
MGG_05531	1845.85186	3693.612665	2.001034181	1.000745811	0.014319892	0.083153942	uncharacterized protein MGG_05531 [Pyricularia oryzae 70-15]
MGG_05532	43.52490448	148.8894319	3.420787103	1.774328319	0.00015815	0.00271357	uncharacterized protein MGG_05532 [Pyricularia oryzae 70-15]
MGG_05533	13.47336023	57.3944337	4.259845555	2.090801125	0.000224445	0.003634799	chitinase 1 [Pyricularia oryzae 70-15]
MGG_05539	343.6034729	803.47964	2.338392081	1.225516848	0.003224845	0.028493392	carboxypeptidase 2 [Pyricularia oryzae 70-15]
MGG_05551	143.870747	440.2789127	3.060239291	1.613644467	0.000188717	0.003139337	uncharacterized protein MGG_05551 [Pyricularia oryzae 70-15]
MGG_05552	403.3293566	1115.222781	2.765042422	1.467301615	0.000365804	0.005341087	uncharacterized protein MGG_05552 [Pyricularia oryzae 70-15]
MGG_05554	0	54.20118527	Inf	Inf	1.41691E-14	2.82175E-12	uncharacterized protein MGG_05554 [Pyricularia oryzae 70-15]
MGG_05555	5.098121574	77.60076493	15.2214426	3.928033191	3.32199E-10	2.84194E-08	xenobiotic compound monooxygenase [Pyricularia oryzae 70-15]
MGG_05575	27.7758995	917.040006	33.01567267	5.045079135	1.46494E-24	1.61782E-21	uncharacterized protein MGG_05575 [Pyricularia oryzae 70-15]
MGG_05577	11.73764968	47.71061514	4.064750309	2.023166732	0.000803399	0.00986824	uncharacterized protein MGG_05577 [Pyricularia oryzae 70-15]
MGG_05580	19.94926189	47.19404283	2.365703709	1.242269395	0.022506068	0.113304481	CAIB/BAIF family enzyme [Pyricularia oryzae 70-15]
MGG_05587	782.2236276	2033.001636	2.599003103	1.377958357	0.000751499	0.009337677	actin-like protein arp-6 [Pyricularia oryzae 70-15]
MGG_05589	101.326704	218.8072153	2.159423001	1.110645875	0.011402302	0.07117943	lovastatin nonaketide synthase [Pyricularia oryzae 70-15]
MGG_05591	21.09448792	64.30413046	3.048385469	1.608045344	0.002801056	0.026034606	uncharacterized protein MGG_05591 [Pyricularia oryzae 70-15]
MGG_05596	202.5553619	417.5642232	2.061481954	1.043681832	0.031787653	0.143712843	hydrolase [Pyricularia oryzae 70-15]
MGG_05599	1592.77766	4167.485347	2.616489075	1.387632235	0.000742031	0.009254817	beta-glucosidase 1 [Pyricularia oryzae 70-15]
MGG_05600	42.6439216	127.2847469	2.98482743	1.577647523	0.000917867	0.010985462	quinate permease [Pyricularia oryzae 70-15]
MGG_05627	1458.713537	496.3060676	0.340235458	-1.555394596	0.000144365	0.002516131	uncharacterized protein MGG_05627 [Pyricularia oryzae 70-15]
MGG_05638	1900.783937	7554.005611	3.974152698	1.990647306	0.001319862	0.014682863	uncharacterized protein MGG_05638 [Pyricularia oryzae 70-15]
MGG_05647	886.664503	1782.986193	2.010891591	1.007835307	0.01225481	0.074622274	50S ribosomal protein L2 [Pyricularia oryzae 70-15]
MGG_05648	988.8692057	423.479434	0.428246154	-1.223487806	0.007288825	0.05248645	uncharacterized protein MGG_05648 [Pyricularia oryzae 70-15]
MGG_05656	637.3102502	298.7730141	0.468803089	-1.092946019	0.008290272	0.056995037	ankyrin repeat protein [Pyricularia oryzae 70-15]
MGG_05662	739.0684894	1597.67361	2.161739586	1.112192739	0.00607149	0.045954184	shwachman-Bodian-Diamond syndrome protein
MGG_05663	6939.147265	15879.13233	2.288340587	1.194301793	0.002986864	0.027195876	carboxypeptidase Y [Pyricularia oryzae 70-15]
MGG_05679	1170.95135	2425.240129	2.071170702	1.050446463	0.01508168	0.085813701	uncharacterized protein MGG_05679 [Pyricularia oryzae 70-15]
MGG_05683	5357.960409	2365.198678	0.441436386	-1.179722545	0.00337945	0.029471327	uncharacterized protein MGG_05683 [Pyricularia oryzae 70-15]
MGG_05694	1223.403002	600.2807996	0.490664809	-1.027190293	0.01132332	0.071033049	Gtr1/RagA G domain-containing protein [Pyricularia oryzae 70-15]
MGG_05702	494.138336	1018.642461	2.061452	1.043660869	0.010778258	0.068587887	methyltransferase [Pyricularia oryzae 70-15]
MGG_05710	249.9382211	122.6119613	0.490569073	-1.027471811	0.014962888	0.085538429	uncharacterized protein MGG_05710 [Pyricularia oryzae 70-15]
MGG_05719	323.3892299	660.0310523	2.040980315	1.029262268	0.013768625	0.080919812	heat shock protein 30 [Pyricularia oryzae 70-15]
MGG_05722	47.45340933	13.25783989	0.279386457	-1.839666007	0.001710847	0.017916697	uncharacterized protein MGG_05722 [Pyricularia oryzae 70-15]
MGG_05723	470.3980869	228.3151068	0.485365721	-1.042855872	0.011686135	0.072430185	fluconazole resistance protein 1 [Pyricularia oryzae 70-15]
MGG_05724	1320.438428	586.5854034	0.444235332	-1.170603954	0.00377744	0.032202349	uncharacterized protein MGG_05724 [Pyricularia oryzae 70-15]
MGG_05750	224.8031831	496.3750834	2.208042949	1.142768235	0.005628079	0.043575462	uncharacterized protein MGG_05750 [Pyricularia oryzae 70-15]
MGG_05763	98.31199763	375.6577508	3.821077385	1.933979475	0.000300459	0.004591438	stress responsive A/B barrel domain-containing protein
MGG_05766	2686.306741	1071.965049	0.399047895	-1.325366183	0.001940311	0.019808939	uncharacterized protein MGG_05766 [Pyricularia oryzae 70-15]
MGG_05788	52.64912516	145.6283491	2.766016504	1.467809764	0.001439921	0.015602334	Poly(3-hydroxybutyrate) depolymerase [Pyricularia oryzae 70-15]
MGG_05805	232.7735109	97.69193507	0.41968665	-1.252615521	0.004198409	0.034818332	uncharacterized protein MGG_05805 [Pyricularia oryzae 70-15]
MGG_05806	3737.976847	601.2718	0.160854875	-2.636168431	4.68752E-10	3.87374E-08	uncharacterized protein MGG_05806 [Pyricularia oryzae 70-15]
MGG_05807	2378.241225	418.6155172	0.176018947	-2.50619736	0.001826632	0.018878165	uncharacterized protein MGG_05807 [Pyricularia oryzae 70-15]
MGG_05812	1310.491779	2749.07309	2.097741576	1.068836961	0.008300297	0.057031678	phosphomevalonate kinase [Pyricularia oryzae 70-15]
MGG_05819	8.999213275	30.34154329	3.37157731	1.753423679	0.00775736	0.054534963	uncharacterized protein MGG_05819 [Pyricularia oryzae 70-15]
MGG_05824	73.67849739	25.31072741	0.343529365	-1.541494669	0.003436265	0.029816962	uncharacterized protein MGG_05824 [Pyricularia oryzae 70-15]
MGG_05826	98.11100564	197.0120947	2.008052954	1.005797315	0.02248827	0.113261817	epoxide hydrolase 2 [Pyricularia oryzae 70-15]
MGG_05827	8.029977528	54.5476869	6.793006171	2.764050165	0.009293231	0.061893734	glutamyl-tRNA(Gln) amidotransferase subunit A

MGG_05828	1.37207216	191.2480173	139.3862676	7.122944623	1.11368E-06	4.13731E-05	chitin deacetylase 1 [Pyricularia oryzae 70-15]
MGG_05829	21.6992008	84.67940963	3.902420666	1.964369303	0.002809554	0.026073691	uncharacterized protein MGG_05829 [Pyricularia oryzae 70-15]
MGG_05830	4.014988764	24.03467679	5.986237623	2.581649548	0.001258622	0.014183429	allantoin transporter [Pyricularia oryzae 70-15]
MGG_05831	1.321458302	11.65313976	8.818393851	3.140515913	0.00693471	0.050687641	uncharacterized protein MGG_05831 [Pyricularia oryzae 70-15]
MGG_05832	2.386213846	13.01296628	5.453394843	2.447154615	0.013990141	0.081747106	uncharacterized protein MGG_05832 [Pyricularia oryzae 70-15]
MGG_05849	563.3821386	2509.967252	4.455177189	2.155482811	0.000423817	0.005956541	uncharacterized protein MGG_05849 [Pyricularia oryzae 70-15]
MGG_05857	1006.397688	2137.978755	2.124387586	1.087047004	0.007505775	0.053540903	histone deacetylase RPD3 [Pyricularia oryzae 70-15]
MGG_05874	704.0365955	1644.119793	2.33527604	1.223593093	0.002630354	0.024770184	uncharacterized protein MGG_05874 [Pyricularia oryzae 70-15]
MGG_05880	364.4059544	2465.93421	6.766997575	2.758515872	9.36867E-11	9.2529E-09	uncharacterized protein MGG_05880 [Pyricularia oryzae 70-15]
MGG_05881	425.460188	869.5983473	2.043900632	1.031325059	0.013518936	0.080072179	uncharacterized protein MGG_05881 [Pyricularia oryzae 70-15]
MGG_05883	119.2863651	634.9252322	5.322697457	2.412157565	4.24621E-08	2.22341E-06	hypothetical protein, variant [Pyricularia oryzae 70-15]
MGG_05889	2263.624264	684.4562454	0.302371845	-1.725604283	0.005644297	0.043673194	lactose permease [Pyricularia oryzae 70-15]
MGG_05896	21.879951	82.90372033	3.789026782	1.921827337	0.000240337	0.003855662	uncharacterized protein MGG_05896 [Pyricularia oryzae 70-15]
MGG_05902	173.6462312	53.15563032	0.306114506	-1.707856681	0.000198111	0.003269907	uncharacterized protein MGG_05902 [Pyricularia oryzae 70-15]
MGG_05907	35.25925489	11.94425414	0.338755149	-1.56168522	0.044132547	0.178291381	uncharacterized protein MGG_05907 [Pyricularia oryzae 70-15]
MGG_05927	83.61056091	207.1946132	2.478091416	1.309229409	0.011089662	0.069913293	uncharacterized protein MGG_05927 [Pyricularia oryzae 70-15]
MGG_05940	1303.326609	2882.402019	2.211573062	1.145072904	0.005066616	0.040123372	short chain dehydrogenase [Pyricularia oryzae 70-15]
MGG_05941	3347.064324	202.9828129	0.060645029	-4.043466796	9.71466E-05	0.001838219	maltose permease MAL31 [Pyricularia oryzae 70-15]
MGG_05943	3.231021165	36.71903037	11.36452796	3.506465856	3.30564E-06	0.000104304	uncharacterized protein MGG_05943 [Pyricularia oryzae 70-15]
MGG_05957	549.0458551	1154.338875	2.102445296	1.072068263	0.008026515	0.05574274	50S ribosomal protein L1 [Pyricularia oryzae 70-15]
MGG_05982	30952.6185	66695.62282	2.154765124	1.10753062	0.006459808	0.048291537	uncharacterized protein MGG_05982 [Pyricularia oryzae 70-15]
MGG_05989	3538.791297	9513.02984	2.688214433	1.426648223	0.001503546	0.016163784	seprase [Pyricularia oryzae 70-15]
MGG_06000	10283.06721	28877.11231	2.808219739	1.489655829	0.007296549	0.052489992	uncharacterized protein MGG_06000 [Pyricularia oryzae 70-15]
MGG_06007	8.035685449	1.281759729	0.15950845	-2.648295243	0.041062666	0.170016791	uncharacterized protein MGG_06007 [Pyricularia oryzae 70-15]
MGG_06021	1741.600925	564.6590067	0.324218366	-1.624962275	0.030510707	0.139812927	uncharacterized protein MGG_06021 [Pyricularia oryzae 70-15]
MGG_06022	1545.309838	673.5201583	0.435847971	-1.1981031	0.003486937	0.030148975	uncharacterized protein MGG_06022 [Pyricularia oryzae 70-15]
MGG_06026	8674.243808	3011.654112	0.347195004	-1.526181907	0.000342587	0.005069114	uncharacterized protein MGG_06026 [Pyricularia oryzae 70-15]
MGG_06030	2753.321703	8012.216073	2.91001813	1.541028142	0.007052744	0.051180845	uncharacterized protein MGG_06030 [Pyricularia oryzae 70-15]
MGG_06036	623.2981143	308.4457343	0.494860689	-1.014905654	0.013132432	0.078665079	general amino acid permease [Pyricularia oryzae 70-15]
MGG_06039	1628.715067	779.1202639	0.478364988	-1.063816293	0.009647871	0.063507145	cortical actin cytoskeleton protein asp1 [Pyricularia oryzae 70-15]
MGG_06059	1931.153849	760.1614001	0.393630679	-1.34508543	0.000898776	0.010810226	arrestin [Pyricularia oryzae 70-15]
MGG_06062	389.2276949	1446.616795	3.71663377	1.893996535	5.95513E-06	0.000176878	nitrate reductase [Pyricularia oryzae 70-15]
MGG_06064	1215.265656	256.0277318	0.210676349	-2.24689973	1.15024E-07	5.37429E-06	chitin synthase D [Pyricularia oryzae 70-15]
MGG_06068	1852.947206	705.63727	0.380818875	-1.392823109	0.000658892	0.008434378	uncharacterized protein MGG_06068 [Pyricularia oryzae 70-15]
MGG_06069	616.7274691	124.6777729	0.20216024	-2.306428816	2.10041E-06	7.20783E-05	endoglucanase [Pyricularia oryzae 70-15]
MGG_06076	152.6269577	542.5716122	3.554887159	1.829803766	0.001296994	0.014467249	kinetochore protein SPC24 [Pyricularia oryzae 70-15]
MGG_06088	2822.356966	10061.99892	3.565104995	1.833944566	0.000268542	0.004220248	uncharacterized protein MGG_06088 [Pyricularia oryzae 70-15]
MGG_06090	8437.957929	1365.477263	0.16182556	-2.627488603	4.28229E-10	3.5631E-08	uncharacterized protein MGG_06090 [Pyricularia oryzae 70-15]
MGG_06112	2014.36644	797.0252441	0.395670434	-1.337628828	0.017985485	0.097019392	hydrolase [Pyricularia oryzae 70-15]
MGG_06129	543.0302822	1252.330724	2.30618948	1.205511052	0.003351437	0.029332321	uncharacterized protein MGG_06129 [Pyricularia oryzae 70-15]
MGG_06156	1407.294574	257.4762353	0.182958309	-2.45041316	7.99686E-09	5.04104E-07	hypothetical protein, variant [Pyricularia oryzae 70-15]
MGG_06176	749.8935306	352.0493411	0.469465766	-1.090908135	0.007602716	0.054019894	uncharacterized protein MGG_06176 [Pyricularia oryzae 70-15]
MGG_06177	15964.85471	34620.6539	2.168554274	1.116733551	0.018476881	0.0988798	uncharacterized protein MGG_06177, partial
MGG_06195	6365.824432	2074.013931	0.32580445	-1.617921784	0.008248881	0.056774737	uncharacterized protein MGG_06195 [Pyricularia oryzae 70-15]
MGG_06200	747.0842894	1506.234114	2.016150165	1.011603096	0.011846978	0.073165781	uncharacterized protein MGG_06200 [Pyricularia oryzae 70-15]
MGG_06203	3112.919389	7447.64725	2.39249602	1.258516525	0.001937345	0.019808939	high-affinity glucose transporter RGT2 [Pyricularia oryzae 70-15]
MGG_06212	82.56299265	37.40070922	0.452996046	-1.142429637	0.020534699	0.106319632	uncharacterized protein MGG_06212 [Pyricularia oryzae 70-15]
MGG_06215	1374.391347	558.4522354	0.406326944	-1.299287062	0.001436771	0.015597758	uncharacterized protein MGG_06215 [Pyricularia oryzae 70-15]

MGG_06216	562.8867475	277.5416084	0.493068294	-1.020140609	0.013964678	0.081676894	uncharacterized protein MGG_06216 [Pyricularia oryzae 70-15]
MGG_06220	150.0495579	49.77894559	0.331750032	-1.591831491	0.002454637	0.023516503	uncharacterized protein MGG_06220 [Pyricularia oryzae 70-15]
MGG_06249	13752.95221	30275.40445	2.201374948	1.138404892	0.004760049	0.038473106	translationally controlled tumor protein [Pyricularia oryzae 70-15]
MGG_06257	2315.475511	1117.068847	0.482436044	-1.051590395	0.007723032	0.054462295	uncharacterized protein MGG_06257 [Pyricularia oryzae 70-15]
MGG_06289	1410.837686	3494.206979	2.476689568	1.30841305	0.001372653	0.015104425	NADH-cytochrome b5 reductase 1 [Pyricularia oryzae 70-15]
MGG_06296	542.6336057	1408.613768	2.595883767	1.376225787	0.023333325	0.115963817	nuclear cap-binding protein subunit 2 [Pyricularia oryzae 70-15]
MGG_06298	7289.116788	3303.178429	0.453165798	-1.141889113	0.004531096	0.036966923	uncharacterized protein MGG_06298 [Pyricularia oryzae 70-15]
MGG_06301	6940.051361	3247.914874	0.467995798	-1.095432519	0.034641142	0.151865967	uncharacterized protein MGG_06301 [Pyricularia oryzae 70-15]
MGG_06304	224.0017862	500.1209861	2.23266517	1.158766908	0.005026994	0.039913675	Mis12 domain-containing protein [Pyricularia oryzae 70-15]
MGG_06307	236.68801	105.1610181	0.444302261	-1.17038661	0.010323097	0.06674028	uncharacterized protein MGG_06307 [Pyricularia oryzae 70-15]
MGG_06310	1400.42852	2871.455563	2.050412087	1.035913888	0.03328897	0.148238418	uncharacterized protein MGG_06310 [Pyricularia oryzae 70-15]
MGG_06312	3680.754914	720.1250653	0.195646024	-2.353682301	0.001709823	0.017916697	C6 zinc finger domain-containing protein
MGG_06314	640.9349129	201.2445115	0.313985878	-1.671228421	7.09567E-05	0.001397054	uncharacterized protein MGG_06314 [Pyricularia oryzae 70-15]
MGG_06323	140.5955453	63.89949219	0.454491585	-1.137674512	0.014137312	0.082442729	solute carrier family 35 member E3 [Pyricularia oryzae 70-15]
MGG_06326	278.84837	40.23388614	0.144285893	-2.79299784	7.00542E-09	4.4556E-07	vacuolar ATP synthase proteolipid subunit
MGG_06344	10.82473571	2.517278689	0.232548744	-2.104394949	0.041788453	0.172145288	uncharacterized protein MGG_06344 [Pyricularia oryzae 70-15]
MGG_06347	2142.092246	474.2404342	0.221391229	-2.17533003	2.09148E-07	8.95872E-06	uncharacterized protein MGG_06347 [Pyricularia oryzae 70-15]
MGG_06379	888.7545359	311.3881739	0.350364652	-1.513070864	0.000241708	0.003868606	midasin [Pyricularia oryzae 70-15]
MGG_06382	68.61423494	25.71122014	0.37472137	-1.416109838	0.008400234	0.05762058	CAMK/CAMK1 protein kinase [Pyricularia oryzae 70-15]
MGG_06415	220.8358681	74.49499709	0.337331964	-1.567759067	0.034212461	0.150366489	uncharacterized protein MGG_06415 [Pyricularia oryzae 70-15]
MGG_06416	1792.407485	891.1512079	0.497181146	-1.008156506	0.011440396	0.071343905	uncharacterized protein MGG_06416 [Pyricularia oryzae 70-15]
MGG_06428	890.3914113	1929.497325	2.167021492	1.115713462	0.006696433	0.04927212	uncharacterized protein MGG_06428 [Pyricularia oryzae 70-15]
MGG_06434	667.5286539	307.2197913	0.460234613	-1.119558608	0.017972305	0.097019392	uncharacterized protein MGG_06434 [Pyricularia oryzae 70-15]
MGG_06445	263.078188	598.253872	2.274053492	1.185266191	0.004211465	0.034874489	histone H3-like centromeric protein cse-4
MGG_06464	8.668695948	1.631525211	0.18820884	-2.409593701	0.043826973	0.17733673	uncharacterized protein MGG_06464 [Pyricularia oryzae 70-15]
MGG_06469	2346.689707	638.5358387	0.272100669	-1.877787592	0.015875452	0.088914242	potassium transporter 1 [Pyricularia oryzae 70-15]
MGG_06471	583.122951	1183.500843	2.029590572	1.021188723	0.016297407	0.090524125	zinc finger protein [Pyricularia oryzae 70-15]
MGG_06473	462.8190274	79.86994296	0.17257273	-2.534723589	0.041852862	0.172231901	uncharacterized protein MGG_06473 [Pyricularia oryzae 70-15]
MGG_06492	4071.260503	1625.021145	0.399144477	-1.325017046	0.000868579	0.010518152	transcriptional activator protein DAL81 [Pyricularia oryzae 70-15]
MGG_06500	2443.596662	5272.705937	2.157764421	1.109537364	0.030014134	0.138267615	WD repeat-containing protein [Pyricularia oryzae 70-15]
MGG_06519	221.691105	44.58706881	0.201122498	-2.313853622	3.93291E-07	1.61956E-05	WD domain-containing protein [Pyricularia oryzae 70-15]
MGG_06523	56.0742404	22.6517667	0.403960295	-1.307714596	0.013395871	0.079498308	uncharacterized protein MGG_06523 [Pyricularia oryzae 70-15]
MGG_06529	6375.967834	1816.718461	0.284932187	-1.81130949	9.87633E-06	0.000272677	hypothetical protein, variant [Pyricularia oryzae 70-15]
MGG_06530	2058.14082	4188.796384	2.03523313	1.02519406	0.010108503	0.065878096	aspartate aminotransferase [Pyricularia oryzae 70-15]
MGG_06534	270.1226269	109.4793475	0.40529499	-1.302955752	0.002647773	0.024895627	retinol dehydrogenase 12 [Pyricularia oryzae 70-15]
MGG_06538	2708.792883	405.9268057	0.149855239	-2.73835857	0.004941117	0.039438036	Bys1 family protein [Pyricularia oryzae 70-15]
MGG_06539	6.085734588	22.40362923	3.681335245	1.880229136	0.022603286	0.113567682	uncharacterized protein MGG_06539 [Pyricularia oryzae 70-15]
MGG_06548	1642.859751	785.8602896	0.478348982	-1.063864567	0.007627644	0.054074397	carotenoid oxygenase [Pyricularia oryzae 70-15]
MGG_06549	182.7050942	81.84749215	0.447975972	-1.158506742	0.008081404	0.056002796	uncharacterized protein MGG_06549 [Pyricularia oryzae 70-15]
MGG_06552	1156.848701	2458.186948	2.12489926	1.087394445	0.007647907	0.054160558	monooxygenase [Pyricularia oryzae 70-15]
MGG_06553	570.2853629	1538.836105	2.698361567	1.432083675	0.000487144	0.006656718	uncharacterized protein MGG_06553 [Pyricularia oryzae 70-15]
MGG_06554	575.5138628	264.6507621	0.459851238	-1.120760871	0.006705844	0.049281662	uncharacterized protein MGG_06554 [Pyricularia oryzae 70-15]
MGG_06556	1044.649391	175.025231	0.167544472	-2.577384005	0.000320714	0.004815868	uncharacterized protein MGG_06556 [Pyricularia oryzae 70-15]
MGG_06580	18.67182505	85.53997523	4.581232687	2.195735841	2.9208E-05	0.000672005	FAD binding domain-containing protein [Pyricularia oryzae 70-15]
MGG_06586	2014.836106	5364.686151	2.662591828	1.412831282	0.020856311	0.107584914	uncharacterized protein MGG_06586 [Pyricularia oryzae 70-15]
MGG_06590	1427.699184	591.8483784	0.414546975	-1.270392502	0.002147833	0.021264774	acetyl-CoA carboxylase [Pyricularia oryzae 70-15]
MGG_06595	277.5058302	67.85244945	0.244508194	-2.032045279	5.05896E-06	0.000153181	uncharacterized protein MGG_06595 [Pyricularia oryzae 70-15]
MGG_06596	1637.99339	487.2415151	0.297462443	-1.749220567	0.001323326	0.014695927	glucose dehydrogenase short protein [Pyricularia oryzae 70-15]

MGG_06597	1456.025659	113.6366572	0.078045779	-3.679535576	0.001644312	0.017369658	uncharacterized protein MGG_06597 [Pyricularia oryzae 70-15]
MGG_06601	784.5785209	2733.478974	3.484009441	1.800748533	0.01038628	0.066953487	uncharacterized protein MGG_06601 [Pyricularia oryzae 70-15]
MGG_06615	8.696414599	32.18445262	3.700887561	1.887871305	0.044987852	0.180784791	taurine catabolism dioxygenase TauD [Pyricularia oryzae 70-15]
MGG_06616	110.2946287	762.6773473	6.914909241	2.789710316	2.42886E-10	2.21848E-08	high-affinity methionine permease [Pyricularia oryzae 70-15]
MGG_06618	617.0336084	163.4730944	0.264933858	-1.916295866	6.69728E-06	0.000196518	beta-lactamase domain-containing protein [Pyricularia oryzae 70-15]
MGG_06619	704.0195802	154.3118614	0.219186889	-2.189766593	0.005056783	0.040071624	uncharacterized protein MGG_06619 [Pyricularia oryzae 70-15]
MGG_06622	37.57803662	9.942325132	0.264578089	-1.918234503	0.002124122	0.021098803	uncharacterized protein MGG_06622 [Pyricularia oryzae 70-15]
MGG_06643	1933.462386	4613.13449	2.385944781	1.254560655	0.00181027	0.018747788	uncharacterized protein MGG_06643 [Pyricularia oryzae 70-15]
MGG_06644	131.0619593	334.0858194	2.549067793	1.349969743	0.002056228	0.020626798	tartrate transporter [Pyricularia oryzae 70-15]
MGG_06662	4980.690756	780.0048271	0.156605753	-2.674790881	2.36213E-09	1.63065E-07	FAD binding domain-containing protein [Pyricularia oryzae 70-15]
MGG_06678	528.9203924	1065.455797	2.014397275	1.010348236	0.013283416	0.079217939	ribose-phosphate pyrophosphokinase 5 [Pyricularia oryzae 70-15]
MGG_06704	2020.31994	970.6930061	0.480464993	-1.057496776	0.0083293	0.057198607	glycerophosphodiesterase GDE1 [Pyricularia oryzae 70-15]
MGG_06723	1019.817933	447.2991999	0.438606917	-1.18899953	0.003479398	0.030126676	uncharacterized protein MGG_06723 [Pyricularia oryzae 70-15]
MGG_06731	240.1914131	114.3312871	0.476000727	-1.070964319	0.016184262	0.090209383	uncharacterized protein MGG_06731 [Pyricularia oryzae 70-15]
MGG_06738	2784.629395	231.1073849	0.082993947	-3.590850067	3.35365E-16	9.25913E-14	G-protein coupled receptor [Pyricularia oryzae 70-15]
MGG_06739	828.021777	344.2691565	0.41577307	-1.266131777	0.024476383	0.11965356	uncharacterized protein MGG_06739 [Pyricularia oryzae 70-15]
MGG_06740	1.674870836	39.1957774	23.40226873	4.548576494	2.6912E-08	1.47264E-06	inner membrane protein yieG [Pyricularia oryzae 70-15]
MGG_06744	384.978065	799.5204135	2.076794722	1.054358622	0.036887057	0.157838666	uncharacterized protein MGG_06744 [Pyricularia oryzae 70-15]
MGG_06747	818.0688359	2025.368858	2.475792707	1.307890526	0.018500318	0.09896163	glutathione S-transferase [Pyricularia oryzae 70-15]
MGG_06750	427.3922003	192.1961941	0.449695137	-1.152980812	0.00605884	0.045915653	uncharacterized protein MGG_06750 [Pyricularia oryzae 70-15]
MGG_06755	646.8842054	281.2387252	0.434758992	-1.201712227	0.003373031	0.029457642	uncharacterized protein MGG_06755 [Pyricularia oryzae 70-15]
MGG_06757	605.8268697	1534.237754	2.532468978	1.340544597	0.00111134	0.012845439	ATP-dependent Clp protease [Pyricularia oryzae 70-15]
MGG_06773	1158.605774	424.066648	0.366014616	-1.450026832	0.000463953	0.006411947	uncharacterized protein MGG_06773 [Pyricularia oryzae 70-15]
MGG_06775	2016.836976	1007.565242	0.499576938	-1.001221214	0.013313976	0.079244576	uncharacterized protein MGG_06775 [Pyricularia oryzae 70-15]
MGG_06785	585.5153693	1175.277845	2.00725362	1.005222915	0.026775314	0.12794472	exosome complex exonuclease RRP41
MGG_06794	111.0854622	248.7761461	2.239502282	1.163178136	0.007606394	0.054019894	puromycin resistance protein pur8 [Pyricularia oryzae 70-15]
MGG_06798	142.3449687	58.58323424	0.411558166	-1.280831753	0.011987681	0.073697542	uncharacterized protein MGG_06798 [Pyricularia oryzae 70-15]
MGG_06800	671.3089253	2972.494593	4.427908644	2.146625457	6.416E-05	0.001292562	uncharacterized protein MGG_06800 [Pyricularia oryzae 70-15]
MGG_06821	994.4584666	462.5868581	0.465164583	-1.104186838	0.008229584	0.056715821	mitochondrial carrier protein [Pyricularia oryzae 70-15]
MGG_06828	4440.375473	854.2856644	0.192390412	-2.377891196	0.000113666	0.00210811	uncharacterized protein MGG_06828 [Pyricularia oryzae 70-15]
MGG_06832	305.2817932	104.4289792	0.342074049	-1.547619433	0.000309233	0.004695698	uncharacterized protein MGG_06832 [Pyricularia oryzae 70-15]
MGG_06842	3.166547981	141.9276854	44.82094894	5.486101289	1.52825E-12	2.13392E-10	uncharacterized protein MGG_06842 [Pyricularia oryzae 70-15]
MGG_06845	1.693248103	11.75184487	6.940415201	2.795021973	0.010463741	0.067327079	uncharacterized protein MGG_06845 [Pyricularia oryzae 70-15]
MGG_06848	1523.345809	695.0701582	0.456278643	-1.132012968	0.005193614	0.040995467	uncharacterized protein MGG_06848 [Pyricularia oryzae 70-15]
MGG_06856	581.319242	1893.365013	3.257014178	1.70355	0.00104725	0.012220936	uncharacterized protein MGG_06856 [Pyricularia oryzae 70-15]
MGG_06859	1115.876081	3126.229843	2.801592306	1.486247027	0.000303892	0.004626164	mago nashi like 2 [Pyricularia oryzae 70-15]
MGG_06861	240.3572857	84.20166871	0.35031877	-1.513259805	0.011961799	0.073584379	uncharacterized protein MGG_06861 [Pyricularia oryzae 70-15]
MGG_06888	4461.686171	976.0084506	0.218753272	-2.192623496	0.029243063	0.135486169	glutamine synthetase [Pyricularia oryzae 70-15]
MGG_06912	1210.918273	582.1838485	0.480778812	-1.056554777	0.009227236	0.061581143	uncharacterized protein MGG_06912 [Pyricularia oryzae 70-15]
MGG_06934	318.025821	111.9453582	0.352000846	-1.506349197	0.00056893	0.007479824	uncharacterized protein MGG_06934 [Pyricularia oryzae 70-15]
MGG_06954	8536.568296	4184.37651	0.4901708	-1.028643551	0.020227758	0.105085797	uncharacterized protein MGG_06954 [Pyricularia oryzae 70-15]
MGG_06986	213.0412063	101.6722687	0.477242269	-1.067206268	0.015080719	0.085813701	uncharacterized protein MGG_06986 [Pyricularia oryzae 70-15]
MGG_07016	338.6912941	144.7789817	0.427465909	-1.226118729	0.006681747	0.04920197	uncharacterized protein MGG_07016 [Pyricularia oryzae 70-15]
MGG_07018	640.1078403	1463.26021	2.285958893	1.192799461	0.020086886	0.104629142	uncharacterized protein MGG_07018 [Pyricularia oryzae 70-15]
MGG_07019	630.5614507	132.3507263	0.209893463	-2.252270862	4.84567E-05	0.001018429	4-coumaryl-CoA ligase [Pyricularia oryzae 70-15]
MGG_07022	13965.21356	3906.261152	0.279713671	-1.837977327	7.39061E-06	0.000213257	uncharacterized protein MGG_07022 [Pyricularia oryzae 70-15]
MGG_07038	417.0468137	860.5881285	2.063528842	1.045113603	0.011375933	0.071124467	uncharacterized protein MGG_07038 [Pyricularia oryzae 70-15]
MGG_07055	106.5558144	268.3759318	2.518641834	1.332645977	0.0187531	0.09971914	uncharacterized protein MGG_07055 [Pyricularia oryzae 70-15]

MGG_07056	847.5205863	1801.668973	2.125811458	1.088013647	0.007724608	0.054462295	uncharacterized protein MGG_07056 [Pyricularia oryzae 70-15]
MGG_07067	385.0923437	153.5096165	0.398630663	-1.326875406	0.003367464	0.02943018	FAD binding domain-containing protein [Pyricularia oryzae 70-15]
MGG_07079	222.0094685	108.9399589	0.490699607	-1.027087979	0.017392643	0.094789514	nudix hydrolase 20 [Pyricularia oryzae 70-15]
MGG_07118	22.74588458	4.472966932	0.196649505	-2.346301544	0.007687676	0.054328036	sporulation-specific protein 5 [Pyricularia oryzae 70-15]
MGG_07121	471.7338691	227.8234025	0.482949005	-1.050057235	0.011956365	0.073584379	DNA mismatch repair protein mutL [Pyricularia oryzae 70-15]
MGG_07124	842.1967959	1820.887544	2.162068952	1.112412534	0.005567375	0.043132953	replication protein A 32 kDa subunit [Pyricularia oryzae 70-15]
MGG_07127	1004.028681	434.1174269	0.432375524	-1.209643238	0.002853708	0.02636262	uncharacterized protein MGG_07127 [Pyricularia oryzae 70-15]
MGG_07130	3382.472099	7662.572426	2.265376388	1.179750771	0.003493744	0.030186346	proteasome component PUP2 [Pyricularia oryzae 70-15]
MGG_07131	1160.337386	403.2291961	0.347510303	-1.524872345	0.001129178	0.012977531	uncharacterized protein MGG_07131 [Pyricularia oryzae 70-15]
MGG_07153	892.0044438	270.1668282	0.302876101	-1.72320035	4.18282E-05	0.000902539	uncharacterized protein MGG_07153 [Pyricularia oryzae 70-15]
MGG_07156	4783.445116	2051.085386	0.428788318	-1.221662493	0.002317229	0.022573931	uncharacterized protein MGG_07156 [Pyricularia oryzae 70-15]
MGG_07158	617.4033999	268.1006328	0.434238996	-1.203438803	0.003144223	0.02818895	uncharacterized protein MGG_07158 [Pyricularia oryzae 70-15]
MGG_07165	2176.727544	4666.447893	2.143790529	1.100163946	0.006052802	0.045898524	proteasome subunit alpha type-2 [Pyricularia oryzae 70-15]
MGG_07179	1306.759761	609.5942451	0.466492972	-1.100072748	0.005874752	0.045026173	rhizopuspepsin-3 [Pyricularia oryzae 70-15]
MGG_07187	11567.46706	4422.130523	0.382290306	-1.387259478	0.000494572	0.006727951	glutamate synthase [Pyricularia oryzae 70-15]
MGG_07195	3609.707846	16458.47104	4.559502247	2.188876337	3.0672E-06	9.75403E-05	O-acetylhomoserine (thiol)-lyase [Pyricularia oryzae 70-15]
MGG_07208	1025.384058	497.0769168	0.48477145	-1.044623359	0.010173672	0.066126149	uncharacterized protein MGG_07208 [Pyricularia oryzae 70-15]
MGG_07214	25.03108065	106.1808031	4.241958412	2.084730477	1.78063E-05	0.000442354	uncharacterized protein MGG_07214 [Pyricularia oryzae 70-15]
MGG_07221	439.4772685	1156.21686	2.630891157	1.395551564	0.00524351	0.041282023	uncharacterized protein MGG_07221 [Pyricularia oryzae 70-15]
MGG_07224	2956.964396	1448.060603	0.489711883	-1.029994891	0.010815696	0.068736853	threonine dehydratase [Pyricularia oryzae 70-15]
MGG_07229	1772.101909	4093.713262	2.310089076	1.207948482	0.003104198	0.027948863	glycylpeptide N-tetradecanoyltransferase
MGG_07231	2706.30578	686.5707162	0.253692957	-1.978844626	0.021934932	0.111212671	uncharacterized protein MGG_07231 [Pyricularia oryzae 70-15]
MGG_07233	1492.277413	736.0756033	0.493256547	-1.019589896	0.011770882	0.072843951	uncharacterized protein MGG_07233 [Pyricularia oryzae 70-15]
MGG_07242	1254.708858	7265.435627	5.790535057	2.533696662	6.65041E-08	3.24455E-06	uncharacterized protein MGG_07242 [Pyricularia oryzae 70-15]
MGG_07246	18.82366662	5.60607609	0.297820621	-1.747484444	0.028379266	0.132718752	uncharacterized protein MGG_07246 [Pyricularia oryzae 70-15]
MGG_07247	436.8965213	132.1728887	0.302526759	-1.724865338	4.494E-05	0.000961147	cytochrome P450 [Pyricularia oryzae 70-15]
MGG_07249	1194.498771	95.38355844	0.079852371	-3.646520953	4.95645E-16	1.30893E-13	uncharacterized protein MGG_07249 [Pyricularia oryzae 70-15]
MGG_07266	881.9790685	1954.925195	2.216521077	1.148297081	0.004810092	0.038697345	MYG1 protein [Pyricularia oryzae 70-15]
MGG_07267	525.4731394	1160.404258	2.208303662	1.14293857	0.004664016	0.037873308	uncharacterized protein MGG_07267 [Pyricularia oryzae 70-15]
MGG_07270	4637.050115	1464.204429	0.315762045	-1.663090325	3.7297E-05	0.000825508	fatty aldehyde dehydrogenase [Pyricularia oryzae 70-15]
MGG_07273	3130.477228	6512.203146	2.08025891	1.056763098	0.008123933	0.056169345	WD repeat-containing protein 2 [Pyricularia oryzae 70-15]
MGG_07291	5822.512129	2797.911137	0.480533329	-1.057291597	0.007942934	0.055454459	CMGC/CDK/CRK7 protein kinase [Pyricularia oryzae 70-15]
MGG_07303	65.70334616	647.8356116	9.860009412	3.301589024	2.07641E-13	3.55271E-11	uncharacterized protein MGG_07303 [Pyricularia oryzae 70-15]
MGG_07304	7.56266793	241.9054481	31.98678699	4.999404179	1.33183E-19	6.2227E-17	uncharacterized protein MGG_07304 [Pyricularia oryzae 70-15]
MGG_07311	53.78562081	10.27271173	0.190993644	-2.388403464	6.36126E-05	0.001285799	uncharacterized protein MGG_07311 [Pyricularia oryzae 70-15]
MGG_07317	849.825254	372.997876	0.438911264	-1.187998801	0.0039256	0.033139808	glutamate-cysteine ligase [Pyricularia oryzae 70-15]
MGG_07320	289.3783247	92.48442092	0.319596919	-1.645674596	0.006474531	0.048342105	uncharacterized protein MGG_07320 [Pyricularia oryzae 70-15]
MGG_07325	594.8659969	1269.70833	2.134444289	1.093860508	0.019995076	0.104338062	uncharacterized protein MGG_07325 [Pyricularia oryzae 70-15]
MGG_07326	455.4103261	2279.229394	5.004781981	2.323307223	0.000349193	0.005140179	uncharacterized protein MGG_07326 [Pyricularia oryzae 70-15]
MGG_07334	3564.689116	1141.77878	0.320302484	-1.642493107	0.000273974	0.004277944	uncharacterized protein MGG_07334 [Pyricularia oryzae 70-15]
MGG_07336	482.1008466	1251.432321	2.595789511	1.376173402	0.011536558	0.07178914	uncharacterized protein MGG_07336 [Pyricularia oryzae 70-15]
MGG_07339	11409.817	3841.17541	0.336655304	-1.5706559	0.000114947	0.002118929	uncharacterized protein MGG_07339 [Pyricularia oryzae 70-15]
MGG_07341	18673.49655	40153.05475	2.15026975	1.104517656	0.025423148	0.123093028	uncharacterized protein MGG_07341 [Pyricularia oryzae 70-15]
MGG_07345	4.717295892	28.7186869	6.08795538	2.605957785	0.000483124	0.006624139	uncharacterized protein MGG_07345 [Pyricularia oryzae 70-15]
MGG_07346	1440.323882	681.3306287	0.473039875	-1.079966294	0.007237618	0.052241582	uncharacterized protein MGG_07346 [Pyricularia oryzae 70-15]
MGG_07347	1237.06564	492.0216484	0.397732855	-1.330128354	0.00135893	0.014980297	uncharacterized protein MGG_07347 [Pyricularia oryzae 70-15]
MGG_07349	153.3919691	62.02802069	0.404375933	-1.306230957	0.003971719	0.033413045	uncharacterized protein MGG_07349 [Pyricularia oryzae 70-15]
MGG_07355	12101.45931	3978.706781	0.32877909	-1.604809547	5.94304E-05	0.001215422	uncharacterized protein MGG_07355 [Pyricularia oryzae 70-15]

MGG_07375	26.37159075	179.2264826	6.796195358	2.764727324	0.000903447	0.010844936	ABC transporter CDR4 [Pyricularia oryzae 70-15]
MGG_07376	130.4828271	1123.137071	8.607547028	3.105602158	1.85721E-12	2.50682E-10	uncharacterized protein MGG_07376 [Pyricularia oryzae 70-15]
MGG_07377	0.339553209	16.29169089	47.97978776	5.584354871	2.424E-05	0.000567374	uncharacterized protein MGG_07377 [Pyricularia oryzae 70-15]
MGG_07384	2593.454576	5504.842545	2.122590693	1.085826198	0.006107832	0.046190425	cystathionine beta-synthase [Pyricularia oryzae 70-15]
MGG_07386	6157.128077	1738.823381	0.282408188	-1.824146179	7.27261E-06	0.000210352	C6 zinc finger domain-containing protein
MGG_07388	79.62698779	205.8675252	2.585398882	1.37038688	0.002375788	0.022942031	uncharacterized protein MGG_07388 [Pyricularia oryzae 70-15]
MGG_07394	520.0820251	1071.800674	2.060830065	1.043225546	0.010553754	0.067682314	uncharacterized protein MGG_07394 [Pyricularia oryzae 70-15]
MGG_07398	0	10.95731349	Inf	Inf	0.002289663	0.022383658	uncharacterized protein MGG_07398 [Pyricularia oryzae 70-15]
MGG_07411	1.390449427	12.57816546	9.046115029	3.177298341	0.024359882	0.119309376	uncharacterized protein MGG_07411 [Pyricularia oryzae 70-15]
MGG_07413	6.598255626	120.2889657	18.23041914	4.188275826	1.97414E-06	6.85194E-05	uncharacterized protein MGG_07413 [Pyricularia oryzae 70-15]
MGG_07414	21.78687469	175.5551216	8.057838679	3.010392923	1.86316E-05	0.000460906	uncharacterized protein MGG_07414 [Pyricularia oryzae 70-15]
MGG_07419	41.00885979	239.8387076	5.848460767	2.548056977	6.10141E-08	3.0377E-06	uncharacterized protein MGG_07419 [Pyricularia oryzae 70-15]
MGG_07428	5.328295661	172.1243827	32.30383478	5.013633532	4.55515E-18	1.62753E-15	allantoate permease [Pyricularia oryzae 70-15]
MGG_07429	14.98372015	323.5753866	21.59513015	4.432634106	2.99897E-18	1.17521E-15	uncharacterized protein MGG_07429, partial
MGG_07443	86.18765527	462.0583275	5.361073185	2.42252183	3.38666E-05	0.000761873	uncharacterized protein MGG_07443 [Pyricularia oryzae 70-15]
MGG_07457	1062.103562	462.6255851	0.435574836	-1.199007485	0.002839505	0.026306389	uncharacterized protein MGG_07457 [Pyricularia oryzae 70-15]
MGG_07476	5064.16284	2035.237148	0.401890147	-1.315126887	0.048263951	0.188342589	uncharacterized protein MGG_07476 [Pyricularia oryzae 70-15]
MGG_07496	734.2319387	1820.293792	2.479180892	1.30986354	0.001457872	0.015756428	N-terminal acetyltransferase A complex catalytic subunit ard1
MGG_07502	1279.394138	2692.262805	2.104326357	1.073358468	0.007537109	0.053701346	chaperone dnaJ 2 [Pyricularia oryzae 70-15]
MGG_07510	166.008546	53.66683447	0.323277541	-1.629154814	0.004010991	0.033626998	uncharacterized protein MGG_07510 [Pyricularia oryzae 70-15]
MGG_07512	1349.731642	501.525688	0.371574373	-1.428277092	0.001485764	0.016015143	glutaminase GtaA [Pyricularia oryzae 70-15]
MGG_07514	460.0812521	144.0979112	0.313201006	-1.674839247	0.008046384	0.0557968	3-oxoacyl-[acyl-carrier-protein] reductase
MGG_07515	559.6401559	1323.746129	2.365352298	1.242055076	0.003041589	0.027553484	N-acetyltransferase 5 [Pyricularia oryzae 70-15]
MGG_07533	257.0057017	93.32267419	0.36311519	-1.461500812	0.000848177	0.010355437	uncharacterized protein MGG_07533 [Pyricularia oryzae 70-15]
MGG_07534	565.2106393	186.7739689	0.3304502	-1.597495225	0.000150111	0.002601358	uncharacterized protein MGG_07534 [Pyricularia oryzae 70-15]
MGG_07541	264.8736118	629.858641	2.377959196	1.24972396	0.003430269	0.029786208	uncharacterized protein MGG_07541 [Pyricularia oryzae 70-15]
MGG_07542	1810.101179	6246.055698	3.45066661	1.786875093	0.001435932	0.015597758	uncharacterized protein MGG_07542 [Pyricularia oryzae 70-15]
MGG_07548	6.365332554	262.0744808	41.17215851	5.363597181	1.02109E-21	6.20213E-19	uncharacterized protein MGG_07548 [Pyricularia oryzae 70-15]
MGG_07551	26.89797111	142.8075825	5.309232503	2.408503322	1.30486E-06	4.74595E-05	cytochrome P450 monooxygenase [Pyricularia oryzae 70-15]
MGG_07553	237.3202631	77.86872212	0.328116618	-1.607719431	0.00584493	0.044825891	CFEM domain-containing protein [Pyricularia oryzae 70-15]
MGG_07556	7.681388437	480.8604022	62.6007142	5.968107211	9.98175E-17	3.03146E-14	uncharacterized protein MGG_07556 [Pyricularia oryzae 70-15]
MGG_07557	47.02590875	237.5995614	5.052524614	2.337004445	4.40908E-07	1.78538E-05	uncharacterized protein MGG_07557 [Pyricularia oryzae 70-15]
MGG_07558	19.9254822	296.2402444	14.86740654	3.894081101	4.86537E-15	1.03692E-12	uncharacterized protein MGG_07558 [Pyricularia oryzae 70-15]
MGG_07559	169.5000625	539.7401663	3.184306592	1.670979248	7.76848E-05	0.001509944	tripeptidyl-peptidase 1 [Pyricularia oryzae 70-15]
MGG_07560	82.15325834	181.9640509	2.21493407	1.147263756	0.010852064	0.068807974	uncharacterized protein MGG_07560 [Pyricularia oryzae 70-15]
MGG_07565	18.97914166	65.25557721	3.438278631	1.781686462	0.000936311	0.011173191	uncharacterized protein MGG_07565 [Pyricularia oryzae 70-15]
MGG_07569	3.726049414	112.9201821	30.30560509	4.921512743	5.91116E-15	1.2171E-12	cellobiose dehydrogenase [Pyricularia oryzae 70-15]
MGG_07571	92.57536758	433.9586499	4.687625458	2.228857303	2.16647E-06	7.35149E-05	LysM domain-containing protein [Pyricularia oryzae 70-15]
MGG_07572	2.725767054	110.0064563	40.35798149	5.334782114	5.53413E-16	1.4304E-13	high-affinity nickel transporter nic1 [Pyricularia oryzae 70-15]
MGG_07574	11.32006946	75.15155114	6.638788871	2.730920072	2.10818E-06	7.21413E-05	uncharacterized protein MGG_07574 [Pyricularia oryzae 70-15]
MGG_07580	74.06088208	176.6510103	2.385213426	1.254118363	0.006047428	0.045898524	glucose oxidase [Pyricularia oryzae 70-15]
MGG_07584	2.422968379	952.4111934	393.076196	8.618665189	1.38931E-42	4.21934E-39	uncharacterized protein MGG_07584 [Pyricularia oryzae 70-15]
MGG_07585	2.065037904	188.0189648	91.04867491	6.508566116	5.14822E-23	3.55048E-20	uncharacterized protein MGG_07585 [Pyricularia oryzae 70-15]
MGG_07597	82.47235982	179.423615	2.17556058	1.12138719	0.014290575	0.083023391	uncharacterized protein MGG_07597 [Pyricularia oryzae 70-15]
MGG_07598	396.3257317	48.28335648	0.121827458	-3.037088763	0.014655568	0.084463938	uncharacterized protein MGG_07598 [Pyricularia oryzae 70-15]
MGG_07606	670.633159	3192.714891	4.760747136	2.251188003	6.08127E-08	3.0377E-06	hypothetical protein, variant [Pyricularia oryzae 70-15]
MGG_07614	240.0723362	92.12176746	0.383725043	-1.381855173	0.00161714	0.0171273	uncharacterized protein MGG_07614 [Pyricularia oryzae 70-15]
MGG_07620	10.49904182	443.9780023	42.28747821	5.402158623	2.86613E-10	2.57909E-08	L-amino-acid oxidase [Pyricularia oryzae 70-15]

MGG_07621	9.398416224	385.7001666	41.03884712	5.358918298	8.00535E-05	0.001548551	endoribonuclease L-PSP [Pyricularia oryzae 70-15]
MGG_07625	1.050896218	10.52129004	10.0117308	3.3236195	0.006426724	0.048192495	uncharacterized protein MGG_07625 [Pyricularia oryzae 70-15]
MGG_07627	876.6074254	312.4493189	0.356430153	-1.488308704	0.00025521	0.004047384	homoserine acetyltransferase [Pyricularia oryzae 70-15]
MGG_07630	2039.651596	6134.598033	3.007669567	1.588646076	0.027061539	0.128767558	uncharacterized protein MGG_07630 [Pyricularia oryzae 70-15]
MGG_07631	732.6714798	2620.562494	3.5767224	1.838638151	9.48707E-06	0.000264332	fungal cellulose binding domain-containing protein
MGG_07632	18.04333249	89.1424483	4.94046476	2.304646765	3.95655E-05	0.000862912	endonuclease/exonuclease/phosphatase
MGG_07639	64546.52993	15766.27606	0.244262179	-2.033497597	7.13687E-07	2.78774E-05	excitatory amino acid transporter 2 [Pyricularia oryzae 70-15]
MGG_07655	1149.335833	552.6119958	0.480809856	-1.056461626	0.008964344	0.060398698	uncharacterized protein MGG_07655 [Pyricularia oryzae 70-15]
MGG_07661	5072.792227	1874.848776	0.369589112	-1.436005838	0.001792265	0.018609888	potassium:hydrogen antiporter [Pyricularia oryzae 70-15]
MGG_07665	5345.097621	11911.07551	2.228411221	1.156015485	0.003949531	0.033272467	uncharacterized protein MGG_07665 [Pyricularia oryzae 70-15]
MGG_07669	11.30346115	124.5472478	11.01850541	3.46185664	0.015665809	0.087942812	uncharacterized protein MGG_07669 [Pyricularia oryzae 70-15]
MGG_07676	23.1929524	722.6766791	31.15932231	4.961591951	1.64614E-23	1.42838E-20	uncharacterized protein MGG_07676 [Pyricularia oryzae 70-15]
MGG_07681	2807.263402	1328.514726	0.473241921	-1.079350217	0.006871019	0.050252344	uncharacterized protein MGG_07681 [Pyricularia oryzae 70-15]
MGG_07686	747.9688751	179.6208315	0.240144794	-2.058023561	0.037068511	0.158169396	endoglucanase II [Pyricularia oryzae 70-15]
MGG_07690	21.08939101	225.5979893	10.69722635	3.419164869	6.12862E-12	7.1587E-10	uncharacterized protein MGG_07690 [Pyricularia oryzae 70-15]
MGG_07691	291.0756897	1698.803268	5.836293886	2.545052532	2.36248E-09	1.63065E-07	general alpha-glucoside permease [Pyricularia oryzae 70-15]
MGG_07697	111.2410648	880.1638883	7.912220993	2.984082722	0.000635203	0.008200262	superoxide dismutase [Pyricularia oryzae 70-15]
MGG_07703	1087.854474	341.7571829	0.314157078	-1.67044201	6.76738E-05	0.001341112	uncharacterized protein MGG_07703 [Pyricularia oryzae 70-15]
MGG_07707	637.2964926	226.5645897	0.355508923	-1.492042325	0.000426467	0.00596857	uncharacterized protein MGG_07707 [Pyricularia oryzae 70-15]
MGG_07710	119.5887948	56.22783505	0.470176451	-1.088725813	0.018167175	0.097782382	uncharacterized protein MGG_07710 [Pyricularia oryzae 70-15]
MGG_07711	2630.644781	7539.201584	2.865913954	1.518995295	0.000179224	0.003007198	uncharacterized protein MGG_07711 [Pyricularia oryzae 70-15]
MGG_07713	85.56472421	23.70106315	0.276995729	-1.852064364	0.00028371	0.004407303	uncharacterized protein MGG_07713 [Pyricularia oryzae 70-15]
MGG_07715	681.754744	289.5901199	0.424771698	-1.235240452	0.00287846	0.026490556	uncharacterized protein MGG_07715 [Pyricularia oryzae 70-15]
MGG_07724	611.1644876	97.51002553	0.159547925	-2.647938254	0.016030723	0.089536193	uncharacterized protein MGG_07724 [Pyricularia oryzae 70-15]
MGG_07727	3415.431746	9951.709239	2.913748533	1.542876373	0.001756314	0.018298203	proteasome component C1 [Pyricularia oryzae 70-15]
MGG_07728	989.9113909	404.3343469	0.408455091	-1.291750631	0.017788657	0.096372795	uncharacterized protein MGG_07728 [Pyricularia oryzae 70-15]
MGG_07732	153.8415439	70.40573651	0.457651001	-1.12768026	0.011940996	0.073584379	uncharacterized protein MGG_07732 [Pyricularia oryzae 70-15]
MGG_07738	932.4415078	312.9805163	0.335656997	-1.574940379	0.000117338	0.002140282	uncharacterized protein MGG_07738 [Pyricularia oryzae 70-15]
MGG_07746	251.1338384	54.727245	0.217920633	-2.198125297	1.20447E-06	4.42052E-05	uncharacterized protein MGG_07746 [Pyricularia oryzae 70-15]
MGG_07747	3187.81836	821.0046404	0.25754436	-1.957107146	2.18643E-06	7.35766E-05	uncharacterized protein MGG_07747 [Pyricularia oryzae 70-15]
MGG_07762	3166.326003	931.5406678	0.294202387	-1.765119144	0.000159384	0.002730891	uncharacterized protein MGG_07762 [Pyricularia oryzae 70-15]
MGG_07767	147.6074233	25.88098668	0.17533662	-2.511800753	0.018234428	0.097927425	uncharacterized protein MGG_07767 [Pyricularia oryzae 70-15]
MGG_07776	109.6300435	30.06736253	0.274262069	-1.866372988	0.047300855	0.186319971	uncharacterized protein MGG_07776 [Pyricularia oryzae 70-15]
MGG_07779	2682.452744	543.0267824	0.202436663	-2.304457496	1.62353E-06	5.73333E-05	quinate permease [Pyricularia oryzae 70-15]
MGG_07780	871.4929816	415.6289112	0.476915959	-1.068193034	0.024033956	0.118300038	3-dehydroshikimate dehydratase [Pyricularia oryzae 70-15]
MGG_07781	1124.851495	373.7801969	0.332292928	-1.589472506	0.000546979	0.007270124	quinate dehydrogenase [Pyricularia oryzae 70-15]
MGG_07787	1444.131769	3382.598885	2.342306261	1.227929723	0.002410339	0.023201897	uncharacterized protein MGG_07787 [Pyricularia oryzae 70-15]
MGG_07792	1036.753991	404.5834704	0.390240572	-1.357564317	0.001127106	0.012977531	uncharacterized protein MGG_07792 [Pyricularia oryzae 70-15]
MGG_07803	0.660729151	8.120872713	12.29077407	3.619503874	0.012865449	0.077533618	D-alanine-poly(phosphoribitol) ligase subunit 1
MGG_07809	0	10.91729629	Inf	Inf	0.000174957	0.002949713	exoglucanase 1 [Pyricularia oryzae 70-15]
MGG_07814	9.328540622	0	0	-Inf	0.000394016	0.005625899	uncharacterized protein MGG_07814 [Pyricularia oryzae 70-15]
MGG_07815	712.3696005	0.321680965	0.000451565	-11.11277967	9.92137E-45	4.0175E-41	uncharacterized protein MGG_07815 [Pyricularia oryzae 70-15]
MGG_07816	839.7512706	0	0	-Inf	5.93861E-06	0.000176878	uncharacterized protein MGG_07816 [Pyricularia oryzae 70-15]
MGG_07817	169.9210662	0	0	-Inf	5.26083E-23	3.55048E-20	uncharacterized protein MGG_07817 [Pyricularia oryzae 70-15]
MGG_07818	33.33198929	0.321680965	0.009650818	-6.695133041	1.38429E-07	6.29824E-06	uncharacterized protein MGG_07818 [Pyricularia oryzae 70-15]
MGG_07819	60.55409528	126.3074304	2.085861077	1.060643074	0.02306318	0.115012933	uncharacterized protein MGG_07819 [Pyricularia oryzae 70-15]
MGG_07824	3.776663273	0	0	-Inf	0.046205841	0.183494133	spherulin-1B [Pyricularia oryzae 70-15]
MGG_07830	40.31158605	13.42590615	0.333053285	-1.586175083	0.016368032	0.090693877	uncharacterized protein MGG_07830 [Pyricularia oryzae 70-15]

MGG_07833	730.693581	228.1654439	0.312258722	-1.679186224	0.016157037	0.090158792	ferric reductase [Pyricularia oryzae 70-15]
MGG_07842	718.6411704	228.0558658	0.317343168	-1.655884311	0.01341152	0.079552315	uncharacterized protein MGG_07842 [Pyricularia oryzae 70-15]
MGG_07844	72.32132812	292.8728165	4.049605063	2.017781216	8.13732E-06	0.000231504	maltose permease [Pyricularia oryzae 70-15]
MGG_07845	58.21672634	161.3866077	2.772169062	1.471015244	0.001444063	0.015621085	C6 zinc finger domain-containing protein
MGG_07846	123.2284558	693.1241421	5.624708494	2.491778329	1.33822E-08	8.08793E-07	endoglucanase family 5 glycoside hydrolase
MGG_07847	0.339553209	17.67960192	52.06725035	5.702304315	9.44955E-06	0.000263892	uncharacterized protein MGG_07847 [Pyricularia oryzae 70-15]
MGG_07848	6.718745088	31.789669	4.731489077	2.242294295	0.01140106	0.07117943	multidrug resistance protein CDR1 [Pyricularia oryzae 70-15]
MGG_07850	9.495126001	39.7829703	4.189830687	2.066891945	0.000810488	0.009945256	uncharacterized protein MGG_07850 [Pyricularia oryzae 70-15]
MGG_07851	0	51.3378826	Inf	Inf	4.18424E-14	7.9422E-12	uncharacterized protein MGG_07851 [Pyricularia oryzae 70-15]
MGG_07854	0	12.91177911	Inf	Inf	4.20518E-05	0.000904763	uncharacterized protein MGG_07854 [Pyricularia oryzae 70-15]
MGG_07855	1.353694894	16.94001695	12.51391065	3.645460803	0.000380721	0.005473367	uncharacterized protein MGG_07855 [Pyricularia oryzae 70-15]
MGG_07857	2.101792437	14.29594864	6.801788982	2.765914249	0.005370162	0.042006907	uncharacterized protein MGG_07857 [Pyricularia oryzae 70-15]
MGG_07861	12.5502204	33.91879167	2.702645099	1.434372075	0.025303279	0.122561495	carboxypeptidase S1 [Pyricularia oryzae 70-15]
MGG_07871	203.4943084	475.9429744	2.338851528	1.225800282	0.003167972	0.028276654	uncharacterized protein MGG_07871 [Pyricularia oryzae 70-15]
MGG_07877	297.5259477	2680.262266	9.008499213	3.171286778	3.01614E-13	4.75844E-11	dipeptidyl-peptidase V [Pyricularia oryzae 70-15]
MGG_07880	1.605879711	29.04284993	18.08532092	4.176747294	4.30165E-05	0.000923258	uncharacterized protein MGG_07880 [Pyricularia oryzae 70-15]
MGG_07883	99.1788792	211.1094841	2.128572997	1.089886566	0.015124992	0.085950062	sorbose reductase sou1 [Pyricularia oryzae 70-15]
MGG_07887	54.02638979	243.4252865	4.505673753	2.171742855	2.19252E-06	7.35766E-05	uncharacterized protein MGG_07887 [Pyricularia oryzae 70-15]
MGG_07890	120.1952131	289.8747328	2.41169948	1.270050145	0.005437619	0.042343712	aldehyde dehydrogenase 3I1 [Pyricularia oryzae 70-15]
MGG_07900	0	16.8408342	Inf	Inf	0.001413855	0.015445603	uncharacterized protein MGG_07900 [Pyricularia oryzae 70-15]
MGG_07903	2.115651762	14.62633524	6.913394492	2.789394251	0.010926024	0.069238051	uncharacterized protein MGG_07903 [Pyricularia oryzae 70-15]
MGG_07905	1.996046778	10.71121725	5.366215545	2.423905005	0.029937593	0.137967328	uncharacterized protein MGG_07905 [Pyricularia oryzae 70-15]
MGG_07912	1652.890485	3724.832812	2.253526683	1.172184532	0.003465309	0.030026084	erythrocyte band 7 integral membrane protein
MGG_07914	3417.538921	7925.643799	2.31910857	1.213570362	0.004697984	0.038098206	uncharacterized protein MGG_07914 [Pyricularia oryzae 70-15]
MGG_07919	22.26529463	76.76835747	3.447893179	1.785715078	0.014878565	0.085256984	uncharacterized protein MGG_07919 [Pyricularia oryzae 70-15]
MGG_07920	0	17.06184244	Inf	Inf	2.12556E-06	7.25318E-05	arylsulfatase [Pyricularia oryzae 70-15]
MGG_07927	15410.22404	6775.229747	0.439658095	-1.185546063	0.039423009	0.164800658	endochitinase 1 [Pyricularia oryzae 70-15]
MGG_07933	1545.084213	5503.802342	3.562137452	1.832743187	0.001587019	0.016911501	dihydrodipicolinate synthase [Pyricularia oryzae 70-15]
MGG_07935	736.1215139	2847.6671	3.868474221	1.95176466	0.001550812	0.016599299	galactonate dehydratase [Pyricularia oryzae 70-15]
MGG_07937	486.9976325	1686.814822	3.463702305	1.79231494	0.021826054	0.110938452	2-dehydropantoate 2-reductase [Pyricularia oryzae 70-15]
MGG_07949	244.8549897	1270.419793	5.188457846	2.375305793	0.000223107	0.003618567	uncharacterized protein MGG_07949 [Pyricularia oryzae 70-15]
MGG_07957	18.39643952	46.69885382	2.538472392	1.343960569	0.018941562	0.100481266	uridine permease [Pyricularia oryzae 70-15]
MGG_07958	47.17625485	151.0453428	3.201723904	1.678848904	0.001388636	0.015224861	naringenin,2-oxoglutarate 3-dioxygenase
MGG_07961	944.2464566	2111.745205	2.236434344	1.161200405	0.003338409	0.029246257	uncharacterized protein MGG_07961 [Pyricularia oryzae 70-15]
MGG_07964	1783.811159	7191.252896	4.031398088	2.011280252	1.4037E-06	5.03014E-05	uncharacterized protein MGG_07964 [Pyricularia oryzae 70-15]
MGG_07965	620.9638435	1485.086824	2.391583406	1.257966106	0.002248052	0.022077066	alkaline proteinase [Pyricularia oryzae 70-15]
MGG_07966	608.4475331	291.9997205	0.479909449	-1.059165875	0.009156146	0.061350721	phosphate transporter [Pyricularia oryzae 70-15]
MGG_07968	571.3360491	1352.871269	2.367908119	1.243613102	0.002704713	0.025372091	high-affinity nicotinic acid transporter [Pyricularia oryzae 70-15]
MGG_07973	225.4381725	562.412928	2.494754645	1.318897936	0.005830489	0.044743389	surface protein 1 [Pyricularia oryzae 70-15]
MGG_07974	1028.413069	2732.395809	2.656904984	1.409746635	0.001737214	0.018130302	C-signal protein [Pyricularia oryzae 70-15]
MGG_07979	0.339553209	28.41642163	83.68768398	6.386943417	2.13609E-08	1.21258E-06	uncharacterized protein MGG_07979 [Pyricularia oryzae 70-15]
MGG_07982	72.5722274	286.8678744	3.95286027	1.982896958	9.62902E-06	0.000267674	cytochrome P450 monooxygenase [Pyricularia oryzae 70-15]
MGG_07985	147.4149391	36.32128703	0.246388102	-2.020995505	2.163E-05	0.000519282	uncharacterized protein MGG_07985 [Pyricularia oryzae 70-15]
MGG_07995	19.81549208	3.140002302	0.15846199	-2.657791272	0.002008353	0.020314297	uncharacterized protein MGG_07995 [Pyricularia oryzae 70-15]
MGG_07997	37.27465897	117.6092668	3.15520705	1.65773468	0.000562359	0.007425589	uncharacterized protein MGG_07997 [Pyricularia oryzae 70-15]
MGG_08003	152.7974501	45.88698479	0.300312504	-1.735463551	0.000184602	0.003086104	UbiA prenyltransferase [Pyricularia oryzae 70-15]
MGG_08014	1007.702729	400.6429235	0.397580469	-1.330681208	0.001001201	0.011760417	uncharacterized protein MGG_08014 [Pyricularia oryzae 70-15]
MGG_08016	807.7446205	313.4406259	0.388044213	-1.365707054	0.016114825	0.089964567	retinal pigment epithelial membrane family protein

MGG_08019	1527.170274	6483.570421	4.245479716	2.08592758	1.25194E-06	4.56755E-05	F-box domain-containing protein [Pyricularia oryzae 70-15]
MGG_08026	424.5949927	1181.613181	2.782918313	1.476598562	0.018710525	0.099603618	uncharacterized protein MGG_08026 [Pyricularia oryzae 70-15]
MGG_08034	244.3895441	65.29351637	0.267169844	-1.904170918	0.000176412	0.002964118	uncharacterized protein MGG_08034 [Pyricularia oryzae 70-15]
MGG_08043	26.66987148	63.20655202	2.369960877	1.244863244	0.019650645	0.103107564	uncharacterized protein MGG_08043 [Pyricularia oryzae 70-15]
MGG_08053	551.3679648	1191.094836	2.160253972	1.111200934	0.006493958	0.048421146	WD repeat-containing protein JIP5 [Pyricularia oryzae 70-15]
MGG_08058	316.5939721	144.9558106	0.457860299	-1.127020621	0.008253656	0.056775431	transcription factor ACEII [Pyricularia oryzae 70-15]
MGG_08060	290.4131474	129.3304714	0.445332701	-1.167044541	0.006946079	0.050740208	cycloheximide resistance protein [Pyricularia oryzae 70-15]
MGG_08062	2022.055803	4762.139956	2.355098187	1.235787209	0.035751612	0.155019645	uncharacterized protein MGG_08062 [Pyricularia oryzae 70-15]
MGG_08063	4178.71896	8971.132563	2.146861909	1.102229397	0.006047863	0.045898524	pyruvate kinase [Pyricularia oryzae 70-15]
MGG_08069	953.2856821	2870.413564	3.011073824	1.59027808	0.002192264	0.021616573	uncharacterized protein MGG_08069 [Pyricularia oryzae 70-15]
MGG_08072	47699.05508	14370.96688	0.3012841	-1.730803557	0.001559892	0.016666292	cholesterol oxidase [Pyricularia oryzae 70-15]
MGG_08094	824.1755824	253.0917245	0.307084716	-1.703291387	4.09862E-05	0.000889109	fungal specific transcription factor domain-containing protein
MGG_08103	123.8206773	365.9226642	2.955262984	1.563286519	0.000268207	0.004220248	ATP binding/alanine-tRNA ligase [Pyricularia oryzae 70-15]
MGG_08118	83.46833419	39.66933598	0.475262102	-1.07320473	0.025177898	0.122149001	uncharacterized protein MGG_08118 [Pyricularia oryzae 70-15]
MGG_08123	978.8829393	369.5543858	0.377526639	-1.405349648	0.019403052	0.102415546	beta-glucosidase [Pyricularia oryzae 70-15]
MGG_08129	18.6591557	66.63719106	3.571286511	1.83644388	0.000660521	0.008445101	lysine-specific permease [Pyricularia oryzae 70-15]
MGG_08130	969.8171714	185.3282254	0.191096045	-2.387630178	2.03405E-08	1.17665E-06	uncharacterized protein MGG_08130 [Pyricularia oryzae 70-15]
MGG_08136	234.8990321	106.68115	0.454157469	-1.138735487	0.007154241	0.05176279	uncharacterized protein MGG_08136 [Pyricularia oryzae 70-15]
MGG_08139	1283.616157	2934.125302	2.285827649	1.192716629	0.003216182	0.028481764	methionine aminopeptidase 1 [Pyricularia oryzae 70-15]
MGG_08156	309.802488	642.5043517	2.073916048	1.052357495	0.010000917	0.065488968	kinesin-II 85 kDa subunit [Pyricularia oryzae 70-15]
MGG_08158	7.443062946	31.99867095	4.299126741	2.104043643	0.001998318	0.020229642	uncharacterized protein MGG_08158 [Pyricularia oryzae 70-15]
MGG_08161	21.22914221	7.42008231	0.349523416	-1.516538983	0.039528409	0.165184423	uncharacterized protein MGG_08161 [Pyricularia oryzae 70-15]
MGG_08164	5050.719185	13622.87348	2.697214591	1.431470308	0.00042527	0.005958678	protein disulfide-isomerase erp38 [Pyricularia oryzae 70-15]
MGG_08185	4775.334336	2102.87649	0.440362149	-1.183237627	0.00593462	0.045384131	uncharacterized protein MGG_08185 [Pyricularia oryzae 70-15]
MGG_08191	201.3762767	83.44515011	0.414374282	-1.270993629	0.003502051	0.03021514	uncharacterized protein MGG_08191 [Pyricularia oryzae 70-15]
MGG_08194	53.37194753	265.4998884	4.974521274	2.314557694	3.82024E-07	1.5839E-05	uncharacterized protein MGG_08194 [Pyricularia oryzae 70-15]
MGG_08200	2322.989733	419.3927906	0.180540096	-2.469608811	4.23544E-09	2.81159E-07	uncharacterized protein MGG_08200 [Pyricularia oryzae 70-15]
MGG_08208	4775.363195	2209.178891	0.462620077	-1.112100216	0.005828041	0.044743389	uncharacterized protein MGG_08208 [Pyricularia oryzae 70-15]
MGG_08209	1013.31488	2036.092507	2.009338408	1.00672056	0.011495174	0.071594554	uncharacterized protein MGG_08209 [Pyricularia oryzae 70-15]
MGG_08239	0	5.458648147	Inf	Inf	0.012541985	0.076167957	uncharacterized protein MGG_08239 [Pyricularia oryzae 70-15]
MGG_08253	8.728651192	92.42817827	10.58905623	3.404502107	2.22751E-09	1.56415E-07	uncharacterized protein MGG_08253 [Pyricularia oryzae 70-15]
MGG_08254	59.58645057	10.59687477	0.177840342	-2.491345465	0.042840602	0.174640144	uncharacterized protein MGG_08254 [Pyricularia oryzae 70-15]
MGG_08256	5094.353973	22681.5768	4.452296978	2.154549827	1.88549E-07	8.23918E-06	mitochondrial peroxiredoxin PRX1 [Pyricularia oryzae 70-15]
MGG_08275	148.3776968	68.47231324	0.461473083	-1.115681594	0.027709607	0.130778955	uncharacterized protein MGG_08275 [Pyricularia oryzae 70-15]
MGG_08279	606.0155293	285.1189899	0.470481326	-1.087790633	0.007475233	0.053383827	inner membrane protein yicO [Pyricularia oryzae 70-15]
MGG_08281	119.9644915	365.6360565	3.047869015	1.607800903	0.00021399	0.003503442	polyketide synthase [Pyricularia oryzae 70-15]
MGG_08288	61.83812452	267.3901875	4.32403456	2.112378054	2.17897E-06	7.35766E-05	short-chain-fatty-acid-CoA ligase [Pyricularia oryzae 70-15]
MGG_08289	47.38805167	245.4656217	5.179905337	2.372925733	0.001912602	0.019606997	aerobactin siderophore biosynthesis protein iucB
MGG_08290	183.8481822	512.2784553	2.786421106	1.478413306	0.000500075	0.006772471	uncharacterized protein MGG_08290 [Pyricularia oryzae 70-15]
MGG_08297	18.28165798	50.33156307	2.753118077	1.461066486	0.009570615	0.06308753	NADH:flavin oxidoreductase/NADH oxidase
MGG_08305	250.1315771	80.33605534	0.321175184	-1.638567669	0.000174424	0.002947013	uncharacterized protein MGG_08305 [Pyricularia oryzae 70-15]
MGG_08337	448.2978308	7368.306406	16.43618572	4.038803632	2.08539E-19	9.38271E-17	uncharacterized protein MGG_08337 [Pyricularia oryzae 70-15]
MGG_08339	935.2354135	328.2902044	0.351024137	-1.510357857	0.000224778	0.003634799	uncharacterized protein MGG_08339 [Pyricularia oryzae 70-15]
MGG_08340	475.6112127	1393.163045	2.929205637	1.550509477	0.000175069	0.002949713	uncharacterized protein MGG_08340 [Pyricularia oryzae 70-15]
MGG_08341	252.2810245	795.5688875	3.153502683	1.656955161	8.06153E-05	0.001554467	uncharacterized protein MGG_08341 [Pyricularia oryzae 70-15]
MGG_08342	309.3604851	128.9769644	0.416914799	-1.262175511	0.003017031	0.027381434	phosphorylcholine phosphatase [Pyricularia oryzae 70-15]
MGG_08359	27.16578421	562.2219622	20.69595922	4.371277211	1.97614E-15	4.52947E-13	flavonol synthase [Pyricularia oryzae 70-15]
MGG_08360	7.144782211	107.5474878	15.05259147	3.911939979	1.69719E-11	1.89151E-09	uncharacterized protein MGG_08360 [Pyricularia oryzae 70-15]

MGG_08363	9.985636309	99.77193087	9.991544634	3.320707727	2.64552E-09	1.80549E-07	enoyl reductase [Pyricularia oryzae 70-15]
MGG_08366	97.76775545	284.8215416	2.913246195	1.542627627	0.00143467	0.015597758	uncharacterized protein MGG_08366 [Pyricularia oryzae 70-15]
MGG_08369	730.3057879	266.3355965	0.364690519	-1.455255403	0.000498808	0.006766252	uncharacterized protein MGG_08369 [Pyricularia oryzae 70-15]
MGG_08370	123.2683792	454.6788505	3.688527855	1.883045131	0.000113221	0.002103065	1,3-beta-glucanosyltransferase gel3 [Pyricularia oryzae 70-15]
MGG_08371	4.065602623	95.70764786	23.54082697	4.557093097	6.28528E-13	9.19923E-11	uncharacterized protein MGG_08371 [Pyricularia oryzae 70-15]
MGG_08374	1280.394146	2689.474259	2.100504963	1.070736195	0.007950882	0.055478069	uncharacterized protein MGG_08374 [Pyricularia oryzae 70-15]
MGG_08376	23.37407163	299.7593824	12.82444014	3.68082394	6.25587E-10	5.03287E-08	uncharacterized protein MGG_08376 [Pyricularia oryzae 70-15]
MGG_08377	4.707954507	128.9629905	27.39257364	4.775712914	2.89117E-15	6.3858E-13	uncharacterized protein MGG_08377 [Pyricularia oryzae 70-15]
MGG_08408	68.94053983	159.9980717	2.320812575	1.214630017	0.009505263	0.062892119	uncharacterized protein MGG_08408 [Pyricularia oryzae 70-15]
MGG_08409	164.9192854	790.825483	4.795227441	2.261599245	0.003362032	0.029403864	cellulose-growth-specific protein [Pyricularia oryzae 70-15]
MGG_08410	11.64212988	66.40105989	5.703514785	2.511851253	1.01097E-05	0.000277856	uncharacterized protein MGG_08410 [Pyricularia oryzae 70-15]
MGG_08412	30.28623627	259.3399354	8.562963488	3.098110174	9.79261E-11	9.5936E-09	uncharacterized protein MGG_08412 [Pyricularia oryzae 70-15]
MGG_08415	52.16795623	121.3763687	2.326646039	1.218251745	0.008927832	0.060224412	serin endopeptidase [Pyricularia oryzae 70-15]
MGG_08428	1.303081035	26.78315933	20.55371739	4.361327441	0.000382061	0.005486144	uncharacterized protein MGG_08428 [Pyricularia oryzae 70-15]
MGG_08429	12.77587655	93.50093605	7.318553502	2.871558531	1.31896E-07	6.0463E-06	uncharacterized protein MGG_08429 [Pyricularia oryzae 70-15]
MGG_08430	0	141.417483	Inf	Inf	7.04757E-14	1.29718E-11	retinol dehydrogenase 12 [Pyricularia oryzae 70-15]
MGG_08431	0	197.2485668	Inf	Inf	5.28661E-29	8.02772E-26	uncharacterized protein MGG_08431 [Pyricularia oryzae 70-15]
MGG_08432	0	14.46153283	Inf	Inf	1.38041E-05	0.000356791	uncharacterized protein MGG_08432 [Pyricularia oryzae 70-15]
MGG_08433	0	66.98243325	Inf	Inf	8.59528E-14	1.55844E-11	uncharacterized protein MGG_08433 [Pyricularia oryzae 70-15]
MGG_08434	3370.048057	7488.260642	2.222004113	1.151861487	0.01642615	0.090867428	uncharacterized protein MGG_08434 [Pyricularia oryzae 70-15]
MGG_08435	26.99281638	76.54664105	2.835815276	1.503763559	0.015982886	0.089433484	uncharacterized protein MGG_08435 [Pyricularia oryzae 70-15]
MGG_08441	0	25.15088914	Inf	Inf	1.44606E-08	8.65355E-07	uncharacterized protein MGG_08441 [Pyricularia oryzae 70-15]
MGG_08447	111.3156683	385.6635116	3.464593237	1.792685982	3.74037E-05	0.000826147	uncharacterized protein MGG_08447 [Pyricularia oryzae 70-15]
MGG_08450	989.0624522	2822.040418	2.853247954	1.512605126	0.000195219	0.003230957	asparagine synthase [Pyricularia oryzae 70-15]
MGG_08451	184.1353526	560.4161577	3.043501151	1.605731912	0.0001625	0.002776443	uncharacterized protein MGG_08451 [Pyricularia oryzae 70-15]
MGG_08455	2432.294594	1080.08469	0.44405998	-1.171173537	0.003521298	0.030338106	uncharacterized protein MGG_08455 [Pyricularia oryzae 70-15]
MGG_08457	43474.7545	18618.4377	0.428258605	-1.223445861	0.002174281	0.021491592	uncharacterized protein MGG_08457 [Pyricularia oryzae 70-15]
MGG_08464	1607.807547	6098.916887	3.793312762	1.923458328	2.47739E-06	8.20036E-05	aflatoxin B1 aldehyde reductase member 2
MGG_08467	1.353694894	9.781705069	7.225930388	2.853183356	0.038760197	0.163152764	uncharacterized protein MGG_08467 [Pyricularia oryzae 70-15]
MGG_08469	1.353694894	33.38906405	24.66513259	4.624401138	1.1275E-07	5.28838E-06	uncharacterized protein MGG_08469 [Pyricularia oryzae 70-15]
MGG_08480	17.10358435	104.0516699	6.083617786	2.604929516	0.000372635	0.005387752	alpha/beta hydrolase [Pyricularia oryzae 70-15]
MGG_08491	0	6.902250567	Inf	Inf	0.013935123	0.081621924	uncharacterized protein MGG_08491 [Pyricularia oryzae 70-15]
MGG_08493	0	5.45616608	Inf	Inf	0.012553801	0.076175614	uncharacterized protein MGG_08493 [Pyricularia oryzae 70-15]
MGG_08494	606.7068981	2668.475421	4.398294184	2.136944103	1.69362E-07	7.50878E-06	cytochrome P450 52A13 [Pyricularia oryzae 70-15]
MGG_08498	35.24481659	135.4492416	3.843096793	1.942269313	0.000414565	0.005869618	uncharacterized protein MGG_08498 [Pyricularia oryzae 70-15]
MGG_08499	8.108310038	50.97444735	6.286691939	2.65230107	2.07846E-05	0.000500975	manganese lipoxygenase [Pyricularia oryzae 70-15]
MGG_08500	24.43430924	112.7444023	4.614184147	2.206075581	1.41185E-05	0.000362604	pisatin demethylase [Pyricularia oryzae 70-15]
MGG_08511	0	18.7226748	Inf	Inf	2.71001E-05	0.000630527	uncharacterized protein MGG_08511 [Pyricularia oryzae 70-15]
MGG_08516	1.014141685	112.2889632	110.7231512	6.790813098	3.29466E-19	1.42941E-16	uncharacterized protein MGG_08516 [Pyricularia oryzae 70-15]
MGG_08519	3.841136457	234.2566335	60.98628261	5.930412875	5.10341E-23	3.55048E-20	aflatoxin B1 aldehyde reductase member 3
MGG_08520	2.693530462	131.52651	48.83052627	5.609711422	6.61563E-18	2.29619E-15	oxidoreductase [Pyricularia oryzae 70-15]
MGG_08521	123.3364539	1350.441422	10.94924801	3.452759884	9.54198E-15	1.93193E-12	uncharacterized protein MGG_08521 [Pyricularia oryzae 70-15]
MGG_08523	0.339553209	110.0931551	324.2294648	8.340871393	1.04272E-20	5.75769E-18	laccase-1 [Pyricularia oryzae 70-15]
MGG_08525	37.34511354	328.0613677	8.784586163	3.134974323	1.41163E-05	0.000362604	uncharacterized protein MGG_08525 [Pyricularia oryzae 70-15]
MGG_08526	0.679106417	89.26379614	131.4430167	7.038293686	1.87984E-17	6.17197E-15	uncharacterized protein MGG_08526 [Pyricularia oryzae 70-15]
MGG_08527	139.9758786	883.3693036	6.310868073	2.657838465	2.04799E-06	7.07503E-05	uncharacterized protein MGG_08527 [Pyricularia oryzae 70-15]
MGG_08528	80.20070492	771.6151794	9.621052336	3.266194702	3.94288E-08	2.0823E-06	uncharacterized protein MGG_08528 [Pyricularia oryzae 70-15]
MGG_08529	4.11169854	92.82718104	22.57635868	4.496740909	5.19667E-12	6.18913E-10	uncharacterized protein MGG_08529 [Pyricularia oryzae 70-15]

MGG_08531	4.790804958	32.17863308	6.716748723	2.747763058	0.001742099	0.018165682	uncharacterized protein MGG_08531 [Pyricularia oryzae 70-15]
MGG_08532	0	10.94986729	Inf	Inf	0.003391812	0.029557912	uncharacterized protein MGG_08532 [Pyricularia oryzae 70-15]
MGG_08536	94.58527369	329.0869137	3.479261632	1.79878117	2.89934E-05	0.000668333	uncharacterized protein MGG_08536 [Pyricularia oryzae 70-15]
MGG_08539	1.390449427	17.66467272	12.70428998	3.667243842	0.000295554	0.004539057	uncharacterized protein MGG_08539 [Pyricularia oryzae 70-15]
MGG_08540	0.321175942	19.85081983	61.80668357	5.94969095	2.76458E-06	9.05233E-05	pyoverdine/dityrosine biosynthesis family protein
MGG_08541	62.47632749	570.0097095	9.123611012	3.189604938	2.02157E-12	2.62928E-10	FAD monooxygenase [Pyricularia oryzae 70-15]
MGG_08542	1011.587982	2254.507665	2.228681742	1.156190613	0.004657845	0.037848494	uncharacterized protein MGG_08542 [Pyricularia oryzae 70-15]
MGG_08543	562.3226395	2429.358709	4.320222125	2.111105491	4.26595E-06	0.000131497	uncharacterized protein MGG_08543 [Pyricularia oryzae 70-15]
MGG_08546	2.776380913	48.35023556	17.41484223	4.122245498	2.54948E-07	1.07166E-05	uncharacterized protein MGG_08546 [Pyricularia oryzae 70-15]
MGG_08551	22.52682083	47.7762716	2.120861703	1.084650549	0.048911295	0.190196677	uncharacterized protein MGG_08551 [Pyricularia oryzae 70-15]
MGG_08560	2369.137449	4869.917414	2.055565588	1.039535405	0.028081006	0.131709675	hypothetical protein, variant [Pyricularia oryzae 70-1]
MGG_08570	1049.904381	2226.880037	2.121031284	1.0847659	0.018012662	0.097079777	tubulin folding cofactor B [Pyricularia oryzae 70-15]
MGG_08572	10.51741909	1.577838244	0.150021429	-2.736759503	0.015418042	0.087006487	uncharacterized protein MGG_08572 [Pyricularia oryzae 70-15]
MGG_08577	965.889246	1954.136812	2.023147912	1.016601799	0.011090131	0.069913293	uncharacterized protein MGG_08577 [Pyricularia oryzae 70-15]
MGG_08580	4453.255818	1864.176346	0.418609759	-1.256322148	0.001575994	0.016808763	Na(+)/H(+) antiporter [Pyricularia oryzae 70-15]
MGG_08584	1557.620518	3447.261015	2.213158452	1.146106746	0.004473938	0.036697769	aconitate hydratase [Pyricularia oryzae 70-15]
MGG_08587	594.1514083	262.5683413	0.4419216	-1.178137645	0.003870568	0.032766313	uncharacterized protein MGG_08587 [Pyricularia oryzae 70-15]
MGG_08612	477.6115359	115.2039116	0.241208394	-2.051647981	1.62216E-06	5.73333E-05	uncharacterized protein MGG_08612 [Pyricularia oryzae 70-15]
MGG_08613	4301.028381	1123.637627	0.261248596	-1.93650481	3.06185E-06	9.75403E-05	polymerase 2 ADP-ribosyltransferase 2
MGG_08616	175.8520003	670.2964448	3.811707821	1.930437536	0.000513372	0.006929379	glycosyl hydrolase [Pyricularia oryzae 70-15]
MGG_08621	69.73952471	259.9262834	3.727101447	1.898054088	2.29313E-05	0.00054302	uncharacterized protein MGG_08621 [Pyricularia oryzae 70-15]
MGG_08622	18004.75866	39379.83145	2.187190186	1.129078675	0.005396589	0.042078154	nucleoside diphosphate kinase [Pyricularia oryzae 70-15]
MGG_08623	1319.379297	2666.414378	2.020961208	1.01504163	0.012234459	0.074622274	neutral alpha-glucosidase AB [Pyricularia oryzae 70-15]
MGG_08628	621.4256556	1364.018627	2.194982802	1.134209636	0.005821891	0.044733922	3'-5' exoribonuclease CSL4 [Pyricularia oryzae 70-15]
MGG_08642	448.0399706	1044.484375	2.331230344	1.221091561	0.002912283	0.026741055	uncharacterized protein MGG_08642 [Pyricularia oryzae 70-15]
MGG_08649	2048.703632	5682.185602	2.773551779	1.471734659	0.000284846	0.004419086	farnesyl pyrophosphate synthase [Pyricularia oryzae 70-15]
MGG_08657	2.097274496	92.53314375	44.1206642	5.463382604	2.76896E-15	6.22913E-13	uncharacterized protein MGG_08657 [Pyricularia oryzae 70-15]
MGG_08658	14.23079916	298.880614	21.00237736	4.392480737	3.21671E-10	2.79119E-08	uncharacterized protein MGG_08658 [Pyricularia oryzae 70-15]
MGG_08659	211.7935625	22160.18005	104.6310369	6.709167054	1.25467E-27	1.69353E-24	uncharacterized protein MGG_08659 [Pyricularia oryzae 70-15]
MGG_08660	110.4294294	249.020138	2.255016071	1.173137715	0.005382465	0.042052933	uncharacterized protein MGG_08660 [Pyricularia oryzae 70-15]
MGG_08675	87.11043873	994.766102	11.41959697	3.51343983	1.61764E-05	0.000406015	uncharacterized protein MGG_08675 [Pyricularia oryzae 70-15]
MGG_08677	29.85116327	67.12493386	2.248653872	1.169061609	0.022546307	0.11341306	uncharacterized protein MGG_08677 [Pyricularia oryzae 70-15]
MGG_08681	446.0074234	1180.357917	2.646498366	1.404084763	0.000736968	0.009220075	uncharacterized protein MGG_08681 [Pyricularia oryzae 70-15]
MGG_08682	62.64508288	131.4469164	2.098279871	1.069207119	0.022332445	0.112850601	uncharacterized protein MGG_08682 [Pyricularia oryzae 70-15]
MGG_08683	286.1188977	1195.344635	4.177789879	2.062739934	0.000302098	0.004610409	uncharacterized protein MGG_08683 [Pyricularia oryzae 70-15]
MGG_08684	0.642351884	27.23537142	42.39945751	5.405973901	2.0564E-07	8.89008E-06	uncharacterized protein MGG_08684 [Pyricularia oryzae 70-15]
MGG_08686	1026.426186	278.2192522	0.271056269	-1.883335722	0.009561161	0.06308753	uncharacterized protein MGG_08686 [Pyricularia oryzae 70-15]
MGG_08688	207.4961948	420.5372347	2.026722635	1.019148664	0.012868041	0.077533618	mitochondrial inner membrane protein [Pyricularia oryzae 70-15]
MGG_08694	465.7532251	1801.244845	3.867380295	1.951356637	0.001277759	0.014345857	uncharacterized protein MGG_08694 [Pyricularia oryzae 70-15]
MGG_08695	420.0049884	1139.94824	2.71413024	1.440489952	0.000520421	0.007008952	NAD-binding Rossmann fold oxidoreductase
MGG_08698	332.5962054	157.71333	0.474188603	-1.076467107	0.010351274	0.066780285	candidapepsin-8 [Pyricularia oryzae 70-15]
MGG_08708	5096.499979	2003.479369	0.393108874	-1.346999163	0.000718838	0.009049169	WD repeat-containing protein [Pyricularia oryzae 70-15]
MGG_08736	297.6343341	659.0211963	2.214197493	1.146783907	0.004201817	0.034818332	carboxypeptidase 2 [Pyricularia oryzae 70-15]
MGG_08741	2534.79338	1132.093519	0.446621617	-1.162875013	0.01349684	0.080019335	tRNA selenocysteine-associated protein 1
MGG_08747	0.339553209	26.17332369	77.0816562	6.268315665	7.92384E-08	3.81979E-06	uncharacterized protein MGG_08747 [Pyricularia oryzae 70-15]
MGG_08749	12.90274846	362.7775243	28.11629828	4.81333476	3.12134E-20	1.51672E-17	uncharacterized protein MGG_08749 [Pyricularia oryzae 70-15]
MGG_08752	352.6917092	87.42905171	0.247890862	-2.012223006	0.002214288	0.021780701	exopolygalacturonase [Pyricularia oryzae 70-15]
MGG_08765	3867.749836	1512.420895	0.391033795	-1.354634799	0.025725017	0.124306885	uncharacterized protein MGG_08765 [Pyricularia oryzae 70-15]

MGG_08772	291.045095	658.6966976	2.263211814	1.178371613	0.028231686	0.132314243	uncharacterized protein MGG_08772 [Pyricularia oryzae 70-15]
MGG_08785	291.1470928	1772.575113	6.088245966	2.606026645	1.07771E-09	8.13169E-08	X-Pro dipeptidyl-peptidase [Pyricularia oryzae 70-15]
MGG_08786	83.11321623	242.2123734	2.914246186	1.543122757	0.000586153	0.007648324	uncharacterized protein MGG_08786 [Pyricularia oryzae 70-15]
MGG_08801	845.9673701	1701.831131	2.011698313	1.008413966	0.010835383	0.068807232	exopolyphosphatase [Pyricularia oryzae 70-15]
MGG_08802	858.3802247	3272.391252	3.812286395	1.930656504	3.14734E-06	9.98273E-05	GMP synthase [Pyricularia oryzae 70-15]
MGG_08804	399.217416	1067.571614	2.674160923	1.419086285	0.000563581	0.00743364	Clp1 [Pyricularia oryzae 70-15]
MGG_08814	1272.601864	4382.277315	3.443557203	1.783899643	0.000998972	0.011747829	dihydroorotate dehydrogenase [Pyricularia oryzae 70-15]
MGG_08823	436.7831717	127.0767003	0.29093772	-1.78121774	3.6336E-05	0.000808443	uncharacterized protein MGG_08823 [Pyricularia oryzae 70-15]
MGG_08831	3462.173142	1586.418303	0.458214606	-1.125904648	0.004099771	0.034253107	uncharacterized protein MGG_08831 [Pyricularia oryzae 70-15]
MGG_08850	81.71180288	24.56818272	0.300668714	-1.733753337	0.000675648	0.008576565	cAMP-independent regulatory protein pac2 [
MGG_08860	2.758003647	13.79256845	5.000924661	2.322194871	0.015248095	0.086517449	exosome complex exonuclease RRP45
MGG_08875	897.2273688	180.4393957	0.20110777	-2.313959269	4.97216E-08	2.57029E-06	grisea protein [Pyricularia oryzae 70-15]
MGG_08881	66.86643402	20.00151162	0.299126339	-1.741173144	0.004934578	0.039411739	hypothetical protein, variant [Pyricularia oryzae 70-15]
MGG_08890	144.5707885	414.9546094	2.8702521	1.521177457	0.000363719	0.005317041	uncharacterized protein MGG_08890 [Pyricularia oryzae 70-15]
MGG_08899	494.0820331	1348.148918	2.728593286	1.448157368	0.00037625	0.005428372	uncharacterized protein MGG_08899 [Pyricularia oryzae 70-15]
MGG_08905	4648.218343	11142.4967	2.397154325	1.26132279	0.00353684	0.030385807	triosephosphate isomerase [Pyricularia oryzae 70-15]
MGG_08914	1201.315619	441.3412953	0.367381634	-1.444648589	0.000367926	0.00535919	uncharacterized protein MGG_08914 [Pyricularia oryzae 70-15]
MGG_08916	444.9965655	1721.631326	3.8688643	1.951910127	0.00173255	0.018112751	uncharacterized protein MGG_08916 [Pyricularia oryzae 70-15]
MGG_08938	127.5879992	284.2434321	2.227822632	1.155634377	0.007572925	0.053893317	uncharacterized protein MGG_08938 [Pyricularia oryzae 70-15]
MGG_08941	6.722378552	122.7440823	18.25902563	4.190537875	5.75072E-13	8.62466E-11	uncharacterized protein MGG_08941 [Pyricularia oryzae 70-15]
MGG_08944	1.064755544	57.72926997	54.21833237	5.760708835	2.03451E-12	2.62928E-10	uncharacterized protein MGG_08944 [Pyricularia oryzae 70-15]
MGG_08946	0.321175942	25.1533712	78.31648609	6.29124413	1.23747E-07	5.71588E-06	uncharacterized protein MGG_08946 [Pyricularia oryzae 70-15]
MGG_08947	1.730002636	39.39689231	22.77273543	4.509235691	2.38287E-08	1.33397E-06	cytochrome P450 [Pyricularia oryzae 70-15]
MGG_08950	28.74815771	183.7616379	6.39211875	2.67629421	3.53494E-08	1.89649E-06	high-affinity glucose transporter [Pyricularia oryzae 70-15]
MGG_08952	4.643481322	176.1112703	37.92655941	5.245136595	2.21835E-17	7.09171E-15	uncharacterized protein MGG_08952 [Pyricularia oryzae 70-15]
MGG_08957	0.321175942	34.50027545	107.4186168	6.747100239	9.64816E-10	7.41809E-08	uncharacterized protein MGG_08957 [Pyricularia oryzae 70-15]
MGG_08962	8.214055696	56.82709733	6.918275141	2.790412391	1.32114E-05	0.000344405	uncharacterized protein MGG_08962, partial
MGG_08973	783.3342691	2183.380034	2.78729033	1.478863284	0.000339664	0.005049232	hypothetical protein, variant [Pyricularia oryzae 70-15]
MGG_08976	668.2408997	2153.985925	3.223367391	1.688568632	0.00160348	0.017056981	nicotinamidase [Pyricularia oryzae 70-15]
MGG_08977	48.98214651	462.379494	9.439755646	3.238749515	3.38928E-07	1.41488E-05	ctr copper transporter [Pyricularia oryzae 70-15]
MGG_08979	4.707954507	19.04761962	4.045837655	2.016438431	0.014286914	0.083023391	uncharacterized protein MGG_08979 [Pyricularia oryzae 70-15]
MGG_08988	56.91935322	151.9335521	2.669277557	1.416449328	0.002121622	0.021098803	uncharacterized protein MGG_08988 [Pyricularia oryzae 70-15]
MGG_08994	235.6911065	1036.044935	4.395774412	2.13611735	0.004231729	0.035018418	uncharacterized protein MGG_08994 [Pyricularia oryzae 70-15]
MGG_09000	45.15331037	458.4361294	10.15287973	3.343817081	2.6911E-13	4.37836E-11	CMGC/CDK protein kinase [Pyricularia oryzae 70-15]
MGG_09001	48.34431256	480.98153	9.949082002	3.314563415	5.5987E-13	8.50163E-11	uncharacterized protein MGG_09001 [Pyricularia oryzae 70-15]
MGG_09015	42.73886242	141.7065299	3.315636445	1.729285826	0.000314274	0.004760355	uncharacterized protein MGG_09015 [Pyricularia oryzae 70-15]
MGG_09016	218.8377595	97.71373294	0.446512216	-1.163228449	0.007845556	0.054947639	uncharacterized protein MGG_09016 [Pyricularia oryzae 70-15]
MGG_09021	41.56048329	13.14097853	0.316189262	-1.661139722	0.02796314	0.131529491	uncharacterized protein MGG_09021 [Pyricularia oryzae 70-15]
MGG_09022	282.3867205	13.59767711	0.048152679	-4.376240124	1.7775E-17	5.99809E-15	uncharacterized protein MGG_09022 [Pyricularia oryzae 70-15]
MGG_09027	1974.380461	832.0597195	0.421428259	-1.246641039	0.002347599	0.022725488	fungal specific transcription factor domain-containing protein
MGG_09029	764.0968592	2148.89989	2.812339645	1.491770838	0.001382359	0.015169734	uncharacterized protein MGG_09029 [Pyricularia oryzae 70-15]
MGG_09030	546.4571985	237.0982225	0.433882513	-1.204623653	0.003404785	0.029620812	uncharacterized protein MGG_09030 [Pyricularia oryzae 70-15]
MGG_09032	497.1666598	3930.799454	7.906401962	2.983021304	8.58629E-07	3.29042E-05	aspergillopepsin-2 [Pyricularia oryzae 70-15]
MGG_09036	118.9857685	5.402479113	0.045404414	-4.461023628	0.00057192	0.007494427	alpha-L-arabinofuranosidase [Pyricularia oryzae 70-15]
MGG_09048	262.7631525	642.9824169	2.44700374	1.291016307	0.002029525	0.020460307	NACHT and Ankyrin domain-containing prote
MGG_09054	63.50246399	184.6746205	2.908148895	1.540101136	0.000641491	0.00824638	uncharacterized protein MGG_09054 [Pyricularia oryzae 70-15]
MGG_09063	9.770511528	155.0466557	15.86883709	3.988124502	1.86075E-13	3.22919E-11	urea active transporter [Pyricularia oryzae 70-15]
MGG_09070	44.78848199	169.9454714	3.794401236	1.923872243	3.35348E-05	0.000756286	uncharacterized protein MGG_09070 [Pyricularia oryzae 70-15]

MGG_09072	117856.0675	57484.45791	0.487751366	-1.035782182	0.035423462	0.154041331	alcohol oxidase [Pyricularia oryzae 70-15]
MGG_09073	69.43043913	170.3525655	2.453571771	1.294883473	0.00774176	0.054534963	minor extracellular protease vpr [Pyricularia oryzae 70-15]
MGG_09075	299.5313994	735.5987238	2.45583176	1.296211731	0.001951833	0.019908369	uncharacterized protein MGG_09075 [Pyricularia oryzae 70-15]
MGG_09076	953.8596025	380.9232754	0.399349416	-1.32427649	0.001187392	0.013506024	uncharacterized protein MGG_09076 [Pyricularia oryzae 70-15]
MGG_09079	868.8972438	239.896483	0.276093042	-1.856773566	8.4327E-06	0.000239347	uncharacterized protein MGG_09079 [Pyricularia oryzae 70-15]
MGG_09080	3185.744891	6959.059441	2.184437134	1.127261587	0.005254068	0.041338351	arginyl-tRNA synthetase [Pyricularia oryzae 70-15]
MGG_09087	29.8665816	131.4989767	4.402880065	2.138447546	1.47305E-05	0.000375637	3-oxoacyl-[acyl-carrier-protein] reductase
MGG_09091	119.1983222	550.8132447	4.620981525	2.208199322	0.00011655	0.002138752	adiponectin receptor protein 1 [Pyricularia oryzae 70-15]
MGG_09100	222.9660984	1857.61632	8.331384606	3.058556279	3.33805E-06	0.000105053	cutinase [Pyricularia oryzae 70-15]
MGG_09119	8601.449989	2740.036684	0.318555207	-1.650384674	0.011909433	0.073476784	potassium transporter [Pyricularia oryzae 70-15]
MGG_09131	91.79628695	878.4727664	9.569807185	3.258489857	7.5907E-05	0.001480126	alpha/beta hydrolase [Pyricularia oryzae 70-15]
MGG_09134	7166.550723	21330.46073	2.97639151	1.573564309	0.000103224	0.001932146	uncharacterized protein MGG_09134 [Pyricularia oryzae 70-15]
MGG_09137	40.01221089	483.1574912	12.07525104	3.593981277	2.17885E-13	3.6762E-11	uncharacterized protein MGG_09137 [Pyricularia oryzae 70-15]
MGG_09138	596.8594091	2366.441152	3.964821725	1.987255998	0.00066112	0.008445101	glutathione S-transferase II [Pyricularia oryzae 70-15]
MGG_09139	39.75550813	88.01059858	2.213796345	1.146522509	0.038595527	0.162651436	laccase-1 [Pyricularia oryzae 70-15]
MGG_09146	49.8616339	292.6152284	5.868544721	2.553002789	2.85597E-08	1.54886E-06	RTA1 domain-containing protein [Pyricularia oryzae 70-15]
MGG_09147	41.19440141	115.9513205	2.814734929	1.492999066	0.001609337	0.017096355	uncharacterized protein MGG_09147 [Pyricularia oryzae 70-15]
MGG_09148	9.577091975	28.14424528	2.938704709	1.555180399	0.020109991	0.104668456	uncharacterized protein MGG_09148 [Pyricularia oryzae 70-15]
MGG_09149	0.679106417	54.47818904	80.22040087	6.32589727	1.21033E-12	1.70967E-10	uncharacterized protein MGG_09149 [Pyricularia oryzae 70-15]
MGG_09150	3111.579611	19840.26856	6.376268982	2.672712491	2.3584E-10	2.17044E-08	hypothetical protein, variant [Pyricularia oryzae 70-15]
MGG_09154	279.3803119	2540.114117	9.091958198	3.184591051	5.06108E-12	6.08733E-10	uncharacterized protein MGG_09154 [Pyricularia oryzae 70-15]
MGG_09158	0.339553209	10.98543482	32.35261672	5.015810499	0.000728969	0.009148263	uncharacterized protein MGG_09158 [Pyricularia oryzae 70-15]
MGG_09167	19630.87324	3996.16456	0.203565298	-2.296436451	2.07714E-06	7.1482E-05	uncharacterized protein MGG_09167 [Pyricularia oryzae 70-15]
MGG_09177	54.58307871	21.96712813	0.40245308	-1.313107499	0.017305287	0.094458269	uncharacterized protein MGG_09177 [Pyricularia oryzae 70-15]
MGG_09181	2344.89913	7233.24987	3.08467421	1.625118126	0.000605447	0.007891604	hydroxymethylglutaryl-CoA lyase [Pyricularia oryzae 70-15]
MGG_09188	1020.864993	2189.408057	2.144659748	1.100748781	0.007034352	0.051139023	malate dehydrogenase [Pyricularia oryzae 70-15]
MGG_09197	121.2269111	355.1809111	2.929885023	1.55084405	0.00032386	0.004851109	uncharacterized protein MGG_09197 [Pyricularia oryzae 70-15]
MGG_09198	314.2261161	1352.551073	4.30438784	2.105808075	5.36973E-07	2.14577E-05	ent-kaurene oxidase [Pyricularia oryzae 70-15]
MGG_09200	22234.91462	2843.457373	0.127882541	-2.967108783	0.049734628	0.19275316	zinc finger protein [Pyricularia oryzae 70-15]
MGG_09225	41.72560521	162.8098654	3.901917409	1.964183241	3.66685E-05	0.000814348	uncharacterized protein MGG_09225 [Pyricularia oryzae 70-15]
MGG_09226	268.0901438	1099.676099	4.101889324	2.036288566	1.82318E-06	6.4197E-05	fumarate reductase [Pyricularia oryzae 70-15]
MGG_09227	990.2881565	2336.414614	2.359328038	1.238376023	0.002109766	0.021042227	uncharacterized protein MGG_09227 [Pyricularia oryzae 70-15]
MGG_09233	31.72760506	3.060712883	0.09646845	-3.373799003	1.31232E-05	0.000342841	uncharacterized protein MGG_09233 [Pyricularia oryzae 70-15]
MGG_09237	596.860262	1199.354238	2.009438915	1.006792722	0.014522089	0.084046849	uncharacterized protein MGG_09237 [Pyricularia oryzae 70-15]
MGG_09248	544.5641352	120.2875695	0.220887792	-2.178614407	4.68471E-07	1.87821E-05	uncharacterized protein MGG_09248 [Pyricularia oryzae 70-15]
MGG_09249	825.0687003	311.5188943	0.377567219	-1.405194584	0.000534478	0.007142835	peptidase S9 prolyl oligopeptidase active site-containing protein
MGG_09266	358.0308926	105.9383012	0.295891509	-1.756859797	3.92675E-05	0.000857953	uncharacterized protein MGG_09266 [Pyricularia oryzae 70-15]
MGG_09271	75.13253553	158.5233313	2.109915899	1.077185495	0.033090207	0.147994011	uncharacterized protein MGG_09271 [Pyricularia oryzae 70-15]
MGG_09273	92.43717538	243.4616358	2.633806526	1.397149372	0.001410285	0.015434364	uncharacterized protein MGG_09273 [Pyricularia oryzae 70-15]
MGG_09300	917.7769914	1938.426156	2.11208842	1.078670233	0.007324233	0.052554512	DNA replication licensing factor mcm7 [Pyricularia oryzae 70-15]
MGG_09312	394.4509062	145.4779352	0.368811259	-1.439045398	0.000773854	0.009574325	uncharacterized protein MGG_09312 [Pyricularia oryzae 70-15]
MGG_09314	529.7153183	56.2832591	0.1062519	-3.234439453	0.000470251	0.006491606	lipolytic enzyme [Pyricularia oryzae 70-15]
MGG_09323	115.1224302	298.3994463	2.592018304	1.374075906	0.001335719	0.014764612	phenylacetone monooxygenase [Pyricularia oryzae 70-15]
MGG_09326	100.9085128	31.23103827	0.309498549	-1.691995451	0.000619377	0.008030083	uncharacterized protein MGG_09326 [Pyricularia oryzae 70-15]
MGG_09332	178.9586251	480.8699843	2.68704559	1.426020799	0.000792974	0.009769822	uncharacterized protein MGG_09332 [Pyricularia oryzae 70-15]
MGG_09333	57.33299447	122.4951998	2.136556811	1.095287679	0.030160681	0.13873228	nucleoside-diphosphate-sugar epimerase
MGG_09341	51.11923003	255.3028289	4.994262019	2.320271513	4.12962E-07	1.6867E-05	LOW QUALITY PROTEIN: uncharacterized protein MGG_09341
MGG_09348	691.9298609	1472.691594	2.12838277	1.08975763	0.0064701	0.048338733	mitochondrial intermembrane space import and assembly protein 40 [P

MGG_09349	3988.291303	10185.20177	2.553775789	1.352631868	0.000555161	0.007354525	uncharacterized protein MGG_09349 [Pyricularia oryzae 70-15]
MGG_09354	17.92912992	41.47644727	2.313355275	1.209986845	0.035103457	0.15339453	uncharacterized protein MGG_09354 [Pyricularia oryzae 70-15]
MGG_09361	4166.622432	1979.146579	0.475000222	-1.073999908	0.019069821	0.101029302	uncharacterized protein MGG_09361 [Pyricularia oryzae 70-15]
MGG_09376	30.83187838	760.1994206	24.65627981	4.623883234	2.23673E-13	3.72216E-11	FAD binding domain-containing protein [Pyricularia oryzae 70-15]
MGG_09378	0.371789801	8.175782309	21.9903351	4.458797684	0.014656732	0.084463938	uncharacterized protein MGG_09378 [Pyricularia oryzae 70-15]
MGG_09380	17.58386879	135.6908575	7.716780593	2.947999087	0.005960179	0.045441286	uncharacterized protein MGG_09380 [Pyricularia oryzae 70-15]
MGG_09381	152.3311205	682.3056735	4.479095745	2.163207505	0.001348817	0.014895848	uncharacterized protein MGG_09381 [Pyricularia oryzae 70-15]
MGG_09384	17.7013358	56.37814909	3.184965798	1.67127788	0.002081228	0.020843159	uncharacterized protein MGG_09384 [Pyricularia oryzae 70-15]
MGG_09396	5682.828142	14863.74527	2.615554245	1.387116691	0.000570812	0.007494427	HET-C2 protein [Pyricularia oryzae 70-15]
MGG_09397	3.964374905	20.86336293	5.262711885	2.395806414	0.003527311	0.030346867	uncharacterized protein MGG_09397 [Pyricularia oryzae 70-15]
MGG_09402	1215.592337	334.459792	0.275141412	-1.861754796	1.06898E-05	0.000290514	uncharacterized protein MGG_09402 [Pyricularia oryzae 70-15]
MGG_09417	1.707107428	12.27089924	7.188123629	2.845615222	0.008586105	0.058461047	uncharacterized protein MGG_09417 [Pyricularia oryzae 70-15]
MGG_09426	27.16184524	118.5467291	4.364457864	2.125802459	0.010027351	0.065560957	uncharacterized protein MGG_09426 [Pyricularia oryzae 70-15]
MGG_09441	34.064974	105.2111265	3.088542692	1.626926273	0.001286537	0.014404473	tricarboxylate transporter [Pyricularia oryzae 70-15]
MGG_09457	637.9145112	1924.547267	3.016936021	1.593084102	0.003519668	0.030338106	thymidylate kinase [Pyricularia oryzae 70-15]
MGG_09461	26.7839785	74.91293791	2.796930931	1.483844628	0.002726843	0.025540235	UV-damage endonuclease [Pyricularia oryzae 70-15]
MGG_09466	2129.798448	5544.917658	2.603494084	1.380449128	0.008696655	0.059020651	uncharacterized protein MGG_09466 [Pyricularia oryzae 70-15]
MGG_09469	416.9442496	956.1706187	2.293281703	1.197413584	0.003770045	0.032161869	uncharacterized protein MGG_09469 [Pyricularia oryzae 70-15]
MGG_09484	2665.241257	1324.416906	0.496921959	-1.008908799	0.030921228	0.140891933	uncharacterized protein MGG_09484 [Pyricularia oryzae 70-15]
MGG_09521	366.0880921	147.5533709	0.403054276	-1.310953968	0.002101867	0.02098067	uncharacterized protein MGG_09521 [Pyricularia oryzae 70-15]
MGG_09535	2515.154742	5781.406358	2.298628495	1.200773316	0.002851744	0.02636262	ATPase GET3 [Pyricularia oryzae 70-15]
MGG_09543	184.583706	45.84866787	0.248389573	-2.009323483	0.000574898	0.007512752	uncharacterized protein MGG_09543 [Pyricularia oryzae 70-15]
MGG_09545	564.8732688	209.7037946	0.371240429	-1.429574263	0.000549215	0.007283696	uncharacterized protein MGG_09545 [Pyricularia oryzae 70-15]
MGG_09546	43680.86202	13760.59559	0.315025733	-1.666458413	0.002340748	0.022711415	uncharacterized protein MGG_09546 [Pyricularia oryzae 70-15]
MGG_09554	350.9351967	1055.685298	3.008205811	1.588903275	0.000126867	0.002273136	anaphase-promoting complex subunit 1
MGG_09557	2690.291989	1327.495551	0.493439209	-1.019055737	0.012849117	0.077503014	vacuolar protein sorting-associated protein 4
MGG_09570	262.4264118	128.2783888	0.488816609	-1.032634788	0.027239465	0.129310286	uncharacterized protein MGG_09570 [Pyricularia oryzae 70-15]
MGG_09575	3719.293263	1477.746471	0.397319159	-1.331629732	0.005962579	0.045441286	uncharacterized protein MGG_09575 [Pyricularia oryzae 70-15]
MGG_09598	0	9.692487386	Inf	Inf	0.000449637	0.006249641	uncharacterized protein MGG_09598 [Pyricularia oryzae 70-15]
MGG_09602	1456.500782	554.6263194	0.380793698	-1.392918491	0.000762633	0.009463194	uncharacterized protein MGG_09602 [Pyricularia oryzae 70-15]
MGG_09605	12.06453354	44.67424527	3.70294012	1.88867122	0.009534308	0.063015654	uncharacterized protein MGG_09605 [Pyricularia oryzae 70-15]
MGG_09607	750.4337315	1934.473493	2.577807223	1.366144378	0.000942186	0.011232257	maltose permease MAL31 [Pyricularia oryzae 70-15]
MGG_09610	7.769641306	0.592157029	0.076214204	-3.713796288	0.016015994	0.089536193	uncharacterized protein MGG_09610 [Pyricularia oryzae 70-15]
MGG_09636	0	6.915920336	Inf	Inf	0.003594304	0.03081412	uncharacterized protein MGG_09636 [Pyricularia oryzae 70-15]
MGG_09639	806.7265264	1998.397769	2.477168784	1.308692171	0.010818642	0.068736853	alpha-1,3-glucan synthase Ags2 [Pyricularia oryzae 70-15]
MGG_09645	640.0319133	51.1256536	0.079879851	-3.646024556	0.003863623	0.032740386	polyketide synthase [Pyricularia oryzae 70-15]
MGG_09647	413.0843609	34.62410536	0.083818485	-3.576587742	0.013102792	0.078596933	acyl-CoA synthetase [Pyricularia oryzae 70-15]
MGG_09648	15.34980203	73.66789472	4.79927328	2.262815965	0.000118545	0.002159054	uncharacterized protein MGG_09648 [Pyricularia oryzae 70-15]
MGG_09649	597.3112688	1643.681423	2.751800458	1.46037586	0.000403905	0.00574455	uncharacterized protein MGG_09649 [Pyricularia oryzae 70-15]
MGG_09659	8.043836853	0	0	-Inf	0.001143002	0.013100398	S-(hydroxymethyl)glutathione dehydrogenase
MGG_09664	123.3404884	262.4022487	2.127462378	1.089133619	0.012710401	0.076857121	beta-mannosidase [Pyricularia oryzae 70-15]
MGG_09676	12.74727342	39.74043422	3.117563491	1.640418942	0.007834372	0.054917459	uncharacterized protein MGG_09676 [Pyricularia oryzae 70-15]
MGG_09700	796.2423433	365.647281	0.459216072	-1.12275496	0.005655976	0.043735708	tyrosine-protein phosphatase YVH1 [Pyricularia oryzae 70-15]
MGG_09705	253.3835654	782.4713223	3.088090267	1.626714924	0.002308178	0.022503809	nucleoside-diphosphate-sugar epimerase
MGG_09720	125.6308134	338.6666116	2.695728878	1.430675405	0.000850085	0.010368301	uncharacterized protein MGG_09720 [Pyricularia oryzae 70-15]
MGG_09727	1489.969263	405.9504232	0.272455569	-1.875907115	0.021758705	0.110688753	FAD binding domain-containing protein [Pyricularia oryzae 70-15]
MGG_09728	30546.78183	6990.978436	0.228861373	-2.127454107	0.023710323	0.117086588	lactose permease [Pyricularia oryzae 70-15]
MGG_09729	45.87888173	1.480392573	0.032267408	-4.953778519	8.24506E-10	6.50396E-08	uncharacterized protein MGG_09729 [Pyricularia oryzae 70-15]

MGG_09732	1319.92956	383.6299774	0.290644281	-1.782673576	0.0069607	0.050804353	feruloyl esterase [Pyricularia oryzae 70-15]
MGG_09733	64.99475214	26.86870912	0.413398132	-1.274396225	0.016243475	0.090379689	secreted glucosidase [Pyricularia oryzae 70-15]
MGG_09750	1386.408524	4163.651118	3.003192094	1.586496757	9.97389E-05	0.001881409	diphosphomevalonate decarboxylase [Pyricularia oryzae 70-15]
MGG_09753	68.87331766	21.02194177	0.305226211	-1.712049239	0.001119343	0.01292564	uncharacterized protein MGG_09753 [Pyricularia oryzae 70-15]
MGG_09758	2345.424041	884.8531898	0.377267895	-1.406338763	0.000452515	0.006275286	allantoate permease [Pyricularia oryzae 70-15]
MGG_09761	4174.807213	8777.218398	2.102424843	1.072054228	0.007462304	0.05332475	uncharacterized protein MGG_09761 [Pyricularia oryzae 70-15]
MGG_09766	1381.645235	2819.612458	2.040764435	1.029109662	0.010403636	0.06701133	3-deoxy-7-phosphoheptulonate synthase
MGG_09767	10935.70303	4613.833989	0.421905567	-1.245007971	0.00256234	0.024204747	uncharacterized protein MGG_09767 [Pyricularia oryzae 70-15]
MGG_09773	89.76290666	221.337041	2.465796276	1.302053609	0.003172563	0.028296838	uncharacterized protein MGG_09773 [Pyricularia oryzae 70-15]
MGG_09781	390.3385143	194.1517149	0.49739318	-1.007541369	0.013956799	0.081670132	uncharacterized protein MGG_09781 [Pyricularia oryzae 70-15]
MGG_09785	17.14998577	185.9119764	10.84035748	3.438340427	0.000143146	0.002498474	uncharacterized protein MGG_09785 [Pyricularia oryzae 70-15]
MGG_09795	2.097274496	14.648233	6.984413831	2.804139042	0.008529307	0.058275601	uncharacterized protein MGG_09795 [Pyricularia oryzae 70-15]
MGG_09796	6.282482103	41.61683305	6.624266073	2.727760622	3.21912E-05	0.000733695	uncharacterized protein MGG_09796 [Pyricularia oryzae 70-15]
MGG_09798	3959.254898	1649.051473	0.416505508	-1.263592521	0.001600624	0.017041526	uncharacterized protein MGG_09798 [Pyricularia oryzae 70-15]
MGG_09805	40.25801326	446.1818386	11.08305658	3.470283909	1.13567E-13	1.99944E-11	alpha-galactosidase [Pyricularia oryzae 70-15]
MGG_09806	9837.029377	21630.49038	2.198884394	1.136771757	0.004957963	0.039520536	uncharacterized protein MGG_09806 [Pyricularia oryzae 70-15]
MGG_09814	2.808617505	19.20249376	6.836991412	2.773361613	0.001400328	0.015339216	3-isopropylmalate dehydratase large subunit 2
MGG_09816	312.991454	82.28844292	0.262909552	-1.927361537	0.033933635	0.149954819	uncharacterized protein MGG_09816 [Pyricularia oryzae 70-15]
MGG_09819	0.71134301	45.19911918	63.54054031	5.989605453	4.08502E-11	4.27801E-09	uncharacterized protein MGG_09819 [Pyricularia oryzae 70-15]
MGG_09820	86.35891767	181.4943337	2.101628165	1.07150744	0.015428019	0.087006487	uncharacterized protein MGG_09820 [Pyricularia oryzae 70-15]
MGG_09827	305.9412877	686.2796197	2.243174254	1.165541696	0.012247529	0.074622274	sugar transporter [Pyricularia oryzae 70-15]
MGG_09830	9228.589948	2635.126292	0.285539428	-1.808238126	9.82267E-06	0.000271813	AAA family ATPase [Pyricularia oryzae 70-15]
MGG_09834	1009.574347	4292.522111	4.251813772	2.088078409	5.65396E-07	2.24458E-05	catalase-peroxidase 2 [Pyricularia oryzae 70-15]
MGG_09838	3862.235709	1561.789016	0.40437434	-1.306236642	0.007569571	0.053893317	sulfate transporter 4.1 [Pyricularia oryzae 70-15]
MGG_09839	111.4073447	257.5264376	2.311575043	1.208876199	0.006431876	0.048201373	uncharacterized protein MGG_09839 [Pyricularia oryzae 70-15]
MGG_09840	463.7823191	1027.694398	2.215898181	1.147891592	0.005530695	0.042982981	acetylxylan esterase 2 [Pyricularia oryzae 70-15]
MGG_09841	63.19368392	527.6863296	8.350301752	3.061828333	1.74583E-07	7.71213E-06	uncharacterized protein MGG_09841 [Pyricularia oryzae 70-15]
MGG_09842	278.2452797	2319.200199	8.335092698	3.059198244	2.00874E-08	1.16757E-06	uncharacterized protein MGG_09842 [Pyricularia oryzae 70-15]
MGG_09845	32.88669042	162.5806089	4.943659786	2.305579463	0.012561029	0.076181417	uncharacterized protein MGG_09845 [Pyricularia oryzae 70-15]
MGG_09851	17.2418721	140.3662926	8.141012284	3.025208196	2.74886E-09	1.86554E-07	uncharacterized protein MGG_09851 [Pyricularia oryzae 70-15]
MGG_09852	53.99637407	342.7966142	6.34851173	2.666418423	1.88671E-09	1.33254E-07	sugar transporter STL1 [Pyricularia oryzae 70-15]
MGG_09856	120.3122281	248.9939475	2.069564759	1.049327393	0.017815138	0.096400133	dihydroxyacetone synthase [Pyricularia oryzae 70-15]
MGG_09870	34.94840037	75.47296478	2.1595542	1.110733525	0.028352982	0.132678067	uncharacterized protein MGG_09870 [Pyricularia oryzae 70-15]
MGG_09887	1987.792523	4355.522037	2.191135135	1.131678463	0.01826089	0.097982901	NEDD8 [Pyricularia oryzae 70-15]
MGG_09920	2761.582607	1274.601135	0.46154735	-1.115449433	0.006614124	0.048992917	cytochrome P450 52A5 [Pyricularia oryzae 70-15]
MGG_09928	5233.885326	2359.648926	0.450840777	-1.149310087	0.008481644	0.058047896	uncharacterized protein MGG_09928 [Pyricularia oryzae 70-15]
MGG_09931	8251.696383	821.3049846	0.099531653	-3.32870078	0.000130859	0.002327877	lipid A export ATP-binding/permease msbA
MGG_09941	30013.2954	2934.494189	0.097773142	-3.354417976	1.64274E-10	1.52336E-08	nod factor export ATP-binding protein I [Pyricularia oryzae 70-15]
MGG_09954	1815.808004	692.470306	0.381356567	-1.390787553	0.000740845	0.009249522	RSP [Pyricularia oryzae 70-15]
MGG_09986	1461.665082	6333.371232	4.332983877	2.115360868	0.000413777	0.005865301	uncharacterized protein MGG_09986 [Pyricularia oryzae 70-15]
MGG_09998	1178.76663	328.9288861	0.279044959	-1.84143051	9.25322E-06	0.000259603	uncharacterized protein MGG_09998 [Pyricularia oryzae 70-15]
MGG_10004	2577.349992	13620.86623	5.284833752	2.401858089	9.38024E-09	5.83374E-07	uncharacterized protein MGG_10004 [Pyricularia oryzae 70-15]
MGG_10008	15.16209039	49.76126698	3.28195293	1.714554548	0.00273783	0.025583964	uncharacterized protein MGG_10008 [Pyricularia oryzae 70-15]
MGG_10010	42.36157465	122.4646701	2.890937627	1.531537482	0.001057788	0.012308432	uncharacterized protein MGG_10010 [Pyricularia oryzae 70-15]
MGG_10011	40.73222076	128.6255355	3.157832623	1.658934705	0.000417873	0.005895846	polyketide synthase [Pyricularia oryzae 70-15]
MGG_10019	73.36217639	331.2916841	4.515837731	2.174993646	9.23197E-07	3.51567E-05	uncharacterized protein MGG_10019 [Pyricularia oryzae 70-15]
MGG_10027	8.301729591	39.7131683	4.783722219	2.258133618	0.011329963	0.071033049	calcium-transporting ATPase 1 [Pyricularia oryzae 70-15]
MGG_10031	2.335599987	33.1792435	14.20587587	3.82841588	2.05006E-06	7.07503E-05	uncharacterized protein MGG_10031 [Pyricularia oryzae 70-15]

MGG_10035	631.3491346	1502.897296	2.380453561	1.251236485	0.019138637	0.101261393	uncharacterized protein MGG_10035 [Pyricularia oryzae 70-15]
MGG_10038	126.136131	62.14491886	0.492681346	-1.021273245	0.027133484	0.128958356	beta-glucosidase C [Pyricularia oryzae 70-15]
MGG_10042	0	9.959699596	Inf	Inf	0.010186349	0.066137767	uncharacterized protein MGG_10042 [Pyricularia oryzae 70-15]
MGG_10046	318.8280195	892.8251709	2.800334714	1.485599278	0.000354905	0.005206987	pantothenate transporter liz [Pyricularia oryzae 70-15]
MGG_10047	21.00260159	141.2075055	6.723334009	2.749176823	5.44387E-08	2.77866E-06	uncharacterized protein MGG_10047 [Pyricularia oryzae 70-15]
MGG_10048	484.5738821	4333.625156	8.94316701	3.160785818	5.12715E-14	9.58224E-12	uncharacterized protein MGG_10048 [Pyricularia oryzae 70-15]
MGG_10051	310.6113297	776.9789929	2.501450909	1.322765139	0.00131635	0.014657215	uncharacterized protein MGG_10051 [Pyricularia oryzae 70-15]
MGG_10052	1514.013189	3878.666352	2.561844493	1.357182905	0.000751749	0.009337677	hypoxanthine guanine phosphoribosyltransferase
MGG_10060	44.58810102	15.62024444	0.35032316	-1.513241727	0.01008474	0.065794533	ABC-type Fe3+ transport system [Pyricularia oryzae 70-15]
MGG_10061	1704.208634	5750.072557	3.374042615	1.754478195	8.0762E-06	0.000230304	catalase-1 [Pyricularia oryzae 70-15]
MGG_10062	20.30749787	102.6252685	5.053565395	2.337301598	4.82899E-06	0.00014711	uncharacterized protein MGG_10062 [Pyricularia oryzae 70-15]
MGG_10065	3.601926489	17.35169739	4.817338012	2.268236155	0.010522908	0.06763613	uncharacterized protein MGG_10065 [Pyricularia oryzae 70-15]
MGG_10066	51.53314475	12.12224867	0.235232077	-2.087843291	0.002868396	0.02643158	uncharacterized protein MGG_10066 [Pyricularia oryzae 70-15]
MGG_10067	8.392731447	115.3943164	13.74931596	3.78128794	1.86802E-05	0.000460906	uncharacterized protein MGG_10067 [Pyricularia oryzae 70-15]
MGG_10070	1.353694894	12.36338078	9.133063023	3.191098789	0.004808422	0.038697345	averantin oxidoreductase [Pyricularia oryzae 70-15]
MGG_10083	14.47275811	34.17363035	2.361238271	1.239543631	0.046350632	0.18388879	endoglucanase 3 [Pyricularia oryzae 70-15]
MGG_10102	4.896550616	743.1887401	151.7780165	7.245819036	8.21353E-36	1.99556E-32	uncharacterized protein MGG_10102 [Pyricularia oryzae 70-15]
MGG_10105	289.3607746	96100.32105	332.1124682	8.375528076	7.40494E-54	8.99552E-50	uncharacterized protein MGG_10105 [Pyricularia oryzae 70-15]
MGG_10106	658.0346479	4356.242072	6.620080092	2.726848671	5.57769E-11	5.69393E-09	uncharacterized protein MGG_10106 [Pyricularia oryzae 70-15]
MGG_10111	1969.348309	23823.89977	12.09735203	3.596619388	2.12679E-12	2.71961E-10	glucose and ribitol dehydrogenase [Pyricularia oryzae 70-15]
MGG_10112	918.6480593	1924.024331	2.09440853	1.066542878	0.009184862	0.061441465	uncharacterized protein MGG_10112 [Pyricularia oryzae 70-15]
MGG_10131	11292.18038	4918.360054	0.435554507	-1.199074822	0.003152986	0.028216058	MFS transporter [Pyricularia oryzae 70-15]
MGG_10140	1365.259898	2773.998537	2.031846494	1.022791411	0.010144804	0.066009145	uncharacterized protein MGG_10140 [Pyricularia oryzae 70-15]
MGG_10157	634.7100569	264.1175498	0.416123152	-1.264917535	0.002447755	0.023483637	uncharacterized protein MGG_10157 [Pyricularia oryzae 70-15]
MGG_10158	9.747310818	42.54756786	4.365057055	2.126000511	0.000575145	0.007512752	QDE-2-interacting protein [Pyricularia oryzae 70-15]
MGG_10170	258.3341534	534.4704699	2.06891138	1.04887185	0.01156708	0.071897572	uncharacterized protein MGG_10170 [Pyricularia oryzae 70-15]
MGG_10200	559.9829415	255.4691318	0.456208775	-1.132233898	0.0062501	0.047097035	OPT family small oligopeptide transporter
MGG_10207	10.41255791	138.57527	13.30847533	3.734273394	5.50701E-12	6.49507E-10	uncharacterized protein MGG_10207 [Pyricularia oryzae 70-15]
MGG_10213	207.648533	86.7849448	0.417941526	-1.258626984	0.004188002	0.034818332	uncharacterized protein MGG_10213 [Pyricularia oryzae 70-15]
MGG_10214	139.7441456	48.49773713	0.347046647	-1.526798505	0.00117946	0.013440979	uncharacterized protein MGG_10214 [Pyricularia oryzae 70-15]
MGG_10216	178.0148744	65.04997581	0.365418766	-1.452377368	0.001378998	0.015160247	uncharacterized protein MGG_10216 [Pyricularia oryzae 70-15]
MGG_10221	27.87349376	70.20458479	2.518686226	1.332671404	0.009141609	0.061287119	uncharacterized protein MGG_10221 [Pyricularia oryzae 70-15]
MGG_10233	484.3941305	2021.549356	4.173356424	2.061208139	0.000114103	0.002109784	uncharacterized protein MGG_10233 [Pyricularia oryzae 70-15]
MGG_10235	1171.338862	297.2659832	0.253783079	-1.978332213	0.007402428	0.053027239	uncharacterized protein MGG_10235 [Pyricularia oryzae 70-15]
MGG_10239	1.404308753	23.33932817	16.61979827	4.054830965	0.000547343	0.007270124	uncharacterized protein MGG_10239 [Pyricularia oryzae 70-15]
MGG_10244	141.9042072	42.60421454	0.300232216	-1.735849305	0.036389633	0.156481863	uncharacterized protein MGG_10244 [Pyricularia oryzae 70-15]
MGG_10245	46.28617256	12.70617771	0.274513467	-1.865051168	0.007748699	0.054534963	uncharacterized protein MGG_10245 [Pyricularia oryzae 70-15]
MGG_10246	160.1135267	347.6307648	2.171151757	1.11846057	0.009991485	0.065488968	uncharacterized protein MGG_10246 [Pyricularia oryzae 70-15]
MGG_10247	6.05713146	19.10753016	3.154551009	1.657434679	0.034673963	0.151955016	uncharacterized protein MGG_10247 [Pyricularia oryzae 70-15]
MGG_10251	8498.557606	1856.304814	0.218425867	-2.194784378	0.002506862	0.02388884	uncharacterized protein MGG_10251 [Pyricularia oryzae 70-15]
MGG_10252	66534.69114	23461.75662	0.352624416	-1.503795723	0.002863127	0.026429536	uncharacterized protein MGG_10252 [Pyricularia oryzae 70-15]
MGG_10253	12372.00149	3467.849802	0.280298204	-1.834965592	5.99236E-05	0.001223449	uncharacterized protein MGG_10253 [Pyricularia oryzae 70-15]
MGG_10255	16.89572649	117.9577991	6.981516843	2.803540518	0.000773953	0.009574325	uncharacterized protein MGG_10255 [Pyricularia oryzae 70-15]
MGG_10257	25.06457075	8.493758579	0.338875086	-1.561174519	0.026137646	0.12565102	uncharacterized protein MGG_10257 [Pyricularia oryzae 70-15]
MGG_10259	15.44168836	49.83144674	3.227072427	1.690225958	0.002896132	0.026612866	uncharacterized protein MGG_10259 [Pyricularia oryzae 70-15]
MGG_10274	44.53177924	372.9755224	8.37549114	3.066173793	1.23273E-05	0.000325474	uncharacterized protein MGG_10274 [Pyricularia oryzae 70-15]
MGG_10278	3.295494349	35.35049821	10.72691817	3.423163746	3.01044E-06	9.6433E-05	uncharacterized protein MGG_10278 [Pyricularia oryzae 70-15]
MGG_10279	3.772145332	27.01531847	7.161791526	2.840320523	0.000247161	0.003936071	uncharacterized protein MGG_10279 [Pyricularia oryzae 70-15]

MGG_10292	3356.895822	12717.1542	3.788367251	1.921576195	0.000909325	0.01089396	uncharacterized protein MGG_10292 [Pyricularia oryzae 70-15]
MGG_10311	29.90873855	11.37647694	0.380373011	-1.39451321	0.027966799	0.131529491	uncharacterized protein MGG_10311 [Pyricularia oryzae 70-15]
MGG_10319	527.76258	1664.190411	3.153293685	1.656859543	0.000542063	0.0072283	uncharacterized protein MGG_10319 [Pyricularia oryzae 70-15]
MGG_10321	316.4116629	959.2536305	3.031663314	1.600109542	0.030272146	0.139087002	siroheme synthase [Pyricularia oryzae 70-15]
MGG_10327	37445.78822	11556.03981	0.308607199	-1.696156379	0.006410712	0.048120712	uncharacterized protein MGG_10327 [Pyricularia oryzae 70-15]
MGG_10331	47.76316943	97.33527466	2.037873027	1.027064165	0.032212101	0.145145625	uncharacterized protein MGG_10331 [Pyricularia oryzae 70-15]
MGG_10357	4097.63812	8591.565866	2.096711719	1.068128516	0.01170064	0.072483108	prolyl-tRNA synthetase [Pyricularia oryzae 70-15]
MGG_10358	1956.151669	4956.126093	2.533610338	1.341194659	0.000960988	0.011411609	T-complex protein 1 subunit alpha [Pyricularia oryzae 70-15]
MGG_10359	4308.640154	1282.333326	0.297619035	-1.748461292	1.96036E-05	0.000478076	uncharacterized protein MGG_10359 [Pyricularia oryzae 70-15]
MGG_10360	219.6227071	550.6312413	2.507168992	1.326059242	0.015434555	0.087006487	haloacid dehalogenase [Pyricularia oryzae 70-15]
MGG_10361	31.91650667	1.902001275	0.059593028	-4.068712636	7.14697E-06	0.000208205	uncharacterized protein MGG_10361 [Pyricularia oryzae 70-15]
MGG_10409	95.72207484	40.43704228	0.422442183	-1.243174192	0.010687695	0.068226023	uncharacterized protein MGG_10409 [Pyricularia oryzae 70-15]
MGG_10414	853.0615885	366.6161128	0.429765116	-1.21837971	0.00701654	0.051040078	cholinesterase [Pyricularia oryzae 70-15]
MGG_10422	77.39166752	33.37495344	0.431247375	-1.213412421	0.015022078	0.085715458	uncharacterized protein MGG_10422 [Pyricularia oryzae 70-15]
MGG_10423	31.44681712	89.1619008	2.835323539	1.503513371	0.002737451	0.025583964	cellulase [Pyricularia oryzae 70-15]
MGG_10424	8.103792097	31.79877867	3.923938113	1.972302288	0.003814585	0.032450683	uncharacterized protein MGG_10424 [Pyricularia oryzae 70-15]
MGG_10432	53.68527757	382.79354	7.130326178	2.833968074	1.95835E-05	0.000478076	uncharacterized protein MGG_10432 [Pyricularia oryzae 70-15]
MGG_10436	1.656493569	8.938487666	5.396029197	2.431898155	0.044630572	0.179765313	uncharacterized protein MGG_10436 [Pyricularia oryzae 70-15]
MGG_10438	28.9936866	107.8677156	3.72038634	1.895452445	0.000140688	0.002469764	uncharacterized protein MGG_10438 [Pyricularia oryzae 70-15]
MGG_10439	249.0636207	981.5311129	3.940885105	1.978519689	2.78103E-06	9.05735E-05	MFS transporter [Pyricularia oryzae 70-15]
MGG_10440	657.1269124	1795.687345	2.732634003	1.450292245	0.006151521	0.046444176	endoribonuclease L-PSP [Pyricularia oryzae 70-15]
MGG_10446	312.8658294	735.2837486	2.350156775	1.232757	0.002962495	0.027079303	DNA-directed RNA polymerase III subunit RPC6
MGG_10449	0.321175942	14.45578691	45.00893441	5.492139504	0.006967406	0.050804353	proteinase T [Pyricularia oryzae 70-15]
MGG_10452	22.04653638	154.0272808	6.986461641	2.804561974	2.19945E-06	7.3606E-05	uncharacterized protein MGG_10452 [Pyricularia oryzae 70-15]
MGG_10453	450.7210037	1384.419457	3.071566326	1.618974536	0.012598679	0.076333542	L-lactate dehydrogenase [Pyricularia oryzae 70-15]
MGG_10456	64.65281897	440.7341512	6.816936341	2.769123511	8.4861E-10	6.65091E-08	uncharacterized protein MGG_10456 [Pyricularia oryzae 70-15]
MGG_10460	4.855278142	94.88231937	19.54209761	4.288513426	2.82243E-12	3.57155E-10	uncharacterized protein MGG_10460 [Pyricularia oryzae 70-15]
MGG_10463	2549.335337	587.2580419	0.230357314	-2.118054687	5.56068E-07	2.21479E-05	uncharacterized protein MGG_10463 [Pyricularia oryzae 70-15]
MGG_10467	35.03457878	89.68918316	2.560018881	1.356154451	0.006720658	0.049360672	uncharacterized protein MGG_10467 [Pyricularia oryzae 70-15]
MGG_10468	3.368118939	35.46980483	10.53104284	3.396576402	6.16716E-06	0.000181841	uncharacterized protein MGG_10468 [Pyricularia oryzae 70-15]
MGG_10469	186.0714121	415.4315054	2.232645524	1.158754213	0.00657155	0.048736988	uncharacterized protein MGG_10469 [Pyricularia oryzae 70-15]
MGG_10480	1965.068936	589.7951017	0.30013965	-1.736294178	2.74696E-05	0.000636704	uncharacterized protein MGG_10480 [Pyricularia oryzae 70-15]
MGG_10485	682.286151	331.5151789	0.48588877	-1.041302005	0.010603059	0.067877241	uncharacterized protein MGG_10485 [Pyricularia oryzae 70-15]
MGG_10487	8961.191816	4409.380957	0.49205296	-1.023114493	0.010562047	0.067682314	uncharacterized protein MGG_10487 [Pyricularia oryzae 70-15]
MGG_10491	685.3426139	332.2429574	0.484783743	-1.044586776	0.010185399	0.066137767	uncharacterized protein MGG_10491 [Pyricularia oryzae 70-15]
MGG_10492	1548.987323	4082.739558	2.635747561	1.398212203	0.000448747	0.006245594	carnitine/acyl carnitine carrier [Pyricularia oryzae 70-15]
MGG_10496	668.1767263	1588.404563	2.377222224	1.249276774	0.00639893	0.048073097	diphthine synthase [Pyricularia oryzae 70-15]
MGG_10497	4926.745812	14520.23624	2.947226586	1.559357981	0.013358573	0.07939039	bli-3 [Pyricularia oryzae 70-15]
MGG_10510	24.08297116	80.97537163	3.362349732	1.749469793	0.00066451	0.008479477	ribonuclease T2 [Pyricularia oryzae 70-15]
MGG_10514	1901.806833	7715.267073	4.056808998	2.02034538	7.80145E-07	3.00864E-05	WSC domain-containing protein [Pyricularia oryzae 70-15]
MGG_10519	93.63508973	39.97211572	0.42689248	-1.228055344	0.010415643	0.067053114	maltose permease MAL31 [Pyricularia oryzae 70-15]
MGG_10527	8.985353949	0.34480135	0.038373708	-4.703738008	0.002560782	0.024204747	ent-kaurene oxidase [Pyricularia oryzae 70-15]
MGG_10530	517.4557742	197.6438754	0.381953174	-1.388532314	0.036934585	0.157868822	sugar transporter [Pyricularia oryzae 70-15]
MGG_10533	50.04571207	1183.852928	23.65543178	4.56409959	2.63187E-18	1.06573E-15	agmatinase 1 [Pyricularia oryzae 70-15]
MGG_10534	190.585451	538.3155343	2.824536351	1.498014068	0.020576017	0.106319632	multidrug and toxin extrusion protein 1 [Pyricularia oryzae 70-15]
MGG_10549	299.3764078	1055.334923	3.525110516	1.817668488	0.000155002	0.002670693	uncharacterized protein MGG_10549 [Pyricularia oryzae 70-15]
MGG_10555	2.032801311	21.04628478	10.35334081	3.372024466	0.007441901	0.053212624	uncharacterized protein MGG_10555 [Pyricularia oryzae 70-15]
MGG_10557	14.93643425	105.7550752	7.080342833	2.823819218	3.26046E-05	0.000740338	uncharacterized protein MGG_10557 [Pyricularia oryzae 70-15]

MGG_10558	158.3528465	330.9291148	2.089821069	1.063379423	0.016669459	0.09196212	uncharacterized protein MGG_10558 [Pyricularia oryzae 70-15]
MGG_10563	48.00839282	103.3532249	2.152815765	1.106224861	0.021927992	0.111212671	efflux pump [Pyricularia oryzae 70-15]
MGG_10564	0.71134301	34.11249719	47.95506067	5.583611165	6.35056E-09	4.08183E-07	uncharacterized protein MGG_10564 [Pyricularia oryzae 70-15]
MGG_10565	2827.014078	6832.056954	2.416704256	1.273040934	0.02380327	0.117450093	zuotin [Pyricularia oryzae 70-15]
MGG_10570	148.7979752	371.5058136	2.49671283	1.320029893	0.002259577	0.022172328	uncharacterized protein MGG_10570 [Pyricularia oryzae 70-15]
MGG_10575	1023.460604	2232.508034	2.181332652	1.125209797	0.005103783	0.040365072	uncharacterized protein MGG_10575 [Pyricularia oryzae 70-15]
MGG_10589	49.34282597	10.14714474	0.205645796	-2.281766514	0.000101867	0.001912637	uncharacterized protein MGG_10589 [Pyricularia oryzae 70-15]
MGG_10593	51.42889457	104.8882597	2.039481125	1.028202155	0.02767662	0.130721453	uncharacterized protein MGG_10593 [Pyricularia oryzae 70-15]
MGG_10598	639.1518529	284.2303916	0.444699316	-1.16909791	0.005291979	0.041503884	uncharacterized protein MGG_10598 [Pyricularia oryzae 70-15]
MGG_10600	202.4972905	71.15595792	0.351392148	-1.50884614	0.000701328	0.00886549	uncharacterized protein MGG_10600 [Pyricularia oryzae 70-15]
MGG_10613	7.06193176	34.17237092	4.838955129	2.274695561	0.00086154	0.010446732	retinol dehydrogenase 11 [Pyricularia oryzae 70-15]
MGG_10619	489.6166932	1307.907244	2.671288097	1.417535578	0.008457533	0.057915509	uncharacterized protein MGG_10619 [Pyricularia oryzae 70-15]
MGG_10627	389.1181373	187.4608222	0.48175812	-1.053619112	0.010852493	0.068807974	ATP-dependent DNA ligase domain-containing protein
MGG_10637	567.8735048	1139.282703	2.0062262	1.004484277	0.014679442	0.084554697	uncharacterized protein MGG_10637 [Pyricularia oryzae 70-15]
MGG_10640	16.18075002	53.46860565	3.304457803	1.724413573	0.002141209	0.021216479	interferon-induced GTP-binding protein Mx
MGG_10648	205.9894175	29.0862046	0.141202422	-2.824163262	0.000136473	0.002402719	uncharacterized protein MGG_10648 [Pyricularia oryzae 70-15]
MGG_10659	4157.047053	1971.369365	0.474223491	-1.076360965	0.007178219	0.051874483	uncharacterized protein MGG_10659 [Pyricularia oryzae 70-15]
MGG_10660	24490.83914	11539.59962	0.471180246	-1.085649038	0.012909035	0.077710088	uncharacterized protein MGG_10660 [Pyricularia oryzae 70-15]
MGG_10661	940.717994	293.0888238	0.311558645	-1.682424345	0.001971951	0.020039142	uncharacterized protein MGG_10661 [Pyricularia oryzae 70-15]
MGG_10679	45.897259	19.65344642	0.428205232	-1.223625673	0.028614193	0.133284207	uncharacterized protein MGG_10679 [Pyricularia oryzae 70-15]
MGG_10683	69.50571716	1048.899901	15.09084352	3.915601544	0.002920613	0.026797288	uncharacterized protein MGG_10683 [Pyricularia oryzae 70-15]
MGG_10685	3401.230663	1346.509379	0.395888874	-1.336832571	0.000828796	0.010139191	uncharacterized protein MGG_10685 [Pyricularia oryzae 70-15]
MGG_10696	404.0681574	933.1245476	2.309324629	1.207470991	0.014389591	0.083399211	uncharacterized protein MGG_10696 [Pyricularia oryzae 70-15]
MGG_10697	385.6357202	1900.523994	4.928288264	2.301086644	4.90311E-08	2.55635E-06	uncharacterized protein MGG_10697 [Pyricularia oryzae 70-15]
MGG_10705	3.841136457	17.03127586	4.433915861	2.148581394	0.010608847	0.067877241	uncharacterized protein MGG_10705 [Pyricularia oryzae 70-15]
MGG_10712	5131.828022	1166.366739	0.227280948	-2.137451339	0.032521109	0.145942531	cellulose 1,4-beta-cellobiosidase [Pyricularia oryzae 70-15]
MGG_10725	0	3.87584577	Inf	Inf	0.047834975	0.187390931	uncharacterized protein MGG_10725 [Pyricularia oryzae 70-15]
MGG_10730	58130.43559	8623.513875	0.148347656	-2.752945962	0.001233537	0.013952525	potassium/sodium efflux P-type ATPase
MGG_10733	5.098121574	18.11936688	3.554126086	1.829494863	0.023598274	0.11681819	uncharacterized protein MGG_10733 [Pyricularia oryzae 70-15]
MGG_10740	701.3507015	1624.040404	2.315589619	1.211379594	0.003046551	0.027577872	sm snRNP core protein Smg1 [Pyricularia oryzae 70-15]
MGG_10745	756.2749748	347.8014236	0.459887521	-1.120647043	0.0062535	0.047097035	monooxygenase [Pyricularia oryzae 70-15]
MGG_10750	202.9774921	749.6732315	3.693381093	1.884942131	1.18053E-05	0.000314497	oligopeptide transporter 2 [Pyricularia oryzae 70-15]
MGG_10751	157.8641051	803.4016662	5.089197862	2.347438283	6.55938E-08	3.23602E-06	peroxisomal copper amine oxidase [Pyricularia oryzae 70-15]
MGG_10764	1.032518952	18.49473481	17.91224731	4.162874447	6.87892E-05	0.001358782	uncharacterized protein MGG_10764 [Pyricularia oryzae 70-15]
MGG_10785	20.05745103	81.05173814	4.040978987	2.014704849	0.000132252	0.002345391	meiotic chromosome segregation protein
MGG_10791	336.5596003	751.8399382	2.233898357	1.159563544	0.004823469	0.038753642	uncharacterized protein MGG_10791 [Pyricularia oryzae 70-15]
MGG_10801	0	14.50947388	Inf	Inf	0.016323875	0.09057049	gluconolactonase [Pyricularia oryzae 70-15]
MGG_10805	2.014424045	11.66058596	5.788545858	2.533200974	0.046848328	0.185017388	uncharacterized protein MGG_10805 [Pyricularia oryzae 70-15]
MGG_10814	4.386778565	23.87409354	5.442283714	2.444212168	0.001702626	0.017861395	D-3-phosphoglycerate dehydrogenase [Pyricularia oryzae 70-15]
MGG_10816	6.850440443	26.08536545	3.80783771	1.928971992	0.008434605	0.057791077	quinate permease [Pyricularia oryzae 70-15]
MGG_10817	15.5567754	63.99778273	4.113820575	2.040478872	0.008588457	0.058461047	4-hydroxy-2-oxovalerate aldolase [Pyricularia oryzae 70-15]
MGG_10834	2166.092538	1069.746034	0.493859803	-1.017826548	0.012153114	0.074355367	methylenetetrahydrofolate dehydrogenase
MGG_10846	7.866351083	42.39191193	5.389018553	2.430022553	0.000113987	0.002109784	uncharacterized protein MGG_10846 [Pyricularia oryzae 70-15]
MGG_10856	119.6966022	753.0962014	6.2917091	2.653451969	0.000115801	0.002128503	uncharacterized protein MGG_10856 [Pyricularia oryzae 70-15]
MGG_10865	282.346059	1277.527696	4.524687542	2.177818168	2.36215E-07	1.00333E-05	uncharacterized protein MGG_10865 [Pyricularia oryzae 70-15]
MGG_10867	522.3697602	1141.494741	2.185223624	1.127780925	0.005725928	0.044080212	zinc-regulated transporter 1 [Pyricularia oryzae 70-15]
MGG_10877	107.7003979	28.31027032	0.262861334	-1.927626151	0.00010123	0.00190363	uncharacterized protein MGG_10877 [Pyricularia oryzae 70-15]
MGG_10878	209.7235421	38.21828736	0.182231747	-2.456153782	1.83009E-06	6.42541E-05	galactose oxidase [Pyricularia oryzae 70-15]

MGG_10893	41727.02508	7812.974574	0.187240153	-2.417038243	6.58188E-05	0.001312919	uncharacterized protein MGG_10893 [Pyricularia oryzae 70-15]
MGG_10896	62.0253207	249.5672033	4.023634227	2.008499161	1.03867E-05	0.000284185	uncharacterized protein MGG_10896 [Pyricularia oryzae 70-15]
MGG_10910	1310.705193	3914.625613	2.986656064	1.57853111	0.021727858	0.110621014	aflatoxin biosynthesis ketoreductase nor-1
MGG_10911	201.426458	850.6686782	4.223222145	2.078344136	0.027453413	0.129970404	cupin domain-containing protein [Pyricularia oryzae 70-15]
MGG_10921	1273.707847	4023.895744	3.159198361	1.659558524	4.3917E-05	0.000940923	uncharacterized protein MGG_10921 [Pyricularia oryzae 70-15]
MGG_10927	157.9924725	77.34974136	0.48957865	-1.030387451	0.036434724	0.156620323	metallo-endopeptidase [Pyricularia oryzae 70-15]
MGG_10941	3460.397746	1643.024	0.474807846	-1.07458432	0.033103023	0.147994011	cation-transporting ATPase pacS [Pyricularia oryzae 70-15]
MGG_10942	794.2801174	2603.972993	3.278406365	1.712994691	3.73069E-05	0.000825508	peptidase [Pyricularia oryzae 70-15]
MGG_10961	1221.264074	2749.601919	2.25143929	1.170847576	0.003339197	0.029246257	uncharacterized protein MGG_10961 [Pyricularia oryzae 70-15]
MGG_10969	3529.464324	1218.610722	0.345267896	-1.5342119	0.000165005	0.002807401	kynureninase [Pyricularia oryzae 70-15]
MGG_10975	65.83126163	148.0077871	2.248290303	1.168828331	0.034054507	0.150161218	uncharacterized protein MGG_10975 [Pyricularia oryzae 70-15]
MGG_11005	32.53535235	99.9304729	3.071442775	1.618916504	0.001145015	0.013109935	uncharacterized protein MGG_11005 [Pyricularia oryzae 70-15]
MGG_11036	1150.86879	508.489276	0.441830798	-1.178434109	0.00316718	0.028276654	cellobiose dehydrogenase [Pyricularia oryzae 70-15]
MGG_11072	62.4581922	10.9647965	0.175554177	-2.510011776	0.002517099	0.02388884	uncharacterized protein MGG_11072 [Pyricularia oryzae 70-15]
MGG_11084	926.334373	4253.355868	4.591598879	2.198996614	1.30128E-07	5.98784E-06	TPR domain-containing protein [Pyricularia oryzae 70-15]
MGG_11091	69.31770002	30.75444635	0.443673785	-1.172428782	0.031912156	0.144005507	uncharacterized protein MGG_11091 [Pyricularia oryzae 70-15]
MGG_11096	1.083132811	9.463765608	8.737400913	3.12720419	0.017996123	0.09703369	uncharacterized protein MGG_11096 [Pyricularia oryzae 70-15]
MGG_11129	971.7086503	481.1447839	0.49515334	-1.014052724	0.014143122	0.082442729	caffeine-induced death protein 1 [Pyricularia oryzae 70-15]
MGG_11148	4710.502499	1128.241649	0.239516198	-2.061804869	0.000120638	0.002184063	uncharacterized protein MGG_11148 [Pyricularia oryzae 70-15]
MGG_11160	911.2817257	433.3719961	0.475563137	-1.072291205	0.007658665	0.054186061	uncharacterized protein MGG_11160 [Pyricularia oryzae 70-15]
MGG_11168	563.0770749	273.0621611	0.48494633	-1.044103004	0.011199256	0.070418507	uncharacterized protein MGG_11168 [Pyricularia oryzae 70-15]
MGG_11179	77.73268418	35.9859731	0.462945201	-1.111086663	0.028024901	0.131598953	uncharacterized protein MGG_11179 [Pyricularia oryzae 70-15]
MGG_11188	4402.517165	1114.025068	0.253042754	-1.982546932	1.557E-06	5.53055E-05	uncharacterized protein MGG_11188 [Pyricularia oryzae 70-15]
MGG_11196	835.271911	386.9855093	0.463304828	-1.109966377	0.006808951	0.049884092	origin recognition complex subunit 4 [Pyricularia oryzae 70-15]
MGG_11214	54.45361689	17.98264899	0.330237917	-1.598422321	0.005388305	0.042052933	uncharacterized protein MGG_11214 [Pyricularia oryzae 70-15]
MGG_11267	434.8992022	202.0329928	0.464551307	-1.106090152	0.00800918	0.055724809	tetrahydrofolylpolyglutamate synthase [Pyricularia oryzae 70-15]
MGG_11269	443.9941452	202.6048525	0.456323253	-1.131871924	0.007044732	0.051180845	uncharacterized protein MGG_11269 [Pyricularia oryzae 70-15]
MGG_11274	2037.219978	197.9945489	0.097188596	-3.363069154	7.05116E-12	8.15786E-10	uncharacterized protein MGG_11274 [Pyricularia oryzae 70-15]
MGG_11285	53.7141862	378.6596285	7.049527421	2.817526547	6.12607E-05	0.001244472	uncharacterized protein MGG_11285 [Pyricularia oryzae 70-15]
MGG_11289	608.8935573	2842.613859	4.668490618	2.222956184	8.8059E-08	4.21158E-06	uncharacterized protein MGG_11289 [Pyricularia oryzae 70-15]
MGG_11292	0	10.12776586	Inf	Inf	0.000297326	0.004560493	uncharacterized protein MGG_11292 [Pyricularia oryzae 70-15]
MGG_11300	92.10027561	567.6164131	6.163026217	2.623638928	0.000690696	0.008740182	uncharacterized protein MGG_11300 [Pyricularia oryzae 70-15]
MGG_11311	2316.632352	717.538762	0.30973355	-1.690900433	0.005327604	0.041727744	uncharacterized protein MGG_11311 [Pyricularia oryzae 70-15]
MGG_11312	261.6147764	101.9101369	0.38954274	-1.360146466	0.001803734	0.01869604	uncharacterized protein MGG_11312 [Pyricularia oryzae 70-15]
MGG_11377	624.8509682	1337.377477	2.140314324	1.097822684	0.007226335	0.052191153	mitochondrial 37S ribosomal protein S8
MGG_11409	253.5664216	80.96870722	0.319319517	-1.646927362	0.000185283	0.003091783	uncharacterized protein MGG_11409 [Pyricularia oryzae 70-15]
MGG_11422	432.6487371	108.6364342	0.251096155	-1.99368816	4.08982E-06	0.000126743	uncharacterized protein MGG_11422 [Pyricularia oryzae 70-15]
MGG_11467	255.8525215	522.5022339	2.042200838	1.030124753	0.009274382	0.06180208	uncharacterized protein MGG_11467 [Pyricularia oryzae 70-15]
MGG_11475	4933.750612	2140.47644	0.433843664	-1.204752837	0.002356468	0.022791698	uncharacterized protein MGG_11475 [Pyricularia oryzae 70-15]
MGG_11492	266.4673564	43.94500313	0.164917023	-2.600187772	0.000318995	0.004811106	uncharacterized protein MGG_11492 [Pyricularia oryzae 70-15]
MGG_11495	11.18385616	1.825193923	0.16319898	-2.61529605	0.019535237	0.102866951	uncharacterized protein MGG_11495 [Pyricularia oryzae 70-15]
MGG_11530	2545.061813	765.3155961	0.300706094	-1.733573991	2.21836E-05	0.000528405	uncharacterized protein MGG_11530 [Pyricularia oryzae 70-15]
MGG_11541	1833.500207	3698.60607	2.01723788	1.012381222	0.011798301	0.072962907	phosphoribosylformylglycinamidine synthase
MGG_11543	1702.806245	7460.371854	4.381221807	2.131333255	0.000218468	0.003557403	lactoylglutathione lyase [Pyricularia oryzae 70-15]
MGG_11544	9.131487605	0.640879865	0.070183511	-3.832724059	0.005535525	0.042982981	uncharacterized protein MGG_11544 [Pyricularia oryzae 70-15]
MGG_11554	503.2135018	182.6211194	0.362909816	-1.462317015	0.000495942	0.006739036	seed imbibition protein [Pyricularia oryzae 70-15]
MGG_11582	193.0440795	56.69812978	0.293705613	-1.767557256	8.32794E-05	0.001603294	O-methyltransferase [Pyricularia oryzae 70-15]
MGG_11589	593.0188709	1776.174987	2.995140751	1.582623802	0.000303154	0.004620717	uncharacterized protein MGG_11589 [Pyricularia oryzae 70-15]

MGG_11608	8.85188964	99.21312663	11.20812964	3.486473643	9.11743E-10	7.05469E-08	laccase-2 [Pyricularia oryzae 70-15]
MGG_11610	115.8214094	434.1197371	3.748182132	1.906191058	0.004178173	0.034764688	uncharacterized protein MGG_11610 [Pyricularia oryzae 70-15]
MGG_11617	18.11113363	313.574143	17.31388821	4.113857844	2.96672E-16	8.38132E-14	uncharacterized protein MGG_11617 [Pyricularia oryzae 70-15]
MGG_11636	3207.897902	563.2201994	0.175572982	-2.509857245	0.024576768	0.120047678	serine/threonine protein kinase [Pyricularia oryzae 70-15]
MGG_11638	152.5010659	58.21660875	0.381745586	-1.389316619	0.00251692	0.02388884	lovastatin nonaketide synthase [Pyricularia oryzae 70-15]
MGG_11643	14886.116	5743.480218	0.38582799	-1.373970286	0.000673297	0.008564614	chromatin structure-remodeling complex protein RSC7
MGG_11657	2199.17542	1080.493069	0.491317363	-1.02527287	0.014568285	0.084193875	uncharacterized protein MGG_11657 [Pyricularia oryzae 70-15]
MGG_11682	246.066312	762.6531042	3.099380399	1.631979833	0.000105163	0.001962394	phenylacetone monooxygenase [Pyricularia oryzae 70-15]
MGG_11683	20.44340566	63.07853977	3.085520134	1.625513709	0.002646014	0.024895627	metallo-beta-lactamase superfamily protein
MGG_11691	12.06541802	34.48908775	2.858507488	1.51526207	0.012929697	0.077795919	streptogrisin-C [Pyricularia oryzae 70-15]
MGG_11697	45.68998012	612.2781158	13.40070874	3.744237399	1.13165E-15	2.74947E-13	carbamoylphosphate synthase large subunit
MGG_11719	19.71337988	45.55629405	2.310932693	1.208475241	0.034034516	0.150161218	uncharacterized protein MGG_11719 [Pyricularia oryzae 70-15]
MGG_11749	115.7016259	256.5624701	2.217449133	1.14890101	0.011099454	0.069935773	uncharacterized protein MGG_11749 [Pyricularia oryzae 70-15]
MGG_11750	4013.517748	10329.816	2.573756153	1.363875374	0.000669995	0.00853155	uncharacterized protein MGG_11750 [Pyricularia oryzae 70-15]
MGG_11754	8490.765536	2342.265087	0.275860295	-1.857990272	4.83183E-06	0.00014711	SMDR1 [Pyricularia oryzae 70-15]
MGG_11770	841.7397248	343.115466	0.407626557	-1.294680048	0.001489892	0.016031187	uncharacterized protein MGG_11770 [Pyricularia oryzae 70-15]
MGG_11771	922.5577164	2318.44223	2.51305928	1.329444703	0.000978949	0.01157705	ubiquitin carboxyl-terminal hydrolase 2 [Pyricularia oryzae 70-15]
MGG_11781	239.256132	43.72569515	0.182756842	-2.452002675	1.06336E-07	5.02634E-06	uncharacterized protein MGG_11781 [Pyricularia oryzae 70-15]
MGG_11927	135.1874216	458.1312461	3.388860004	1.760800039	0.041382079	0.171047802	3-oxoacyl-[acyl-carrier-protein] reductase
MGG_11934	425.6223063	1767.186893	4.15200723	2.053808956	7.66472E-07	2.96532E-05	acetylornithine aminotransferase [Pyricularia oryzae 70-15]
MGG_11952	0.743579602	9.485626555	12.75670625	3.673183972	0.030045797	0.138361011	uncharacterized protein MGG_11952 [Pyricularia oryzae 70-15]
MGG_11962	338.5911608	6662.990267	19.67857121	4.29855357	3.08876E-21	1.78677E-18	G-protein coupled receptor [Pyricularia oryzae 70-15]
MGG_11991	652.1488483	262.703769	0.402827928	-1.311764388	0.001465652	0.015812381	uncharacterized protein MGG_11991 [Pyricularia oryzae 70-15]
MGG_12022	143.8089593	69.81898445	0.485498155	-1.042462281	0.032484768	0.145869337	uncharacterized protein MGG_12022 [Pyricularia oryzae 70-15]
MGG_12029	77.94967347	30.09544704	0.386088173	-1.372997732	0.006220922	0.046909847	uncharacterized protein MGG_12029 [Pyricularia oryzae 70-15]
MGG_12043	435.9517398	30.51005039	0.069984926	-3.836811969	8.03504E-16	1.99203E-13	uncharacterized protein MGG_12043 [Pyricularia oryzae 70-15]
MGG_12098	1823.236933	3777.677401	2.071961868	1.050997452	0.00956281	0.06308753	orotidine 5'-phosphate decarboxylase [Pyricularia oryzae 70-15]
MGG_12108	13213.48684	3235.154085	0.244837273	-2.03010489	0.002869876	0.02643158	leucine Rich Repeat domain-containing protein
MGG_12111	3165.403642	1415.76593	0.447262368	-1.160806717	0.003946843	0.033272467	uncharacterized protein MGG_12111 [Pyricularia oryzae 70-15]
MGG_12174	2494.286394	5162.523474	2.069739661	1.049449312	0.023143625	0.115286263	uncharacterized protein MGG_12174 [Pyricularia oryzae 70-15]
MGG_12175	28463.40508	7943.474911	0.279076761	-1.841266098	0.00395448	0.033291075	gramicidin S synthetase 1 [Pyricularia oryzae 70-15]
MGG_12213	851.4584772	347.5056799	0.40812992	-1.292899618	0.00143134	0.015594548	HET domain-containing protein [Pyricularia oryzae 70-15]
MGG_12222	244.6543862	119.4055576	0.488058111	-1.034875161	0.047517577	0.18687068	F-box domain-containing protein [Pyricularia oryzae 70-15]
MGG_12230	8.664178007	1692.932989	195.3945299	7.610246269	1.32486E-06	4.80432E-05	uncharacterized protein MGG_12230 [Pyricularia oryzae 70-15]
MGG_12231	1.404308753	209.8805559	149.454709	7.223564543	4.99002E-13	7.67326E-11	allantoate permease [Pyricularia oryzae 70-15]
MGG_12243	769.5156793	1712.922213	2.225974413	1.15443701	0.004777841	0.03853998	uncharacterized protein MGG_12243 [Pyricularia oryzae 70-15]
MGG_12267	803.3802276	1791.597615	2.23007432	1.157091791	0.004562626	0.037199178	FAD synthetase [Pyricularia oryzae 70-15]
MGG_12304	654.6807444	231.0189963	0.352872753	-1.50278006	0.000300477	0.004591438	uncharacterized protein MGG_12304 [Pyricularia oryzae 70-15]
MGG_12339	405.2379841	166.792618	0.411591767	-1.28071397	0.002035244	0.020500946	uncharacterized protein MGG_12339 [Pyricularia oryzae 70-15]
MGG_12383	6.764841006	144.1052898	21.3020956	4.412923458	0.000146056	0.002541968	uncharacterized protein MGG_12383 [Pyricularia oryzae 70-15]
MGG_12392	803.8296692	293.079247	0.36460367	-1.455599014	0.002884529	0.02652631	pentafunctional AROM polypeptide [Pyricularia oryzae 70-15]
MGG_12423	222.0413487	41.02919931	0.184781797	-2.436105456	0.003838024	0.03260442	choline monooxygenase [Pyricularia oryzae 70-15]
MGG_12441	64.26088295	633.1325119	9.852533653	3.300494772	9.83405E-10	7.4665E-08	uncharacterized protein MGG_12441 [Pyricularia oryzae 70-15]
MGG_12442	411.4040616	1858.330296	4.517044116	2.175379004	2.03514E-07	8.83919E-06	aminopeptidase Y [Pyricularia oryzae 70-15]
MGG_12444	4.465111075	15.15858173	3.394894657	1.763366809	0.042144753	0.173148401	uncharacterized protein MGG_12444 [Pyricularia oryzae 70-15]
MGG_12467	3032.822967	6626.899577	2.18505981	1.12767277	0.043058619	0.175293601	uncharacterized protein MGG_12467 [Pyricularia oryzae 70-15]
MGG_12473	11620.43567	3354.53287	0.28867531	-1.792480374	0.001972902	0.020039142	nitrogen metabolic regulation protein [Pyricularia oryzae 70-15]
MGG_12476	117.5587104	288.3832735	2.453100009	1.294606051	0.002748582	0.025663841	uncharacterized protein MGG_12476 [Pyricularia oryzae 70-15]

MGG_12478	0.321175942	5.43056363	16.90837612	4.079666205	0.038536163	0.162604136	P450 monooxygenase [Pyricularia oryzae 70-15]
MGG_12484	160.0217868	744.8793213	4.654861918	2.218738372	1.68429E-07	7.4948E-06	uncharacterized protein MGG_12484 [Pyricularia oryzae 70-15]
MGG_12489	227.986689	52.20085664	0.228964493	-2.126804208	0.016841191	0.092657057	beta-xylosidase [Pyricularia oryzae 70-15]
MGG_12491	3.996611497	14.48339378	3.623918359	1.857550454	0.045583768	0.182035377	uncharacterized protein MGG_12491 [Pyricularia oryzae 70-15]
MGG_12492	16.70801486	103.0688117	6.168824519	2.624995607	6.21502E-07	2.4513E-05	uncharacterized protein MGG_12492 [Pyricularia oryzae 70-15]
MGG_12496	0.963527826	12.89610492	13.3842579	3.742465245	0.010540599	0.067678224	cytochrome P450 52A2 [Pyricularia oryzae 70-15]
MGG_12501	2033.363011	4919.266688	2.419276175	1.274575471	0.04169784	0.171943434	uncharacterized protein MGG_12501 [Pyricularia oryzae 70-15]
MGG_12536	1373.731993	494.6569577	0.360082578	-1.473600295	0.000277391	0.004325735	uncharacterized protein MGG_12536 [Pyricularia oryzae 70-15]
MGG_12568	12.34776497	2.442953403	0.197845797	-2.337551676	0.028932922	0.134510957	serine/threonine protein kinase [Pyricularia oryzae 70-15]
MGG_12569	1433.812713	3367.038158	2.348310995	1.231623482	0.045799911	0.182658346	uncharacterized protein MGG_12569 [Pyricularia oryzae 70-15]
MGG_12599	178.7897106	84.41794334	0.47216332	-1.082642125	0.013567411	0.080202877	uncharacterized protein MGG_12599 [Pyricularia oryzae 70-15]
MGG_12613	117.6376539	44.03503941	0.374327759	-1.417626056	0.005295606	0.041503884	polyketide synthase [Pyricularia oryzae 70-15]
MGG_12631	2431.497963	1206.892097	0.496357437	-1.010548686	0.011422594	0.071269476	uncharacterized protein MGG_12631 [Pyricularia oryzae 70-15]
MGG_12632	404.0994842	135.0395288	0.33417397	-1.581328735	0.000861153	0.010446732	uncharacterized protein MGG_12632 [Pyricularia oryzae 70-15]
MGG_12654	8.98987189	58.62665199	6.521411285	2.705184209	0.000120599	0.002184063	uncharacterized protein MGG_12654 [Pyricularia oryzae 70-15]
MGG_12655	37.07849043	140.9403301	3.801134526	1.926430085	4.96532E-05	0.001041774	uncharacterized protein MGG_12655 [Pyricularia oryzae 70-15]
MGG_12678	200.7383793	470.9577578	2.346127131	1.230281191	0.003398511	0.029595061	glucan endo-1,3-alpha-glucosidase agn1
MGG_12679	3.584433699	20.98641105	5.854874943	2.549638356	0.001552702	0.016604073	uncharacterized protein MGG_12679 [Pyricularia oryzae 70-15]
MGG_12691	151.8932985	41.15268827	0.27093156	-1.883999638	5.55341E-05	0.001147327	uncharacterized protein MGG_12691 [Pyricularia oryzae 70-15]
MGG_12696	26.9220563	89.40262888	3.32079496	1.731528647	0.000643038	0.008257529	cellulose-growth-specific protein [Pyricularia oryzae 70-15]
MGG_12707	991.452542	416.2168491	0.419805116	-1.252208348	0.001940454	0.019808939	alpha/beta hydrolase fold protein [Pyricularia oryzae 70-15]
MGG_12733	668.4868945	117.761218	0.176160848	-2.505034779	0.038456233	0.162473013	uncharacterized protein MGG_12733 [Pyricularia oryzae 70-15]
MGG_12748	380.393476	102.8157101	0.270287785	-1.887431779	1.17058E-05	0.000313219	uncharacterized protein MGG_12748 [Pyricularia oryzae 70-15]
MGG_12775	184.5289941	867.9457506	4.703573847	2.233757355	0.00068737	0.008707168	voltage-gated K+ channel beta subunit [Pyricularia oryzae 70-15]
MGG_12805	2942.038355	6535.963851	2.221576697	1.151583949	0.004266191	0.035159899	tryptophan synthase [Pyricularia oryzae 70-15]
MGG_12818	3001.529011	7167.067924	2.387805647	1.255685415	0.002123839	0.021098803	F-actin-capping protein subunit alpha [Pyricularia oryzae 70-15]
MGG_12844	587.388534	282.8828439	0.481594086	-1.05411042	0.009377433	0.062283795	zeta toxin family protein [Pyricularia oryzae 70-15]
MGG_12848	3.211759421	14.3215879	4.459109796	2.156755723	0.038191101	0.161653483	uncharacterized protein MGG_12848 [Pyricularia oryzae 70-15]
MGG_12858	23778.72147	6820.077801	0.286814319	-1.801811045	1.01724E-05	0.00027895	uncharacterized protein MGG_12858 [Pyricularia oryzae 70-15]
MGG_12861	7310.1918	2976.473882	0.407167686	-1.296305024	0.001235244	0.013958332	uncharacterized protein MGG_12861 [Pyricularia oryzae 70-15]
MGG_12865	479.6838596	1069.725529	2.23006363	1.157084875	0.004237523	0.035042499	uncharacterized protein MGG_12865 [Pyricularia oryzae 70-15]
MGG_12891	48.13340022	8.907921083	0.185067355	-2.433877659	7.55494E-05	0.001477897	uncharacterized protein MGG_12891 [Pyricularia oryzae 70-15]
MGG_12918	639.7970299	170.4430426	0.266401741	-1.908324584	5.94093E-06	0.000176878	phosducin [Pyricularia oryzae 70-15]
MGG_12939	403.7173536	119.4648538	0.29591211	-1.756759358	4.71719E-05	0.000996599	uncharacterized protein MGG_12939 [Pyricularia oryzae 70-15]
MGG_12967	19.14484256	47.8274765	2.498191162	1.320883877	0.018377981	0.098437262	uncharacterized protein MGG_12967 [Pyricularia oryzae 70-15]
MGG_12979	77.11665101	179.8627086	2.332345949	1.221781794	0.007288098	0.05248645	uncharacterized protein MGG_12979 [Pyricularia oryzae 70-15]
MGG_12981	62.70857603	190.0921287	3.031357762	1.59996413	0.007703414	0.054375989	cupin domain-containing protein [Pyricularia oryzae 70-15]
MGG_12982	355.3628472	1773.533481	4.990767872	2.319261804	0.014224324	0.082757225	2-(R)-hydroxypropyl-CoM dehydrogenase
MGG_12983	572.6399064	1874.097716	3.272733343	1.710496059	3.24811E-05	0.000738914	uncharacterized protein MGG_12983 [Pyricularia oryzae 70-15]
MGG_13007	2337.837555	369.9233627	0.158233134	-2.659876364	3.57703E-10	3.03873E-08	uncharacterized protein MGG_13007 [Pyricularia oryzae 70-15]
MGG_13019	26.81325615	101.5207583	3.78621521	1.920756417	0.000142397	0.002492566	uncharacterized protein MGG_13019 [Pyricularia oryzae 70-15]
MGG_13032	526.9001781	1744.882213	3.311599209	1.727528079	0.003875831	0.032779691	formyl-coenzyme A transferase [Pyricularia oryzae 70-15]
MGG_13049	60740.88619	16291.58557	0.268214486	-1.898540937	2.6615E-06	8.76204E-05	uncharacterized protein MGG_13049 [Pyricularia oryzae 70-15]
MGG_13063	22697.46516	8219.6668	0.362140298	-1.465379369	0.000254416	0.004040055	uncharacterized protein MGG_13063 [Pyricularia oryzae 70-15]
MGG_13101	151.3101638	432.5935753	2.858985573	1.51550334	0.000282426	0.004392976	uncharacterized protein MGG_13101 [Pyricularia oryzae 70-15]
MGG_13106	1948.068729	4845.862093	2.487521113	1.314708771	0.004696266	0.038098206	uncharacterized protein MGG_13106 [Pyricularia oryzae 70-15]
MGG_13107	4001.580177	1470.75605	0.367543816	-1.444011845	0.000391146	0.005603347	uncharacterized protein MGG_13107 [Pyricularia oryzae 70-15]
MGG_13108	22.09840375	76.99511164	3.484193362	1.800824691	0.019662202	0.103107564	uncharacterized protein MGG_13108 [Pyricularia oryzae 70-15]

MGG_13110	12.98648339	371.2401711	28.58665891	4.837270109	1.31069E-08	8.00116E-07	uncharacterized protein MGG_13110 [Pyricularia oryzae 70-15]
MGG_13121	1033.079395	2341.97345	2.266983023	1.180773587	0.003693857	0.031578448	uncharacterized protein MGG_13121 [Pyricularia oryzae 70-15]
MGG_13179	47.13223338	139.0024572	2.949201581	1.560324435	0.000860116	0.010446732	uncharacterized protein MGG_13179 [Pyricularia oryzae 70-15]
MGG_13188	5518.877334	12671.43082	2.296016029	1.199132714	0.002926197	0.026828259	voltage-gated potassium channel subunit beta-2
MGG_13219	4721.33964	9659.803974	2.045987942	1.032797643	0.010817761	0.068736853	uncharacterized protein MGG_13219 [Pyricularia oryzae 70-15]
MGG_13221	938.2441768	2002.316687	2.134110434	1.093634833	0.006979814	0.050838542	hsp10-like protein [Pyricularia oryzae 70-15]
MGG_13241	1295.241305	212.1694643	0.163806901	-2.609931956	0.021541223	0.109916067	endoglucanase IV [Pyricularia oryzae 70-15]
MGG_13252	376.3397538	114.4833383	0.304202086	-1.716898047	5.93134E-05	0.001215075	extracellular elastinolytic metalloproteinase
MGG_13254	624.8058838	1721.674143	2.755534459	1.462332169	0.000370928	0.005377134	NAPE-hydrolyzing phospholipase D [Pyricularia oryzae 70-15]
MGG_13261	55.62500257	327.8701119	5.894293874	2.559318991	2.0589E-08	1.18013E-06	uncharacterized protein MGG_13261 [Pyricularia oryzae 70-15]
MGG_13262	431.5449741	1221.453486	2.830419908	1.5010161	0.000318515	0.004811106	FAD binding domain-containing protein [Pyricularia oryzae 70-15]
MGG_13275	264.3326976	88.70224251	0.335570451	-1.575312412	0.000319209	0.004811106	uncharacterized protein MGG_13275 [Pyricularia oryzae 70-15]
MGG_13291	29.9364572	115.6964818	3.864735264	1.950369592	8.68758E-05	0.001667693	copper amine oxidase [Pyricularia oryzae 70-15]
MGG_13324	2274.661046	758.2772045	0.333358329	-1.584854323	0.000130824	0.002327877	uncharacterized protein MGG_13324 [Pyricularia oryzae 70-15]
MGG_13325	0.321175942	7.651763804	23.82421222	4.574356605	0.007915506	0.05532746	uncharacterized protein MGG_13325 [Pyricularia oryzae 70-15]
MGG_13326	1328.506414	393.1483116	0.295932566	-1.756659629	1.99565E-05	0.000484863	uncharacterized protein MGG_13326 [Pyricularia oryzae 70-15]
MGG_13334	635.9435102	1776.971848	2.794229078	1.482450301	0.000366941	0.005351253	general amino acid permease AGP2 [Pyricularia oryzae 70-15]
MGG_13338	104.5383044	288.1547086	2.756450952	1.462811931	0.000567748	0.007473059	MFS transporter [Pyricularia oryzae 70-15]
MGG_13349	8006.111262	3277.867407	0.409420666	-1.288344168	0.003261996	0.02873584	HAL protein kinase [Pyricularia oryzae 70-15]
MGG_13360	64.8878165	249.0782379	3.838597926	1.940579453	1.52656E-05	0.000386348	uncharacterized protein MGG_13360 [Pyricularia oryzae 70-15]
MGG_13365	2.400073171	22.76658683	9.485788644	3.245767724	0.000171561	0.002902671	uncharacterized protein MGG_13365 [Pyricularia oryzae 70-15]
MGG_13374	46.91197966	115.5549733	2.463229524	1.300551064	0.006515651	0.048529812	uncharacterized protein MGG_13374 [Pyricularia oryzae 70-15]
MGG_13420	0	64.86123443	Inf	Inf	2.30867E-16	6.76039E-14	uncharacterized protein MGG_13420 [Pyricularia oryzae 70-15]
MGG_13430	5.703718925	28.69804858	5.031462623	2.330977846	0.003269606	0.028762379	uncharacterized protein MGG_13430 [Pyricularia oryzae 70-15]
MGG_13438	2221.397033	5094.638401	2.293438915	1.197512482	0.003085136	0.027864861	xaa-Pro dipeptidase [Pyricularia oryzae 70-15]
MGG_13441	98.26294278	226.9774883	2.309899153	1.207829867	0.006812455	0.049884092	cytochrome b2 [Pyricularia oryzae 70-15]
MGG_13455	11.77559419	46.01247817	3.907444281	1.966225301	0.001892249	0.019431145	uncharacterized protein MGG_13455 [Pyricularia oryzae 70-15]
MGG_13463	2.014424045	34.53084203	17.14179402	4.099446201	3.00269E-05	0.00068824	uncharacterized protein MGG_13463 [Pyricularia oryzae 70-15]
MGG_13464	300.4762318	974.3914041	3.242823562	1.69725053	3.42364E-05	0.000768768	laccase [Pyricularia oryzae 70-15]
MGG_13483	702.0524994	1407.494478	2.004827957	1.003478438	0.012111969	0.074161392	glutamyl-tRNA(Gln) amidotransferase subunit A
MGG_13512	851.329537	403.5244455	0.47399324	-1.077061611	0.008564447	0.058417124	aspartyl-tRNA synthetase [Pyricularia oryzae 70-15]
MGG_13549	177.1182633	44.34471407	0.250367823	-1.997878932	1.59392E-05	0.000401721	uncharacterized protein MGG_13549 [Pyricularia oryzae 70-15]
MGG_13557	975.9665334	418.9528995	0.429269739	-1.220043619	0.003186786	0.028361226	uncharacterized protein MGG_13557 [Pyricularia oryzae 70-15]
MGG_13578	163.6261883	464.4864525	2.83870484	1.50523285	0.000386347	0.005541135	sulphydryl oxidase [Pyricularia oryzae 70-15]
MGG_13582	14.06146479	1771.523764	125.9842975	6.977100119	2.50509E-14	4.90836E-12	uncharacterized protein MGG_13582 [Pyricularia oryzae 70-15]
MGG_13583	95.81158122	518.7172122	5.413930191	2.436676285	2.52601E-08	1.40761E-06	FAD dependent oxidoreductase superfamily protein
MGG_13584	1268.091235	3039.08295	2.396580677	1.260977506	0.001934684	0.019799947	uncharacterized protein MGG_13584 [Pyricularia oryzae 70-15]
MGG_13585	2.37235452	137.9041908	58.12967228	5.86120287	7.51202E-19	3.14676E-16	uncharacterized protein MGG_13585 [Pyricularia oryzae 70-15]
MGG_13591	26.11094903	76.37181048	2.924896005	1.54838533	0.011832299	0.073112296	mycocerosic acid synthase [Pyricularia oryzae 70-15]
MGG_13597	0	4.49360525	Inf	Inf	0.02802418	0.131598953	FAD/FMN-dependent oxygenase/oxidase
MGG_13598	4.772427691	19.5378445	4.093900581	2.033476067	0.010557262	0.067682314	endothiapepsin [Pyricularia oryzae 70-15]
MGG_13615	56687.11723	24800.86599	0.437504449	-1.192630407	0.003154215	0.028216058	uncharacterized protein MGG_13615 [Pyricularia oryzae 70-15]
MGG_13646	983.6094619	2299.635713	2.337956072	1.225247823	0.002489121	0.023771891	uncharacterized protein MGG_13646 [Pyricularia oryzae 70-15]
MGG_13651	75.44220007	291.7237396	3.866850904	1.951159139	6.9941E-06	0.000204241	high-affinity glucose transporter [Pyricularia oryzae 70-15]
MGG_13654	196.7234414	639.0824437	3.248633916	1.699833177	0.000325506	0.004869764	uncharacterized protein MGG_13654 [Pyricularia oryzae 70-15]
MGG_13669	905.7844903	4555.634277	5.029490266	2.330412192	2.0595E-08	1.18013E-06	peptide transporter PTR2 [Pyricularia oryzae 70-15]
MGG_13713	58.88679687	11.37399487	0.193150171	-2.372205142	0.000130881	0.002327877	uncharacterized protein MGG_13713 [Pyricularia oryzae 70-15]
MGG_13715	481.6375389	215.1146384	0.446631795	-1.162842138	0.005190959	0.040995467	beta-lactamase [Pyricularia oryzae 70-15]

MGG_13716	268.5689836	1052.314364	3.918227452	1.970201148	0.000448831	0.006245594	uncharacterized protein MGG_13716 [Pyricularia oryzae 70-15]
MGG_13742	0	3.829605001	Inf	Inf	0.048702812	0.18950729	uncharacterized protein MGG_13742 [Pyricularia oryzae 70-15]
MGG_13764	167.688864	17.42728211	0.103926294	-3.266367389	6.42066E-07	2.51607E-05	bilirubin oxidase [Pyricularia oryzae 70-15]
MGG_13765	1616.448473	7056.431558	4.365392202	2.126111276	2.43297E-07	1.02982E-05	uncharacterized protein MGG_13765 [Pyricularia oryzae 70-15]
MGG_13767	62.50108721	499.7488331	7.995842239	2.999250007	4.98889E-11	5.17991E-09	lovastatin nonaketide synthase [Pyricularia oryzae 70-15]
MGG_13768	25.35174114	288.9316552	11.39691564	3.510571534	7.76341E-13	1.12274E-10	uncharacterized protein MGG_13768 [Pyricularia oryzae 70-15]
MGG_13781	3583.178592	7291.395528	2.034895929	1.024955012	0.009993304	0.065488968	serine hydroxymethyltransferase [Pyricularia oryzae 70-15]
MGG_13793	253.3863459	1717.663341	6.77883149	2.761036608	1.34821E-10	1.26962E-08	nitrate transporter [Pyricularia oryzae 70-15]
MGG_13805	1633.84092	477.2935116	0.292129733	-1.775318892	1.89754E-05	0.000465683	uncharacterized protein MGG_13805 [Pyricularia oryzae 70-15]
MGG_13834	1352.941938	3118.146863	2.304715949	1.204588953	0.021249325	0.108826645	YjeF [Pyricularia oryzae 70-15]
MGG_13871	159.8288507	20.42359797	0.127784176	-2.968218902	4.57488E-09	3.02042E-07	uncharacterized protein MGG_13871 [Pyricularia oryzae 70-15]
MGG_13875	381.9681179	1690.411831	4.425531221	2.14585064	0.000291668	0.004502139	uncharacterized protein MGG_13875 [Pyricularia oryzae 70-15]
MGG_13894	30.73968655	96.98791763	3.155136845	1.657702579	0.000924656	0.011044961	uncharacterized protein MGG_13894 [Pyricularia oryzae 70-15]
MGG_13912	16.32355571	62.53510557	3.830973268	1.937710959	0.000407798	0.005787307	uncharacterized protein MGG_13912 [Pyricularia oryzae 70-15]
MGG_13926	159.6299778	45.38034075	0.284284577	-1.814592262	0.00239001	0.023061033	uncharacterized protein MGG_13926 [Pyricularia oryzae 70-15]
MGG_13931	1376.674513	687.0752258	0.499083276	-1.002647534	0.012979365	0.078017479	CMGC/CDK/CDK8 protein kinase [Pyricularia oryzae 70-15]
MGG_13971	19.50396302	109.2839417	5.603165959	2.486242224	1.13338E-06	4.19765E-05	uncharacterized protein MGG_13971 [Pyricularia oryzae 70-15]
MGG_13977	434.9487211	966.2112756	2.221437215	1.151493367	0.005292769	0.041503884	alkaline proteinase [Pyricularia oryzae 70-15]
MGG_14010	1533.603719	673.9723372	0.439469681	-1.186164457	0.003498638	0.030207142	uncharacterized protein MGG_14010 [Pyricularia oryzae 70-15]
MGG_14042	141.329141	12.41462249	0.087841916	-3.508946672	6.00735E-11	6.08144E-09	uncharacterized protein MGG_14042 [Pyricularia oryzae 70-15]
MGG_14052	365.2404026	880.603741	2.411024998	1.269646609	0.00248342	0.023753136	uncharacterized protein MGG_14052 [Pyricularia oryzae 70-15]
MGG_14055	417.7716853	1318.697802	3.156503537	1.658327368	3.3556E-05	0.000756286	uncharacterized protein MGG_14055 [Pyricularia oryzae 70-15]
MGG_14057	205.989112	414.3248865	2.011392168	1.008194396	0.018333687	0.098286685	lipase 5 [Pyricularia oryzae 70-15]
MGG_14061	64.11444379	26.27777472	0.40985733	-1.286806296	0.012106941	0.074161392	oxalate decarboxylase oxdD [Pyricularia oryzae 70-15]
MGG_14068	438.799225	1766.205236	4.025087409	2.009020113	7.95308E-07	3.0574E-05	FAD dependent oxidoreductase [Pyricularia oryzae 70-15]
MGG_14069	219.082194	30.41665036	0.138836707	-2.848539044	1.84629E-09	1.32715E-07	heavy metal tolerance protein [Pyricularia oryzae 70-15]
MGG_14109	6.979081309	31.34286188	4.490972449	2.167027871	0.002152463	0.021293256	uncharacterized protein MGG_14109 [Pyricularia oryzae 70-15]
MGG_14110	14.97437876	69.46670009	4.639037198	2.213825415	5.0903E-05	0.001066154	uncharacterized protein MGG_14110 [Pyricularia oryzae 70-15]
MGG_14113	153.3043908	735.9685816	4.800701258	2.263245162	0.010035459	0.065578674	uncharacterized protein MGG_14113 [Pyricularia oryzae 70-15]
MGG_14114	3.996611497	18.21555311	4.557749264	2.18832156	0.011089637	0.069913293	citrinin biosynthesis oxydoreductase CtnB
MGG_14115	1058.049477	470.2721744	0.444470873	-1.169839216	0.003861572	0.032740386	GabA permease [Pyricularia oryzae 70-15]
MGG_14118	26776.32934	8141.842101	0.304068642	-1.717531053	2.94562E-05	0.000676433	uncharacterized protein MGG_14118 [Pyricularia oryzae 70-15]
MGG_14126	15.44287834	2.321127911	0.150304099	-2.734043743	0.007680973	0.054312257	uncharacterized protein MGG_14126 [Pyricularia oryzae 70-15]
MGG_14136	2738.406788	1357.623339	0.495771244	-1.012253502	0.034914388	0.152782139	quinate permease [Pyricularia oryzae 70-15]
MGG_14146	187.8734798	14.57713476	0.077590167	-3.68798235	2.02516E-11	2.21636E-09	uncharacterized protein MGG_14146 [Pyricularia oryzae 70-15]
MGG_14151	52.70596239	472.9214742	8.972826845	3.165562571	1.85699E-12	2.50682E-10	uncharacterized protein MGG_14151 [Pyricularia oryzae 70-15]
MGG_14179	13.27994068	2.713429467	0.204325421	-2.291059389	0.020355011	0.105626942	uncharacterized protein MGG_14179 [Pyricularia oryzae 70-15]
MGG_14195	0.660729151	12.89362285	19.51423339	4.286454883	0.000856947	0.010431059	uncharacterized protein MGG_14195 [Pyricularia oryzae 70-15]
MGG_14198	1.656493569	16.83386565	10.16234893	3.345162	0.000874281	0.010557418	uncharacterized protein MGG_14198 [Pyricularia oryzae 70-15]
MGG_14219	2410.752815	5262.904733	2.183095961	1.126375547	0.005272233	0.041454428	ubiquitin thioesterase [Pyricularia oryzae 70-15]
MGG_14251	50.89836529	18.87959017	0.37092724	-1.430791874	0.010123502	0.065905842	uncharacterized protein MGG_14251 [Pyricularia oryzae 70-15]
MGG_14264	529.8634812	2491.132723	4.701461436	2.233109284	1.1249E-07	5.28838E-06	cytochrome b2 [Pyricularia oryzae 70-15]
MGG_14269	184.9236156	982.9813631	5.315607528	2.410234589	1.32963E-08	8.07617E-07	uncharacterized protein MGG_14269 [Pyricularia oryzae 70-15]
MGG_14292	1295.221932	3150.515425	2.432413586	1.282388553	0.001630797	0.017256901	leupeptin-inactivating enzyme 1 [Pyricularia oryzae 70-15]
MGG_14324	208.2135959	478.4153655	2.297714342	1.20019945	0.004767232	0.038479953	uncharacterized protein MGG_14324 [Pyricularia oryzae 70-15]
MGG_14330	62.78550862	307.8926189	4.903880302	2.293923765	4.48711E-07	1.81094E-05	uncharacterized protein MGG_14330 [Pyricularia oryzae 70-15]
MGG_14338	172.7829195	50.42238114	0.291825033	-1.776824452	0.000129326	0.002310373	uncharacterized protein MGG_14338 [Pyricularia oryzae 70-15]
MGG_14340	6.346955287	22.26568871	3.508089738	1.810685653	0.01268805	0.076760174	uncharacterized protein MGG_14340 [Pyricularia oryzae 70-15]

MGG_14348	2628.819729	755.5368763	0.287405358	-1.798841135	1.27924E-05	0.000334919	uncharacterized protein MGG_14348 [Pyricularia oryzae 70-15]
MGG_14350	722.2157093	1724.488494	2.387774832	1.255666796	0.001827521	0.018878165	ribosome assembly protein RRB1 [Pyricularia oryzae 70-15]
MGG_14351	61.35494466	240.7810139	3.924394606	1.972470115	0.00127969	0.014354264	glycosyltransferase [Pyricularia oryzae 70-15]
MGG_14367	1016.105037	378.4420201	0.372443799	-1.424905353	0.000637191	0.008217191	MFS transporter [Pyricularia oryzae 70-15]
MGG_14422	7.823888629	115.3806835	14.74722979	3.88237207	3.84089E-08	2.04645E-06	uncharacterized protein MGG_14422 [Pyricularia oryzae 70-15]
MGG_14523	79.97609233	309.2176229	3.866375737	1.950981846	9.39951E-06	0.0002631	uncharacterized protein MGG_14523 [Pyricularia oryzae 70-15]
MGG_14540	260.8634465	87.96466195	0.33720578	-1.568298829	0.000292215	0.004504851	uncharacterized protein MGG_14540 [Pyricularia oryzae 70-15]
MGG_14547	3014.470696	1080.335788	0.358383244	-1.480424907	0.000252678	0.004017711	uncharacterized protein MGG_14547 [Pyricularia oryzae 70-15]
MGG_14561	1816.525493	4291.985887	2.362744648	1.240463719	0.002136801	0.02120058	regulatory protein Cys-3 [Pyricularia oryzae 70-15]
MGG_14564	106.9723322	5.973997824	0.055846196	-4.162397178	6.64896E-11	6.67534E-09	uncharacterized protein MGG_14564 [Pyricularia oryzae 70-15]
MGG_14571	130.3457929	62.4888648	0.479408375	-1.060672981	0.030503524	0.139812927	uncharacterized protein MGG_14571 [Pyricularia oryzae 70-15]
MGG_14578	29.92467233	12.3328142	0.412128629	-1.278833408	0.041634898	0.171742186	uncharacterized protein MGG_14578 [Pyricularia oryzae 70-15]
MGG_14587	1211.65526	593.4491328	0.489783813	-1.029783001	0.012016781	0.073839078	Ser/Thr protein phosphatase [Pyricularia oryzae 70-15]
MGG_14591	10.41619137	28.49322898	2.735474796	1.451791263	0.030699012	0.140405774	cytochrome P450 3A24 [Pyricularia oryzae 70-15]
MGG_14597	117.9210953	39.79742185	0.337491962	-1.567074951	0.001128152	0.012977531	uncharacterized protein MGG_14597 [Pyricularia oryzae 70-15]
MGG_14598	64.7070663	19.27886028	0.297940571	-1.746903504	0.001185101	0.013492604	uncharacterized protein MGG_14598 [Pyricularia oryzae 70-15]
MGG_14601	79.39919366	8.90295695	0.11212906	-3.156767871	3.54382E-08	1.89649E-06	uncharacterized protein MGG_14601 [Pyricularia oryzae 70-15]
MGG_14602	390.7700688	182.4098351	0.466795821	-1.099136449	0.008717686	0.059130349	endo-1,6-beta-D-glucanase [Pyricularia oryzae 70-15]
MGG_14603	91.31651795	7.968480637	0.087262204	-3.518499274	5.47388E-05	0.001132822	uncharacterized protein MGG_14603 [Pyricularia oryzae 70-15]
MGG_14606	30.85144563	12.59958557	0.408395306	-1.291961812	0.04332398	0.175794198	uncharacterized protein MGG_14606 [Pyricularia oryzae 70-15]
MGG_14611	60.81219797	15.47285331	0.254436673	-1.97462147	0.015799821	0.088572326	uncharacterized protein MGG_14611 [Pyricularia oryzae 70-15]
MGG_14612	9625.631891	20257.54038	2.104541355	1.073505859	0.007780152	0.054663553	uncharacterized protein MGG_14612 [Pyricularia oryzae 70-15]
MGG_14632	94.36633752	17.47970964	0.185232468	-2.432591091	0.000783667	0.009674779	uncharacterized protein MGG_14632 [Pyricularia oryzae 70-15]
MGG_14636	3.217161839	36.1405063	11.23366125	3.4897563	0.000344261	0.005081517	uncharacterized protein MGG_14636 [Pyricularia oryzae 70-15]
MGG_14637	1551.525016	400.2039666	0.257942323	-1.954879587	0.003661556	0.031346426	uncharacterized protein MGG_14637 [Pyricularia oryzae 70-15]
MGG_14655	86.74093333	196.5557476	2.266009138	1.180153679	0.006653731	0.04910664	uncharacterized protein MGG_14655 [Pyricularia oryzae 70-15]
MGG_14661	154.0950016	57.63233876	0.374005245	-1.418869594	0.009179299	0.061438083	uncharacterized protein MGG_14661 [Pyricularia oryzae 70-15]
MGG_14663	1325.195997	371.5919454	0.280405273	-1.834414615	8.55258E-06	0.000242184	uncharacterized protein MGG_14663 [Pyricularia oryzae 70-15]
MGG_14668	23.88051572	7.348239091	0.307708559	-1.70036352	0.016654298	0.091962003	uncharacterized protein MGG_14668 [Pyricularia oryzae 70-15]
MGG_14685	516.5723031	231.3717846	0.447898161	-1.158757352	0.004855814	0.038914416	uncharacterized protein MGG_14685 [Pyricularia oryzae 70-15]
MGG_14692	1226.596346	349.2770255	0.284753029	-1.812216904	1.09665E-05	0.000297367	uncharacterized protein MGG_14692 [Pyricularia oryzae 70-15]
MGG_14701	22.01341531	0.888235544	0.040349738	-4.631296878	6.88312E-06	0.000201485	uncharacterized protein MGG_14701 [Pyricularia oryzae 70-15]
MGG_14702	1048.172106	2476.476856	2.36266243	1.240413516	0.003024545	0.027419529	uncharacterized protein MGG_14702 [Pyricularia oryzae 70-15]
MGG_14703	86.19929372	34.55226214	0.400841592	-1.318895881	0.006522413	0.048550414	uncharacterized protein MGG_14703 [Pyricularia oryzae 70-15]
MGG_14704	71.58943783	24.69252709	0.344918578	-1.535672258	0.003299866	0.028964435	uncharacterized protein MGG_14704 [Pyricularia oryzae 70-15]
MGG_14706	42.68638405	210.5742043	4.933053219	2.302480852	7.38359E-07	2.86568E-05	uncharacterized protein MGG_14706 [Pyricularia oryzae 70-15]
MGG_14711	661.636714	226.6571035	0.342570324	-1.545527915	0.00032325	0.004847951	uncharacterized protein MGG_14711 [Pyricularia oryzae 70-15]
MGG_14712	1170.987373	244.514878	0.208810858	-2.259731363	8.26771E-08	3.96981E-06	uncharacterized protein MGG_14712 [Pyricularia oryzae 70-15]
MGG_14737	70.52104882	34.11620188	0.483773319	-1.047596889	0.033911048	0.149909537	uncharacterized protein MGG_14737 [Pyricularia oryzae 70-15]
MGG_14743	1252.550426	160.739598	0.128329842	-2.962071402	7.89833E-12	9.05178E-10	uncharacterized protein MGG_14743 [Pyricularia oryzae 70-15]
MGG_14745	25.67950948	3.628490091	0.141299042	-2.82317641	0.000247219	0.003936071	uncharacterized protein MGG_14745 [Pyricularia oryzae 70-15]
MGG_14758	642.1133875	178.4541592	0.277916895	-1.847274551	1.22332E-05	0.000324473	uncharacterized protein MGG_14758 [Pyricularia oryzae 70-15]
MGG_14764	948.6172565	473.6826633	0.499340129	-1.001905242	0.012776203	0.077101499	uncharacterized protein MGG_14764 [Pyricularia oryzae 70-15]
MGG_14767	82.84259062	330.6606957	3.991433552	1.996906994	6.12273E-06	0.00018097	non-ribosomal peptide synthetase [Pyricularia oryzae 70-15]
MGG_14773	5133.563459	2186.163631	0.425856941	-1.231559231	0.002266215	0.022219518	AGC/AKT protein kinase [Pyricularia oryzae 70-15]
MGG_14778	4232.923474	1043.799167	0.246590606	-2.019810251	0.002027738	0.020460307	leptomycin B resistance protein pmd1 [Pyricularia oryzae 70-15]
MGG_14779	151.0648895	66.51836209	0.44032973	-1.183343839	0.007847724	0.054947639	uncharacterized protein MGG_14779 [Pyricularia oryzae 70-15]
MGG_14785	707.7921966	1686.773549	2.383147988	1.252868542	0.002071082	0.02075867	uncharacterized protein MGG_14785 [Pyricularia oryzae 70-15]

MGG_14793	30.89723604	137.6839073	4.45618848	2.155810255	8.6716E-06	0.000244983	uncharacterized protein MGG_14793 [Pyricularia oryzae 70-15]
MGG_14794	166.0170985	77.44636843	0.466496338	-1.10006234	0.014895447	0.085313477	uncharacterized protein MGG_14794 [Pyricularia oryzae 70-15]
MGG_14806	3610.745992	1529.135655	0.42349577	-1.239580534	0.023891487	0.117741899	uncharacterized protein MGG_14806 [Pyricularia oryzae 70-15]
MGG_14810	48.82880945	14.61592933	0.299330037	-1.740191039	0.004934185	0.039411739	uncharacterized protein MGG_14810 [Pyricularia oryzae 70-15]
MGG_14819	0.642351884	38.73285525	60.29850025	5.914050215	8.60611E-10	6.70173E-08	class II Aldolase [Pyricularia oryzae 70-15]
MGG_14824	67.7443624	179.5326629	2.650149127	1.406073544	0.001821486	0.018847886	P-glycoprotein 5 [Pyricularia oryzae 70-15]
MGG_14831	0.371789801	12.68009761	34.10555529	5.091934847	0.000271803	0.004265965	fatty acid synthase S-acetyltransferase [Pyricularia oryzae 70-15]
MGG_14837	42.83282321	94.60592388	2.208724917	1.143213751	0.018610543	0.099145935	uncharacterized protein MGG_14837 [Pyricularia oryzae 70-15]
MGG_14847	3716.794407	1781.5277	0.479318333	-1.060943973	0.009491648	0.062870521	STE/STE11 protein kinase [Pyricularia oryzae 70-15]
MGG_14851	98.78470964	38.56638938	0.390408491	-1.356943666	0.004962898	0.039520536	uncharacterized protein MGG_14851 [Pyricularia oryzae 70-15]
MGG_14856	542.6215788	160.2694506	0.295361366	-1.759446966	3.7798E-05	0.00083334	uncharacterized protein MGG_14856 [Pyricularia oryzae 70-15]
MGG_14857	208.3564525	500.8963016	2.404035467	1.265458181	0.046221092	0.183494714	uncharacterized protein MGG_14857 [Pyricularia oryzae 70-15]
MGG_14861	239.5227745	87.26020366	0.364308588	-1.456767092	0.045238176	0.181370745	riboflavin aldehyde-forming enzyme [Pyricularia oryzae 70-15]
MGG_14862	109.0010355	52.26688032	0.479508108	-1.060372884	0.023882962	0.117741899	uncharacterized protein MGG_14862 [Pyricularia oryzae 70-15]
MGG_14868	1094.308595	451.4844207	0.412575048	-1.277271524	0.001609996	0.017096355	myo-inositol transporter 1 [Pyricularia oryzae 70-15]
MGG_14870	2572.940817	943.0741528	0.366535501	-1.447975155	0.000369905	0.005376242	uncharacterized protein MGG_14870 [Pyricularia oryzae 70-15]
MGG_14872	3907.407775	436.9143079	0.111816921	-3.160789566	2.90903E-13	4.64986E-11	calpain-9 [Pyricularia oryzae 70-15]
MGG_14873	100.3297496	229.215559	2.284622057	1.191955521	0.006556299	0.048663747	uncharacterized protein MGG_14873 [Pyricularia oryzae 70-15]
MGG_14878	155.1475204	339.1731084	2.186132963	1.12838115	0.008230984	0.056715821	thymocyte nuclear protein 1 [Pyricularia oryzae 70-15]
MGG_14882	3392.771364	1607.094999	0.473682081	-1.078008999	0.024287197	0.119064112	epoxide hydrolase 1 [Pyricularia oryzae 70-15]
MGG_14883	375.0785624	123.7488751	0.32992788	-1.599777398	0.004785045	0.038559532	methyltransferase small domain-containing protein
MGG_14896	0	3.878327837	Inf	Inf	0.047789103	0.187271622	uncharacterized protein MGG_14896 [Pyricularia oryzae 70-15]
MGG_14908	448.5019413	198.7559317	0.44315512	-1.174116313	0.004506704	0.03686696	uncharacterized protein MGG_14908 [Pyricularia oryzae 70-15]
MGG_14931	1519.402802	602.6128357	0.396611639	-1.334201079	0.001037138	0.012114572	zinc finger protein 32 [Pyricularia oryzae 70-15]
MGG_14943	17.61189295	88.42683912	5.020859449	2.32793434	1.13319E-05	0.000305658	lovastatin nonaketide synthase [Pyricularia oryzae 70-15]
MGG_14944	2.744144321	17.59408894	6.411502778	2.680662547	0.002851461	0.02636262	uncharacterized protein MGG_14944 [Pyricularia oryzae 70-15]
MGG_14949	284.8846808	136.4275871	0.47888706	-1.062242643	0.04431247	0.178839829	uncharacterized protein MGG_14949 [Pyricularia oryzae 70-15]
MGG_14954	3902.718487	767.1726056	0.196573903	-2.346856291	0.03659449	0.157099491	endoglucanase 1 [Pyricularia oryzae 70-15]
MGG_14957	0	3.878327837	Inf	Inf	0.047789103	0.187271622	uncharacterized protein MGG_14957 [Pyricularia oryzae 70-15]
MGG_14966	4539.377742	831.3045926	0.183131839	-2.44904546	3.33701E-05	0.000754898	uncharacterized protein MGG_14966 [Pyricularia oryzae 70-15]
MGG_14988	29.76586933	62.61391734	2.10354741	1.072824334	0.035153679	0.153503555	uncharacterized protein MGG_14988 [Pyricularia oryzae 70-15]
MGG_14990	162.0727869	46.80779134	0.288807222	-1.791821276	0.001663775	0.017533054	uncharacterized protein MGG_14990 [Pyricularia oryzae 70-15]
MGG_15000	466.233573	163.3121273	0.350279638	-1.513420968	0.000349505	0.005140179	uncharacterized protein MGG_15000 [Pyricularia oryzae 70-15]
MGG_15015	6.158359177	19.67034323	3.194088339	1.675404214	0.030793221	0.140479191	uncharacterized protein MGG_15015 [Pyricularia oryzae 70-15]
MGG_15021	453.5601583	220.8602861	0.486948163	-1.038159892	0.012236714	0.074622274	uncharacterized protein MGG_15021 [Pyricularia oryzae 70-15]
MGG_15025	999.1077708	3180.830149	3.183670713	1.670691126	4.58384E-05	0.000975209	uncharacterized protein MGG_15025 [Pyricularia oryzae 70-15]
MGG_15027	1755.668561	4604.376651	2.622577378	1.390985339	0.000532794	0.007136029	sulfate adenylyltransferase [Pyricularia oryzae 70-15]
MGG_15041	2.000564719	10.63937403	5.318185375	2.410934066	0.030210458	0.138908645	medium-chain specific acyl-CoA dehydrogenase
MGG_15057	54.30503975	17.67337835	0.325446375	-1.619508248	0.003216742	0.028481764	uncharacterized protein MGG_15057 [Pyricularia oryzae 70-15]
MGG_15063	2024.201415	413.8956625	0.204473557	-2.290013815	4.94146E-08	2.56534E-06	uncharacterized protein MGG_15063 [Pyricularia oryzae 70-15]
MGG_15065	536.0816686	239.8054652	0.447330098	-1.160588264	0.004851969	0.038914416	uncharacterized protein MGG_15065 [Pyricularia oryzae 70-15]
MGG_15066	89.39074784	35.50289029	0.397165156	-1.332189038	0.00779213	0.05471607	uncharacterized protein MGG_15066 [Pyricularia oryzae 70-15]
MGG_15077	535.2294673	136.6113906	0.255238919	-1.970079766	3.98073E-06	0.000123995	uncharacterized protein MGG_15077 [Pyricularia oryzae 70-15]
MGG_15085	771.8202321	307.1930451	0.398011133	-1.329119309	0.004500148	0.036838135	uncharacterized protein MGG_15085 [Pyricularia oryzae 70-15]
MGG_15095	1.945432919	20.61474781	10.59648349	3.405513672	0.000259325	0.004101924	uncharacterized protein MGG_15095 [Pyricularia oryzae 70-15]
MGG_15097	0	17.17618493	Inf	Inf	2.30936E-06	7.68605E-05	polyketide synthase [Pyricularia oryzae 70-15]
MGG_15100	140.4413237	394.4727854	2.808808511	1.489958273	0.008688571	0.058998749	polyketide synthase [Pyricularia oryzae 70-15]
MGG_15117	672.0302008	206.6846257	0.307552585	-1.701094991	5.28154E-05	0.001096754	uncharacterized protein MGG_15117 [Pyricularia oryzae 70-15]

MGG_15129	222.3339403	107.1832707	0.48208236	-1.052648453	0.013217911	0.078904758	uncharacterized protein MGG_15129 [Pyricularia oryzae 70-15]
MGG_15135	6.571421452	107.71981	16.39216276	4.034934309	4.33063E-12	5.31399E-10	uncharacterized protein MGG_15135 [Pyricularia oryzae 70-15]
MGG_15143	223.8690473	88.49494083	0.395297795	-1.338988186	0.007076471	0.051322368	uncharacterized protein MGG_15143 [Pyricularia oryzae 70-15]
MGG_15145	515.0927667	110.2568716	0.214052456	-2.223963708	2.31611E-07	9.87233E-06	uncharacterized protein MGG_15145 [Pyricularia oryzae 70-15]
MGG_15150	686.8208914	3343.748471	4.86844316	2.283460498	2.64013E-08	1.45123E-06	uncharacterized protein MGG_15150 [Pyricularia oryzae 70-15]
MGG_15152	0	10.54689249	Inf	Inf	0.000225952	0.003645231	uncharacterized protein MGG_15152 [Pyricularia oryzae 70-15]
MGG_15167	215.1075932	60.30527338	0.280349347	-1.834702383	4.59694E-05	0.000976287	succinyl-CoA ligase subunit alpha [Pyricularia oryzae 70-15]
MGG_15175	5471.083688	1969.854964	0.360048407	-1.473737212	0.014445554	0.083683637	uncharacterized protein MGG_15175 [Pyricularia oryzae 70-15]
MGG_15176	445.9459353	897.8286165	2.013312703	1.009571266	0.010329112	0.066740481	cyclin-H [Pyricularia oryzae 70-15]
MGG_15182	0	12.93986362	Inf	Inf	4.16723E-05	0.000901495	MFS transporter [Pyricularia oryzae 70-15]
MGG_15205	427.8738532	200.8980362	0.469526321	-1.090722059	0.012155891	0.074355367	uncharacterized protein MGG_15205 [Pyricularia oryzae 70-15]
MGG_15207	85.93718854	199.2795356	2.318897546	1.21343908	0.00740321	0.053027239	uncharacterized protein MGG_15207 [Pyricularia oryzae 70-15]
MGG_15210	144.4666974	475.9603646	3.294602652	1.720104479	0.003929906	0.033153124	uncharacterized protein MGG_15210 [Pyricularia oryzae 70-15]
MGG_15211	0.71134301	87.2829831	122.7016811	6.939011204	3.03095E-17	9.44103E-15	uncharacterized protein MGG_15211 [Pyricularia oryzae 70-15]
MGG_15246	382.5529072	147.1591648	0.384676634	-1.378281894	0.001001978	0.011760417	uncharacterized protein MGG_15246 [Pyricularia oryzae 70-15]
MGG_15247	270.8406894	104.0635395	0.384224172	-1.379979813	0.001372677	0.015104425	dipeptidase 3 [Pyricularia oryzae 70-15]
MGG_15248	276.4253188	110.1542988	0.398495692	-1.327363965	0.002325059	0.022606544	uncharacterized protein MGG_15248 [Pyricularia oryzae 70-15]
MGG_15253	935.9139604	0	0	-Inf	9.6304E-50	5.8495E-46	uncharacterized protein MGG_15253 [Pyricularia oryzae 70-15]
MGG_15258	178.1199456	383.1486211	2.151070841	1.105055038	0.008432044	0.057791077	uncharacterized protein MGG_15258 [Pyricularia oryzae 70-15]
MGG_15265	1.757721287	13.16161685	7.487886134	2.904558496	0.005276783	0.041463366	uncharacterized protein MGG_15265 [Pyricularia oryzae 70-15]
MGG_15266	359.8027975	134.0188313	0.372478569	-1.424770672	0.000902471	0.010843929	uncharacterized protein MGG_15266 [Pyricularia oryzae 70-15]
MGG_15280	194.7385811	510.6458643	2.622212103	1.390784386	0.001065445	0.012385675	uncharacterized protein MGG_15280 [Pyricularia oryzae 70-15]
MGG_15286	148.0532056	69.44031585	0.469022711	-1.092270312	0.035197298	0.153511641	uncharacterized protein MGG_15286 [Pyricularia oryzae 70-15]
MGG_15288	1178.329297	391.9886184	0.332664748	-1.587859101	0.000128068	0.002291271	N-acetylated-alpha-linked acidic dipeptidase 2
MGG_15326	160.7271799	72.0744191	0.448427075	-1.157054709	0.008574805	0.058454957	uncharacterized protein MGG_15326 [Pyricularia oryzae 70-15]
MGG_15327	73.06865557	24.43938868	0.33447158	-1.580044464	0.002773514	0.025818124	uncharacterized protein MGG_15327 [Pyricularia oryzae 70-15]
MGG_15329	87.42068225	32.56971201	0.372563004	-1.424443674	0.006682849	0.04920197	uncharacterized protein MGG_15329 [Pyricularia oryzae 70-15]
MGG_15331	162.8999109	392.5788236	2.409938848	1.268996539	0.002326159	0.022606544	uncharacterized protein MGG_15331 [Pyricularia oryzae 70-15]
MGG_15337	20.91248421	186.0284338	8.895568403	3.153086793	1.02309E-07	4.87391E-06	zygote-specific protein [Pyricularia oryzae 70-15]
MGG_15339	468.3047005	175.5164682	0.374791173	-1.415841118	0.000801986	0.009860851	hypothetical protein, variant [Pyricularia oryzae 70-15]
MGG_15343	16.79990119	82.02004489	4.882174243	2.287523785	1.84677E-05	0.000457849	uncharacterized protein MGG_15343 [Pyricularia oryzae 70-15]
MGG_15348	20.70493186	6.885353752	0.332546555	-1.588371771	0.033806904	0.149738831	uncharacterized protein MGG_15348 [Pyricularia oryzae 70-15]
MGG_15349	712.5774207	351.3353848	0.49304872	-1.020197885	0.011366543	0.071102353	uncharacterized protein MGG_15349 [Pyricularia oryzae 70-15]
MGG_15350	41.11548992	120.655685	2.934555449	1.553141968	0.001381115	0.015169734	uncharacterized protein MGG_15350 [Pyricularia oryzae 70-15]
MGG_15351	61.80476147	125.6927981	2.033707356	1.024112095	0.029839778	0.137673231	uncharacterized protein MGG_15351 [Pyricularia oryzae 70-15]
MGG_15353	56.22971544	732.3089729	13.02352265	3.703047822	1.26798E-15	3.02027E-13	uncharacterized protein MGG_15353 [Pyricularia oryzae 70-15]
MGG_15354	68.18761885	536.1784817	7.863282085	2.97513161	5.24236E-11	5.39696E-09	uncharacterized protein MGG_15354 [Pyricularia oryzae 70-15]
MGG_15357	77.51969738	300.065788	3.870832809	1.952643995	0.000240582	0.003855662	MYB DNA-binding domain-containing protein
MGG_15360	1.385931486	18.56283653	13.39376204	3.743489337	0.000180575	0.003025697	uncharacterized protein MGG_15360 [Pyricularia oryzae 70-15]
MGG_15362	0.71134301	180.7727945	254.128869	7.989416465	4.31997E-13	6.72807E-11	uncharacterized protein MGG_15362 [Pyricularia oryzae 70-15]
MGG_15378	93.30457241	10.34825965	0.110908387	-3.172559624	9.45344E-09	5.83374E-07	uncharacterized protein MGG_15378 [Pyricularia oryzae 70-15]
MGG_15380	33.40040144	94.10768133	2.817561385	1.494447042	0.003001721	0.027273682	salicylate hydroxylase [Pyricularia oryzae 70-15]
MGG_15394	2.166265621	12.11354303	5.591901065	2.483338836	0.015573298	0.087526448	hypothetical protein, variant [Pyricularia oryzae 70-15]
MGG_15413	58.64385789	132.1587578	2.253582261	1.172220113	0.009231088	0.061581143	uncharacterized protein MGG_15413 [Pyricularia oryzae 70-15]
MGG_15414	10.19172521	2.123754504	0.208380275	-2.262709373	0.041140715	0.170223911	uncharacterized protein MGG_15414 [Pyricularia oryzae 70-15]
MGG_15415	76.95577356	274.9195912	3.572436199	1.836908246	0.00310594	0.027948863	uncharacterized protein MGG_15415 [Pyricularia oryzae 70-15]
MGG_15416	435.5005677	1473.734619	3.384001602	1.758730252	2.44822E-05	0.000571941	uncharacterized protein MGG_15416 [Pyricularia oryzae 70-15]
MGG_15418	240.1827589	498.7692771	2.076623982	1.054240009	0.013003576	0.07812435	uncharacterized protein MGG_15418 [Pyricularia oryzae 70-15]

MGG_15430	127.6629849	12.68261649	0.099344509	-3.331415968	0.028472019	0.132915008	endo-1,4-beta-xylanase A [Pyricularia oryzae 70-15]
MGG_15443	0	18.06241606	Inf	Inf	0.000300031	0.004591438	uncharacterized protein MGG_15443 [Pyricularia oryzae 70-15]
MGG_15444	11.37608574	87.33691112	7.677237422	2.940587265	0.01321491	0.078904758	uncharacterized protein MGG_15444 [Pyricularia oryzae 70-15]
MGG_15457	7.19539607	115.9306454	16.11178096	4.01004407	3.04585E-12	3.81453E-10	arylsulfatase [Pyricularia oryzae 70-15]
MGG_15458	0	22.95403197	Inf	Inf	5.99408E-08	3.00893E-06	uncharacterized protein MGG_15458 [Pyricularia oryzae 70-15]
MGG_15459	0	18.87781627	Inf	Inf	0.03402962	0.150161218	uncharacterized protein MGG_15459 [Pyricularia oryzae 70-15]
MGG_15460	0	11.61434519	Inf	Inf	0.006452358	0.048291537	cutinase [Pyricularia oryzae 70-15]
MGG_15461	3.941479697	22.09017625	5.604538891	2.486595681	0.002397924	0.023100702	uncharacterized protein MGG_15461 [Pyricularia oryzae 70-15]
MGG_15485	1.321458302	32.05109848	24.25433965	4.600170996	2.25676E-06	7.53164E-05	TKL protein kinase [Pyricularia oryzae 70-15]
MGG_15499	37.33757315	7.345757025	0.196739006	-2.345645077	0.001333423	0.014754387	uncharacterized protein MGG_15499 [Pyricularia oryzae 70-15]
MGG_15501	639.2667741	1552.217833	2.42812218	1.279841018	0.011172999	0.070289795	hypothetical protein, variant [Pyricularia oryzae 70-15]
MGG_15504	6.05713146	20.19436174	3.333981089	1.737245921	0.027734152	0.130778955	uncharacterized protein MGG_15504 [Pyricularia oryzae 70-15]
MGG_15517	65.43678657	500.0990515	7.642475704	2.934040061	8.72284E-09	5.46212E-07	uncharacterized protein MGG_15517 [Pyricularia oryzae 70-15]
MGG_15520	71.74988274	171.4854336	2.390044792	1.257037656	0.003785501	0.032225832	uncharacterized protein MGG_15520 [Pyricularia oryzae 70-15]
MGG_15523	638.7952715	300.793236	0.470875802	-1.086581508	0.008742528	0.059265753	uncharacterized protein MGG_15523 [Pyricularia oryzae 70-15]
MGG_15529	298.2765837	107.4307	0.360171418	-1.473244398	0.009654306	0.063507145	uncharacterized protein MGG_15529 [Pyricularia oryzae 70-15]
MGG_15564	36.4720086	15.50216045	0.425042685	-1.234320364	0.039161846	0.164274206	uncharacterized protein MGG_15564 [Pyricularia oryzae 70-15]
MGG_15569	1175.177842	582.70264	0.495842092	-1.012047347	0.011656381	0.07228265	uncharacterized protein MGG_15569 [Pyricularia oryzae 70-15]
MGG_15570	16.44648866	1.330482564	0.080897667	-3.627758091	0.000343803	0.005080923	uncharacterized protein MGG_15570 [Pyricularia oryzae 70-15]
MGG_15571	12.78039449	1.976326561	0.154637368	-2.693039111	0.008244541	0.056774737	uncharacterized protein MGG_15571 [Pyricularia oryzae 70-15]
MGG_15579	169.1241238	76.19796149	0.450544605	-1.150258152	0.008928951	0.060224412	uncharacterized protein MGG_15579 [Pyricularia oryzae 70-15]
MGG_15588	40.36375892	17.25921585	0.427591887	-1.225693614	0.033810801	0.149738831	uncharacterized protein MGG_15588 [Pyricularia oryzae 70-15]
MGG_15590	27.59722375	10.88798915	0.394532046	-1.341785607	0.044809208	0.18024578	uncharacterized protein MGG_15590 [Pyricularia oryzae 70-15]
MGG_15591	102.9663793	40.09802366	0.389428316	-1.360570308	0.016648069	0.091962003	uncharacterized protein MGG_15591 [Pyricularia oryzae 70-15]
MGG_15592	31.2416127	6.650408404	0.212870202	-2.231954083	0.001205074	0.013656001	uncharacterized protein MGG_15592 [Pyricularia oryzae 70-15]
MGG_15598	1248.062433	602.5362763	0.482777352	-1.050570097	0.007987608	0.055675695	uncharacterized protein MGG_15598 [Pyricularia oryzae 70-15]
MGG_15612	6944.014772	3384.720039	0.487429844	-1.036733508	0.033469342	0.148877909	uncharacterized protein MGG_15612 [Pyricularia oryzae 70-15]
MGG_15613	0.71134301	6.709841292	9.432638265	3.237661342	0.033525745	0.149038243	uncharacterized protein MGG_15613 [Pyricularia oryzae 70-15]
MGG_15618	27.47279532	70.70592375	2.573670532	1.363827379	0.009127743	0.061261778	hypothetical protein, variant [Pyricularia oryzae 70-15]
MGG_15619	4.992375916	20.39921815	4.086074145	2.030715383	0.011126805	0.070071764	uncharacterized protein MGG_15619 [Pyricularia oryzae 70-15]
MGG_15622	73.27877899	21.40631947	0.292121672	-1.7753587	0.000640307	0.00823988	uncharacterized protein MGG_15622 [Pyricularia oryzae 70-15]
MGG_15628	2128.177562	9332.623178	4.385265283	2.13266412	0.010846881	0.068807974	hypothetical protein, variant [Pyricularia oryzae 70-15]
MGG_15631	15.93219867	5.160087566	0.323877933	-1.626477921	0.04531585	0.181497051	uncharacterized protein MGG_15631 [Pyricularia oryzae 70-15]
MGG_15634	0.71134301	6.102010077	8.578154272	3.100667262	0.049904099	0.193249742	uncharacterized protein MGG_15634 [Pyricularia oryzae 70-15]
MGG_15635	294.4755492	135.0149553	0.458492923	-1.12502863	0.008785311	0.059456241	uncharacterized protein MGG_15635 [Pyricularia oryzae 70-15]
MGG_15649	52.78170499	127.6679651	2.418791987	1.274286705	0.006739019	0.049465623	uncharacterized protein MGG_15649 [Pyricularia oryzae 70-15]
MGG_15654	695.1075512	199.5873576	0.28713162	-1.800215878	2.36305E-05	0.000556323	uncharacterized protein MGG_15654 [Pyricularia oryzae 70-15]
MGG_15656	216.5256531	43.64018217	0.201547399	-2.310808931	0.00372494	0.031821783	uncharacterized protein MGG_15656 [Pyricularia oryzae 70-15]
MGG_15662	15.46852253	61.11044119	3.95063207	1.982083492	0.000441329	0.006161626	uncharacterized protein MGG_15662, partial
MGG_15668	2147.451632	715.7170685	0.3332867	-1.585164347	9.45343E-05	0.001797187	uncharacterized protein MGG_15668 [Pyricularia oryzae 70-15]
MGG_15688	629.4728718	229.2967897	0.364267945	-1.456928047	0.000479221	0.006602445	uncharacterized protein MGG_15688 [Pyricularia oryzae 70-15]
MGG_15690	23.73890001	4.02575578	0.169584765	-2.559921527	0.001261543	0.014203175	uncharacterized protein MGG_15690 [Pyricularia oryzae 70-15]
MGG_15695	5.873937768	18.84201823	3.207732014	1.681553619	0.030390267	0.139473769	uncharacterized protein MGG_15695 [Pyricularia oryzae 70-15]
MGG_15699	44.0467669	20.89345186	0.474347003	-1.075985263	0.046531314	0.18424459	uncharacterized protein MGG_15699 [Pyricularia oryzae 70-15]
MGG_15702	562.3469982	148.176672	0.263496867	-1.924142289	5.0238E-06	0.000152573	uncharacterized protein MGG_15702 [Pyricularia oryzae 70-15]
MGG_15703	32.91736801	10.43455442	0.316992368	-1.65747999	0.010757745	0.068493229	uncharacterized protein MGG_15703 [Pyricularia oryzae 70-15]
MGG_15726	94.19666562	34.22235319	0.36330748	-1.460737027	0.002836511	0.026303771	uncharacterized protein MGG_15726 [Pyricularia oryzae 70-15]
MGG_15734	55.32760631	12.05489193	0.217882044	-2.198380791	0.000117128	0.002140282	uncharacterized protein MGG_15734 [Pyricularia oryzae 70-15]

MGG_15742	35.04147668	14.23233341	0.406156782	-1.29989136	0.030445811	0.139673607	uncharacterized protein MGG_15742 [Pyricularia oryzae 70-15]
MGG_15753	587.868029	224.3255742	0.381591723	-1.389898216	0.000869297	0.010518152	uncharacterized protein MGG_15753 [Pyricularia oryzae 70-15]
MGG_15762	199.6635249	70.92723614	0.355233817	-1.493159166	0.001099445	0.012732181	uncharacterized protein MGG_15762 [Pyricularia oryzae 70-15]
MGG_15774	2989.141462	10025.5657	3.353995062	1.745880565	1.96378E-05	0.000478076	ketol-acid reductoisomerase [Pyricularia oryzae 70-15]
MGG_15775	125.940637	48.65954301	0.386368881	-1.371949194	0.008678157	0.058960989	hypothetical protein, variant [Pyricularia oryzae 70-15]
MGG_15777	100.0030122	14.93560587	0.14935156	-2.743215788	5.43206E-05	0.001126086	hypothetical protein, variant [Pyricularia oryzae 70-15]
MGG_15788	37.05590072	5.302551376	0.143096006	-2.804944694	5.1555E-05	0.001072414	uncharacterized protein MGG_15788 [Pyricularia oryzae 70-15]
MGG_15795	7.255351314	0.913837995	0.125953652	-2.989035141	0.038114625	0.161554943	uncharacterized protein MGG_15795 [Pyricularia oryzae 70-15]
MGG_15798	249.6386233	62.23872292	0.249315279	-2.003956798	7.43181E-06	0.000213937	uncharacterized protein MGG_15798 [Pyricularia oryzae 70-15]
MGG_15821	17.61277742	4.542328086	0.257899591	-1.955118612	0.017080746	0.093656418	uncharacterized protein MGG_15821 [Pyricularia oryzae 70-15]
MGG_15833	85.01072075	30.12349475	0.354349363	-1.496755639	0.0020897	0.020893561	uncharacterized protein MGG_15833 [Pyricularia oryzae 70-15]
MGG_15836	51.84137787	15.2642922	0.294442255	-1.76394337	0.002290327	0.022383658	uncharacterized protein MGG_15836 [Pyricularia oryzae 70-15]
MGG_15840	162.3387675	72.51047935	0.446661512	-1.162746149	0.008552097	0.05836566	uncharacterized protein MGG_15840 [Pyricularia oryzae 70-15]
MGG_15841	84.22125518	18.94520982	0.224945707	-2.152351259	4.0099E-05	0.000872979	uncharacterized protein MGG_15841 [Pyricularia oryzae 70-15]
MGG_15849	12.40497122	151.7349823	12.23178834	3.612563442	1.13337E-10	1.09272E-08	uncharacterized protein MGG_15849 [Pyricularia oryzae 70-15]
MGG_15863	60.90506433	27.64130594	0.453842488	-1.139736416	0.028445179	0.132904627	uncharacterized protein MGG_15863 [Pyricularia oryzae 70-15]
MGG_15869	24.62134634	6.635516007	0.269502566	-1.891629088	0.017243868	0.094232347	uncharacterized protein MGG_15869 [Pyricularia oryzae 70-15]
MGG_15877	31.28200069	4.766563381	0.152373994	-2.7143114	0.00015392	0.002656528	uncharacterized protein MGG_15877 [Pyricularia oryzae 70-15]
MGG_15879	4.598575385	15.55088329	3.381674103	1.757737632	0.033904523	0.149909537	uncharacterized protein MGG_15879 [Pyricularia oryzae 70-15]
MGG_15886	1429.307714	72.3981781	0.050652618	-4.303219344	0.005472291	0.042586414	uncharacterized protein MGG_15886 [Pyricularia oryzae 70-15]
MGG_15892	21.84289097	1.577838244	0.072235779	-3.791142597	4.20803E-05	0.000904763	uncharacterized protein MGG_15892 [Pyricularia oryzae 70-15]
MGG_15905	226.7774412	34.20245985	0.150819498	-2.729105142	0.000547145	0.007270124	uncharacterized protein MGG_15905 [Pyricularia oryzae 70-15]
MGG_15912	237.0821475	82.96489031	0.349941534	-1.51481419	0.000480454	0.006602445	uncharacterized protein MGG_15912 [Pyricularia oryzae 70-15]
MGG_15915	19.9104329	62.82625676	3.155444037	1.657843037	0.002246927	0.022077066	uncharacterized protein MGG_15915 [Pyricularia oryzae 70-15]
MGG_15920	2125.338128	753.825846	0.354685137	-1.495389218	0.000188908	0.003139337	uncharacterized protein MGG_15920 [Pyricularia oryzae 70-15]
MGG_15930	14.61644829	1476.640751	101.0259621	6.658582281	2.33731E-16	6.76039E-14	uncharacterized protein MGG_15930 [Pyricularia oryzae 70-15]
MGG_15938	29.20673693	218.1620855	7.469580942	2.901027307	2.15498E-08	1.21761E-06	uncharacterized protein MGG_15938 [Pyricularia oryzae 70-15]
MGG_15943	0	7.210739413	Inf	Inf	0.003225092	0.028493392	uncharacterized protein MGG_15943, partial
MGG_15955	80.79556095	29.37153626	0.363529084	-1.459857304	0.004201465	0.034818332	uncharacterized protein MGG_15955 [Pyricularia oryzae 70-15]
MGG_15970	24.41318298	86.73040243	3.552605266	1.828877398	0.00035279	0.005182211	uncharacterized protein MGG_15970, partial
MGG_15977	6.901938779	48.19866209	6.983351146	2.803919518	1.00468E-05	0.000276753	uncharacterized protein MGG_15977 [Pyricularia oryzae 70-15]
MGG_15983	0.660729151	8.573047998	12.97513207	3.697677319	0.010170484	0.066126149	uncharacterized protein MGG_15983 [Pyricularia oryzae 70-15]
MGG_15992	1933.03351	8601.22711	4.449600623	2.153675852	9.69043E-05	0.001836495	uncharacterized protein MGG_15992 [Pyricularia oryzae 70-15]
MGG_15993	249.7180497	9996.48958	40.03110544	5.323049551	8.77554E-14	1.56772E-11	uncharacterized protein MGG_15993 [Pyricularia oryzae 70-15]
MGG_15995	756.0586921	1833.531853	2.42511841	1.27805519	0.001686611	0.017739346	uncharacterized protein MGG_15995 [Pyricularia oryzae 70-15]
MGG_15996	59.12866027	169.4864275	2.866400603	1.519240252	0.000835086	0.010205859	uncharacterized protein MGG_15996 [Pyricularia oryzae 70-15]
MGG_15999	34.6281089	9.012812951	0.260274478	-1.941894247	0.002485202	0.023753136	uncharacterized protein MGG_15999 [Pyricularia oryzae 70-15]
MGG_16001	13.47336023	36.39357109	2.701150304	1.43357392	0.018597838	0.099145935	uncharacterized protein MGG_16001 [Pyricularia oryzae 70-15]
MGG_16006	9.173950059	1.23551896	0.134676879	-2.892425903	0.022895945	0.114413797	uncharacterized protein MGG_16006 [Pyricularia oryzae 70-15]
MGG_16011	932.10723	213.1599993	0.228686134	-2.128559199	3.9032E-07	1.61279E-05	uncharacterized protein MGG_16011 [Pyricularia oryzae 70-15]
MGG_16019	23.5584553	9.470734157	0.402009981	-1.314696776	0.046821697	0.184972349	uncharacterized protein MGG_16019 [Pyricularia oryzae 70-15]
MGG_16025	70.26397704	156.7648797	2.231084637	1.157745244	0.009341039	0.062109985	uncharacterized protein MGG_16025 [Pyricularia oryzae 70-15]
MGG_16027	2888.430788	338.0837265	0.117047543	-3.094833446	0.011332075	0.071033049	uncharacterized protein MGG_16027 [Pyricularia oryzae 70-15]
MGG_16028	41.47128242	12.59754434	0.303765488	-1.718970126	0.004092097	0.034212525	uncharacterized protein MGG_16028 [Pyricularia oryzae 70-15]
MGG_16029	9.783180874	1.92512166	0.196778705	-2.345353994	0.033571889	0.149061148	uncharacterized protein MGG_16029 [Pyricularia oryzae 70-15]
MGG_16037	3.051460937	13.42835141	4.400630284	2.13771017	0.027752159	0.130778955	uncharacterized protein MGG_16037 [Pyricularia oryzae 70-15]
MGG_16045	1.404308753	14.0424062	9.999514829	3.321858098	0.001763676	0.018359157	uncharacterized protein MGG_16045 [Pyricularia oryzae 70-15]
MGG_16053	2854.422553	6687.655455	2.342910109	1.228301603	0.04246991	0.173770452	uncharacterized protein MGG_16053 [Pyricularia oryzae 70-15]

MGG_16055	2.097274496	22.22889856	10.59894573	3.405848863	0.000119166	0.002163874	uncharacterized protein MGG_16055, partial
MGG_16063	2107.89669	313.1993414	0.14858382	-2.750651077	1.79088E-09	1.29498E-07	uncharacterized protein MGG_16063 [Pyricularia oryzae 70-15]
MGG_16069	257.0273754	114.4192559	0.445163694	-1.167592158	0.012219164	0.074622274	uncharacterized protein MGG_16069 [Pyricularia oryzae 70-15]
MGG_16081	83.30461219	310.4734796	3.726966268	1.898001762	0.001872274	0.019258585	uncharacterized protein MGG_16081 [Pyricularia oryzae 70-15]
MGG_16084	1640.633766	418.0897798	0.254834314	-1.972368544	0.000340068	0.005049232	uncharacterized protein MGG_16084 [Pyricularia oryzae 70-15]
MGG_16087	2.845372039	13.60512331	4.781491884	2.257460827	0.015432379	0.087006487	uncharacterized protein MGG_16087 [Pyricularia oryzae 70-15]
MGG_16089	0	10.66623592	Inf	Inf	0.000190679	0.003160127	uncharacterized protein MGG_16089 [Pyricularia oryzae 70-15]
MGG_16091	0.321175942	11.47065875	35.71456404	5.158440606	0.044398346	0.179126905	uncharacterized protein MGG_16091 [Pyricularia oryzae 70-15]
MGG_16092	11.54001768	98.15774332	8.505857271	3.088456647	6.00901E-06	0.000178043	uncharacterized protein MGG_16092 [Pyricularia oryzae 70-15]
MGG_16093	70.60420477	419.791526	5.945701497	2.571847035	8.0089E-09	5.04104E-07	uncharacterized protein MGG_16093, partial
MGG_16094	16.9186217	163.7244281	9.677172942	3.274585645	0.000291384	0.004502139	uncharacterized protein MGG_16094 [Pyricularia oryzae 70-15]
MGG_16095	9.411391073	36.60046873	3.888954188	1.95938224	0.002206497	0.021721662	uncharacterized protein MGG_16095 [Pyricularia oryzae 70-15]
MGG_16099	15.01024882	100.0647823	6.666430621	2.736914512	4.13761E-07	1.6867E-05	uncharacterized protein MGG_16099 [Pyricularia oryzae 70-15]
MGG_16102	9.462889409	50.6074441	5.347990652	2.418996944	0.047689218	0.187242604	uncharacterized protein MGG_16102 [Pyricularia oryzae 70-15]
MGG_16103	9.466522873	53.07983528	5.607110023	2.487257379	0.045966924	0.182964023	uncharacterized protein MGG_16103, partial
MGG_16114	275.2904142	82.64022335	0.300192884	-1.736038316	8.92904E-05	0.001702824	uncharacterized protein MGG_16114 [Pyricularia oryzae 70-15]
MGG_16131	231.9295051	80.68041585	0.347866115	-1.52339594	0.000481867	0.006614375	uncharacterized protein MGG_16131 [Pyricularia oryzae 70-15]
MGG_16141	3647.780052	1217.235608	0.333692161	-1.583410299	9.50689E-05	0.001804527	uncharacterized protein MGG_16141 [Pyricularia oryzae 70-15]
MGG_16142	142.363925	50.89934027	0.357529762	-1.483864751	0.008047085	0.0557968	uncharacterized protein MGG_16142 [Pyricularia oryzae 70-15]
MGG_16148	407.4225823	166.634543	0.408996826	-1.289838447	0.002088703	0.020893561	uncharacterized protein MGG_16148 [Pyricularia oryzae 70-15]
MGG_16156	24.82053734	7.350721157	0.296154795	-1.755576648	0.019916146	0.10407944	uncharacterized protein MGG_16156 [Pyricularia oryzae 70-15]
MGG_16162	184.3069714	85.95851379	0.46638775	-1.100398198	0.010678748	0.06820475	uncharacterized protein MGG_16162 [Pyricularia oryzae 70-15]
MGG_16173	774.69828	196.3225919	0.253418133	-1.980408336	2.18871E-06	7.35766E-05	uncharacterized protein MGG_16173 [Pyricularia oryzae 70-15]
MGG_16181	742.9923934	209.1574112	0.2815068	-1.828758322	1.33627E-05	0.000347602	uncharacterized protein MGG_16181 [Pyricularia oryzae 70-15]
MGG_16182	49.38892189	14.47846646	0.293152106	-1.770278675	0.002038626	0.020517998	uncharacterized protein MGG_16182 [Pyricularia oryzae 70-15]
MGG_16187	619.4509711	1531.39579	2.472182403	1.305785192	0.022484084	0.113261817	uncharacterized protein MGG_16187 [Pyricularia oryzae 70-15]
MGG_16190	17.89600885	52.5485809	2.936329621	1.554013929	0.005772952	0.044414075	uncharacterized protein MGG_16190 [Pyricularia oryzae 70-15]
MGG_16198	80.41235531	33.56488065	0.417409495	-1.260464675	0.01149168	0.071594554	uncharacterized protein MGG_16198 [Pyricularia oryzae 70-15]
MGG_16209	1.66101151	9.255204499	5.572029116	2.478202797	0.038022482	0.161389625	uncharacterized protein MGG_16209 [Pyricularia oryzae 70-15]
MGG_16214	29.40015648	8.369451021	0.28467369	-1.812618934	0.00601257	0.045764848	uncharacterized protein MGG_16214 [Pyricularia oryzae 70-15]
MGG_16216	95.46313855	20.21625949	0.211770321	-2.239427681	0.037103152	0.158261621	uncharacterized protein MGG_16216 [Pyricularia oryzae 70-15]
MGG_16220	2250.481949	5979.355217	2.656922096	1.409755927	0.000524652	0.007058112	uncharacterized protein MGG_16220 [Pyricularia oryzae 70-15]
MGG_16228	184.4302098	91.01140093	0.49347339	-1.018955803	0.018560321	0.099108034	hypothetical protein, variant [Pyricularia oryzae 70-15]
MGG_16233	149.1163386	40.28682813	0.270170449	-1.888058213	6.32639E-05	0.001280883	uncharacterized protein MGG_16233 [Pyricularia oryzae 70-15]
MGG_16242	110.975377	29.06549267	0.261909384	-1.932860342	0.001032168	0.012079742	uncharacterized protein MGG_16242 [Pyricularia oryzae 70-15]
MGG_16244	239.1996511	117.7416551	0.492231717	-1.022590473	0.017781636	0.096372795	uncharacterized protein MGG_16244 [Pyricularia oryzae 70-15]
MGG_16253	133.8798226	46.68195701	0.348685531	-1.520001597	0.001082946	0.012565072	uncharacterized protein MGG_16253 [Pyricularia oryzae 70-15]
MGG_16269	228.8643313	73.44407399	0.320906598	-1.639774642	0.000236295	0.003796972	uncharacterized protein MGG_16269 [Pyricularia oryzae 70-15]
MGG_16278	126.2744188	59.94298714	0.474704122	-1.074899517	0.015187906	0.086256515	uncharacterized protein MGG_16278 [Pyricularia oryzae 70-15]
MGG_16292	50.32982798	14.28353831	0.283798671	-1.81706026	0.001266488	0.01424155	uncharacterized protein MGG_16292 [Pyricularia oryzae 70-15]
MGG_16296	160.1440452	24.67232961	0.15406336	-2.698404304	5.026E-08	2.58711E-06	uncharacterized protein MGG_16296 [Pyricularia oryzae 70-15]
MGG_16297	6.763956528	0	0	-Inf	0.003406345	0.029620812	uncharacterized protein MGG_16297 [Pyricularia oryzae 70-15]
MGG_16298	728.4268965	3254.428806	4.467749367	2.159548256	0.007936334	0.055440242	uncharacterized protein MGG_16298 [Pyricularia oryzae 70-15]
MGG_16299	386.006498	1241.129475	3.215307208	1.684956587	4.57257E-05	0.000974519	uncharacterized protein MGG_16299 [Pyricularia oryzae 70-15]
MGG_16304	94.01985438	28.5650722	0.303819575	-1.71871327	0.000485636	0.006643591	uncharacterized protein MGG_16304 [Pyricularia oryzae 70-15]
MGG_16318	236.5663305	112.4853813	0.475491931	-1.072507236	0.011221723	0.070486808	uncharacterized protein MGG_16318 [Pyricularia oryzae 70-15]
MGG_16326	93.59085833	298.915604	3.193854714	1.675298687	0.000153951	0.002656528	uncharacterized protein MGG_16326 [Pyricularia oryzae 70-15]
MGG_16328	3.033083671	23.51684505	7.753444218	2.954837324	0.000424627	0.005956541	uncharacterized protein MGG_16328 [Pyricularia oryzae 70-15]

MGG_16333	709.5069715	287.4340047	0.405117943	-1.303586111	0.001506917	0.016185701	uncharacterized protein MGG_16333 [Pyricularia oryzae 70-15]
MGG_16341	12268.60686	5688.796783	0.463687267	-1.108775985	0.006315393	0.04750427	uncharacterized protein MGG_16341 [Pyricularia oryzae 70-15]
MGG_16347	20.36418867	56.63869689	2.781289145	1.475753736	0.008590136	0.058461047	uncharacterized protein MGG_16347 [Pyricularia oryzae 70-15]
MGG_16354	2.964092545	16.7719875	5.65838861	2.500391262	0.023263641	0.115822421	uncharacterized protein MGG_16354 [Pyricularia oryzae 70-15]
MGG_16355	61.78476167	17.6552221	0.285753665	-1.807156092	0.00810798	0.05611776	uncharacterized protein MGG_16355 [Pyricularia oryzae 70-15]
MGG_16361	1135.303035	408.6415193	0.35994048	-1.474169734	0.032327537	0.145611762	H/K ATPase alpha subunit [Pyricularia oryzae 70-15]
MGG_16364	603.9668257	207.8994327	0.344223265	-1.538583487	0.000176005	0.002961363	uncharacterized protein MGG_16364 [Pyricularia oryzae 70-15]
MGG_16374	767.4289937	2688.495746	3.503250161	1.808694012	0.002788928	0.025941726	uncharacterized protein MGG_16374 [Pyricularia oryzae 70-15]
MGG_16375	271.1418655	2538.926159	9.363829351	3.227098643	0.000124568	0.002240985	aldehyde reductase [Pyricularia oryzae 70-15]
MGG_16379	131.0947749	49.9510943	0.381030398	-1.392021995	0.019588061	0.102903445	uncharacterized protein MGG_16379 [Pyricularia oryzae 70-15]
MGG_16382	17.26840077	216.0211871	12.50962321	3.644966431	5.89862E-13	8.73859E-11	uncharacterized protein MGG_16382 [Pyricularia oryzae 70-15]
MGG_16392	1216.546854	3429.597128	2.81912457	1.495247228	0.002606086	0.024579758	uncharacterized protein MGG_16392 [Pyricularia oryzae 70-15]
MGG_16397	0.679106417	7.795450243	11.47898186	3.520922781	0.017834873	0.096463951	uncharacterized protein MGG_16397 [Pyricularia oryzae 70-15]
MGG_16399	61.02564935	205.5893987	3.368901451	1.752278227	0.000534287	0.007142835	uncharacterized protein MGG_16399 [Pyricularia oryzae 70-15]
MGG_16403	57.87598315	967.8607949	16.72301259	4.063762862	3.69039E-18	1.35851E-15	uncharacterized protein MGG_16403 [Pyricularia oryzae 70-15]
MGG_16404	0	10.6418561	Inf	Inf	0.00021875	0.003557403	uncharacterized protein MGG_16404 [Pyricularia oryzae 70-15]
MGG_16411	4.570856733	37.60522469	8.227172033	3.040396611	0.006497075	0.048421146	uncharacterized protein MGG_16411 [Pyricularia oryzae 70-15]
MGG_16417	1633.695245	3352.619497	2.052169465	1.037149872	0.010005558	0.065488968	uncharacterized protein MGG_16417 [Pyricularia oryzae 70-15]
MGG_16426	634.5922147	181.5460207	0.286082962	-1.805494514	1.87048E-05	0.000460906	uncharacterized protein MGG_16426 [Pyricularia oryzae 70-15]
MGG_16431	0.339553209	5.772882913	17.00140881	4.087582394	0.03033024	0.13930123	uncharacterized protein MGG_16431 [Pyricularia oryzae 70-15]
MGG_16432	795.3784021	181.4862731	0.228176014	-2.131780955	0.030925814	0.140891933	uncharacterized protein MGG_16432 [Pyricularia oryzae 70-15]
MGG_16452	115.5593677	50.57599584	0.437662449	-1.192109488	0.010663683	0.068144359	uncharacterized protein MGG_16452 [Pyricularia oryzae 70-15]
MGG_16453	61.5157586	16.86647345	0.274181345	-1.866797678	0.000750027	0.009337677	uncharacterized protein MGG_16453 [Pyricularia oryzae 70-15]
MGG_16461	193.0895644	80.48810647	0.416843379	-1.262422675	0.004000658	0.033585188	uncharacterized protein MGG_16461 [Pyricularia oryzae 70-15]
MGG_16463	65.58078225	32.31290571	0.492719126	-1.021162621	0.045127732	0.181227005	uncharacterized protein MGG_16463 [Pyricularia oryzae 70-15]
MGG_16474	94.93127287	326.0451974	3.434539405	1.780116637	0.025004119	0.121541222	uncharacterized protein MGG_16474 [Pyricularia oryzae 70-15]
MGG_16477	412.7125964	201.0694504	0.487190001	-1.03744357	0.015331693	0.086734458	uncharacterized protein MGG_16477 [Pyricularia oryzae 70-15]
MGG_16478	4.620586115	19.84911955	4.295801237	2.102927243	0.048182008	0.188083237	uncharacterized protein MGG_16478, partial
MGG_16509	122.4990599	46.09755032	0.376309421	-1.410008686	0.013654042	0.080587337	uncharacterized protein MGG_16509 [Pyricularia oryzae 70-15]
MGG_16517	168.4416894	50.49867403	0.299799142	-1.737931838	0.000132077	0.002345391	uncharacterized protein MGG_16517 [Pyricularia oryzae 70-15]
MGG_16522	176.4560386	25.60636508	0.1451147	-2.784734426	1.22633E-08	7.52397E-07	uncharacterized protein MGG_16522 [Pyricularia oryzae 70-15]
MGG_16534	58.94311865	18.15071525	0.30793612	-1.699296994	0.001735396	0.018126903	uncharacterized protein MGG_16534 [Pyricularia oryzae 70-15]
MGG_16535	0.339553209	21.21557367	62.48085167	5.965342214	0.000751657	0.009337677	uncharacterized protein MGG_16535 [Pyricularia oryzae 70-15]
MGG_16540	6.116781201	0	0	-Inf	0.005961499	0.045441286	uncharacterized protein MGG_16540 [Pyricularia oryzae 70-15]
MGG_16543	136.5556504	304.3902366	2.229056328	1.156433074	0.041868696	0.172238713	uncharacterized protein MGG_16543 [Pyricularia oryzae 70-15]
MGG_16544	0.743579602	10.26648816	13.80684481	3.787311762	0.003544197	0.030427493	uncharacterized protein MGG_16544, partial
MGG_16550	239.1199187	652.660932	2.729429382	1.448599371	0.007153247	0.05176279	uncharacterized protein MGG_16550 [Pyricularia oryzae 70-15]
MGG_16556	0.321175942	14.91744962	46.44634814	5.537493264	5.64931E-05	0.001165158	uncharacterized protein MGG_16556 [Pyricularia oryzae 70-15]
MGG_16568	0	5.333117959	Inf	Inf	0.01316066	0.078756503	uncharacterized protein MGG_16568, partial
MGG_16590	286.62783	819.6387541	2.859592365	1.515809505	0.000315225	0.004768808	uncharacterized protein MGG_16590 [Pyricularia oryzae 70-15]
MGG_16591	749.7595503	1721.527521	2.296106159	1.199189345	0.049679401	0.192628586	uncharacterized protein MGG_16591 [Pyricularia oryzae 70-15]
MGG_16595	8770.377314	3461.921512	0.394728914	-1.341065894	0.00088855	0.010697822	uncharacterized protein MGG_16595 [Pyricularia oryzae 70-15]
MGG_16600	48.7666527	283.8990715	5.821582082	2.541411275	1.83354E-07	8.04108E-06	uncharacterized protein MGG_16600 [Pyricularia oryzae 70-15]
MGG_16601	8.19567843	25.88176847	3.157977548	1.659000914	0.017092219	0.093656418	uncharacterized protein MGG_16601 [Pyricularia oryzae 70-15]
MGG_16605	1.66101151	11.63127881	7.002527521	2.807875748	0.010730076	0.06838875	uncharacterized protein MGG_16605 [Pyricularia oryzae 70-15]
MGG_16607	116.8974023	331.3139228	2.834228275	1.502955961	0.000547593	0.007270124	uncharacterized protein MGG_16607 [Pyricularia oryzae 70-15]
MGG_16608	451.0876705	146.2703885	0.324261553	-1.624770119	0.000134033	0.002370071	uncharacterized protein MGG_16608 [Pyricularia oryzae 70-15]
MGG_16615	199.5098823	432.1107396	2.165861332	1.114940878	0.031017695	0.141177579	uncharacterized protein MGG_16615 [Pyricularia oryzae 70-15]

MGG_16623	10.70964866	78.4168163	7.322071784	2.872251918	0.000147423	0.002562088	uncharacterized protein MGG_16623 [Pyricularia oryzae 70-15]
MGG_16624	12.48330373	0	0	-Inf	0.004003239	0.033585188	uncharacterized protein MGG_16624 [Pyricularia oryzae 70-15]
MGG_16627	3.469346656	14.32281053	4.12838841	2.04557871	0.023476644	0.116405826	uncharacterized protein MGG_16627 [Pyricularia oryzae 70-15]
MGG_16628	0.71134301	17.70768644	24.89331617	4.637686527	3.54637E-05	0.000791937	uncharacterized protein MGG_16628 [Pyricularia oryzae 70-15]
MGG_16629	1662.648417	3892.22234	2.340977383	1.227110996	0.013210207	0.078904758	uncharacterized protein MGG_16629 [Pyricularia oryzae 70-15]
MGG_16635	56.95638123	17.77656995	0.312108487	-1.679880509	0.001982468	0.020102688	uncharacterized protein MGG_16635 [Pyricularia oryzae 70-15]
MGG_16643	11.34836708	55.5184389	4.892196251	2.290482279	0.010744794	0.068446646	uncharacterized protein MGG_16643, partial
MGG_16647	66.84472879	135.4607027	2.026497903	1.018988683	0.030881367	0.140820888	uncharacterized protein MGG_16647 [Pyricularia oryzae 70-15]
MGG_16650	190.595626	73.07985695	0.383428825	-1.382969296	0.004634091	0.037680683	uncharacterized protein MGG_16650 [Pyricularia oryzae 70-15]
MGG_16654	33.11737996	217.0835661	6.554974045	2.712590068	2.72614E-06	8.95057E-05	uncharacterized protein MGG_16654 [Pyricularia oryzae 70-15]
MGG_16655	133.732162	566.6690804	4.237343298	2.083160017	9.7266E-07	3.68096E-05	uncharacterized protein MGG_16655 [Pyricularia oryzae 70-15]
MGG_16657	1.014141685	16.20169142	15.97576715	3.997813305	0.00024237	0.0038741	uncharacterized protein MGG_16657 [Pyricularia oryzae 70-15]
MGG_16660	4.354541973	62.16007859	14.2747685	3.835395444	3.52953E-06	0.000110793	uncharacterized protein MGG_16660 [Pyricularia oryzae 70-15]
MGG_16670	1.064755544	13.20289349	12.39992932	3.632259992	0.001417299	0.015469319	uncharacterized protein MGG_16670 [Pyricularia oryzae 70-15]
MGG_16674	0	31.09884367	Inf	Inf	0.000163889	0.002796246	uncharacterized protein MGG_16674, partial
MGG_16683	32.82880964	2.226164307	0.067811301	-3.882330456	1.19463E-06	4.39769E-05	uncharacterized protein MGG_16683, partial
MGG_16696	1.018659626	13.85121955	13.59749537	3.76526903	0.000989276	0.011667693	uncharacterized protein MGG_16696 [Pyricularia oryzae 70-15]
MGG_16699	4.042707415	0	0	-Inf	0.035140803	0.153502508	uncharacterized protein MGG_16699 [Pyricularia oryzae 70-15]
MGG_16704	45.26305852	270.0154177	5.96547	2.576635808	0.000973203	0.011534118	uncharacterized protein MGG_16704 [Pyricularia oryzae 70-15]
MGG_16708	94.80174752	41.48215638	0.437567423	-1.192422761	0.036138677	0.155844036	uncharacterized protein MGG_16708 [Pyricularia oryzae 70-15]
MGG_16709	22.57655021	52.99705149	2.347437983	1.231087044	0.024855289	0.120970372	uncharacterized protein MGG_16709 [Pyricularia oryzae 70-15]
MGG_16710	20.96340357	72.70907539	3.468381226	1.794262481	0.000629213	0.00814024	uncharacterized protein MGG_16710 [Pyricularia oryzae 70-15]
MGG_16718	1630.033205	469.6453069	0.288120086	-1.795257856	1.25354E-05	0.000328898	uncharacterized protein MGG_16718 [Pyricularia oryzae 70-15]
MGG_16720	629.508996	309.1568307	0.491107884	-1.025888113	0.011065859	0.069905385	uncharacterized protein MGG_16720 [Pyricularia oryzae 70-15]
MGG_16723	0.660729151	10.34207289	15.65251491	3.968322571	0.003141404	0.028184468	uncharacterized protein MGG_16723 [Pyricularia oryzae 70-15]
MGG_16724	509.7946846	250.2737089	0.490930401	-1.026409586	0.013536848	0.080118556	uncharacterized protein MGG_16724 [Pyricularia oryzae 70-15]
MGG_16741	745.4950433	196.5787418	0.263688865	-1.923091444	0.000304863	0.004635139	uncharacterized protein MGG_16741 [Pyricularia oryzae 70-15]
MGG_16743	1467.073962	660.1092752	0.449949554	-1.152164831	0.005144137	0.040657756	uncharacterized protein MGG_16743 [Pyricularia oryzae 70-15]
MGG_16764	514.1755961	252.3096358	0.490707139	-1.027065834	0.013941871	0.081622098	uncharacterized protein MGG_16764 [Pyricularia oryzae 70-15]
MGG_16765	76.96382941	30.33906122	0.394198956	-1.343004139	0.007314716	0.052517239	uncharacterized protein MGG_16765 [Pyricularia oryzae 70-15]
MGG_16773	4.134593748	21.39931412	5.17567515	2.37174707	0.003287037	0.028872687	uncharacterized protein MGG_16773 [Pyricularia oryzae 70-15]
MGG_16784	28.74809418	84.26776601	2.931247041	1.551514561	0.013114585	0.078596933	uncharacterized protein MGG_16784 [Pyricularia oryzae 70-15]
MGG_16792	2490.215471	12488.37441	5.014977442	2.326243212	2.29049E-08	1.28819E-06	uncharacterized protein MGG_16792 [Pyricularia oryzae 70-15]
MGG_16793	353.4391195	789.3752145	2.233412124	1.159249491	0.004581786	0.037305316	actin-like protein [Pyricularia oryzae 70-15]
MGG_16796	25.79429102	244.9529689	9.496402467	3.247381079	1.90046E-11	2.09879E-09	uncharacterized protein MGG_16796 [Pyricularia oryzae 70-15]
MGG_16814	79.84388152	300.994236	3.769784613	1.914482098	0.031603202	0.143038634	uncharacterized protein MGG_16814, partial
MGG_16826	1230.356159	502.2095896	0.408182286	-1.29271452	0.007105728	0.051503811	uncharacterized protein MGG_16826 [Pyricularia oryzae 70-15]
MGG_16830	442.6074757	139.1200635	0.314319281	-1.669697322	8.84161E-05	0.001688802	uncharacterized protein MGG_16830 [Pyricularia oryzae 70-15]
MGG_16831	290.3884895	17.04242675	0.058688369	-4.090781581	0.026695055	0.127623585	uncharacterized protein MGG_16831 [Pyricularia oryzae 70-15]
MGG_16845	18.68479989	4.743442996	0.253866406	-1.977858598	0.026425383	0.126571919	uncharacterized protein MGG_16845 [Pyricularia oryzae 70-15]
MGG_16853	281.1802537	113.171012	0.402485631	-1.312990814	0.002397914	0.023100702	uncharacterized protein MGG_16853 [Pyricularia oryzae 70-15]
MGG_16864	187.7402381	84.4657845	0.449907731	-1.152298938	0.008026989	0.05574274	uncharacterized protein MGG_16864 [Pyricularia oryzae 70-15]
MGG_16868	2759.514472	896.9280954	0.325031126	-1.621350214	0.002176629	0.021497309	uncharacterized protein MGG_16868 [Pyricularia oryzae 70-15]
MGG_16871	21.64702793	3.454237068	0.159570962	-2.647729956	0.00146359	0.015804168	uncharacterized protein MGG_16871 [Pyricularia oryzae 70-15]
MGG_16887	2709.652584	1049.343255	0.387261179	-1.368621208	0.001864166	0.01919143	uncharacterized protein MGG_16887 [Pyricularia oryzae 70-15]
MGG_16891	1.725484695	12.44889377	7.2147228	2.850943965	0.007442233	0.053212624	uncharacterized protein MGG_16891 [Pyricularia oryzae 70-15]
MGG_16905	180.1697877	71.75189326	0.398245978	-1.328268305	0.011343134	0.071057623	uncharacterized protein MGG_16905 [Pyricularia oryzae 70-15]
MGG_16914	198.5440254	65.2332018	0.328557869	-1.605780602	0.000372947	0.005387752	uncharacterized protein MGG_16914 [Pyricularia oryzae 70-15]

MGG_16915	2130.695502	728.5793228	0.341944366	-1.548166476	0.000155211	0.002670693	uncharacterized protein MGG_16915 [Pyricularia oryzae 70-15]
MGG_16917	883.905226	236.9535439	0.268075736	-1.899287451	5.19394E-06	0.000155793	uncharacterized protein MGG_16917 [Pyricularia oryzae 70-15]
MGG_16921	1726.444554	811.8871782	0.470265423	-1.088452835	0.005669998	0.043760571	uncharacterized protein MGG_16921 [Pyricularia oryzae 70-15]
MGG_16924	11.94944649	81.26022563	6.800333862	2.765605577	5.88212E-05	0.001208352	uncharacterized protein MGG_16924 [Pyricularia oryzae 70-15]
MGG_16925	9.057978538	34.52788232	3.811875042	1.930500827	0.003535501	0.030385807	hypothetical protein, variant [Pyricularia oryzae 70-15]
MGG_16926	44.76045784	106.3360077	2.375668454	1.248333509	0.022287656	0.112671847	uncharacterized protein MGG_16926 [Pyricularia oryzae 70-15]
MGG_16928	105.3888196	24.05161042	0.228217856	-2.131516423	0.021520913	0.109893255	uncharacterized protein MGG_16928, partial
MGG_16929	49.36171869	183.7653794	3.722831869	1.896400462	2.82652E-05	0.000652786	uncharacterized protein MGG_16929 [Pyricularia oryzae 70-15]
MGG_16947	18.0339911	83.08919787	4.607366023	2.203942215	3.43597E-05	0.000770115	uncharacterized protein MGG_16947 [Pyricularia oryzae 70-15]
MGG_16951	13.87650215	87.20522045	6.284380568	2.65177055	1.39292E-06	5.00626E-05	uncharacterized protein MGG_16951 [Pyricularia oryzae 70-15]
MGG_16952	0.679106417	38.15685006	56.18684948	5.812160602	3.95958E-08	2.0823E-06	uncharacterized protein MGG_16952 [Pyricularia oryzae 70-15]
MGG_16956	46.92526001	493.4264614	10.51515668	3.394398442	2.70314E-13	4.37836E-11	uncharacterized protein MGG_16956 [Pyricularia oryzae 70-15]
MGG_16961	1.064755544	10.65382559	10.00588881	3.322777421	0.026794733	0.127949064	uncharacterized protein MGG_16961 [Pyricularia oryzae 70-15]
MGG_16964	0	28.43131402	Inf	Inf	6.29541E-07	2.47497E-05	uncharacterized protein MGG_16964 [Pyricularia oryzae 70-15]
MGG_16965	258.9400116	3805.684547	14.69716682	3.877466168	6.1958E-09	4.00354E-07	uncharacterized protein MGG_16965 [Pyricularia oryzae 70-15]
MGG_16966	196.406217	428.6459967	2.182446173	1.125946072	0.007612945	0.054019894	uncharacterized protein MGG_16966 [Pyricularia oryzae 70-15]
MGG_16970	283.3490075	590.2525101	2.083128914	1.058752123	0.013310074	0.079244576	uncharacterized protein MGG_16970 [Pyricularia oryzae 70-15]
MGG_16971	0	12.6557546	Inf	Inf	4.572E-05	0.000974519	uncharacterized protein MGG_16971 [Pyricularia oryzae 70-15]
MGG_16977	0	20.76465782	Inf	Inf	2.0944E-07	8.95872E-06	uncharacterized protein MGG_16977 [Pyricularia oryzae 70-15]
MGG_16978	2.891467956	29.25963903	10.11930254	3.339037952	1.96202E-05	0.000478076	uncharacterized protein MGG_16978 [Pyricularia oryzae 70-15]
MGG_16998	6.245727569	25.58068902	4.095710025	2.034113576	0.01259826	0.076333542	uncharacterized protein MGG_16998 [Pyricularia oryzae 70-15]
MGG_17010	5.144217492	19.72528964	3.834458724	1.93902294	0.01369929	0.080629346	uncharacterized protein MGG_17010 [Pyricularia oryzae 70-15]
MGG_17014	3.37263688	27.87050536	8.263713634	3.046790261	0.0001068	0.001989896	uncharacterized protein MGG_17014 [Pyricularia oryzae 70-15]
MGG_17015	29.59778847	127.325793	4.301868471	2.104963415	1.605E-05	0.000403676	uncharacterized protein MGG_17015 [Pyricularia oryzae 70-15]
MGG_17018	20.85194999	66.8181453	3.204407517	1.680057632	0.00165393	0.017456078	uncharacterized protein MGG_17018 [Pyricularia oryzae 70-15]
MGG_17020	20.86550382	0.913837995	0.043796594	-4.513037519	1.23306E-05	0.000325474	uncharacterized protein MGG_17020 [Pyricularia oryzae 70-15]
MGG_17022	0.321175942	20.17450521	62.81449686	5.97302565	2.16724E-05	0.000519282	uncharacterized protein MGG_17022 [Pyricularia oryzae 70-15]
MGG_17025	5.130358167	24.28825604	4.734222301	2.243127451	0.002951292	0.026997209	uncharacterized protein MGG_17025 [Pyricularia oryzae 70-15]
MGG_17027	0.339553209	34.40282978	101.3179346	6.662745762	9.80409E-10	7.4665E-08	uncharacterized protein MGG_17027 [Pyricularia oryzae 70-15]
MGG_17029	27.87682172	84.18996655	3.020070487	1.594582222	0.00195812	0.019922315	uncharacterized protein MGG_17029 [Pyricularia oryzae 70-15]
MGG_17030	4.721813832	15.39600915	3.260613335	1.705143367	0.048585019	0.189221409	uncharacterized protein MGG_17030 [Pyricularia oryzae 70-15]
MGG_17038	2091.905579	941.4089556	0.450024592	-1.151924253	0.004255313	0.03514875	uncharacterized protein MGG_17038 [Pyricularia oryzae 70-15]
MGG_17041	2416.749496	9913.437805	4.101971604	2.036317505	0.000116946	0.002140282	saccharopine dehydrogenase [Pyricularia oryzae 70-15]
MGG_17046	102.0633857	32.25795931	0.316058095	-1.661738327	0.00056037	0.007407369	uncharacterized protein MGG_17046 [Pyricularia oryzae 70-15]
MGG_17047	196.7469917	50.30051883	0.25566093	-1.967696391	1.39693E-05	0.000360295	uncharacterized protein MGG_17047 [Pyricularia oryzae 70-15]
MGG_17051	490.2992546	162.0694092	0.33055202	-1.597050762	0.000142013	0.002489428	uncharacterized protein MGG_17051 [Pyricularia oryzae 70-15]
MGG_17056	151.1669382	57.98200435	0.383562736	-1.38246553	0.002841125	0.026306389	uncharacterized protein MGG_17056 [Pyricularia oryzae 70-15]
MGG_17059	50.23794164	9.595041716	0.190991936	-2.388416371	6.65877E-05	0.001323908	uncharacterized protein MGG_17059 [Pyricularia oryzae 70-15]
MGG_17060	3496.126238	1609.852029	0.460467363	-1.118829191	0.005505441	0.042816967	hypothetical protein, variant [Pyricularia oryzae 70-15]
MGG_17069	59.36668026	24.1520158	0.406827798	-1.297509835	0.015012889	0.085703276	uncharacterized protein MGG_17069 [Pyricularia oryzae 70-15]
MGG_17080	80.52781138	29.38972932	0.364963716	-1.454175052	0.004113904	0.034340252	uncharacterized protein MGG_17080 [Pyricularia oryzae 70-15]
MGG_17088	69.56560887	18.91011994	0.271831444	-1.879215744	0.000404313	0.00574455	uncharacterized protein MGG_17088 [Pyricularia oryzae 70-15]
MGG_17097	5598.912051	1200.471978	0.214411651	-2.22154479	0.01102526	0.069777901	uncharacterized protein MGG_17097 [Pyricularia oryzae 70-15]
MGG_17103	3546.117689	13214.55313	3.726484649	1.897815317	0.003066301	0.027715348	uncharacterized protein MGG_17103 [Pyricularia oryzae 70-15]
MGG_17105	88.42099664	18.8296079	0.212954034	-2.231386038	1.70991E-05	0.000425656	uncharacterized protein MGG_17105 [Pyricularia oryzae 70-15]
MGG_17107	2.166265621	27.14210809	12.52944599	3.64725072	1.16921E-05	0.000313219	uncharacterized protein MGG_17107 [Pyricularia oryzae 70-15]
MGG_17118	128.6823252	22.53813239	0.175145517	-2.513374033	0.039383807	0.164750169	uncharacterized protein MGG_17118 [Pyricularia oryzae 70-15]
MGG_17123	241.9826371	499.5389142	2.064358502	1.045693535	0.013693935	0.080629346	uncharacterized protein MGG_17123 [Pyricularia oryzae 70-15]

MGG_17129	43.36008805	17.56104029	0.405004719	-1.303989376	0.022050633	0.111566466	uncharacterized protein MGG_17129 [Pyricularia oryzae 70-15]
MGG_17133	20.2270909	5.160087566	0.255107746	-1.97082139	0.010020525	0.065551606	uncharacterized protein MGG_17133 [Pyricularia oryzae 70-15]
MGG_17134	84.57103425	16.50273406	0.19513459	-2.357458561	1.1129E-05	0.000301104	uncharacterized protein MGG_17134 [Pyricularia oryzae 70-15]
MGG_17137	56.382747	20.80467502	0.368990092	-1.438346016	0.006458058	0.048291537	uncharacterized protein MGG_17137 [Pyricularia oryzae 70-15]
MGG_17152	187.0832253	61.97381927	0.331263368	-1.593949421	0.000419371	0.005910122	uncharacterized protein MGG_17152 [Pyricularia oryzae 70-15]
MGG_17153	2645.827584	5889.912732	2.226113587	1.154527208	0.005544479	0.042982981	uncharacterized protein MGG_17153 [Pyricularia oryzae 70-15]
MGG_17154	49.19638681	16.48410016	0.335067293	-1.577477227	0.004480828	0.036704722	uncharacterized protein MGG_17154 [Pyricularia oryzae 70-15]
MGG_17155	36.92094093	12.87050247	0.348596275	-1.520370942	0.011006822	0.069713698	uncharacterized protein MGG_17155 [Pyricularia oryzae 70-15]
MGG_17160	20.18099498	5.852172332	0.289984331	-1.785953146	0.016402054	0.090775469	uncharacterized protein MGG_17160 [Pyricularia oryzae 70-15]
MGG_17170	111.5126258	51.19912348	0.459132974	-1.123016048	0.016543842	0.091476826	uncharacterized protein MGG_17170 [Pyricularia oryzae 70-15]
MGG_17175	343.7640958	119.5591355	0.347794132	-1.523694501	0.000377224	0.005435961	uncharacterized protein MGG_17175, partial
MGG_17176	4.336164706	18.53475201	4.27445756	2.09574135	0.013364615	0.07939039	uncharacterized protein MGG_17176, partial
MGG_17182	8.667811471	0.913837995	0.105428919	-3.245657442	0.014335352	0.083203947	uncharacterized protein MGG_17182 [Pyricularia oryzae 70-15]
MGG_17184	1035.143001	391.7115042	0.378412938	-1.40196668	0.0005678	0.007473059	uncharacterized protein MGG_17184 [Pyricularia oryzae 70-15]
MGG_17190	4.162312399	90.91061778	21.84137303	4.448991647	0.000499058	0.006766252	uncharacterized protein MGG_17190 [Pyricularia oryzae 70-15]
MGG_17191	55.37519771	22.63034659	0.408672971	-1.290981264	0.01711184	0.093679418	uncharacterized protein MGG_17191 [Pyricularia oryzae 70-15]
MGG_17194	116.4207513	53.07712269	0.455907749	-1.133186163	0.013981379	0.081735224	uncharacterized protein MGG_17194, partial
MGG_17222	310.7130855	742.9009527	2.39095483	1.257586875	0.002557229	0.024203425	uncharacterized protein MGG_17222 [Pyricularia oryzae 70-15]
MGG_17223	8.736802596	105.0926017	12.02872567	3.588411906	3.0325E-10	2.66948E-08	uncharacterized protein MGG_17223 [Pyricularia oryzae 70-15]
MGG_17232	0	5.680401375	Inf	Inf	0.009999358	0.065488968	uncharacterized protein MGG_17232 [Pyricularia oryzae 70-15]
MGG_17239	7.979363669	543.1393158	68.0679987	6.088904788	1.25921E-10	1.20448E-08	uncharacterized protein MGG_17239 [Pyricularia oryzae 70-15]
MGG_17240	0	15.07851052	Inf	Inf	0.013539957	0.080118556	uncharacterized protein MGG_17240 [Pyricularia oryzae 70-15]
MGG_17265	7337.447123	16848.75871	2.296269864	1.199292201	0.003224986	0.028493392	uncharacterized protein MGG_17265 [Pyricularia oryzae 70-15]
MGG_17266	14.18833671	43.2888163	3.051014167	1.609288879	0.041803474	0.172145288	uncharacterized protein MGG_17266 [Pyricularia oryzae 70-15]
MGG_17273	98.81786274	35.36665005	0.357897338	-1.482382281	0.002533026	0.02402123	uncharacterized protein MGG_17273 [Pyricularia oryzae 70-15]
MGG_17276	56391.39675	5850.803165	0.103753471	-3.268768489	2.80696E-14	5.41253E-12	uncharacterized protein MGG_17276 [Pyricularia oryzae 70-15]
MGG_17280	184.7038773	53.37931435	0.288999425	-1.790861472	0.000378626	0.005449705	uncharacterized protein MGG_17280 [Pyricularia oryzae 70-15]
MGG_17286	9.208935638	25.77609481	2.799030836	1.484927381	0.03556489	0.154565239	uncharacterized protein MGG_17286 [Pyricularia oryzae 70-15]
MGG_17293	1.743861961	9.730500168	5.579856883	2.480228119	0.031249789	0.141861895	uncharacterized protein MGG_17293 [Pyricularia oryzae 70-15]
MGG_17302	0	9.626830928	Inf	Inf	0.004476987	0.036697999	uncharacterized protein MGG_17302 [Pyricularia oryzae 70-15]
MGG_17307	32.67608358	1.184314059	0.036244064	-4.786111465	7.48637E-08	3.63778E-06	uncharacterized protein MGG_17307 [Pyricularia oryzae 70-15]
MGG_17311	22.93624964	1.330482564	0.058007852	-4.107608003	1.19325E-05	0.00031719	uncharacterized protein MGG_17311 [Pyricularia oryzae 70-15]
MGG_17312	92.8212655	35.71593788	0.384781846	-1.377887361	0.005043072	0.039989063	uncharacterized protein MGG_17312 [Pyricularia oryzae 70-15]
MGG_17324	130.1579221	311.5695479	2.393780899	1.25929111	0.003165778	0.028276654	uncharacterized protein MGG_17324 [Pyricularia oryzae 70-15]
MGG_17327	0.321175942	8.672975734	27.00381503	4.755091337	0.01254627	0.076167957	uncharacterized protein MGG_17327, partial
MGG_17330	43.57307485	15.97327378	0.366585875	-1.447776896	0.013067073	0.078467028	uncharacterized protein MGG_17330 [Pyricularia oryzae 70-15]
MGG_17339	47.44406794	7.970962703	0.168007573	-2.573401831	3.49592E-05	0.000782108	uncharacterized protein MGG_17339 [Pyricularia oryzae 70-15]
MGG_17365	6086.734533	1576.259601	0.258966379	-1.949163288	1.47165E-05	0.000375637	uncharacterized protein MGG_17365 [Pyricularia oryzae 70-15]
MGG_17369	13.37182701	2.221200175	0.166110448	-2.589785277	0.032487899	0.145869337	uncharacterized protein MGG_17369, partial
MGG_17373	266.6023609	554.923794	2.081466166	1.057600108	0.009697171	0.063745254	uncharacterized protein MGG_17373 [Pyricularia oryzae 70-15]
MGG_17383	53.53136154	25.79425107	0.481853073	-1.053334789	0.043436305	0.176005415	uncharacterized protein MGG_17383 [Pyricularia oryzae 70-15]
MGG_17384	418.4959014	189.9256996	0.453829294	-1.139778358	0.0066363	0.04910664	uncharacterized protein MGG_17384 [Pyricularia oryzae 70-15]
MGG_17391	62.06384419	16.23178035	0.261533596	-1.934931811	0.000363161	0.005315283	uncharacterized protein MGG_17391 [Pyricularia oryzae 70-15]
MGG_17392	20.49401952	0.61775948	0.030143403	-5.052013914	4.82232E-06	0.00014711	uncharacterized protein MGG_17392 [Pyricularia oryzae 70-15]
MGG_17397	67.17100165	32.37277944	0.48194576	-1.053057305	0.033649506	0.149296642	uncharacterized protein MGG_17397 [Pyricularia oryzae 70-15]
MGG_17399	1599.555005	3802.402789	2.377162884	1.249240761	0.002117456	0.021098803	adenine phosphoribosyltransferase [Pyricularia oryzae 70-15]
MGG_17410	96.03730089	40.78884899	0.424718819	-1.235420061	0.027413375	0.129970404	uncharacterized protein MGG_17410 [Pyricularia oryzae 70-15]
MGG_17414	539.0258442	124.2905091	0.23058358	-2.116638311	9.26405E-07	3.51687E-05	uncharacterized protein MGG_17414 [Pyricularia oryzae 70-15]

MGG_17416	142.3588154	50.80267639	0.356863579	-1.486555425	0.033779051	0.149738831	uncharacterized protein MGG_17416 [Pyricularia oryzae 70-15]
MGG_17420	99.71703178	18.04378216	0.180949852	-2.466338166	0.015235226	0.086484826	uncharacterized protein MGG_17420 [Pyricularia oryzae 70-15]
MGG_17439	780.9908736	1694.797065	2.170060013	1.117734941	0.006122224	0.046251725	deoxyuridine 5'-triphosphate nucleotidohydrolase
MGG_17444	4.231303525	15.37411139	3.633422018	1.861328943	0.03163484	0.143128507	uncharacterized protein MGG_17444 [Pyricularia oryzae 70-15]
MGG_17447	33.18045322	76.87236766	2.316796795	1.212131512	0.018225064	0.097927425	uncharacterized protein MGG_17447 [Pyricularia oryzae 70-15]
MGG_17455	671.0418691	1716.235396	2.557568276	1.354772754	0.000716834	0.009033301	uncharacterized protein MGG_17455 [Pyricularia oryzae 70-15]
MGG_17462	58.5574375	171.9495416	2.93642531	1.554060942	0.000715895	0.009030832	uncharacterized protein MGG_17462 [Pyricularia oryzae 70-15]
MGG_17463	1.693248103	10.60506594	6.263149462	2.646888308	0.048447536	0.188876979	uncharacterized protein MGG_17463 [Pyricularia oryzae 70-15]
MGG_17464	86.62621532	35.09613717	0.405144529	-1.303491437	0.006764791	0.049624809	uncharacterized protein MGG_17464 [Pyricularia oryzae 70-15]
MGG_17469	411.2521244	179.1536166	0.435629644	-1.198825964	0.003877545	0.032779691	uncharacterized protein MGG_17469 [Pyricularia oryzae 70-15]
MGG_17473	545.4856724	55.1265151	0.101059511	-3.306722996	2.01934E-05	0.000488665	uncharacterized protein MGG_17473 [Pyricularia oryzae 70-15]
MGG_17474	187.1627157	24.7131286	0.132040874	-2.9209435	1.77269E-08	1.03532E-06	uncharacterized protein MGG_17474 [Pyricularia oryzae 70-15]
MGG_17479	246.6309419	81.98607775	0.332424136	-1.588902961	0.000330032	0.004931407	uncharacterized protein MGG_17479 [Pyricularia oryzae 70-15]
MGG_17485	0	17.90461901	Inf	Inf	0.000608584	0.007919333	uncharacterized protein MGG_17485 [Pyricularia oryzae 70-15]
MGG_17486	211.8007659	791.8840804	3.738815943	1.902581451	0.00032011	0.004813541	uncharacterized protein MGG_17486 [Pyricularia oryzae 70-15]
MGG_17489	34.77543254	73.75218508	2.120812876	1.084617334	0.031074687	0.14127818	uncharacterized protein MGG_17489 [Pyricularia oryzae 70-15]
MGG_17507	132.3489475	34.74470822	0.262523495	-1.929481551	6.47334E-05	0.001298927	uncharacterized protein MGG_17507 [Pyricularia oryzae 70-15]
MGG_17512	9.246574648	0.888235544	0.096061036	-3.379904816	0.011211115	0.070456611	uncharacterized protein MGG_17512 [Pyricularia oryzae 70-15]
MGG_17513	13284.03839	27567.61684	2.07524369	1.053280758	0.009030984	0.060780275	delta-1-pyrroline-5-carboxylate dehydrogenase
MGG_17520	130.2658378	59.97933645	0.460437959	-1.11892132	0.013686497	0.080629346	uncharacterized protein MGG_17520 [Pyricularia oryzae 70-15]
MGG_17524	24.91391915	5.994636142	0.240613936	-2.055207893	0.00541568	0.042199921	uncharacterized protein MGG_17524 [Pyricularia oryzae 70-15]
MGG_17527	117.7613443	43.18674744	0.366731101	-1.447205475	0.002449273	0.023483637	uncharacterized protein MGG_17527 [Pyricularia oryzae 70-15]
MGG_17528	104.7349055	39.50052474	0.377147662	-1.406798612	0.003001146	0.027273682	uncharacterized protein MGG_17528 [Pyricularia oryzae 70-15]
MGG_17536	39.7870702	113.6371874	2.856133585	1.514063457	0.045401333	0.18167245	uncharacterized protein MGG_17536 [Pyricularia oryzae 70-15]
MGG_17540	917.9167997	319.8872165	0.348492605	-1.520800053	0.002720092	0.025496669	uncharacterized protein MGG_17540 [Pyricularia oryzae 70-15]
MGG_17550	0	12.93738156	Inf	Inf	4.17056E-05	0.000901495	uncharacterized protein MGG_17550 [Pyricularia oryzae 70-15]
MGG_17578	623.7979533	156.2663007	0.250507877	-1.997072129	1.34043E-05	0.000347939	uncharacterized protein MGG_17578 [Pyricularia oryzae 70-15]
MGG_17595	347.0349071	839.7347367	2.419741413	1.274852882	0.031829226	0.143793766	uncharacterized protein MGG_17595 [Pyricularia oryzae 70-15]
MGG_17602	0	4.789683765	Inf	Inf	0.021610814	0.110167086	uncharacterized protein MGG_17602 [Pyricularia oryzae 70-15]
MGG_17607	14.85327829	165.2659478	11.12656375	3.475936204	2.54257E-11	2.73338E-09	uncharacterized protein MGG_17607 [Pyricularia oryzae 70-15]
MGG_17608	9.173065582	120.7217253	13.16045592	3.718137565	0.000121372	0.00219409	uncharacterized protein MGG_17608 [Pyricularia oryzae 70-15]
MGG_17616	18.87064702	5.278171554	0.279702734	-1.838033742	0.019453782	0.102526919	uncharacterized protein MGG_17616 [Pyricularia oryzae 70-15]
MGG_17620	45.02894547	0.592157029	0.013150586	-6.248729078	7.20378E-07	2.80486E-05	uncharacterized protein MGG_17620 [Pyricularia oryzae 70-15]
MGG_17622	96.8595185	206.1098799	2.127925919	1.089447926	0.014265425	0.082956622	uncharacterized protein MGG_17622 [Pyricularia oryzae 70-15]
MGG_17649	0	62.24895532	Inf	Inf	6.70162E-16	1.69607E-13	uncharacterized protein MGG_17649 [Pyricularia oryzae 70-15]
MGG_17652	33.53053779	232.8738339	6.945126718	2.796001017	4.07526E-09	2.75035E-07	uncharacterized protein MGG_17652 [Pyricularia oryzae 70-15]
MGG_17656	30.71315787	115.7214024	3.767811921	1.913726951	0.04846657	0.188890565	uncharacterized protein MGG_17656 [Pyricularia oryzae 70-15]
MGG_17672	68.05592349	30.23791085	0.444309757	-1.170362271	0.02192329	0.111212671	uncharacterized protein MGG_17672 [Pyricularia oryzae 70-15]
MGG_17678	994.6836401	472.5413419	0.475066969	-1.073797194	0.010703966	0.068294001	uncharacterized protein MGG_17678 [Pyricularia oryzae 70-15]
MGG_17688	389.4700417	168.2737555	0.432058278	-1.210702172	0.00386481	0.032740386	uncharacterized protein MGG_17688 [Pyricularia oryzae 70-15]
MGG_17689	98.63079361	45.25651084	0.45884768	-1.123912781	0.017089316	0.093656418	uncharacterized protein MGG_17689 [Pyricularia oryzae 70-15]
MGG_17720	98.21528785	47.01060652	0.478648564	-1.062961313	0.023029651	0.114892894	uncharacterized protein MGG_17720 [Pyricularia oryzae 70-15]
MGG_17723	28.68243102	11.51642187	0.401514846	-1.316474763	0.040499958	0.168202904	uncharacterized protein MGG_17723 [Pyricularia oryzae 70-15]
MGG_17726	17.47027723	5.037039445	0.288320522	-1.794254568	0.0285045	0.132915008	uncharacterized protein MGG_17726 [Pyricularia oryzae 70-15]
MGG_17730	1031.63167	489.7064969	0.474691221	-1.074938726	0.009588901	0.063170268	uncharacterized protein MGG_17730 [Pyricularia oryzae 70-15]
MGG_17731	115.8491789	631.217024	5.448610255	2.445888297	0.00020537	0.003371395	uncharacterized protein MGG_17731 [Pyricularia oryzae 70-15]
MGG_17738	73.35368799	16.54152863	0.225503708	-2.148776939	0.001792358	0.018609888	uncharacterized protein MGG_17738 [Pyricularia oryzae 70-15]
MGG_17745	1.963810186	43.20956369	22.00292268	4.459623267	1.52475E-08	9.03543E-07	uncharacterized protein MGG_17745 [Pyricularia oryzae 70-15]

MGG_17746	14.18833671	57.13262646	4.02673179	2.009609381	0.000462998	0.006411947	uncharacterized protein MGG_17746 [Pyricularia oryzae 70-15]
MGG_17750	1.674870836	9.106553927	5.437167888	2.442855376	0.038795426	0.163244489	uncharacterized protein MGG_17750 [Pyricularia oryzae 70-15]
MGG_17752	2.73962638	43.8275905	15.99765239	3.999788304	0.000273495	0.004275956	uncharacterized protein MGG_17752 [Pyricularia oryzae 70-15]
MGG_17762	2.45068703	11.08162105	4.521842616	2.17691078	0.03692387	0.157868822	uncharacterized protein MGG_17762 [Pyricularia oryzae 70-15]
MGG_17765	90.28161904	14.62585759	0.162002606	-2.625911071	4.61974E-06	0.000141719	uncharacterized protein MGG_17765 [Pyricularia oryzae 70-15]
MGG_17778	1206.964582	3581.221592	2.96713064	1.569068449	0.000124889	0.002240985	uncharacterized protein MGG_17778 [Pyricularia oryzae 70-15]
MGG_17791	7.282764462	420.7907253	57.77898317	5.85247291	2.07838E-08	1.18536E-06	uncharacterized protein MGG_17791 [Pyricularia oryzae 70-15]
MGG_17792	16.3554868	445.851962	27.26008509	4.76871816	0.000272938	0.004272748	uncharacterized protein MGG_17792 [Pyricularia oryzae 70-15]
MGG_17805	9.343284425	44.68298771	4.782364068	2.257723963	0.00132561	0.014706404	uncharacterized protein MGG_17805 [Pyricularia oryzae 70-15]
MGG_17807	28.18376931	143.6584871	5.097206324	2.349706752	0.01051786	0.06763613	uncharacterized protein MGG_17807 [Pyricularia oryzae 70-15]
MGG_17809	2.473582238	14.30914076	5.78478473	2.532263273	0.008517774	0.058262344	uncharacterized protein MGG_17809 [Pyricularia oryzae 70-15]
MGG_17822	183.790028	1215.02196	6.610924291	2.724851993	2.93106E-10	2.61364E-08	uncharacterized protein MGG_17822 [Pyricularia oryzae 70-15]
MGG_17830	1480.071759	3825.507457	2.584677015	1.369984011	0.004385009	0.036041336	uncharacterized protein MGG_17830 [Pyricularia oryzae 70-15]
MGG_17840	4.446733808	15.03305154	3.380695178	1.757319941	0.042604996	0.174030093	uncharacterized protein MGG_17840 [Pyricularia oryzae 70-15]
MGG_17843	39.18754979	13.96930354	0.356473003	-1.488135276	0.010248029	0.066396299	uncharacterized protein MGG_17843 [Pyricularia oryzae 70-15]
MGG_17844	42.84009014	286.0824955	6.67791535	2.739397806	0.005917811	0.045299038	uncharacterized protein MGG_17844 [Pyricularia oryzae 70-15]
MGG_17847	6.428921261	0.296078515	0.046054152	-4.440524973	0.015502252	0.087266618	uncharacterized protein MGG_17847 [Pyricularia oryzae 70-15]
MGG_17858	99.4041028	437.650608	4.402741895	2.138402271	8.82223E-05	0.001687754	uncharacterized protein MGG_17858 [Pyricularia oryzae 70-15]
MGG_17860	258.3572653	1220.73704	4.724995981	2.240313102	0.002288597	0.022383658	uncharacterized protein MGG_17860 [Pyricularia oryzae 70-15]
MGG_17864	253.9224687	858.7280024	3.381851188	1.757813178	3.08854E-05	0.000706584	glycosyl hydrolase family 88 [Pyricularia oryzae 70-15]
MGG_17865	10.48066456	39.41300734	3.760544679	1.910941638	0.002347752	0.022725488	uncharacterized protein MGG_17865 [Pyricularia oryzae 70-15]
MGG_17884	107.3392986	2125.894314	19.80536805	4.307819606	1.89487E-06	6.6337E-05	uncharacterized protein MGG_17884 [Pyricularia oryzae 70-15]
MGG_17890	68.46446782	27.57276338	0.402730997	-1.312111579	0.011347694	0.071057623	uncharacterized protein MGG_17890 [Pyricularia oryzae 70-15]
MGG_17903	124.9712422	755.1846243	6.042867231	2.595233245	1.8713E-09	1.33254E-07	uncharacterized protein MGG_17903 [Pyricularia oryzae 70-15]
MGG_17911	37.68252877	7.177690763	0.190477948	-2.39230411	0.006110307	0.046190425	uncharacterized protein MGG_17911 [Pyricularia oryzae 70-15]
MGG_17922	440.6885394	84.06733299	0.190763602	-2.390142168	6.57966E-08	3.23602E-06	uncharacterized protein MGG_17922 [Pyricularia oryzae 70-15]
MGG_17928	355.0080348	835.9820968	2.354825849	1.235620369	0.003527077	0.030346867	uncharacterized protein MGG_17928 [Pyricularia oryzae 70-15]
MGG_17938	20.46297291	2.174959406	0.106287557	-3.233955379	0.001550889	0.016599299	uncharacterized protein MGG_17938 [Pyricularia oryzae 70-15]
MGG_17940	161.155387	60.70920348	0.376712219	-1.408465265	0.004256158	0.03514875	uncharacterized protein MGG_17940 [Pyricularia oryzae 70-15]
MGG_17968	38.35867626	16.03562957	0.418044394	-1.258271938	0.028327197	0.13265952	uncharacterized protein MGG_17968 [Pyricularia oryzae 70-15]
MGG_17973	567.8698272	170.1932154	0.299704628	-1.738386732	5.10667E-05	0.001067742	uncharacterized protein MGG_17973 [Pyricularia oryzae 70-15]
MGG_17983	1762.605434	370.2433066	0.210054559	-2.251163998	1.06748E-06	4.00239E-05	MFS monocarboxylate transporter [Pyricularia oryzae 70-15]
MGG_17986	249.0788799	83.40992353	0.334873529	-1.578311756	0.000262941	0.004148322	uncharacterized protein MGG_17986 [Pyricularia oryzae 70-15]
MGG_17990	584.7492697	224.6005367	0.384097165	-1.38045678	0.002513655	0.02388884	uncharacterized protein MGG_17990 [Pyricularia oryzae 70-15]
MGG_17992	32.95293256	9.498818674	0.288254123	-1.794586852	0.004755791	0.038464279	uncharacterized protein MGG_17992 [Pyricularia oryzae 70-15]
MGG_17994	168.9671853	24.4720333	0.144833053	-2.787537211	1.60338E-08	9.45529E-07	uncharacterized protein MGG_17994 [Pyricularia oryzae 70-15]
MGG_17995	159.3132563	72.9408305	0.457845331	-1.127067786	0.011498269	0.071594554	uncharacterized protein MGG_17995 [Pyricularia oryzae 70-15]
MGG_18002	1474.527938	517.0115186	0.3506285	-1.51198483	0.000169497	0.002871753	uncharacterized protein MGG_18002 [Pyricularia oryzae 70-15]
MGG_18003	2626.514947	5372.185182	2.045366309	1.032359241	0.021433389	0.109630657	ATP synthase subunit D [Pyricularia oryzae 70-15]
MGG_18013	19.54767898	3.551682739	0.181693322	-2.460422696	0.002703149	0.025372091	uncharacterized protein MGG_18013 [Pyricularia oryzae 70-15]
MGG_18016	790.6284938	350.943283	0.443878871	-1.171762059	0.008024929	0.05574274	uncharacterized protein MGG_18016 [Pyricularia oryzae 70-15]
MGG_18017	2584.764226	1162.119041	0.449603499	-1.15327483	0.004158202	0.034645977	uncharacterized protein MGG_18017 [Pyricularia oryzae 70-15]
MGG_18021	533.3335218	1393.818909	2.613409531	1.385933218	0.011391546	0.07117943	hypothetical protein, variant [Pyricularia oryzae 70-15]
MGG_18022	67.55274384	23.76175547	0.351751152	-1.507372946	0.004061055	0.03402324	uncharacterized protein MGG_18022 [Pyricularia oryzae 70-15]
MGG_18029	4.786287017	18.90393318	3.949602921	1.981707617	0.014362531	0.083321883	uncharacterized protein MGG_18029 [Pyricularia oryzae 70-15]
MGG_18036	1.963810186	28.92973008	14.73142887	3.880825466	0.003202391	0.028458407	uncharacterized protein MGG_18036 [Pyricularia oryzae 70-15]
MGG_18041	1.436545345	13.35528556	9.296807519	3.216735386	0.002498154	0.023839418	uncharacterized protein MGG_18041 [Pyricularia oryzae 70-15]
MGG_18042	1.808335146	30.84570526	17.05751577	4.092335645	1.02614E-06	3.85929E-05	uncharacterized protein MGG_18042 [Pyricularia oryzae 70-15]

MGG_18046	343.6891233	128.7990435	0.374754494	-1.415982315	0.000960319	0.011411609	uncharacterized protein MGG_18046 [Pyricularia oryzae 70-15]
MGG_18061	10.77412185	49.62410825	4.605861058	2.203470891	0.000267615	0.004216587	uncharacterized protein MGG_18061 [Pyricularia oryzae 70-15]
MGG_18095	2.046660637	21.64344275	10.57500318	3.402586192	0.00554404	0.042982981	uncharacterized protein MGG_18095 [Pyricularia oryzae 70-15]
MGG_18114	79.88271051	205.4792017	2.572261261	1.363037183	0.024635852	0.120239587	uncharacterized protein MGG_18114, partial
MGG_18115	102.8029628	217.0379231	2.111202995	1.078065302	0.012245049	0.074622274	uncharacterized protein MGG_18115 [Pyricularia oryzae 70-15]
MGG_18116	4.16683034	23.43555121	5.624311359	2.491676464	0.001438134	0.015598615	uncharacterized protein MGG_18116 [Pyricularia oryzae 70-15]
MGG_18137	1.014141685	11.6106405	11.44873608	3.51711643	0.003208537	0.028471369	uncharacterized protein MGG_18137 [Pyricularia oryzae 70-15]

Table S3. Primers used in this study.

Primers	Sequences (5' to 3')
CLP1-upF	ACTGACACTCTAGAGTCGACCCTACCCACTACGTCACTAAAC
CLP1-upR	CATTCATTGTTGACCTCCACTAGTCGGTAGAGGTTGGAGAATG
CLP1-dnF	GGGCAAAGGAATAGAGTAGATGCAATCACTTTCTTGGCGTAACC
CLP1-dnR	GGCCAGTGCCAAGCTTGGGCAAGTCATCACTACTCAA
HPH-F	TAGTGGAGGTCAACAATGAATG
HPH-R	CTATTCCTTTGCCCTCGGACGA
CLP1-S-F	CTGCCAAGGAAGCGTTAGAT
CLP1-S-R	ATTTCGTAGTTGCGAGGGTAAT
CLP1-LF	TGACCCTCCATTTTAGCTGC
HPH-CKR	GGGCGAACTTAAGAAGGTATGA
Tbl-gF	TTCCGCGCTGTCACCGTTCC
Tbl-gR	GGGCCTCCTCCTCGTACTCCTCTT
qtub-F	ACAACTTCGTCTTCGGTCAG
qtub-R	GTGATCTGGAAACCCTGGAG
qHPH-F	ATGTCCTGCGGGTAAATAGC
qHPH-R	GATGCAATAGGTCAGGCTCTC
CLP1c-F	GTCACCGAGATTTAGGAATTCGACAGCCCACTACAACTCAA
CLP1c-R	TTACTGCAGGTCGACTCTAGAGGCTGTCTGTTACCACATCT
qCLP1-RT-F	CCTTCAGTGTGATGTTTGCA
qCLP1-RT-R	TCCTTGCTGATTGTGTGTCTA
qTbl-RT-F	TTCCGCGCTGTCACCGTTCC
qTbl-RT-R	GGGCCTCCTCCTCGTACTCCTCTT
PHD-1-R	CAACGGAGATCTTGTGTAGGTCGACAGATTCGTCGTCCTCTTGG
PHD-2-F	GACCTACACAAGATCTCCGTTG
RT-CLP1-PHD1-F	CCAAGAGGGTAAAACGAGGTC
RT-CLP1-PHD1-R	GACCATCGTCAGAGTCATCTTG
40S-qF	ACAAGCTCAAGACCCTCGTC
40S-qR	GGTGGTGATGGTGAAGCAG
α-ACTIN-qF	ACAATGGTTCGGGTATGTGC
α-ACTIN-qR	CGACAATGGACGGGAAGAC
pKD3-CLP1-GFP-F	TCAATCACAATGGCCGGATCCATGGGAGAATCAGACACAAGA
PKD3-CLP1-GFP-R	GCCCTTGCTCACCATCCCGGGTGACCTCGTATCGACACCAAAT
pKD8-H2B-mCherry-F	ATGGTCGGATCCATCCCCGGGATGCCCCCCAAGGCCGCTGACAA

pKD8-H2B-mCherry-R	TTACTGCAGGTCGACTCTAGAGCCGCCGGTGGAGTGGCGGCC
pKD8-ATG5-RED-F	GTCAAAATGGTCGGATCCATGGCTTCGCCGCGCCGATCAG
pKD8-ATG5-RED-R	GGAGGAGGCCATCCCGGGTAATGGCACGACGGTTAAACAA
pKD8-ATG7-RED-F	ACCGTCAAAATGGTCGGATCCATGTCCGGAAATGATGAGGCG
pKD8-ATG7-RED-R	CTCGGAGGAGGCCATCCCGGGAAGCATCTCACCATCCCCTTC
pKD8-ATG16-RED-F	GTCAAAATGGTCGGATCCATGTCTTCGCTGCCGGACTGGC
pKD8-ATG16-RED-R	GGAGGAGGCCATCCCGGGTCCTCGTTTGGCGAATTTCGGC
pKD8-ATG24-mCherry-F	ACCGTCAAAATGGTCGGATCC ATGGGGGGAATCGACCAAGAC
pKD8-ATG24-mCherry-R	GCCCTTGCTCACCATCCCGGGAGCAGCCACGGCGCCCTCCTT
pKD8-ATG28-mCherry-F	ACCGTCAAAATGGTCGGATCCATGGCTTCCAAGTCATCCTTC
pKD8-ATG28-mCherry-R	GCCCTTGCTCACCATCCCGGGATCCTGATGCTCTCTGAGGAAC
pKD7-ATG5-3×FLAG-F	TCAATCACAATGGCCGGATCCATGGCTTCGCCGCGCCGATCAG
pKD7-ATG5-3×FLAG-R	GTGGTCCTTGTAGTCCCCGGGTAATGGCACGACGGTTAAACA
pKD7-ATG7-3×FLAG-F	TCAATCACAATGGCCGGATCC ATGTCCGGAAATGATGAGGCG
pKD7-ATG7-3×FLAG-R	GTGGTCCTTGTAGTCCCCGGGAAGCATCTCACCATCCCCTTC
pKD7-ATG16-3×FLAG-F	TCAATCACAATGGCCGGATCC ATGTCTTCGCTGCCGGACTGGC
pKD7-ATG16-3×FLAG-R	GTGGTCCTTGTAGTCCCCGGGTCCTCGTTTGGCGAATTTCGGC
CLP1-BDF	ATGGCCATGGAGGCCGAATTCATGGGAGAATCAGACACAAG
CLP1-BDR	TCGACGGATCCCCGGGAATTCTTATGACCTCGTATCGACACC
Cyc8-ADF	GCCATGGAGGCCAGTGAATTCATGGCGTCGCATCGGCCGT
Cyc8-ADR	ATGCCCACCCGGGTGGAATTCTTAGGAGCTCTCAACCTTGG
Rpd3-ADF	GCCATGGAGGCCAGTGAATTCATGACGGACAAGGCGGGCGTGG
Rpd3-ADR	ATGCCCACCCGGGTGGAATTCTTATGGCTCTGCCTCCTCTGTG
Sin3-ADF	GCCATGGAGGCCAGTGAATTCATGAATTCTCAACGGTCCCACG
Sin3-ADR	ATGCCCACCCGGGTGGAATTCTTATGCAGAAGACCCAGCACCGC
ATG5-ADF	GCCATGGAGGCCAGTGAATTCATGGCTTCGCCGCGCCGATCAG
ATG5-ADR	ATGCCCACCCGGGTGGAATTCTAATGGCACGACGGTTAAACA
ATG7-ADF	GCCATGGAGGCCAGTGAATTC ATGTCCGGAAATGATGAGGCG
ATG7-ADR	ATGCCCACCCGGGTGGAATTCAAGCATCTCACCATCCCCTTC
ATG16-ADF	GCCATGGAGGCCAGTGAATTCATGTCTTCGCTGCCGGACTGGC
ATG16-ADR	ATGCCCACCCGGGTGGAATTCTCCTCGTTTGGCGAATTTCGGC
ATG24-ADF	GCCATGGAGGCCAGTGAATTCATGGGGGGAATCGACCAAGAC
ATG24-ADR	ATGCCCACCCGGGTGGAATTCAGCAGCCACGGCGCCCTCCTT
ATG28-ADF	GCCATGGAGGCCAGTGAATTCATGGCTTCCAAGTCATCCTTC
ATG28-ADR	ATGCCCACCCGGGTGGAATTCATCCTGATGCTCTCTGAGGAAC
pET21a-AT5-F	GATGATGACGACAAGGTCGACATGGCTTCGCCGCGCCGATCAG

pET21a-ATG5-R	CTCGAGTGCGGCCGCAAGCTTTAATGGCACGACGGTTAAACA
pET21a-ATG7-F	GATGATGACGACAAGGTCGACATGTCCGGAAATGATGAGGCG
pET21a-ATG7-R	CTCGAGTGCGGCCGCAAGCTTAAGCATCTCACCATCCCCTTC
pET21a-AT16-F	GATGATGACGACAAGGTCGACATGTCTTCGCTGCCGGACTGGC
pET21a-ATG16-R	CTCGAGTGCGGCCGCAAGCTTTCCTCGTTTGGCGAATTTCGGC
pET21a-ATG24 -F	GATGATGACGACAAGGTCGAC ATGGGGGGAATCGACCAAGAC
pET21a- ATG24-R	CTCGAGTGCGGCCGCAAGCTTAGCAGCCACGGCGCCCTCCTT
pET21a-ATG28 -F	GATGATGACGACAAGGTCGACATGGCTTCCAAGTCATCCTTC
pET21a- ATG28-R	CTCGAGTGCGGCCGCAAGCTTATCCTGATGCTCTCTGAGGAAC
qPig1-F	TGTGGACAGAAGTTTACTCGC
qPig1-R	CTCGTGGTATGCTTGGTAGTG
qALB1-F	ACTAAACGAGCGGTATCATGC
qALB1-R	GGTAGGTTTTGTCATGCTGTG
qRSY-F	GTTCAAAAGAGCGATGAGATAACC
qRSY-R	GCGCAGAGTAGGCGCAATGACC
qBuf1-F	AGAGCAAGTATGACGCGATC
qBuf1-R	GTTGACAATGACCTTGCAGC
RT-MSTU1-F	GATCAGGGTCACAATAACGGG
RT-MSTU1-R	ACTAGAGATGGACGAGACAGG
RT-FLBC-F	TCCCTCAACTACGACCCTTAC
RT-FLBC-R	GTGAGCTGCCAGAGTAGATAC
qCNF1-F	ACCTTTCGACTACCGCAAC
qCNF1-R	CAGTTCATACATTTGCCATCGG
RT-GTA1-F	AAGACATCCACGACCAGTTC
RT-GTA1-R	GTAACGTGGTTCCTGGTAGC
RT-GCC1-F	CCAAGTTTGAAAGCTCCGAG
RT-GCC1-R	GTGAACAAAGGGCAAGGAAG
RT-COS1-F	CACAACCATTCAAAGACGCAC
RT-COS1-R	AGAGTTGACTTTGGCGATGAG
RT-HOX2-F	GGACTGCCTCTCAATGGTATG
RT-HOX2-R	AGGTGTGGGAGCAATTATCG
RT-CONX2-F	GCCCGAGAGCAAGAGTAATAC
RT-CONX2-R	CTGCTTCATGTACGGGTAGTC
qCON7-F	ACCATTTAAACGCGCATGTG
qCON7-R	CGTTCCTCGTCTGCCTTG
qSPF1-F	CGACCTCAGCCAAACATTTTC

qSPF1-R	TTCTGCTTCGAGATCAACCG
qHAC1-F	AATCAACGCCTACACCCG
qHAC1-R	GCTCCTTCTCATCTTCTGTCTTG
qKAR2-F	AACGGTCTCGAGAACTATGC
qKAR2-R	TCCTTCTGCTCCTCAAAATC
qREI1-F	GCCAAAGCATTCTTGTCTACG
qREI1-R	ACTGGTTGAACTGAAGCTCTC
qMBF1-F	CATCTACAGTGCGACCTACAG
qMBF1-R	GTTCTTGCGGGTTTGTCAAAG
qCREA-F	CGCCAATGATAGAGACCAACA
qCREA-R	GGGTGTTGCAAGATGAAGATC
qTea1-F	CTTACAAAACAGCGACAGTGG
qTea1-R	TCGTAACACCAGACATCGTTG
qArf6-F	TGGAGGCATTACTTTAGCGG
qArf6-R	GCAAACACCAACAGCAAGC
qSpa2-F	TGAACGGTGATTCTGGCAAG
qSpa2-R	GTCTGGTCCTCGACAAAGATC
RT-CLP1-PHD-F	CCAAGAGGGTAAAACGAGGTC
RT-CLP1-PHD-R	GACCATCGTCAGAGTCATCTTG
